# Supplementary figures and images for: Targeting the WSB2–NOXA axis in cancer cells for enhanced sensitivity to BCL-2 family protein inhibitors (part 2 of 5)
Source: eLife. 2025 Jul 23;13:RP98372. doi: 10.7554/eLife.98372 (PMC12286604; doi:10.7554/eLife.98372)

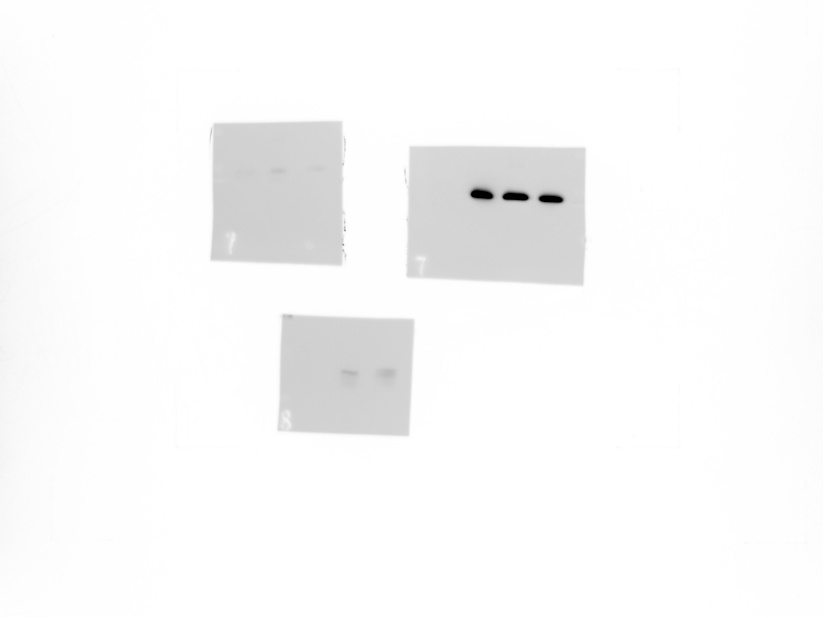

Supplement: Figure 2—figure supplement 1—source data 1. [file elife-98372-fig2-figsupp1-data1.zip › Figure 2-supplementary figure 1-data1/Figure_2-figure supplement_1_ source_data_1_ Figure_A_FLAG(BAD).jpg]

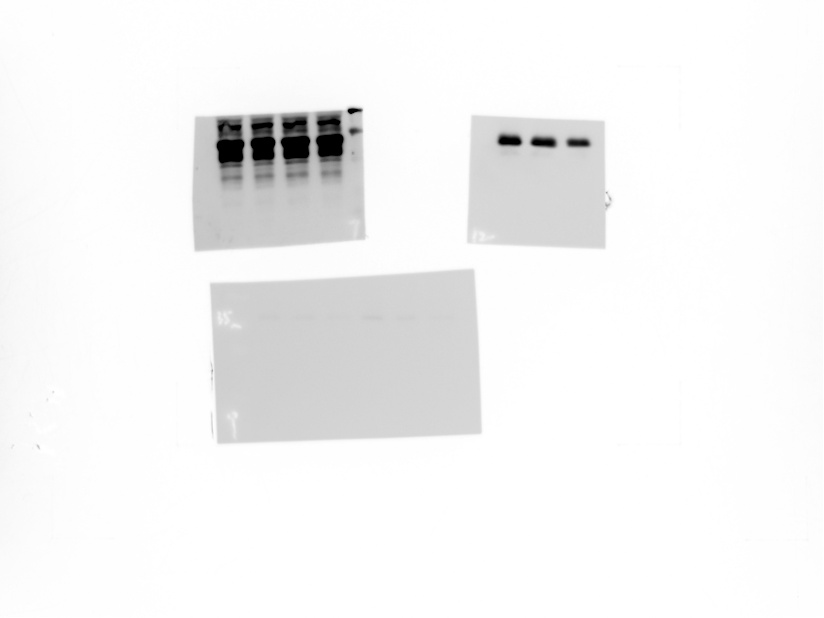

Supplement: Figure 2—figure supplement 1—source data 1. [file elife-98372-fig2-figsupp1-data1.zip › Figure 2-supplementary figure 1-data1/Figure_2-figure supplement_1_ source_data_1_ Figure_A_FLAG(BAX).jpg]

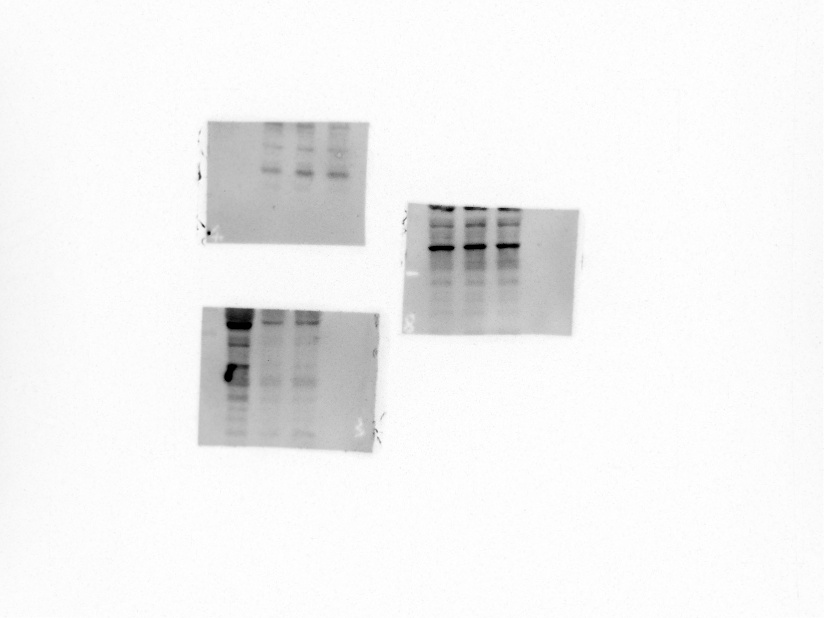

Supplement: Figure 2—figure supplement 1—source data 1. [file elife-98372-fig2-figsupp1-data1.zip › Figure 2-supplementary figure 1-data1/Figure_2-figure supplement_1_ source_data_1_ Figure_A_FLAG(BCL-2).jpg]

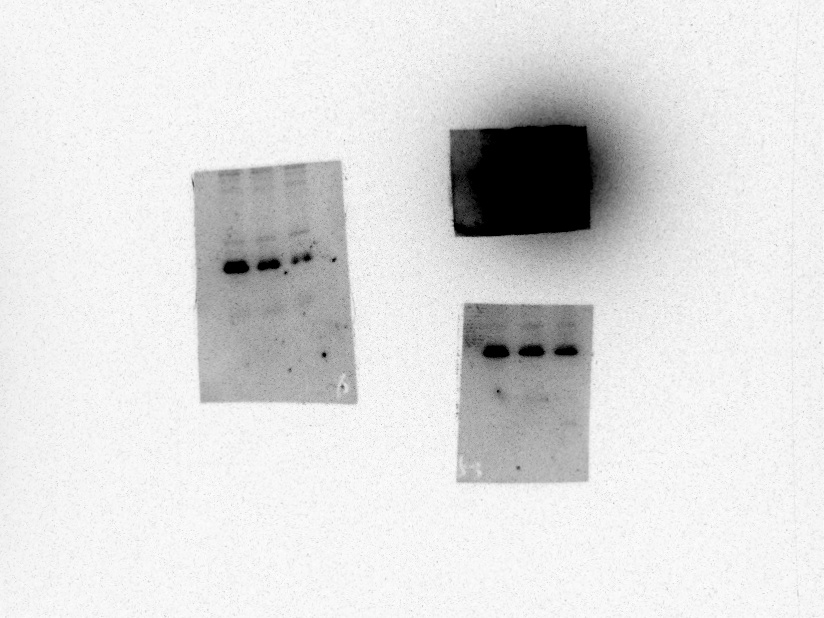

Supplement: Figure 2—figure supplement 1—source data 1. [file elife-98372-fig2-figsupp1-data1.zip › Figure 2-supplementary figure 1-data1/Figure_2-figure supplement_1_ source_data_1_ Figure_A_FLAG(BCL-W).jpg]

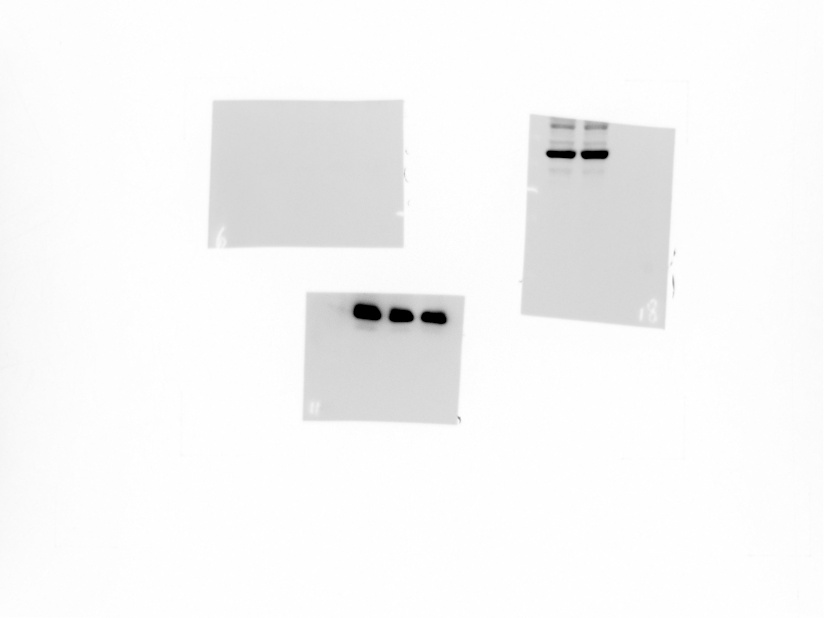

Supplement: Figure 2—figure supplement 1—source data 1. [file elife-98372-fig2-figsupp1-data1.zip › Figure 2-supplementary figure 1-data1/Figure_2-figure supplement_1_ source_data_1_ Figure_A_FLAG(Bcl-xl).jpg]

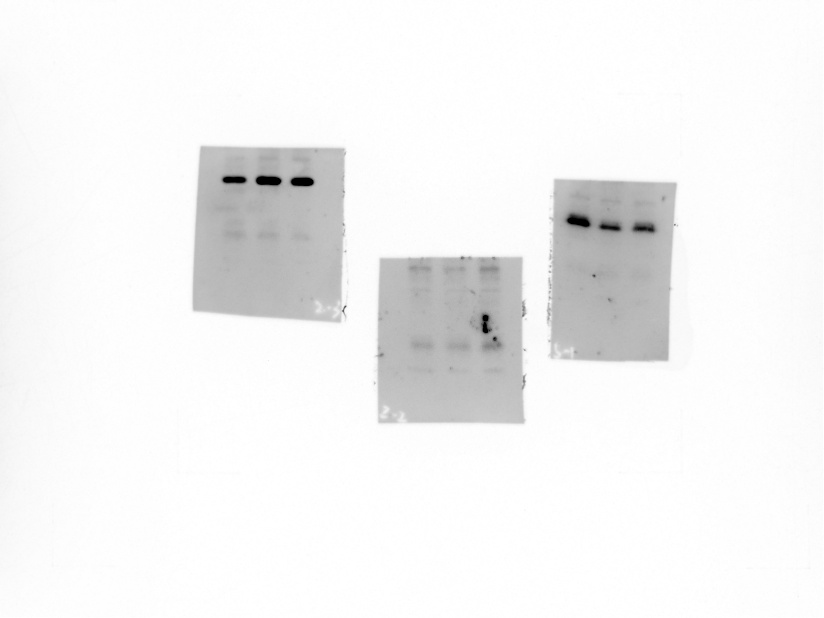

Supplement: Figure 2—figure supplement 1—source data 1. [file elife-98372-fig2-figsupp1-data1.zip › Figure 2-supplementary figure 1-data1/Figure_2-figure supplement_1_ source_data_1_ Figure_A_FLAG(MCL-1).jpg]

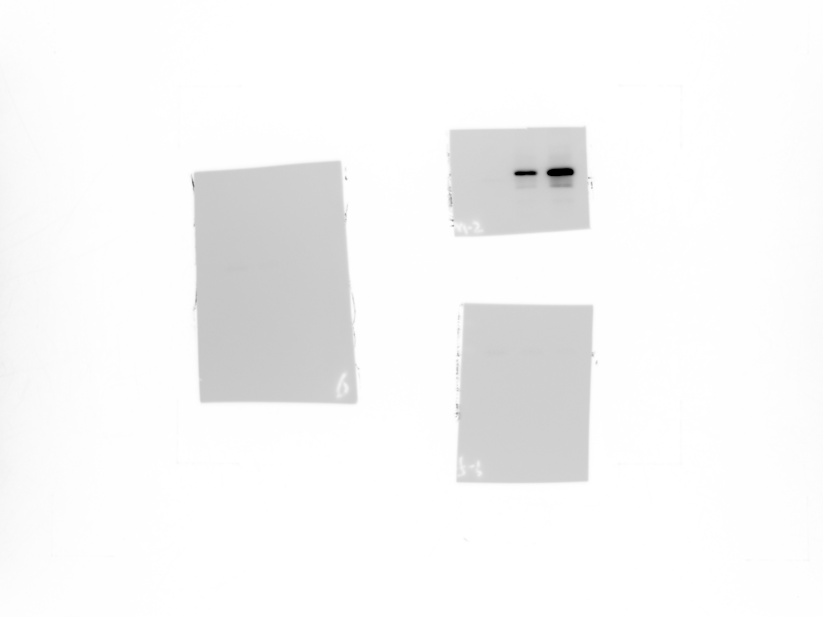

Supplement: Figure 2—figure supplement 1—source data 1. [file elife-98372-fig2-figsupp1-data1.zip › Figure 2-supplementary figure 1-data1/Figure_2-figure supplement_1_ source_data_1_ Figure_A_Myc(BAD).jpg]

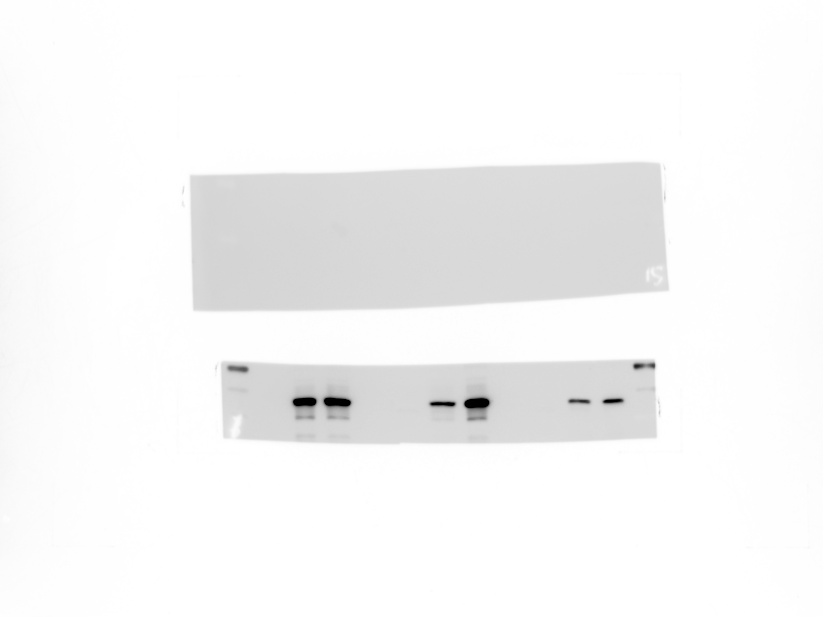

Supplement: Figure 2—figure supplement 1—source data 1. [file elife-98372-fig2-figsupp1-data1.zip › Figure 2-supplementary figure 1-data1/Figure_2-figure supplement_1_ source_data_1_ Figure_A_Myc(BAX).jpg]

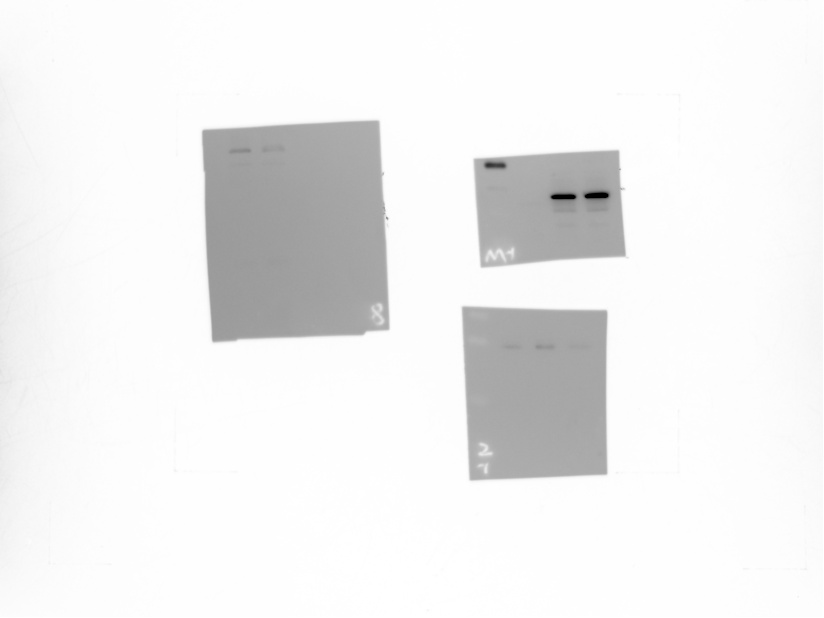

Supplement: Figure 2—figure supplement 1—source data 1. [file elife-98372-fig2-figsupp1-data1.zip › Figure 2-supplementary figure 1-data1/Figure_2-figure supplement_1_ source_data_1_ Figure_A_Myc(BCL-2).jpg]

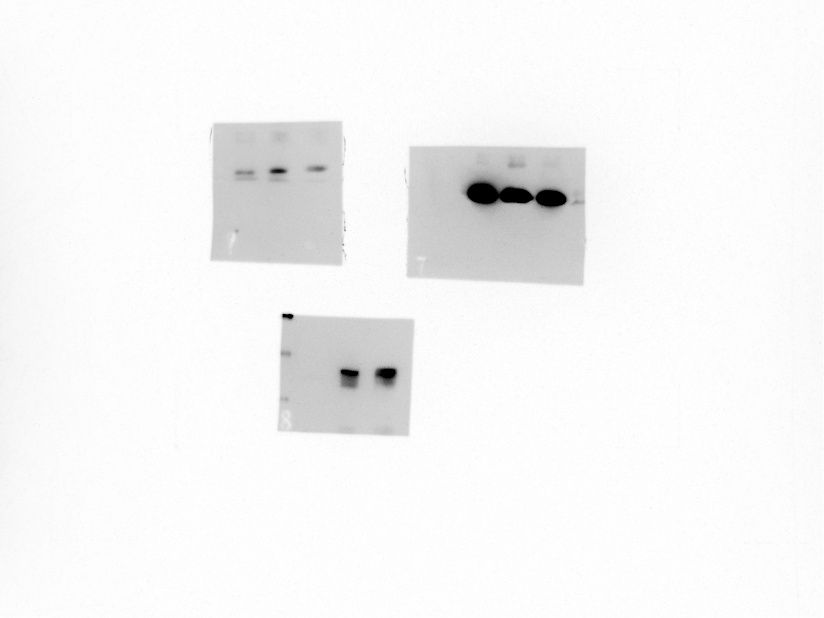

Supplement: Figure 2—figure supplement 1—source data 1. [file elife-98372-fig2-figsupp1-data1.zip › Figure 2-supplementary figure 1-data1/Figure_2-figure supplement_1_ source_data_1_ Figure_A_Myc(BCL-XL).jpg]

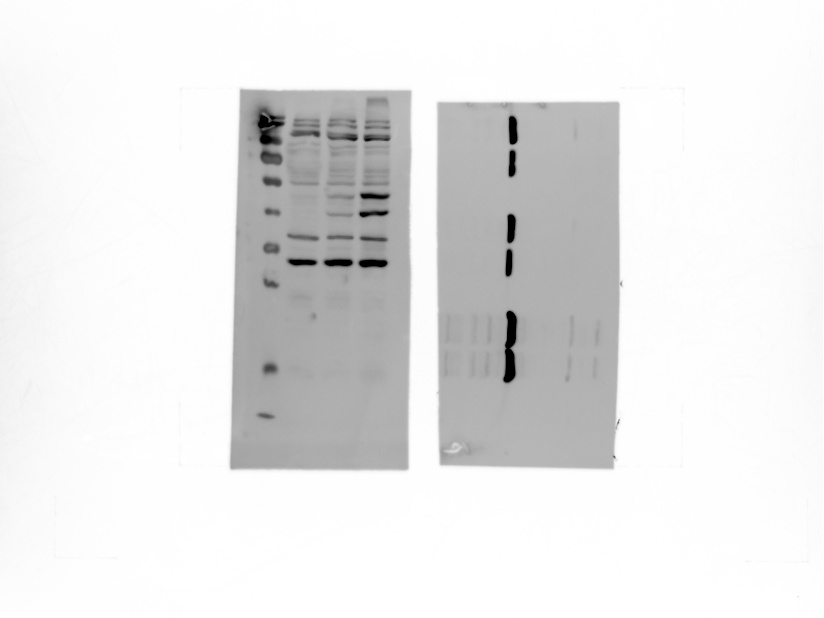

Supplement: Figure 2—figure supplement 1—source data 1. [file elife-98372-fig2-figsupp1-data1.zip › Figure 2-supplementary figure 1-data1/Figure_2-figure supplement_1_ source_data_1_ Figure_A_Myc(NOXA).jpg]

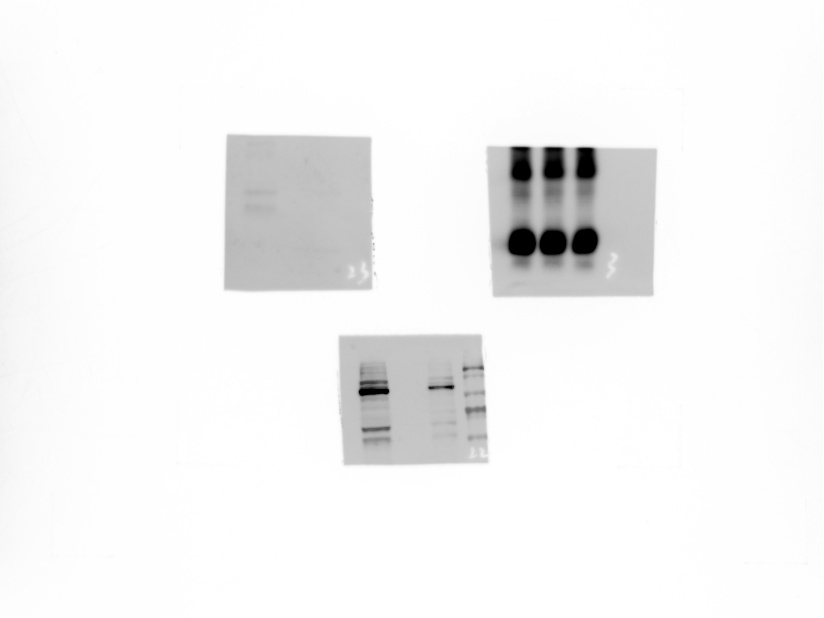

Supplement: Figure 2—figure supplement 1—source data 1. [file elife-98372-fig2-figsupp1-data1.zip › Figure 2-supplementary figure 1-data1/Figure_2-figure supplement_1_ source_data_1_ Figure_C_CUL5.jpg]

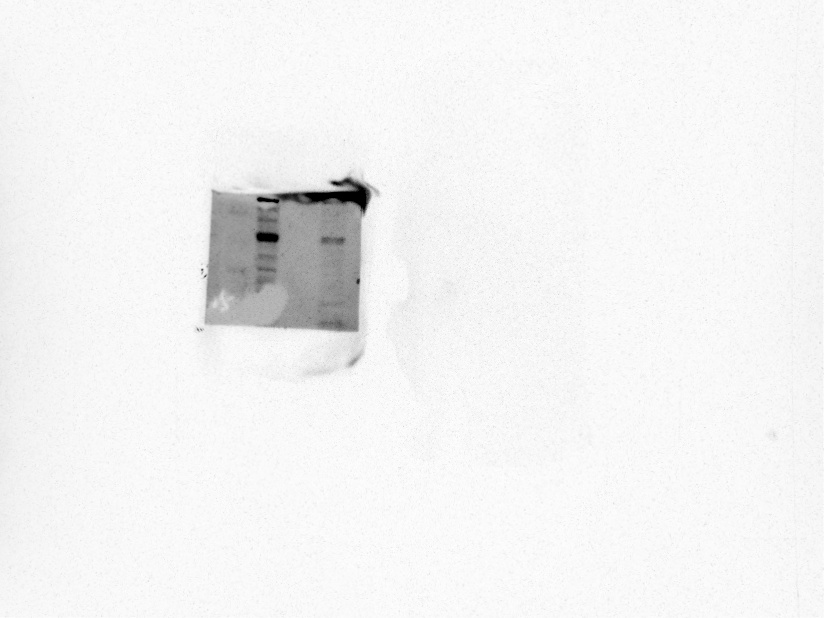

Supplement: Figure 2—figure supplement 1—source data 1. [file elife-98372-fig2-figsupp1-data1.zip › Figure 2-supplementary figure 1-data1/Figure_2-figure supplement_1_ source_data_1_ Figure_C_ELOB.jpg]

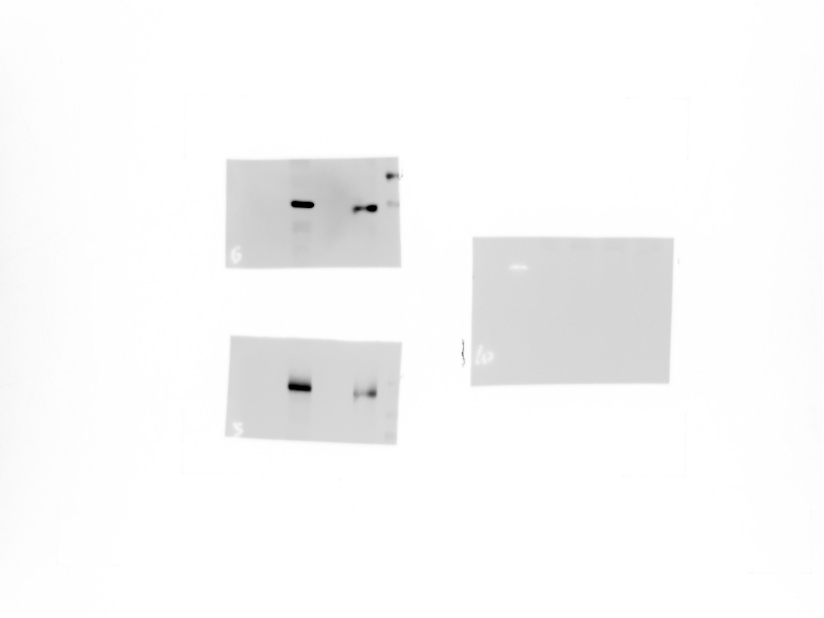

Supplement: Figure 2—figure supplement 1—source data 1. [file elife-98372-fig2-figsupp1-data1.zip › Figure 2-supplementary figure 1-data1/Figure_2-figure supplement_1_ source_data_1_ Figure_C_ELOC.jpg]

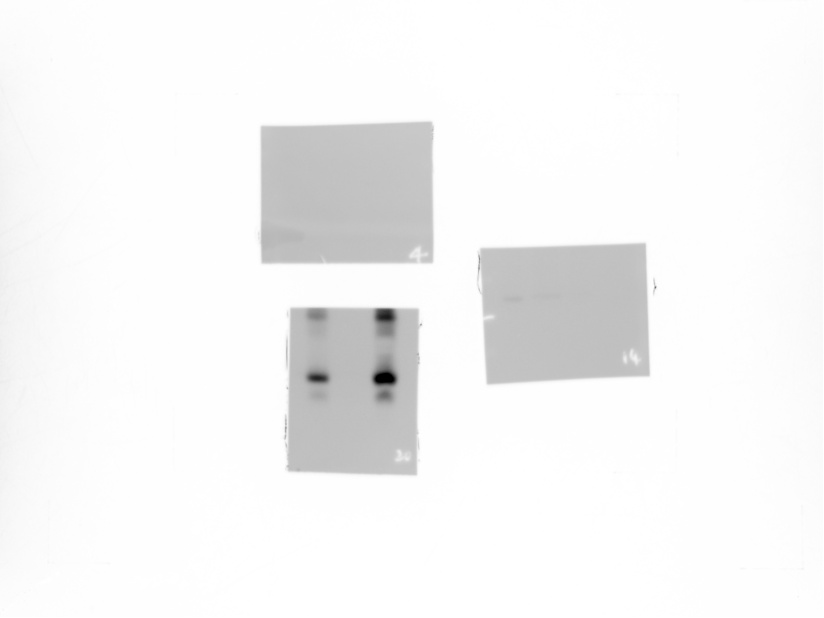

Supplement: Figure 2—figure supplement 1—source data 1. [file elife-98372-fig2-figsupp1-data1.zip › Figure 2-supplementary figure 1-data1/Figure_2-figure supplement_1_ source_data_1_ Figure_C_NOXA.jpg]

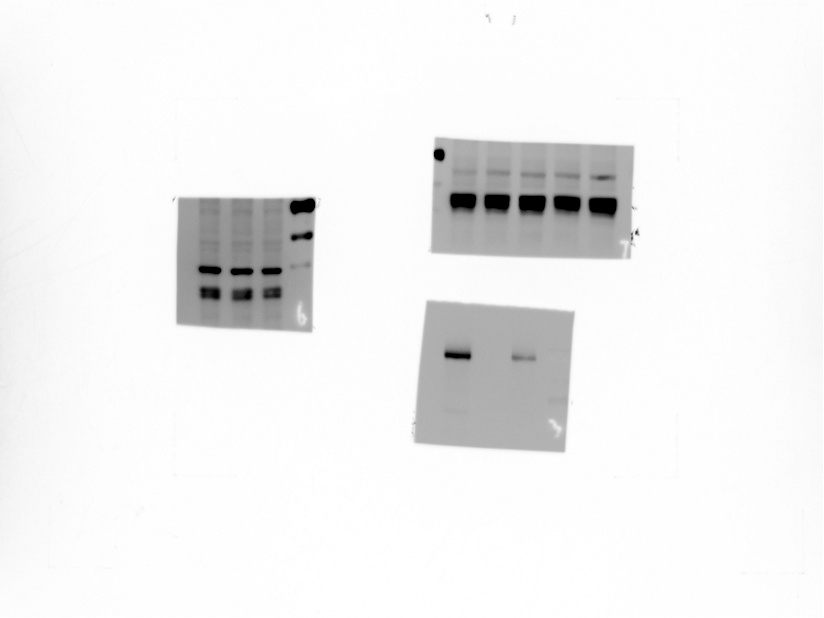

Supplement: Figure 2—figure supplement 1—source data 1. [file elife-98372-fig2-figsupp1-data1.zip › Figure 2-supplementary figure 1-data1/Figure_2-figure supplement_1_ source_data_1_ Figure_C_RBX2.jpg]

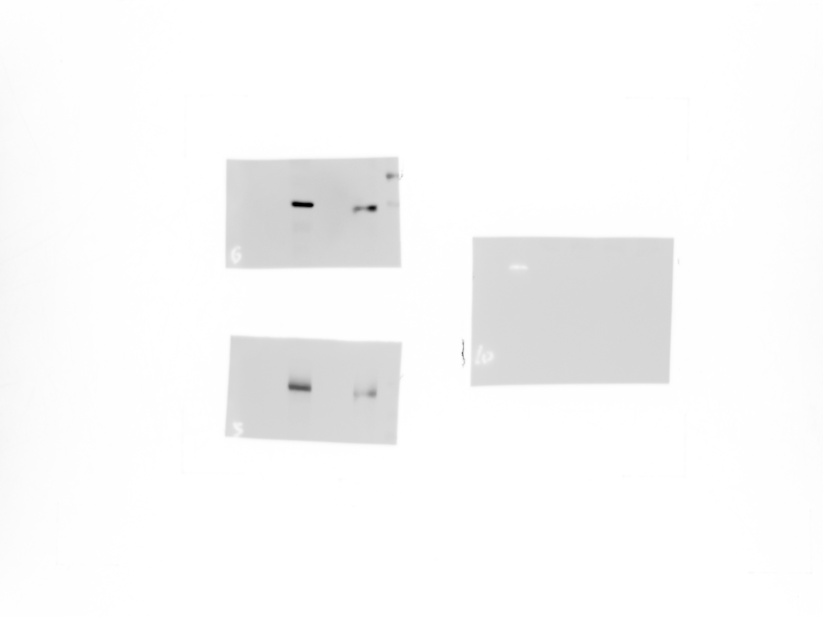

Supplement: Figure 2—figure supplement 1—source data 1. [file elife-98372-fig2-figsupp1-data1.zip › Figure 2-supplementary figure 1-data1/Figure_2-figure supplement_1_ source_data_1_ Figure_C_WSB2.jpg]

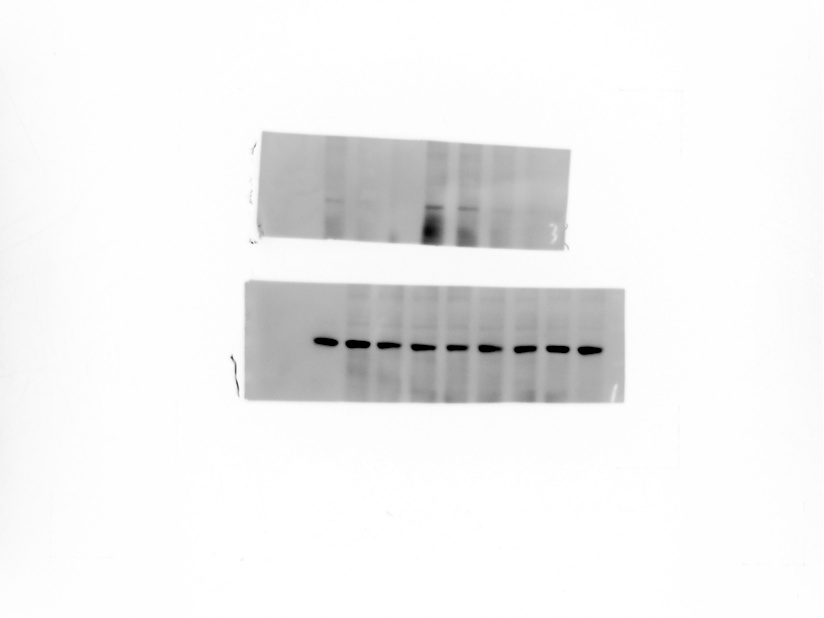

Supplement: Figure 2—figure supplement 1—source data 1. [file elife-98372-fig2-figsupp1-data1.zip › Figure 2-supplementary figure 1-data1/Figure_2-figure supplement_1_ source_data_1_ Figure_D_Actin.jpg]

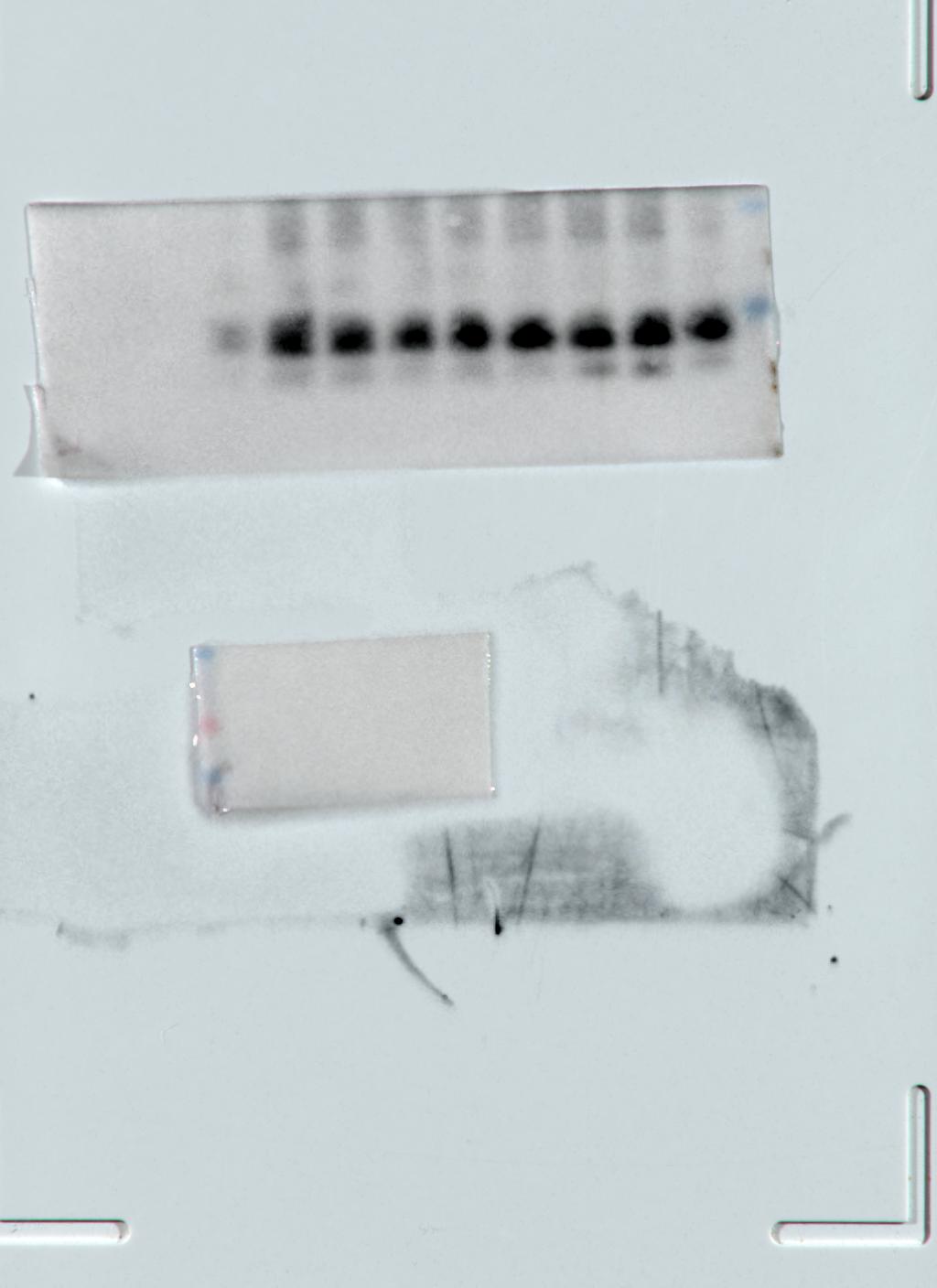

Supplement: Figure 2—figure supplement 1—source data 1. [file elife-98372-fig2-figsupp1-data1.zip › Figure 2-supplementary figure 1-data1/Figure_2-figure supplement_1_ source_data_1_ Figure_D_NOXA.jpg]

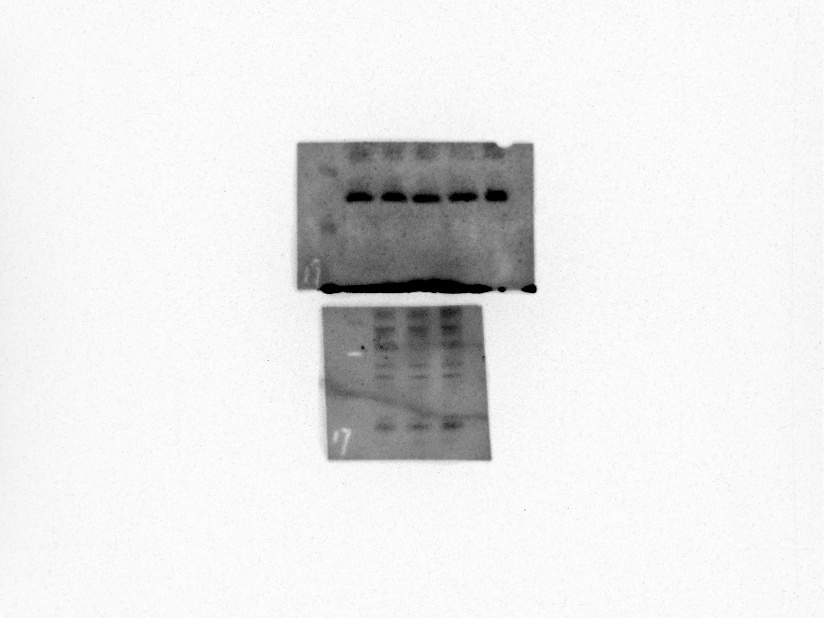

Supplement: Figure 2—figure supplement 1—source data 1. [file elife-98372-fig2-figsupp1-data1.zip › Figure 2-supplementary figure 1-data1/Figure_2-figure supplement_1_ source_data_1_ Figure_E_FLAG(left).jpg]

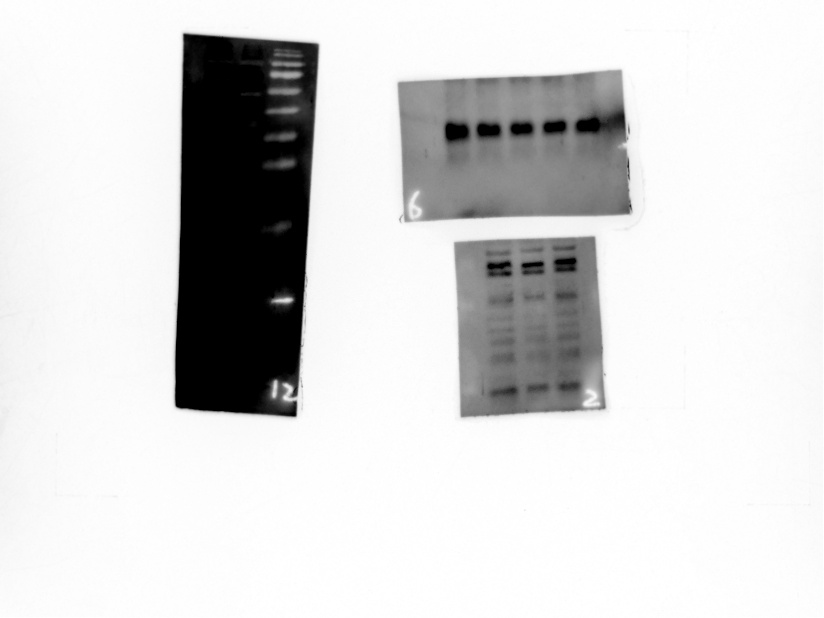

Supplement: Figure 2—figure supplement 1—source data 1. [file elife-98372-fig2-figsupp1-data1.zip › Figure 2-supplementary figure 1-data1/Figure_2-figure supplement_1_ source_data_1_ Figure_E_FLAG(right).jpg]

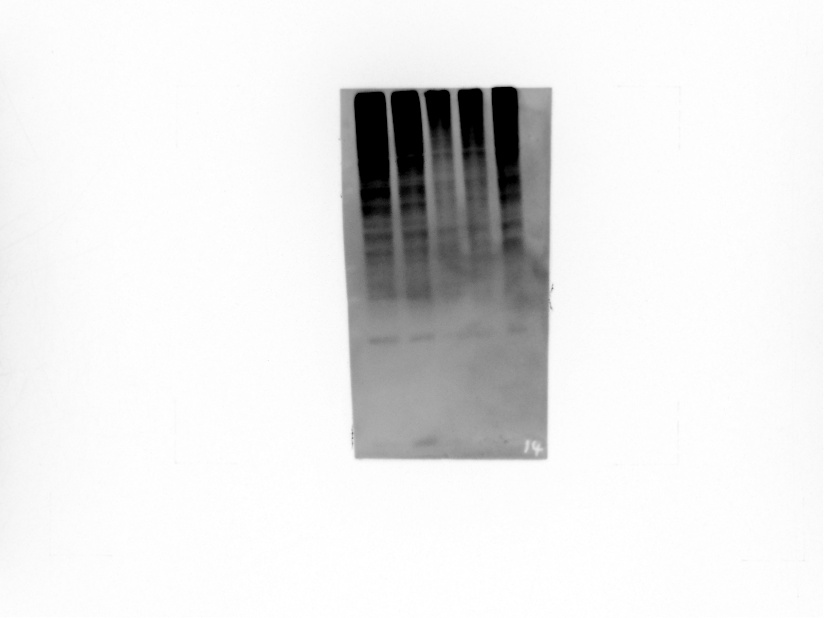

Supplement: Figure 2—figure supplement 1—source data 1. [file elife-98372-fig2-figsupp1-data1.zip › Figure 2-supplementary figure 1-data1/Figure_2-figure supplement_1_ source_data_1_ Figure_E_HA(left).jpg]

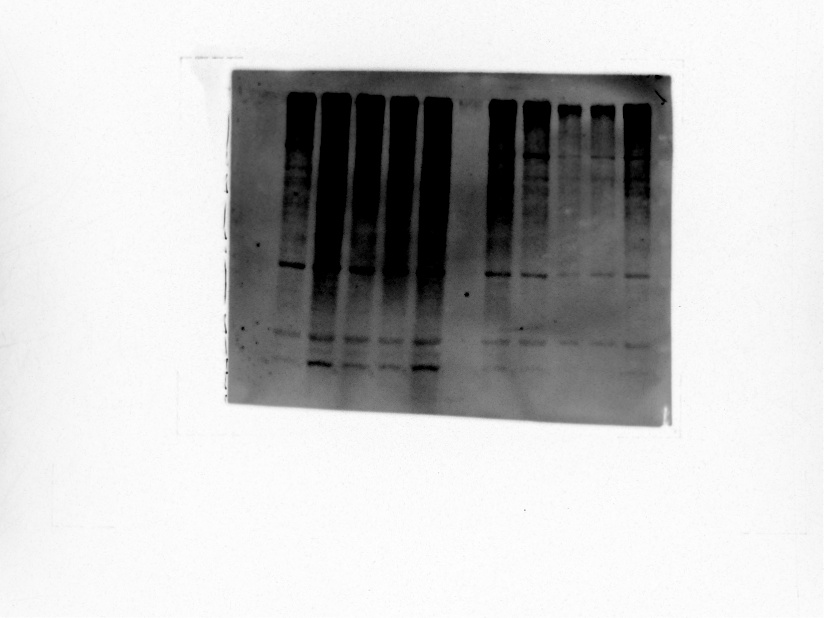

Supplement: Figure 2—figure supplement 1—source data 1. [file elife-98372-fig2-figsupp1-data1.zip › Figure 2-supplementary figure 1-data1/Figure_2-figure supplement_1_ source_data_1_ Figure_E_HA(right).jpg]

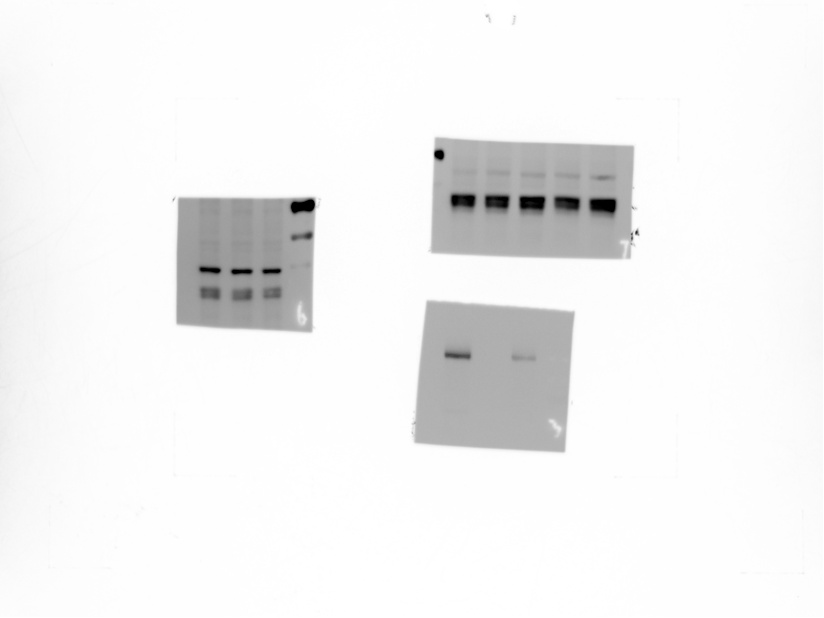

Supplement: Figure 2—figure supplement 1—source data 1. [file elife-98372-fig2-figsupp1-data1.zip › Figure 2-supplementary figure 1-data1/Figure_2-figure supplement_1_ source_data_1_ Figure_E_Myc(left).jpg]

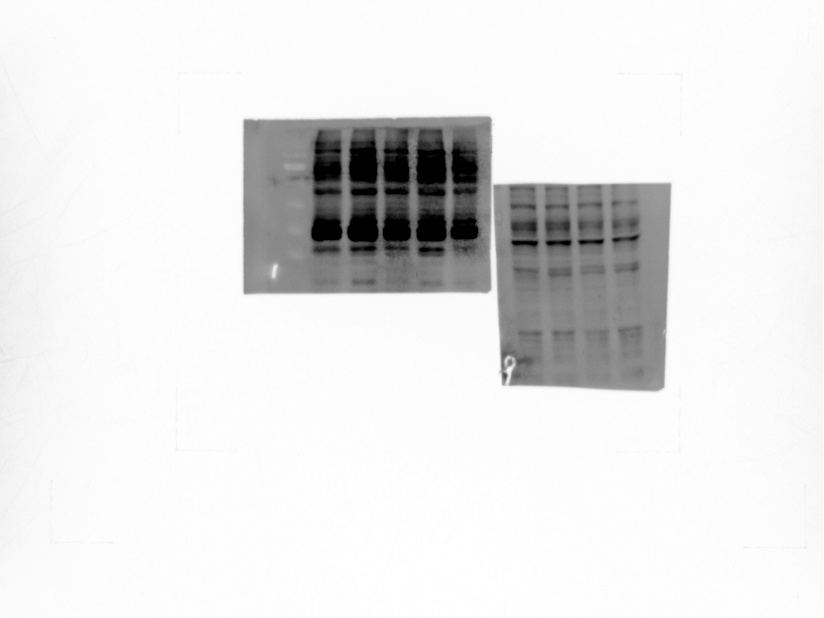

Supplement: Figure 2—figure supplement 1—source data 1. [file elife-98372-fig2-figsupp1-data1.zip › Figure 2-supplementary figure 1-data1/Figure_2-figure supplement_1_ source_data_1_ Figure_E_Myc(right).jpg]

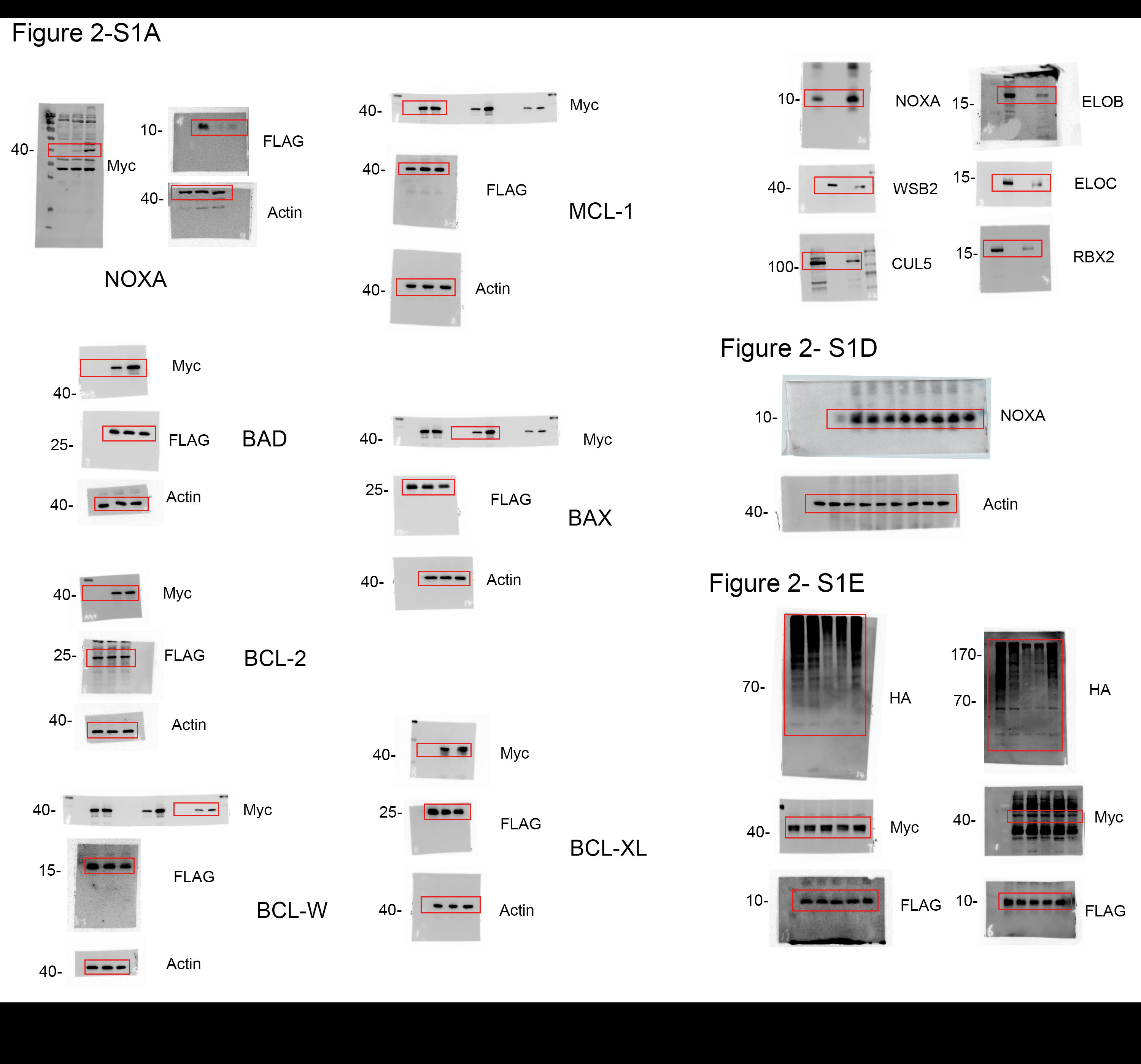

Supplement: Figure 2—figure supplement 1—source data 2. [file elife-98372-fig2-figsupp1-data2.zip › Figure 2-supplementary figure 1-data2/Figure_2_supplement figure_1_data_2 .jpg]

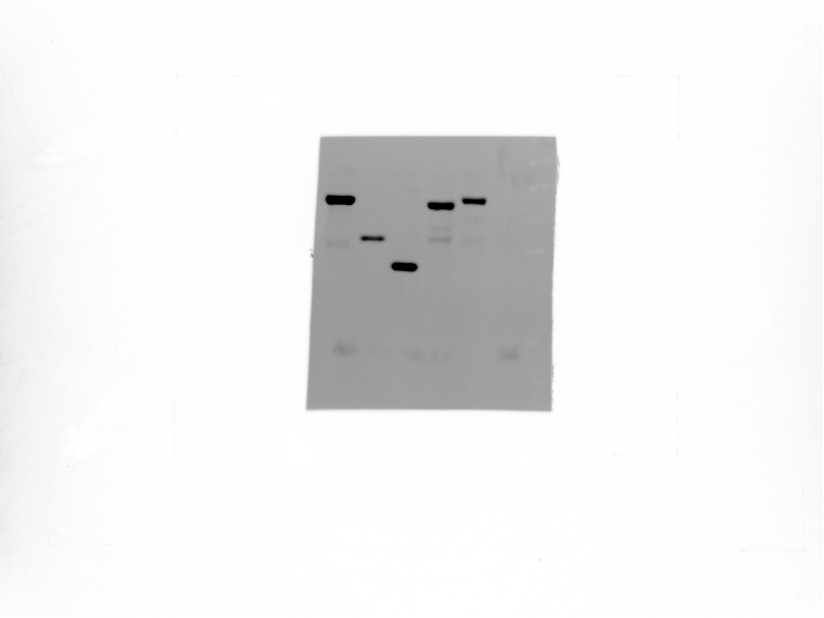

Supplement: Figure 3—source data 1. [file elife-98372-fig3-data1.zip › Figure 3-data1/Figure_3-source_data_1_ Figure_3B_IP-FLAG.jpg]

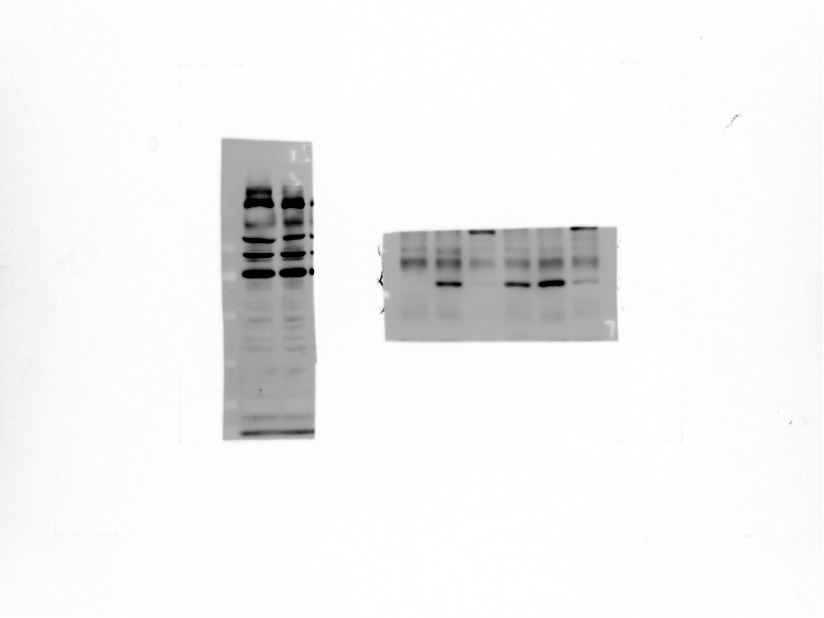

Supplement: Figure 3—source data 1. [file elife-98372-fig3-data1.zip › Figure 3-data1/Figure_3-source_data_1_ Figure_3B_IP-GFP.jpg]

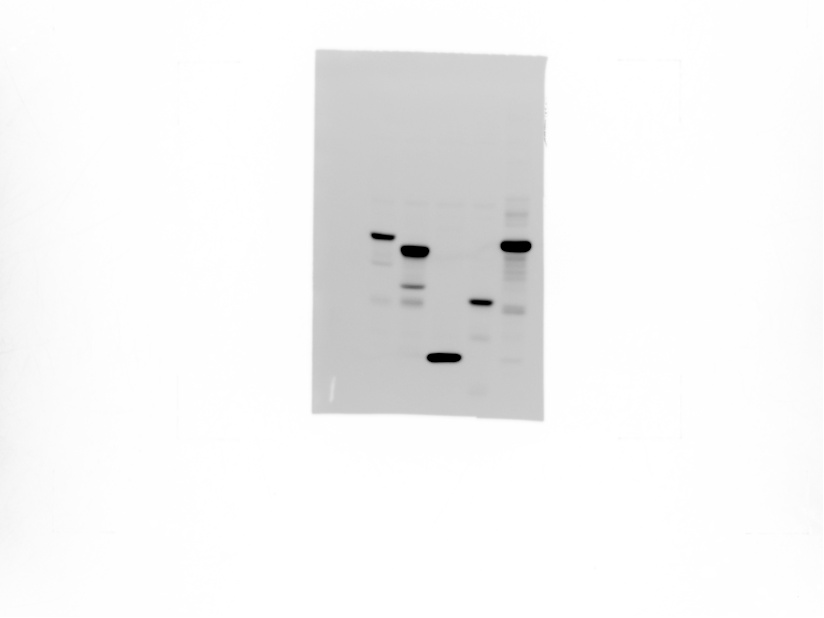

Supplement: Figure 3—source data 1. [file elife-98372-fig3-data1.zip › Figure 3-data1/Figure_3-source_data_1_ Figure_3B_WCL-FLAG.jpg]

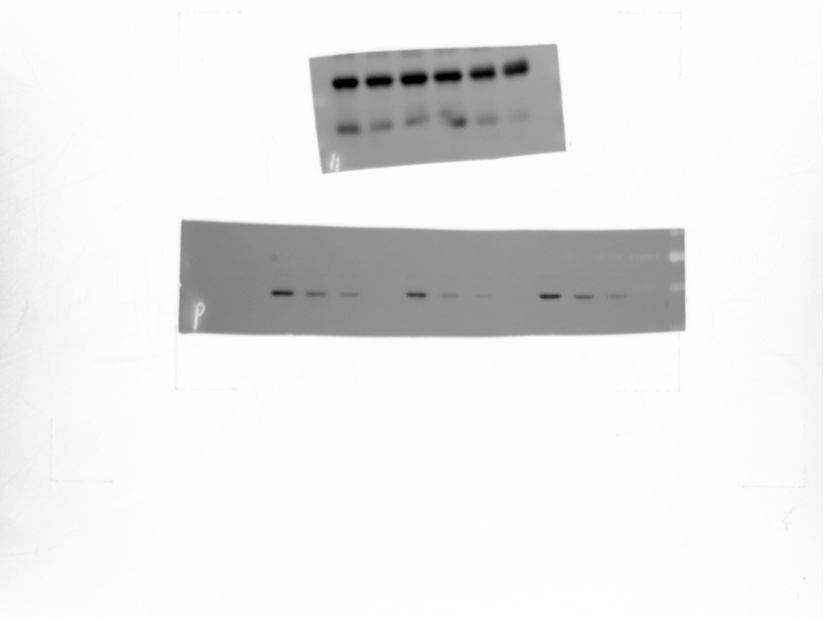

Supplement: Figure 3—source data 1. [file elife-98372-fig3-data1.zip › Figure 3-data1/Figure_3-source_data_1_ Figure_3B_WCL-GFP.jpg]

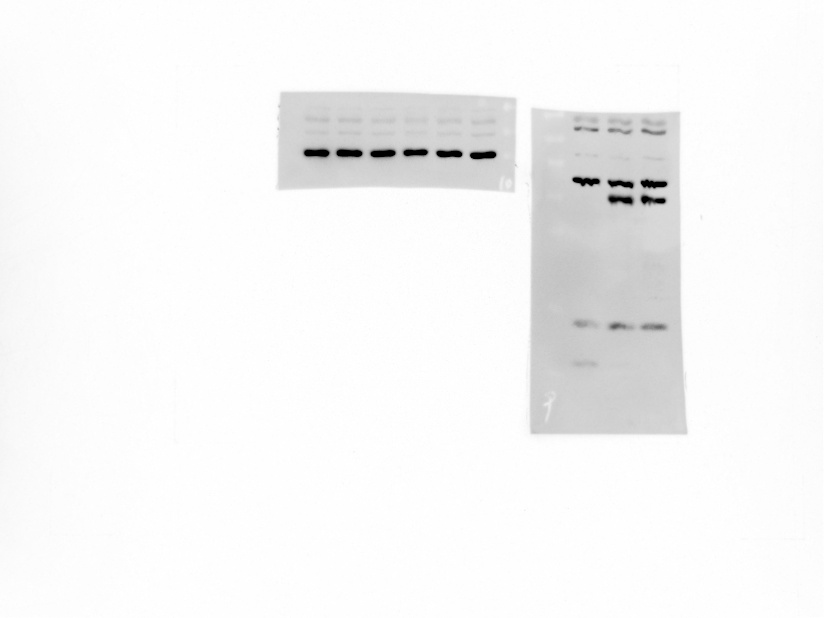

Supplement: Figure 3—source data 1. [file elife-98372-fig3-data1.zip › Figure 3-data1/Figure_3-source_data_1_ Figure_3C_Actin.jpg]

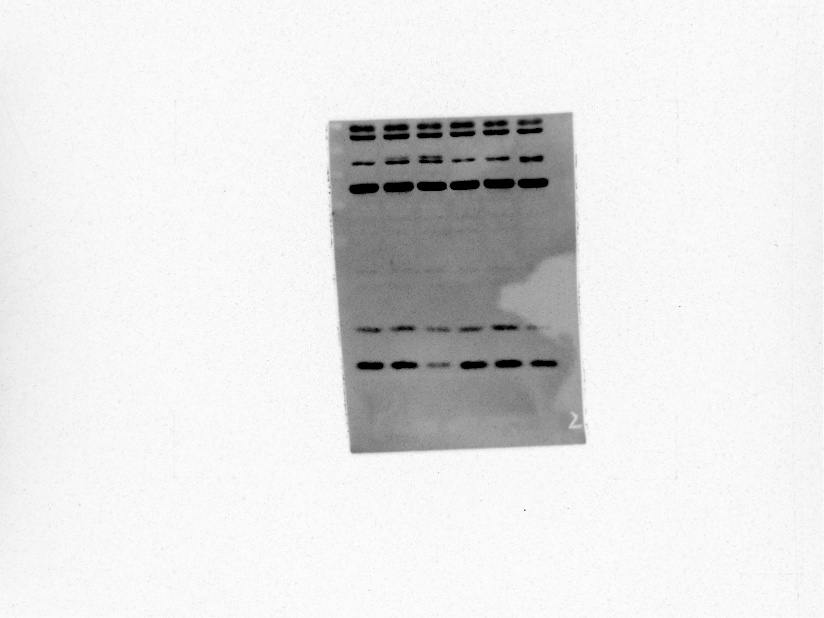

Supplement: Figure 3—source data 1. [file elife-98372-fig3-data1.zip › Figure 3-data1/Figure_3-source_data_1_ Figure_3C_FLAG.jpg]

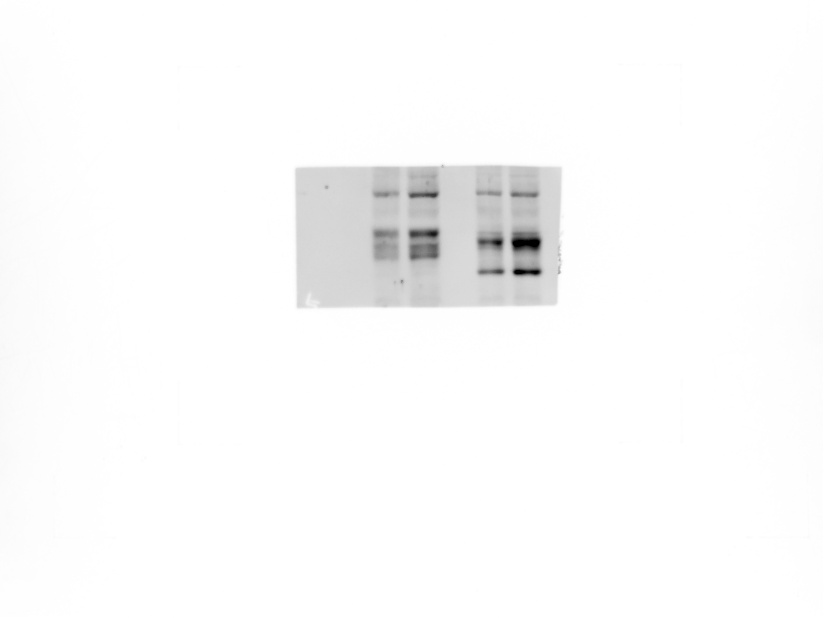

Supplement: Figure 3—source data 1. [file elife-98372-fig3-data1.zip › Figure 3-data1/Figure_3-source_data_1_ Figure_3C_Myc.jpg]

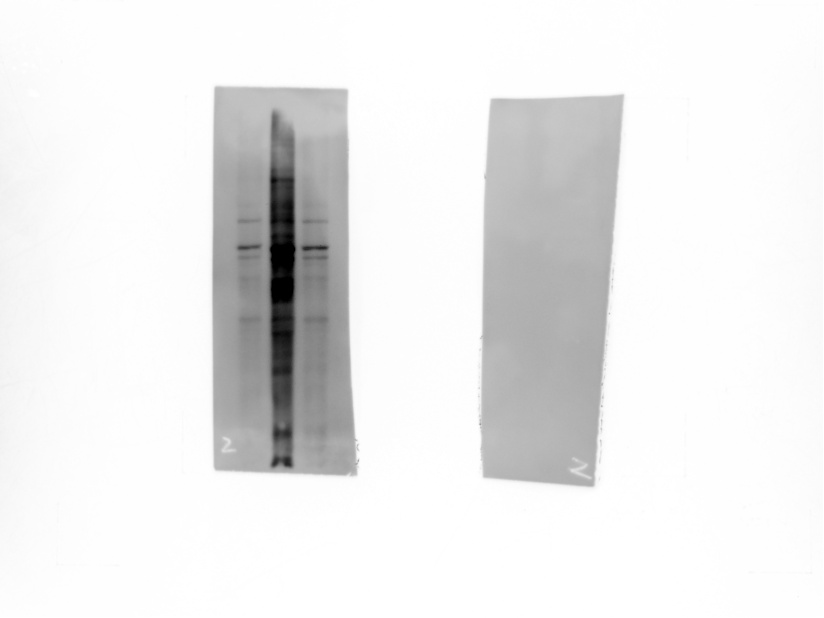

Supplement: Figure 3—source data 1. [file elife-98372-fig3-data1.zip › Figure 3-data1/Figure_3-source_data_1_ Figure_3D_IP-HA.jpg]

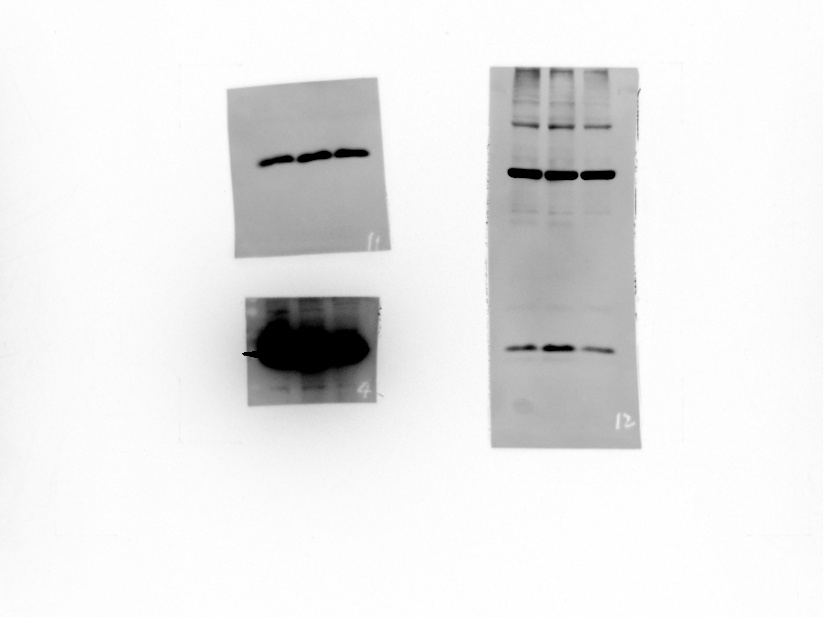

Supplement: Figure 3—source data 1. [file elife-98372-fig3-data1.zip › Figure 3-data1/Figure_3-source_data_1_ Figure_3D_WCL-FLAG.jpg]

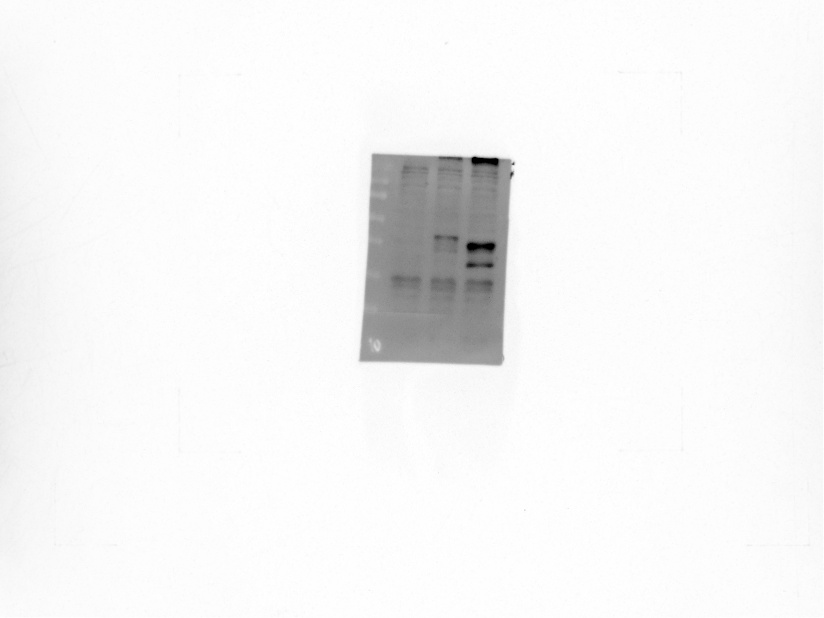

Supplement: Figure 3—source data 1. [file elife-98372-fig3-data1.zip › Figure 3-data1/Figure_3-source_data_1_ Figure_3D_WCL-Myc.jpg]

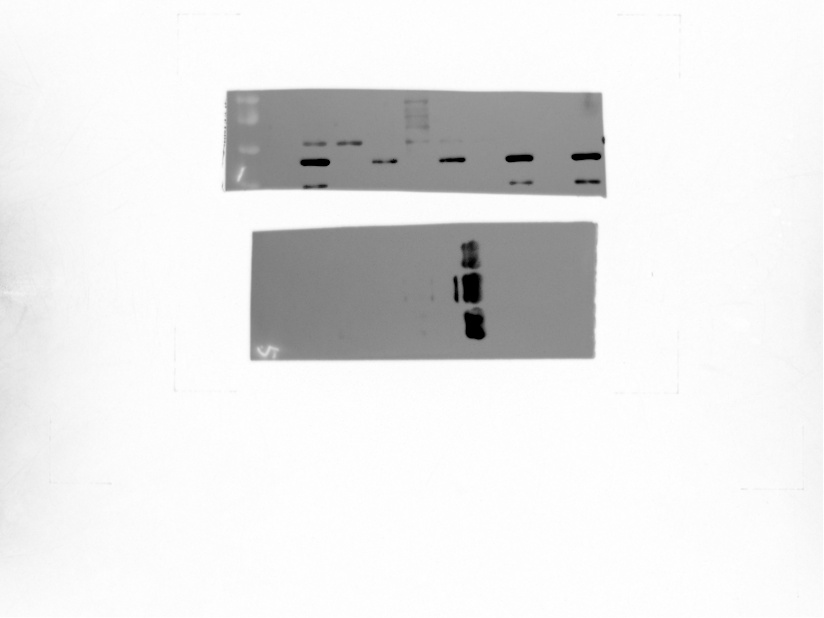

Supplement: Figure 3—source data 1. [file elife-98372-fig3-data1.zip › Figure 3-data1/Figure_3-source_data_1_ Figure_3F_IP-FLAG.jpg]

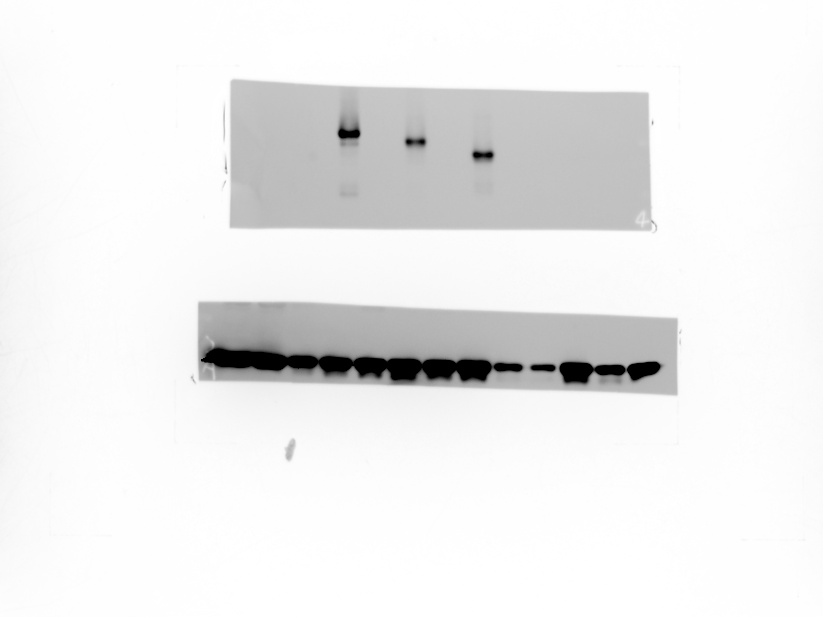

Supplement: Figure 3—source data 1. [file elife-98372-fig3-data1.zip › Figure 3-data1/Figure_3-source_data_1_ Figure_3F_IP-GFP.jpg]

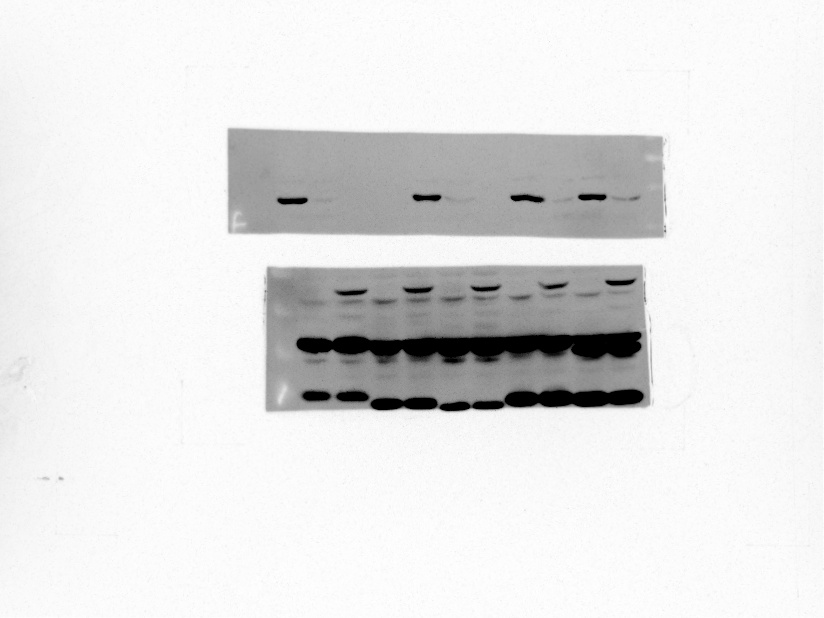

Supplement: Figure 3—source data 1. [file elife-98372-fig3-data1.zip › Figure 3-data1/Figure_3-source_data_1_ Figure_3F_WCL-FLAG.jpg]

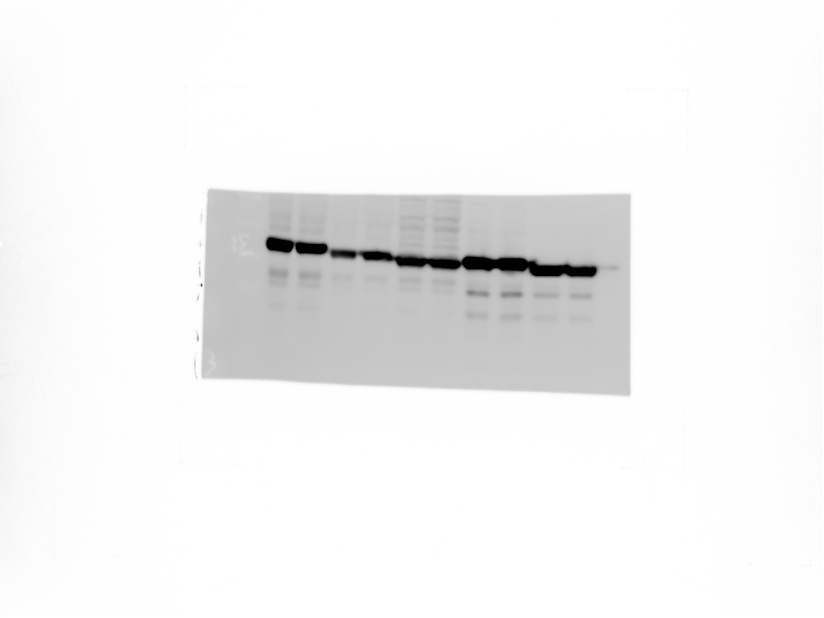

Supplement: Figure 3—source data 1. [file elife-98372-fig3-data1.zip › Figure 3-data1/Figure_3-source_data_1_ Figure_3F_WCL-GFP.jpg]

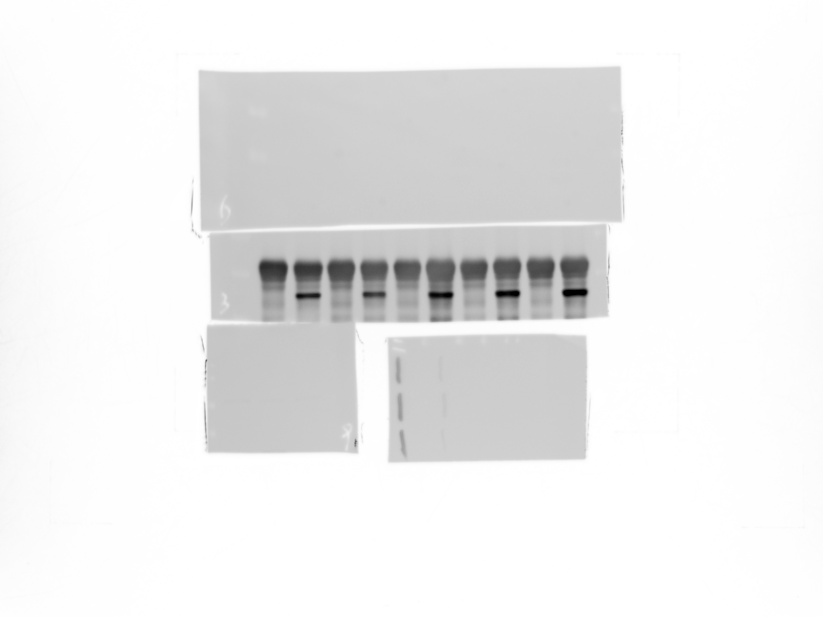

Supplement: Figure 3—source data 1. [file elife-98372-fig3-data1.zip › Figure 3-data1/Figure_3-source_data_1_ Figure_3G_IP-FLAG.jpg]

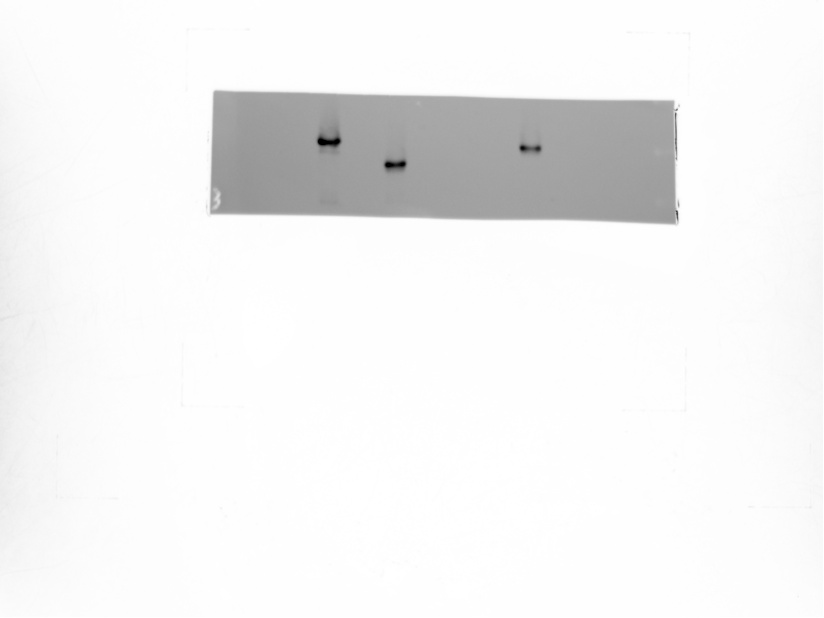

Supplement: Figure 3—source data 1. [file elife-98372-fig3-data1.zip › Figure 3-data1/Figure_3-source_data_1_ Figure_3G_IP-GFP.jpg]

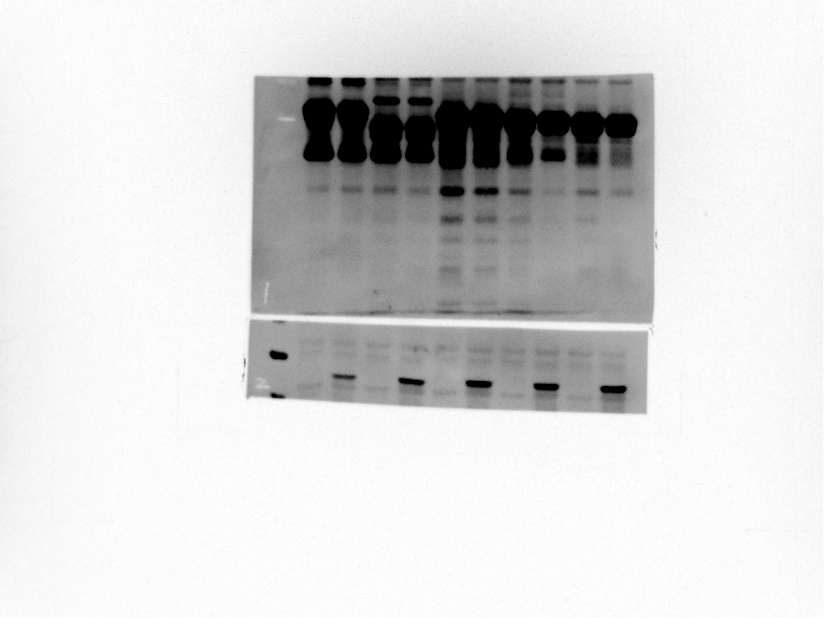

Supplement: Figure 3—source data 1. [file elife-98372-fig3-data1.zip › Figure 3-data1/Figure_3-source_data_1_ Figure_3G_WCL-FLAG.jpg]

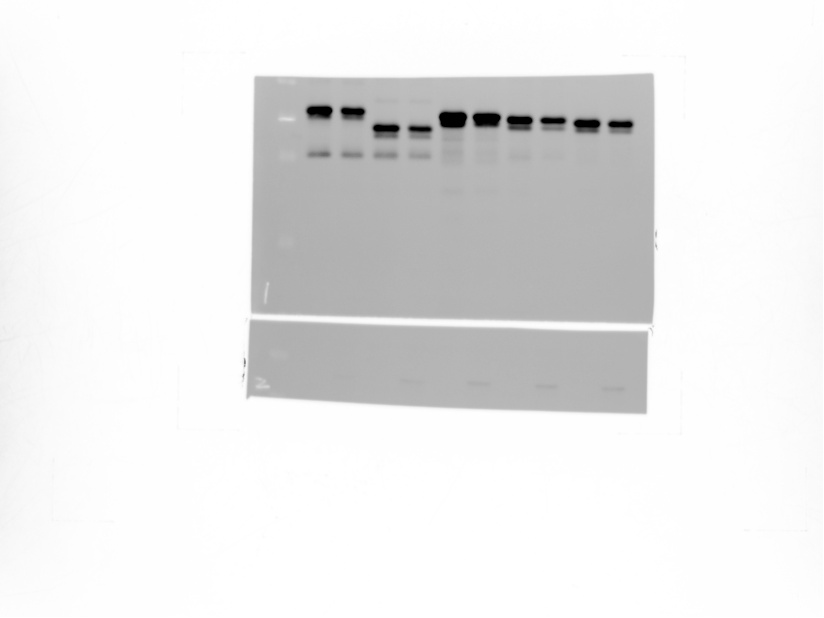

Supplement: Figure 3—source data 1. [file elife-98372-fig3-data1.zip › Figure 3-data1/Figure_3-source_data_1_ Figure_3G_WCL-GFP.jpg]

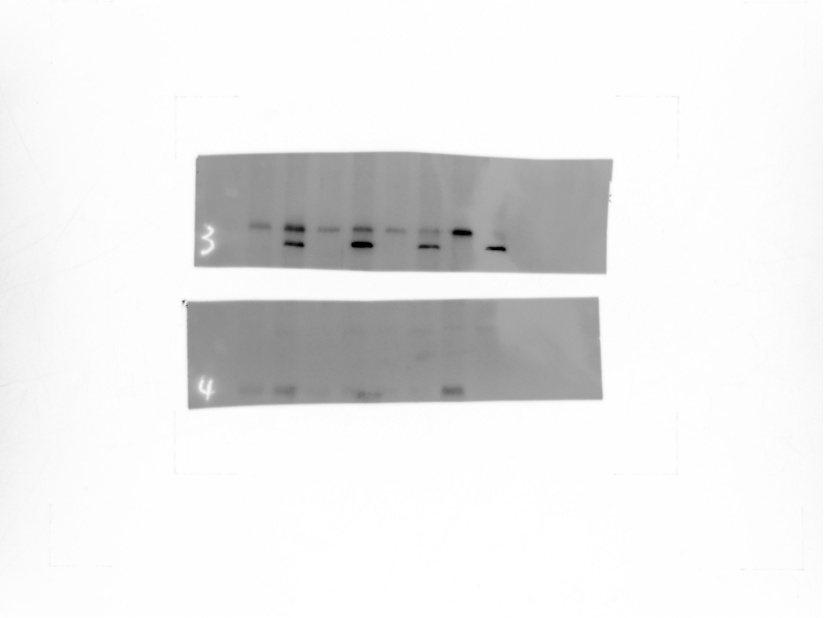

Supplement: Figure 3—source data 1. [file elife-98372-fig3-data1.zip › Figure 3-data1/Figure_3-source_data_1_ Figure_3H_IP-FLAG.jpg]

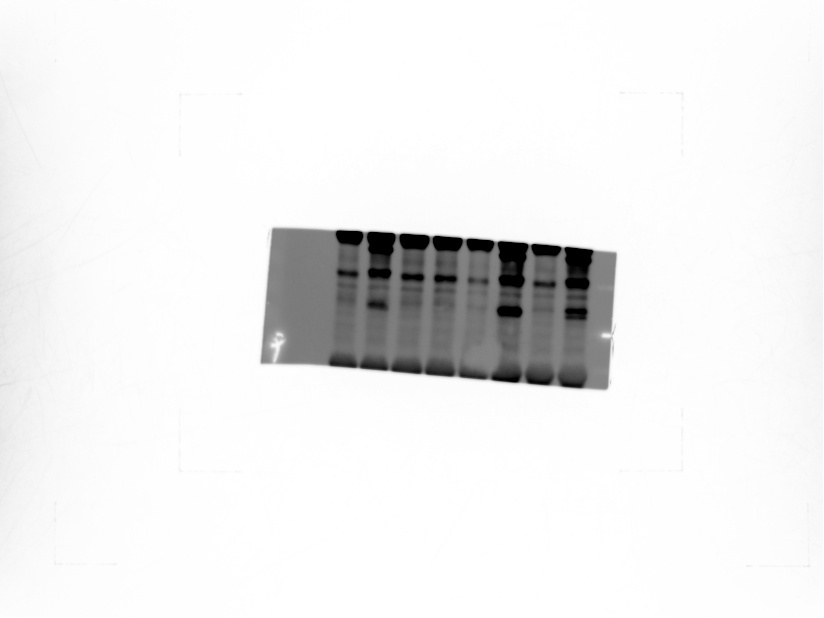

Supplement: Figure 3—source data 1. [file elife-98372-fig3-data1.zip › Figure 3-data1/Figure_3-source_data_1_ Figure_3H_IP-GFP.jpg]

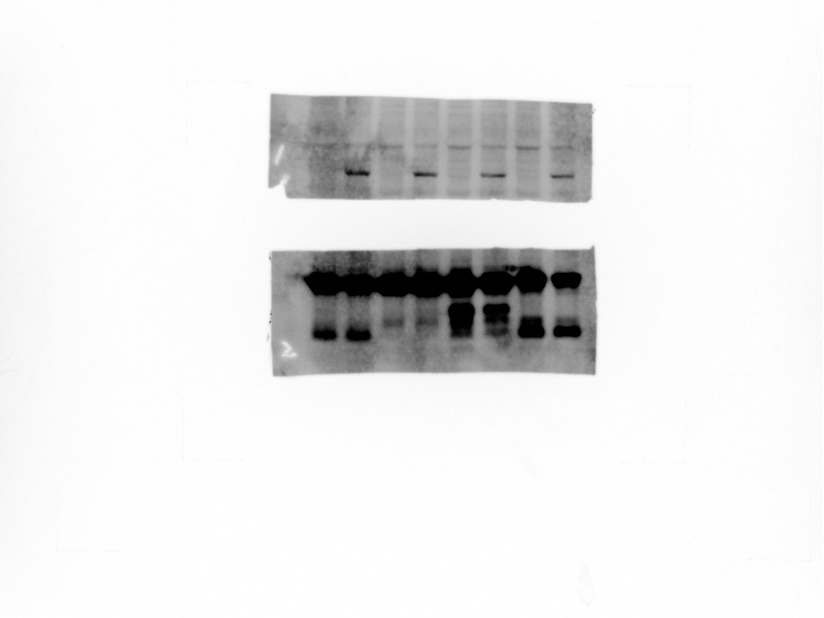

Supplement: Figure 3—source data 1. [file elife-98372-fig3-data1.zip › Figure 3-data1/Figure_3-source_data_1_ Figure_3H_WCL-FLAG.jpg]

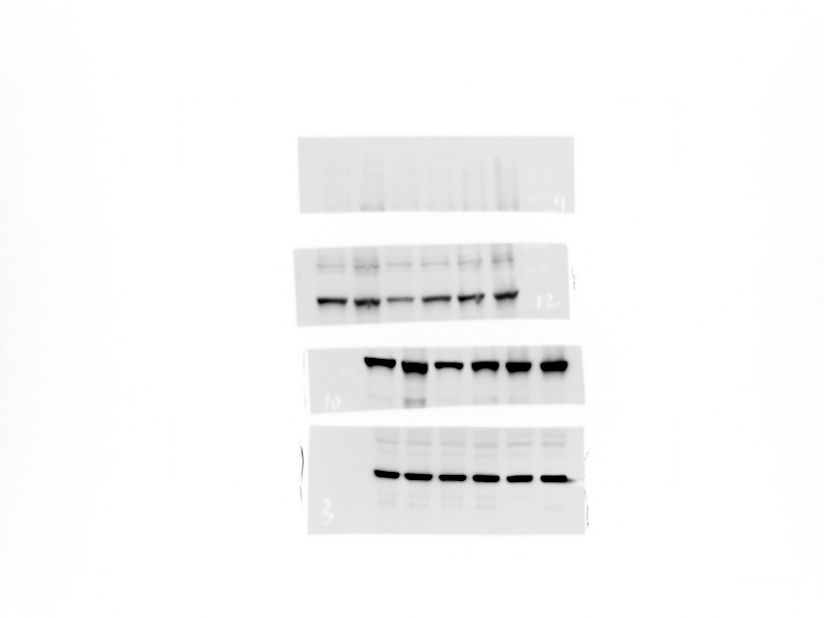

Supplement: Figure 3—source data 1. [file elife-98372-fig3-data1.zip › Figure 3-data1/Figure_3-source_data_1_ Figure_3I_Actin.jpg]

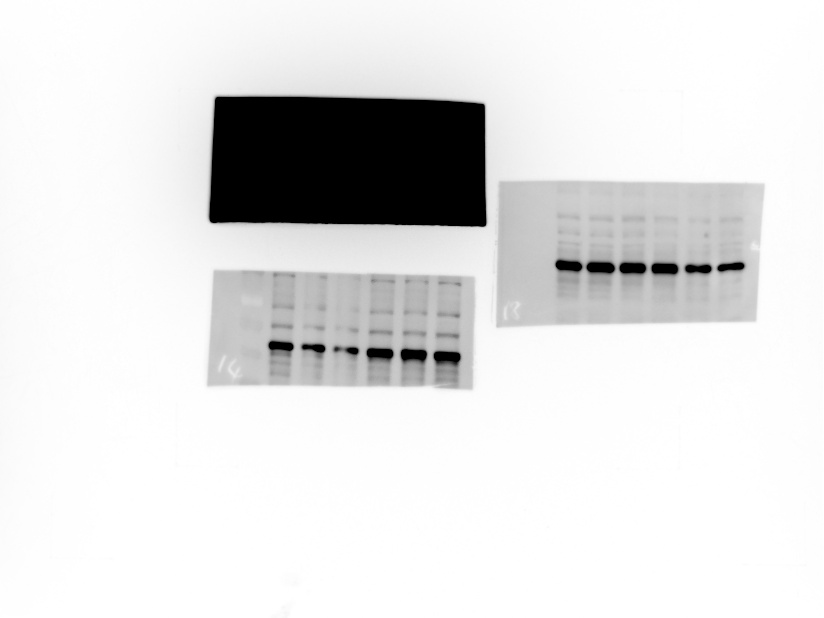

Supplement: Figure 3—source data 1. [file elife-98372-fig3-data1.zip › Figure 3-data1/Figure_3-source_data_1_ Figure_3I_FLAG.jpg]

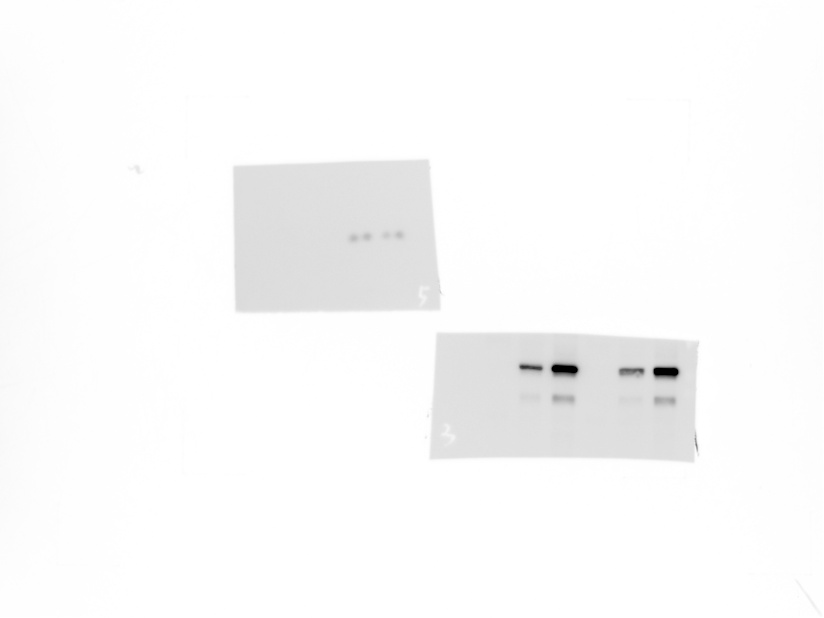

Supplement: Figure 3—source data 1. [file elife-98372-fig3-data1.zip › Figure 3-data1/Figure_3-source_data_1_ Figure_3I_Myc.jpg]

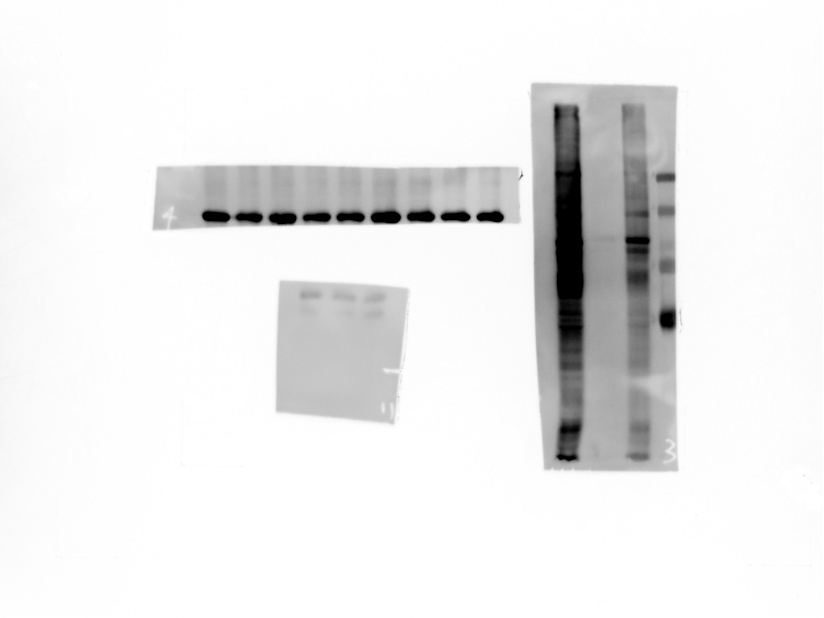

Supplement: Figure 3—source data 1. [file elife-98372-fig3-data1.zip › Figure 3-data1/Figure_3-source_data_1_ Figure_3J_IP-HA.jpg]

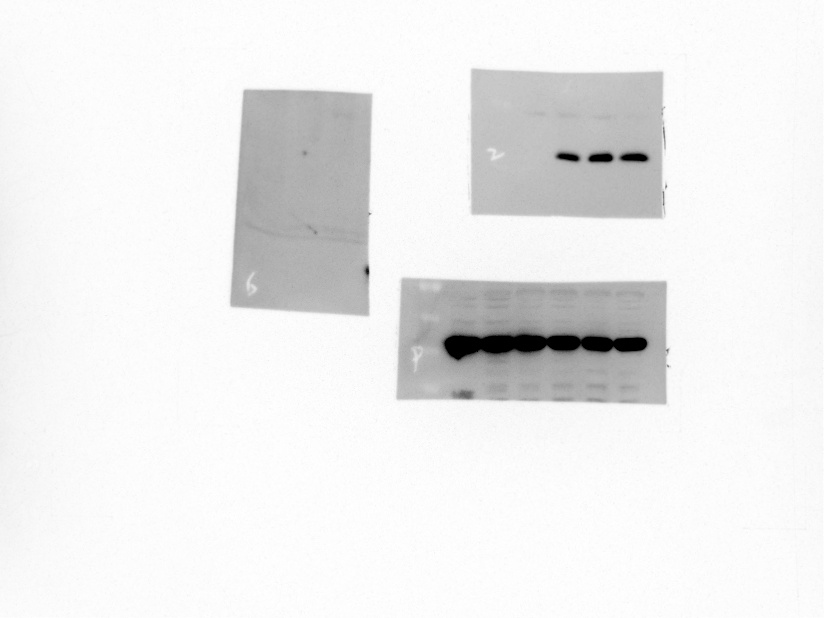

Supplement: Figure 3—source data 1. [file elife-98372-fig3-data1.zip › Figure 3-data1/Figure_3-source_data_1_ Figure_3J_WCL-FLAG.jpg]

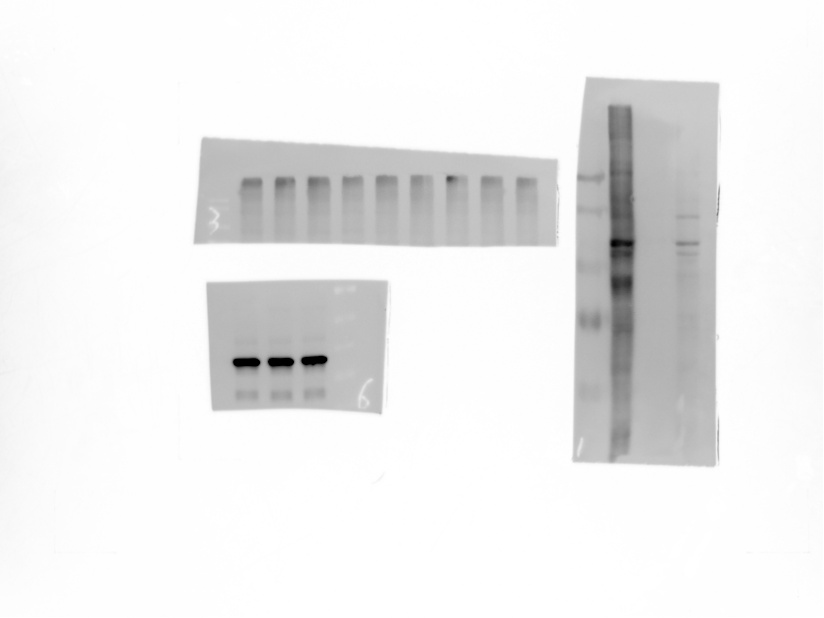

Supplement: Figure 3—source data 1. [file elife-98372-fig3-data1.zip › Figure 3-data1/Figure_3-source_data_1_ Figure_3J_WCL-Myc.jpg]

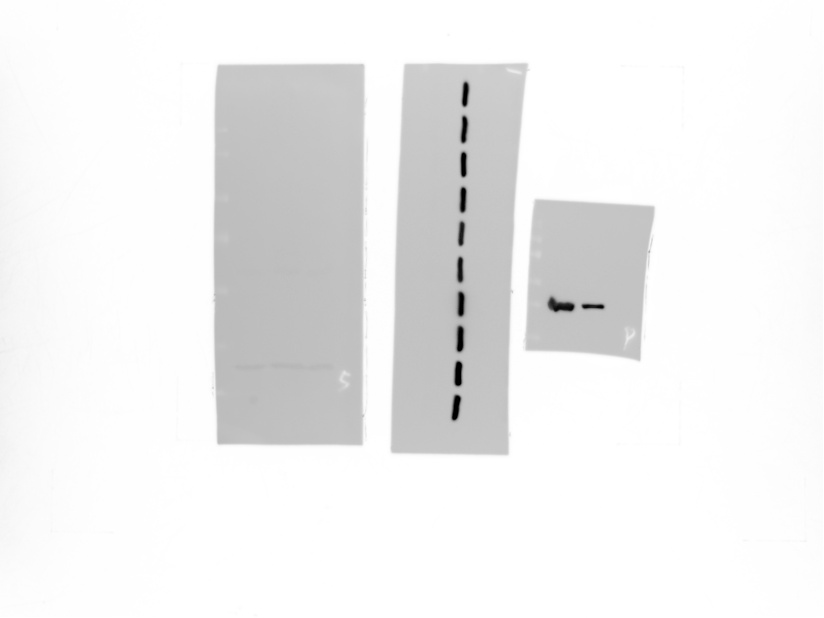

Supplement: Figure 3—source data 1. [file elife-98372-fig3-data1.zip › Figure 3-data1/Figure_3-source_data_1_ Figure_3K_Actin.jpg]

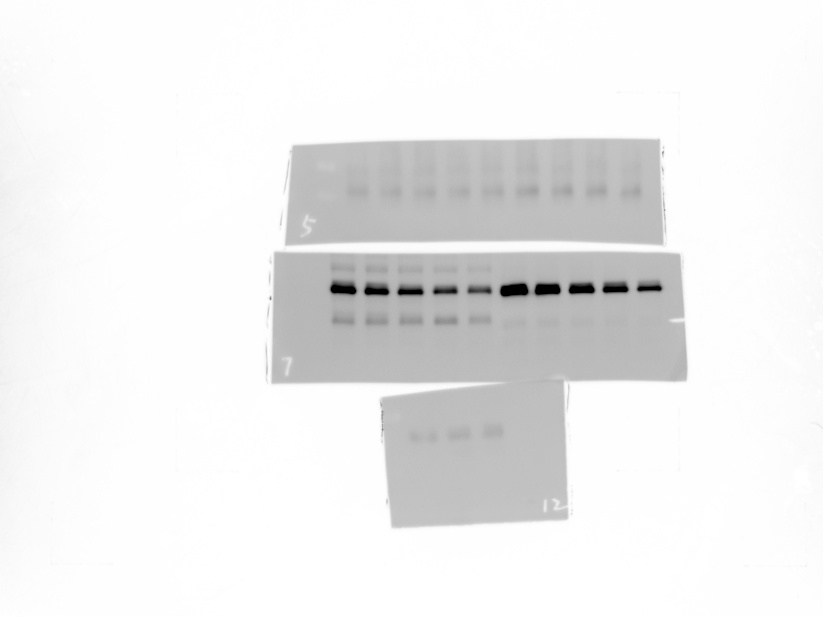

Supplement: Figure 3—source data 1. [file elife-98372-fig3-data1.zip › Figure 3-data1/Figure_3-source_data_1_ Figure_3K_GFP.jpg]

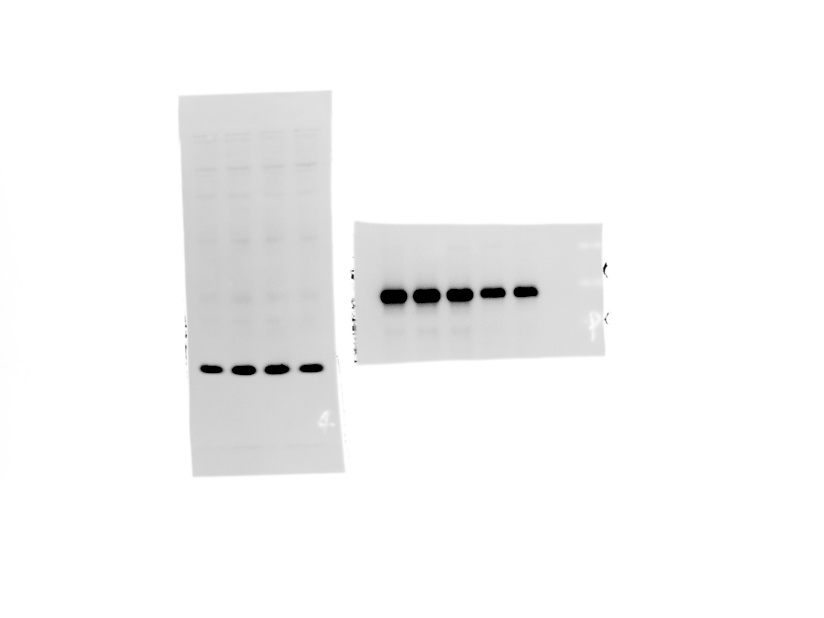

Supplement: Figure 3—source data 1. [file elife-98372-fig3-data1.zip › Figure 3-data1/Figure_3-source_data_1_ Figure_3M_IP-FLAG.jpg]

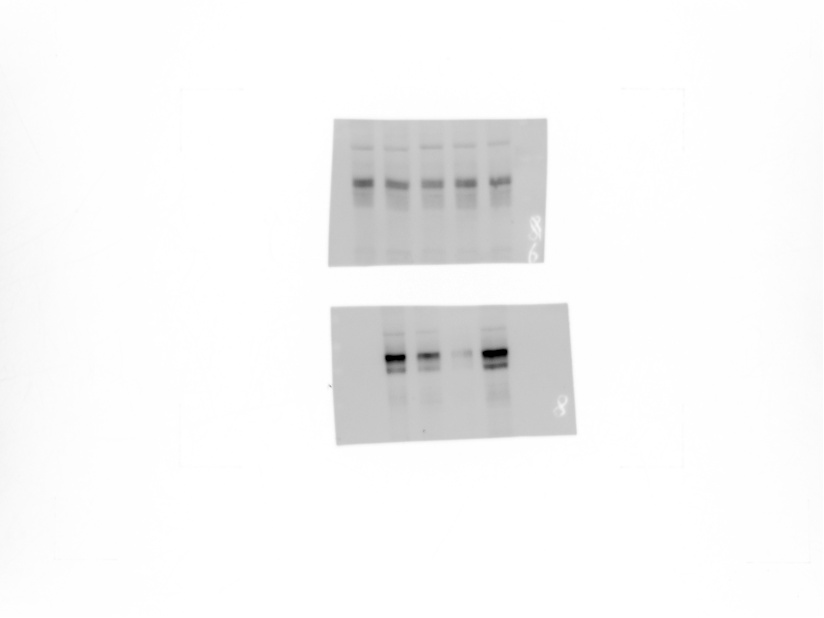

Supplement: Figure 3—source data 1. [file elife-98372-fig3-data1.zip › Figure 3-data1/Figure_3-source_data_1_ Figure_3M_IP-Myc.jpg]

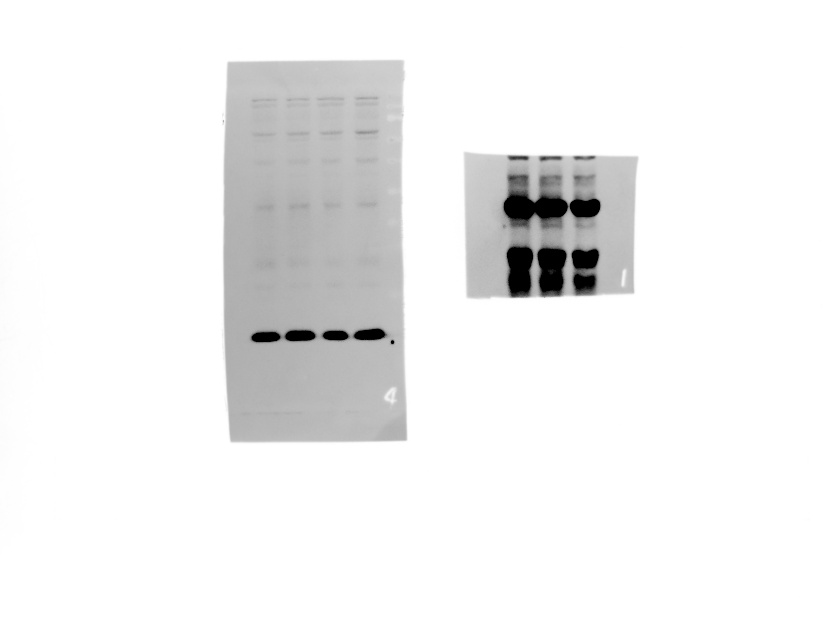

Supplement: Figure 3—source data 1. [file elife-98372-fig3-data1.zip › Figure 3-data1/Figure_3-source_data_1_ Figure_3M_WCL-FLAG.jpg]

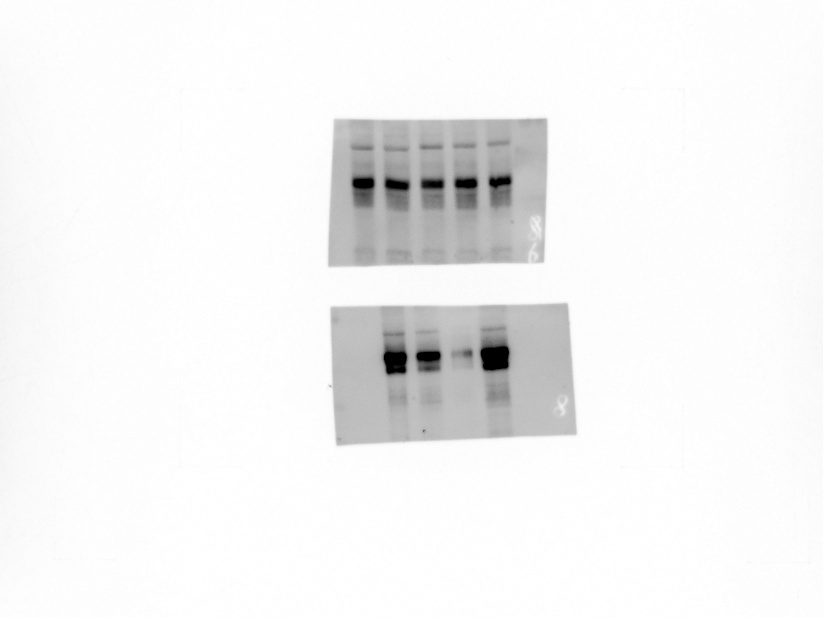

Supplement: Figure 3—source data 1. [file elife-98372-fig3-data1.zip › Figure 3-data1/Figure_3-source_data_1_ Figure_3M_WCL-Myc.jpg]

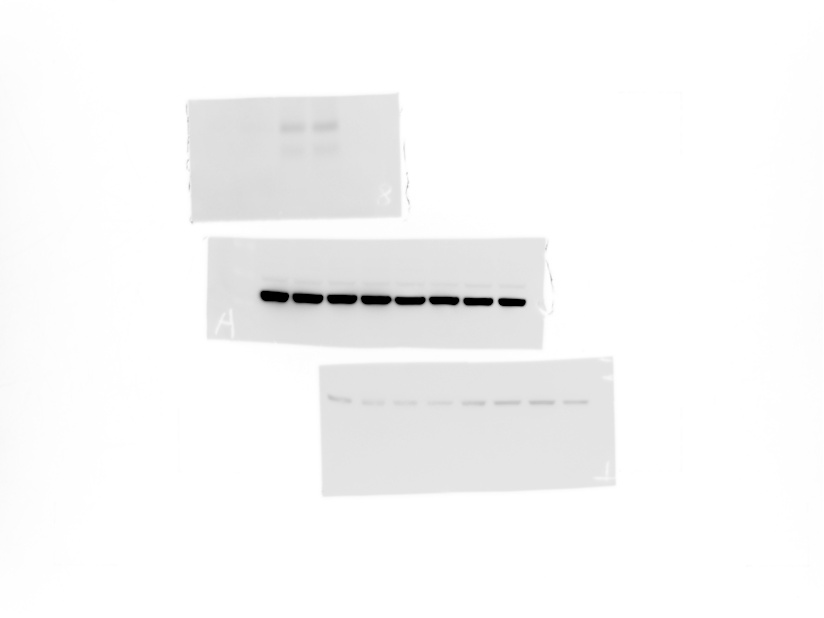

Supplement: Figure 3—source data 1. [file elife-98372-fig3-data1.zip › Figure 3-data1/Figure_3-source_data_1_ Figure_3N_Actin(C4-2B).jpg]

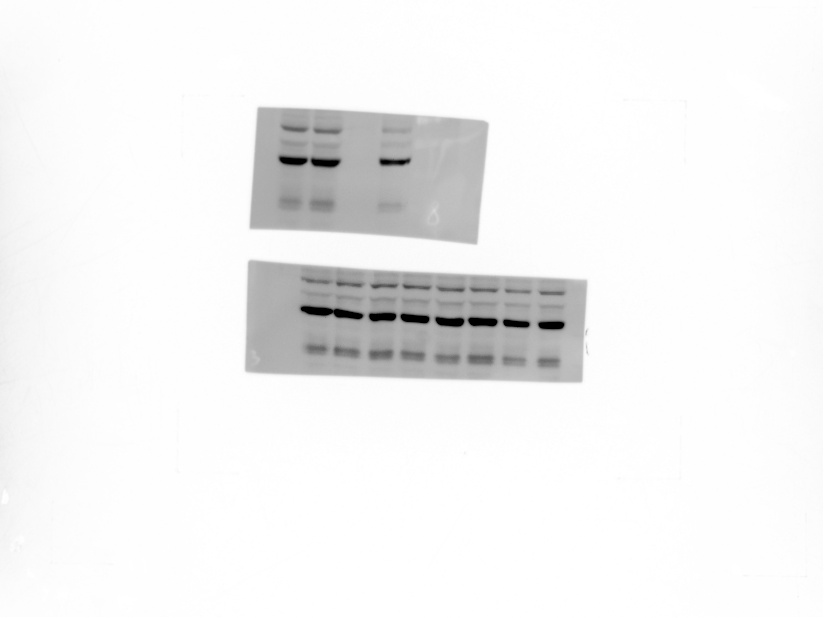

Supplement: Figure 3—source data 1. [file elife-98372-fig3-data1.zip › Figure 3-data1/Figure_3-source_data_1_ Figure_3N_Actin(Huh-7).jpg]

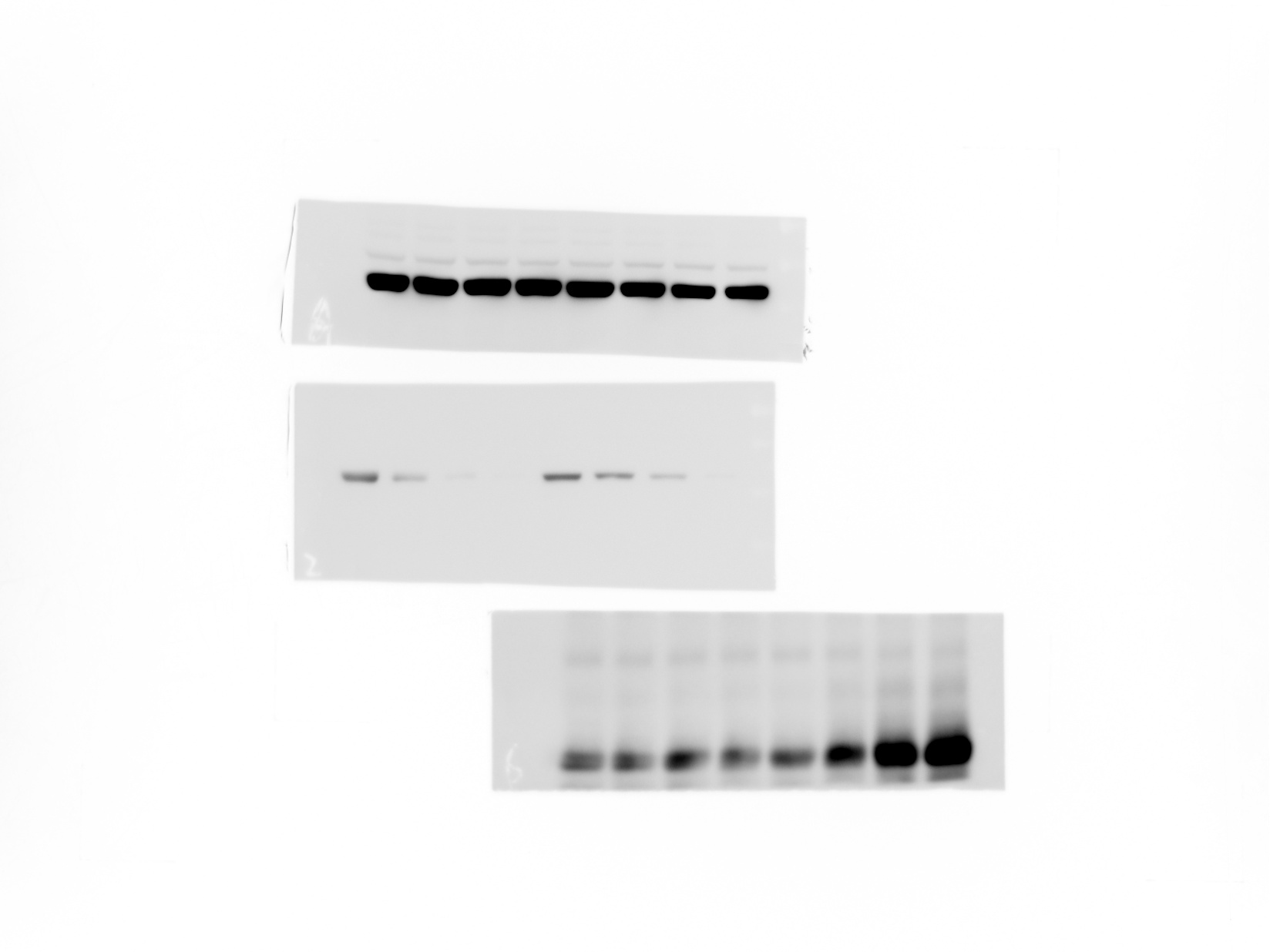

Supplement: Figure 3—source data 1. [file elife-98372-fig3-data1.zip › Figure 3-data1/Figure_3-source_data_1_ Figure_3N_NOXA(C4-2B).jpg]

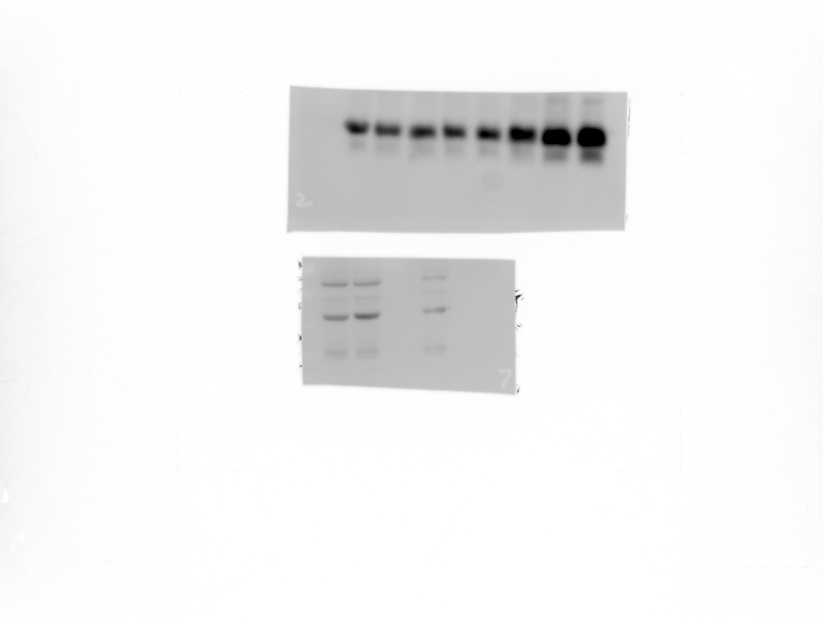

Supplement: Figure 3—source data 1. [file elife-98372-fig3-data1.zip › Figure 3-data1/Figure_3-source_data_1_ Figure_3N_NOXA(Huh-7).jpg]

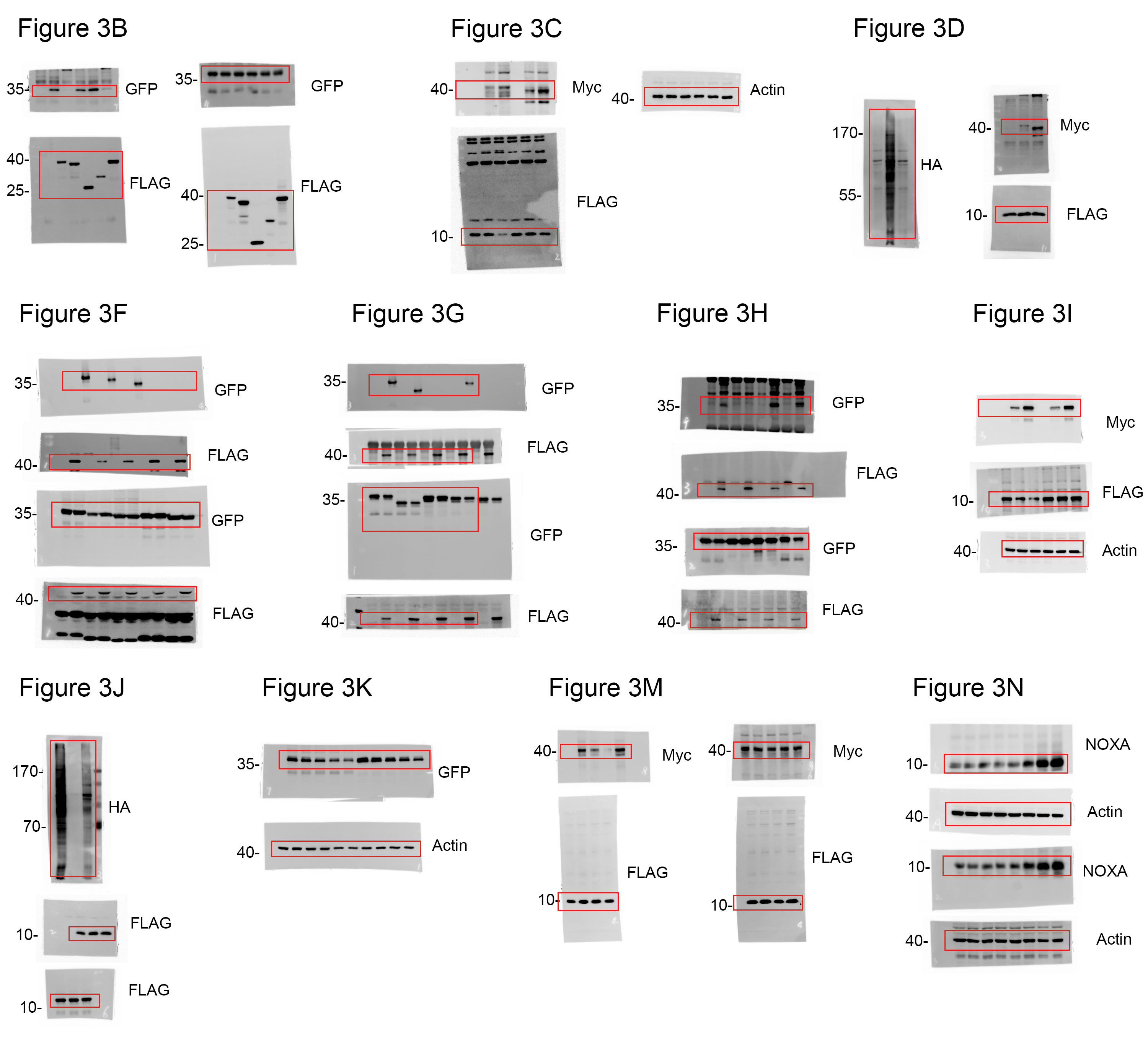

Supplement: Figure 3—source data 2. [file elife-98372-fig3-data2.zip › Figure 3-data2/Figure_3_data_2.jpg]

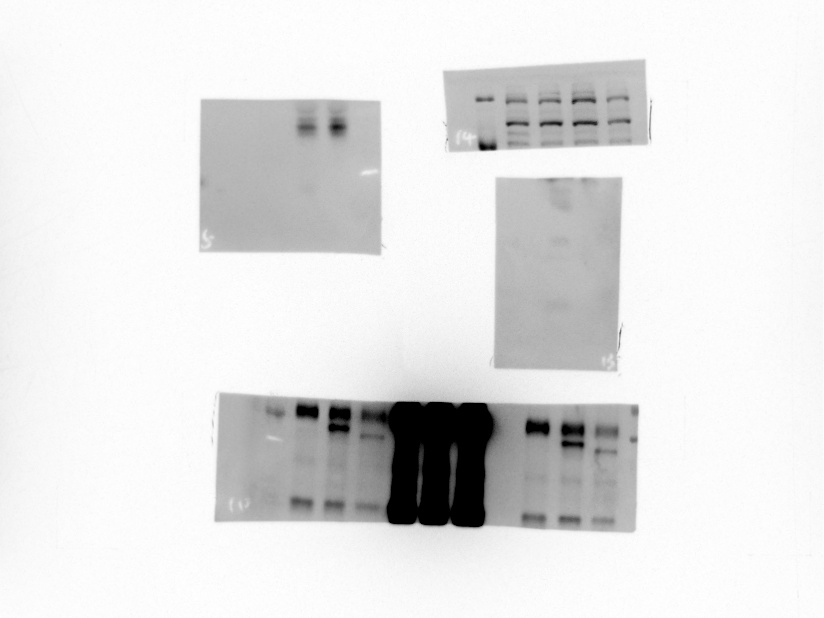

Supplement: Figure 3—figure supplement 1—source data 1. [file elife-98372-fig3-figsupp1-data1.zip › Figure 3-supplementary figure 1-data1/Figure_3-figure supplement_1_ source_data_1_ Figure_A_FLAG(IP).jpg]

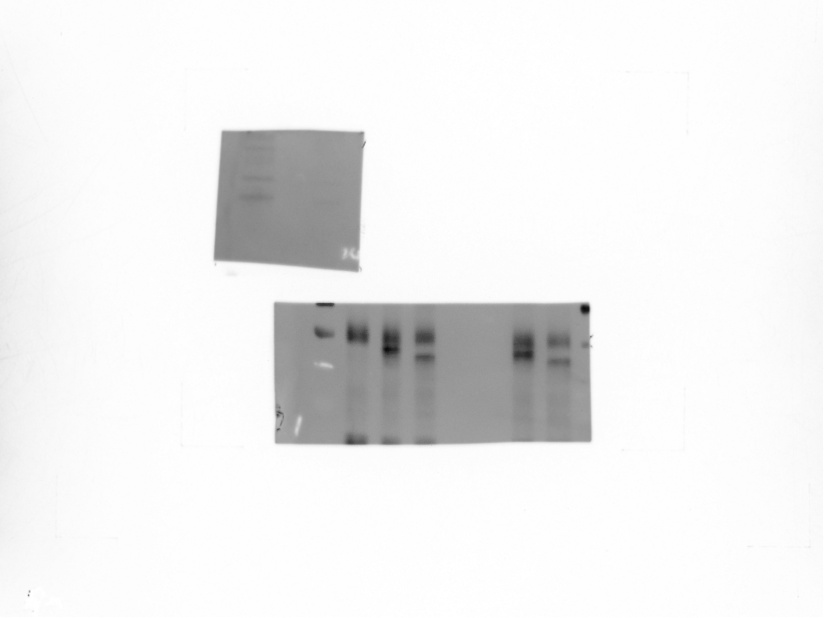

Supplement: Figure 3—figure supplement 1—source data 1. [file elife-98372-fig3-figsupp1-data1.zip › Figure 3-supplementary figure 1-data1/Figure_3-figure supplement_1_ source_data_1_ Figure_A_FLAG(WCL).jpg]

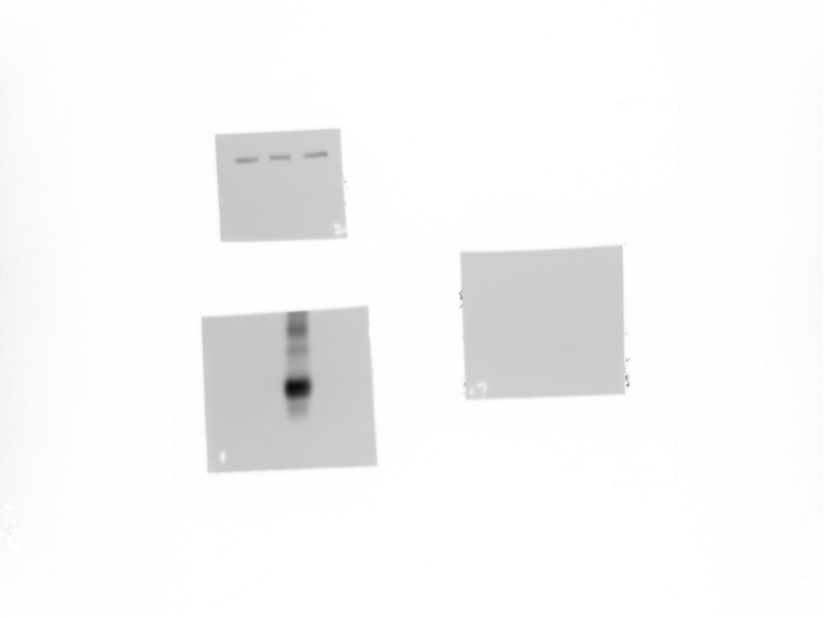

Supplement: Figure 3—figure supplement 1—source data 1. [file elife-98372-fig3-figsupp1-data1.zip › Figure 3-supplementary figure 1-data1/Figure_3-figure supplement_1_ source_data_1_ Figure_A_NOXA(IP).jpg]

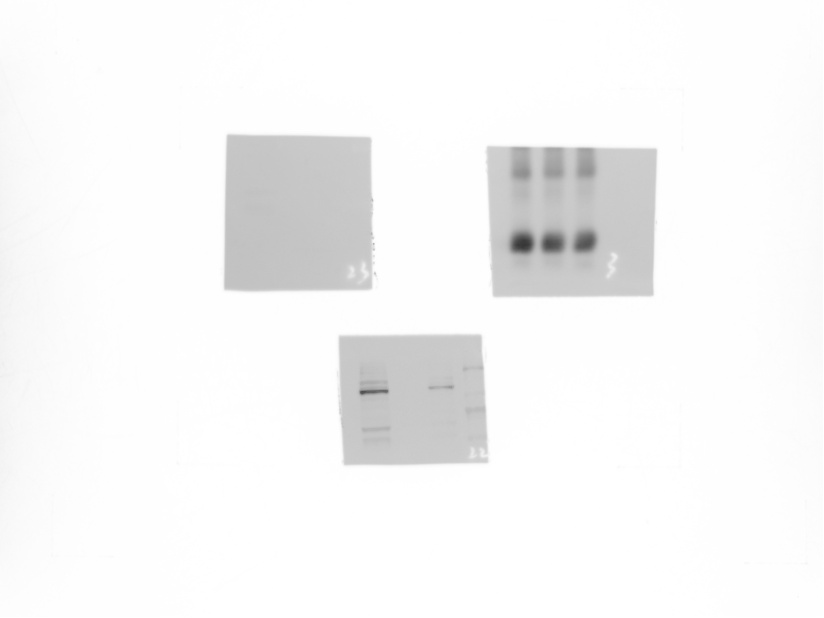

Supplement: Figure 3—figure supplement 1—source data 1. [file elife-98372-fig3-figsupp1-data1.zip › Figure 3-supplementary figure 1-data1/Figure_3-figure supplement_1_ source_data_1_ Figure_A_NOXA(WCL).jpg]

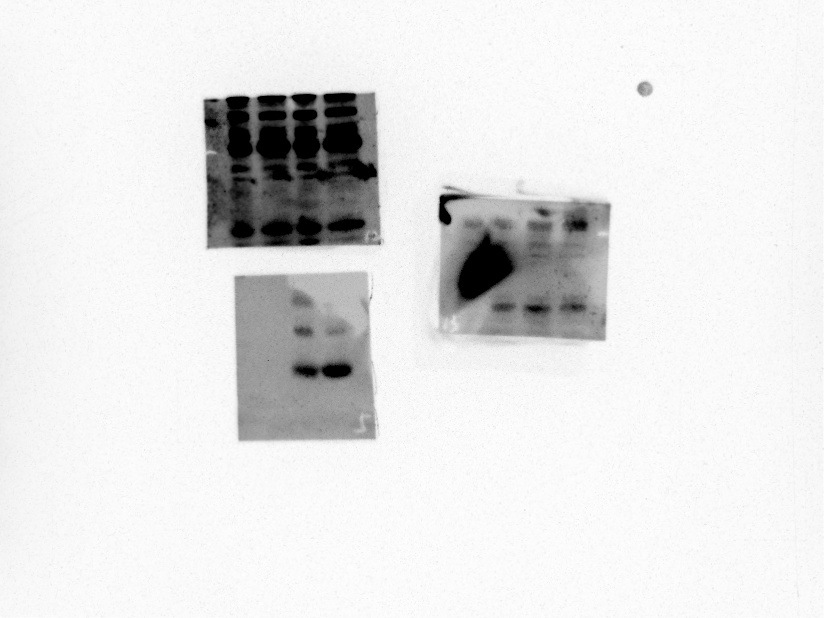

Supplement: Figure 3—figure supplement 1—source data 1. [file elife-98372-fig3-figsupp1-data1.zip › Figure 3-supplementary figure 1-data1/Figure_3-figure supplement_1_ source_data_1_ Figure_B_FLAG(IP).jpg]

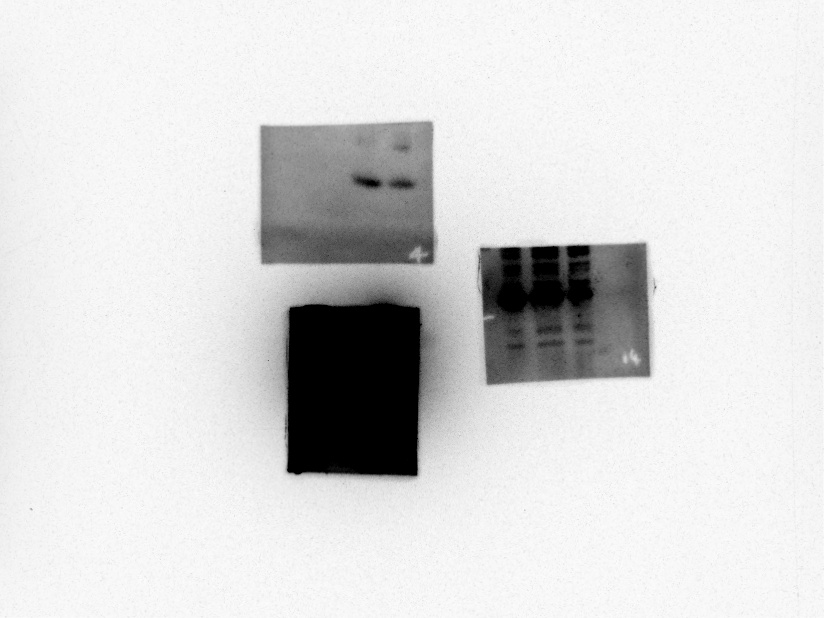

Supplement: Figure 3—figure supplement 1—source data 1. [file elife-98372-fig3-figsupp1-data1.zip › Figure 3-supplementary figure 1-data1/Figure_3-figure supplement_1_ source_data_1_ Figure_B_FLAG(WCL).jpg]

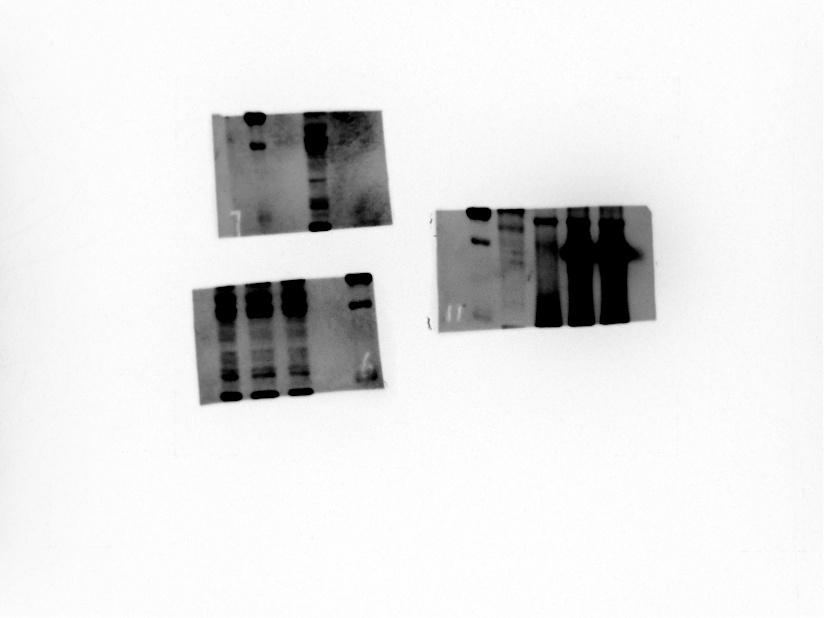

Supplement: Figure 3—figure supplement 1—source data 1. [file elife-98372-fig3-figsupp1-data1.zip › Figure 3-supplementary figure 1-data1/Figure_3-figure supplement_1_ source_data_1_ Figure_B_WSB2(IP).jpg]

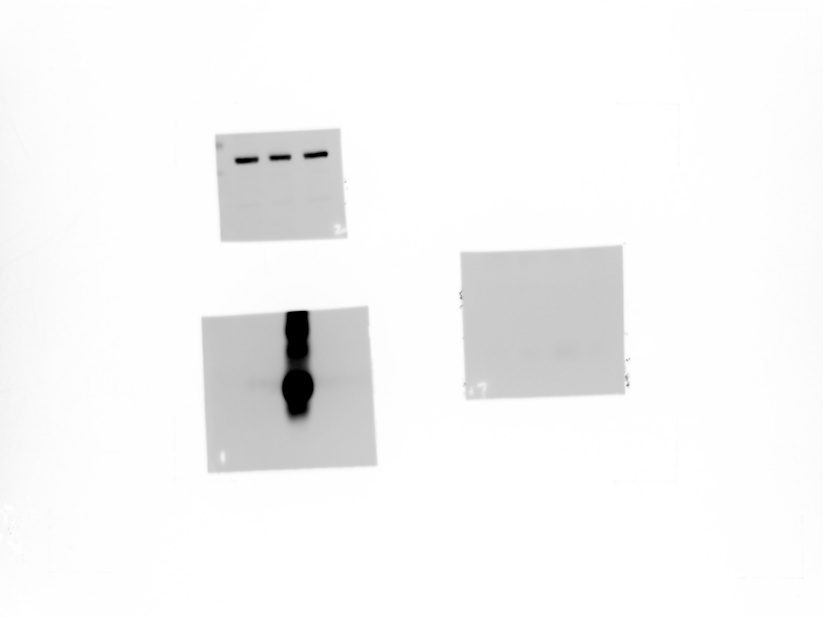

Supplement: Figure 3—figure supplement 1—source data 1. [file elife-98372-fig3-figsupp1-data1.zip › Figure 3-supplementary figure 1-data1/Figure_3-figure supplement_1_ source_data_1_ Figure_B_WSB2(WCL).jpg]

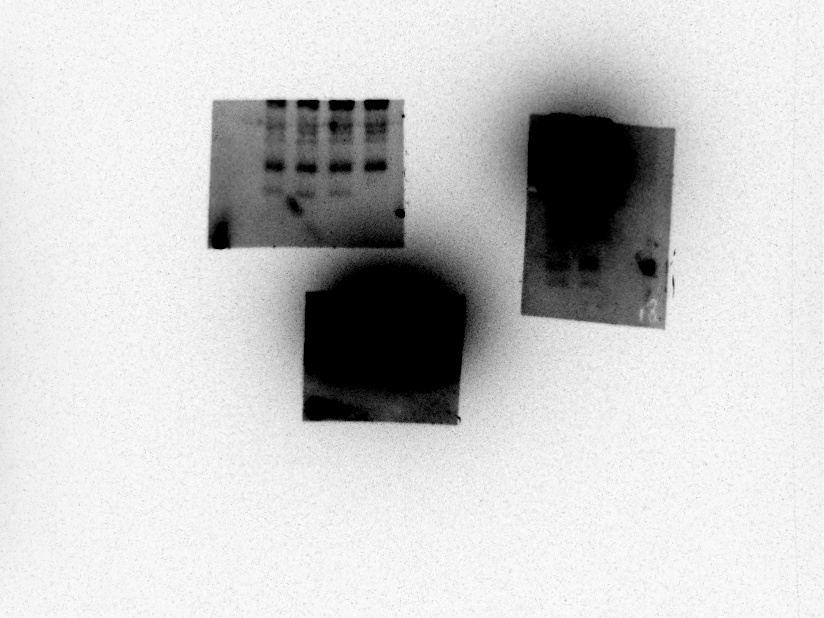

Supplement: Figure 3—figure supplement 1—source data 1. [file elife-98372-fig3-figsupp1-data1.zip › Figure 3-supplementary figure 1-data1/Figure_3-figure supplement_1_ source_data_1_ Figure_C_FLAG.jpg]

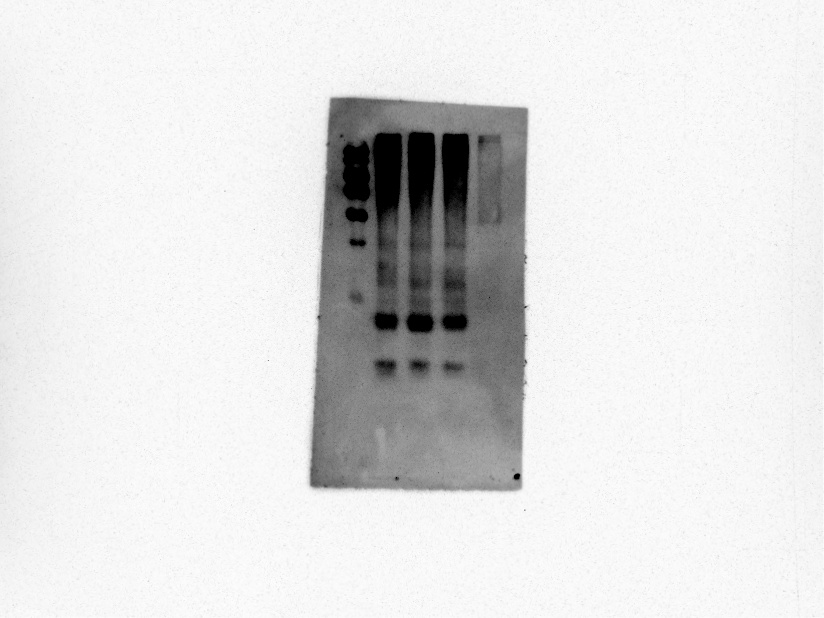

Supplement: Figure 3—figure supplement 1—source data 1. [file elife-98372-fig3-figsupp1-data1.zip › Figure 3-supplementary figure 1-data1/Figure_3-figure supplement_1_ source_data_1_ Figure_C_HA.jpg]

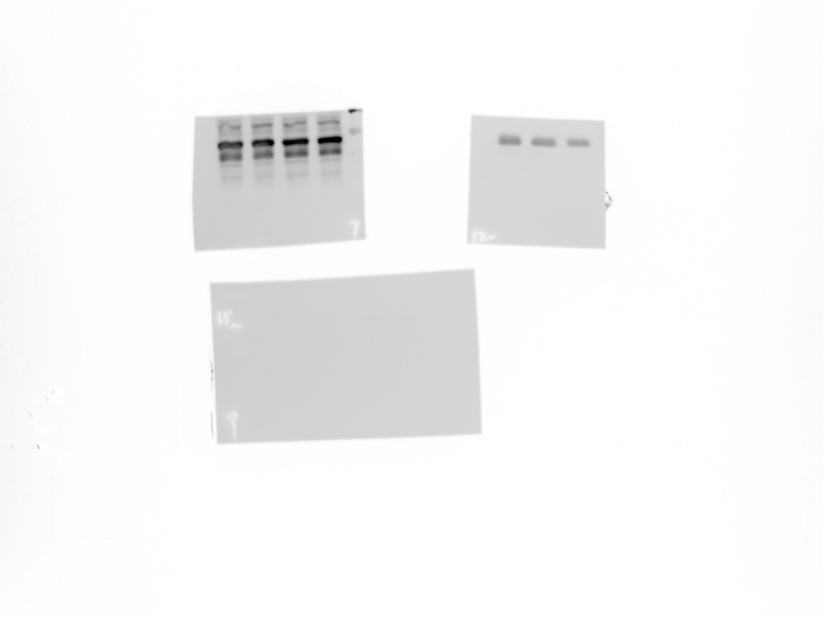

Supplement: Figure 3—figure supplement 1—source data 1. [file elife-98372-fig3-figsupp1-data1.zip › Figure 3-supplementary figure 1-data1/Figure_3-figure supplement_1_ source_data_1_ Figure_C_Myc.jpg]

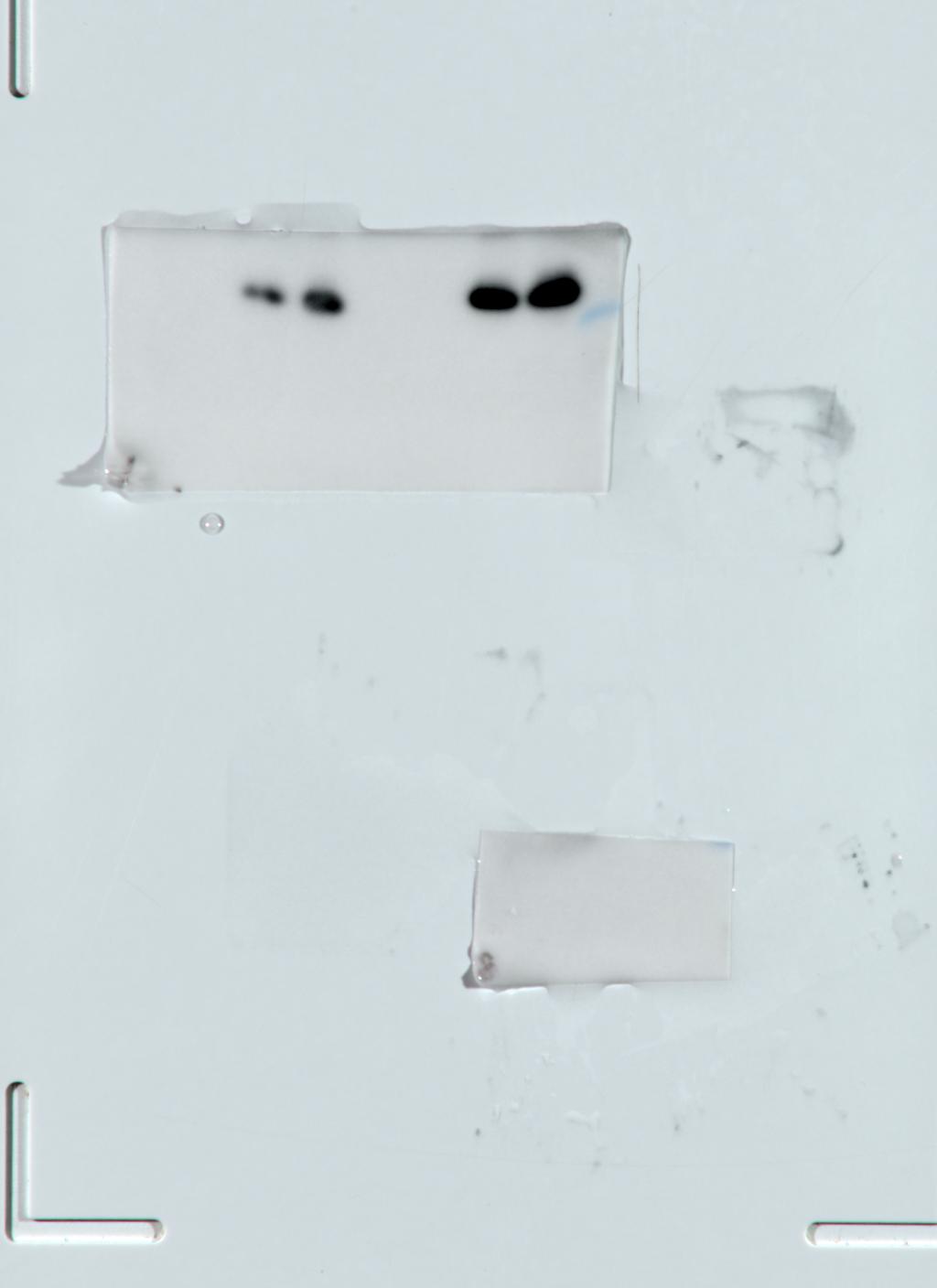

Supplement: Figure 3—figure supplement 1—source data 1. [file elife-98372-fig3-figsupp1-data1.zip › Figure 3-supplementary figure 1-data1/Figure_3-figure supplement_1_ source_data_1_ Figure_D_NOXA.jpg]

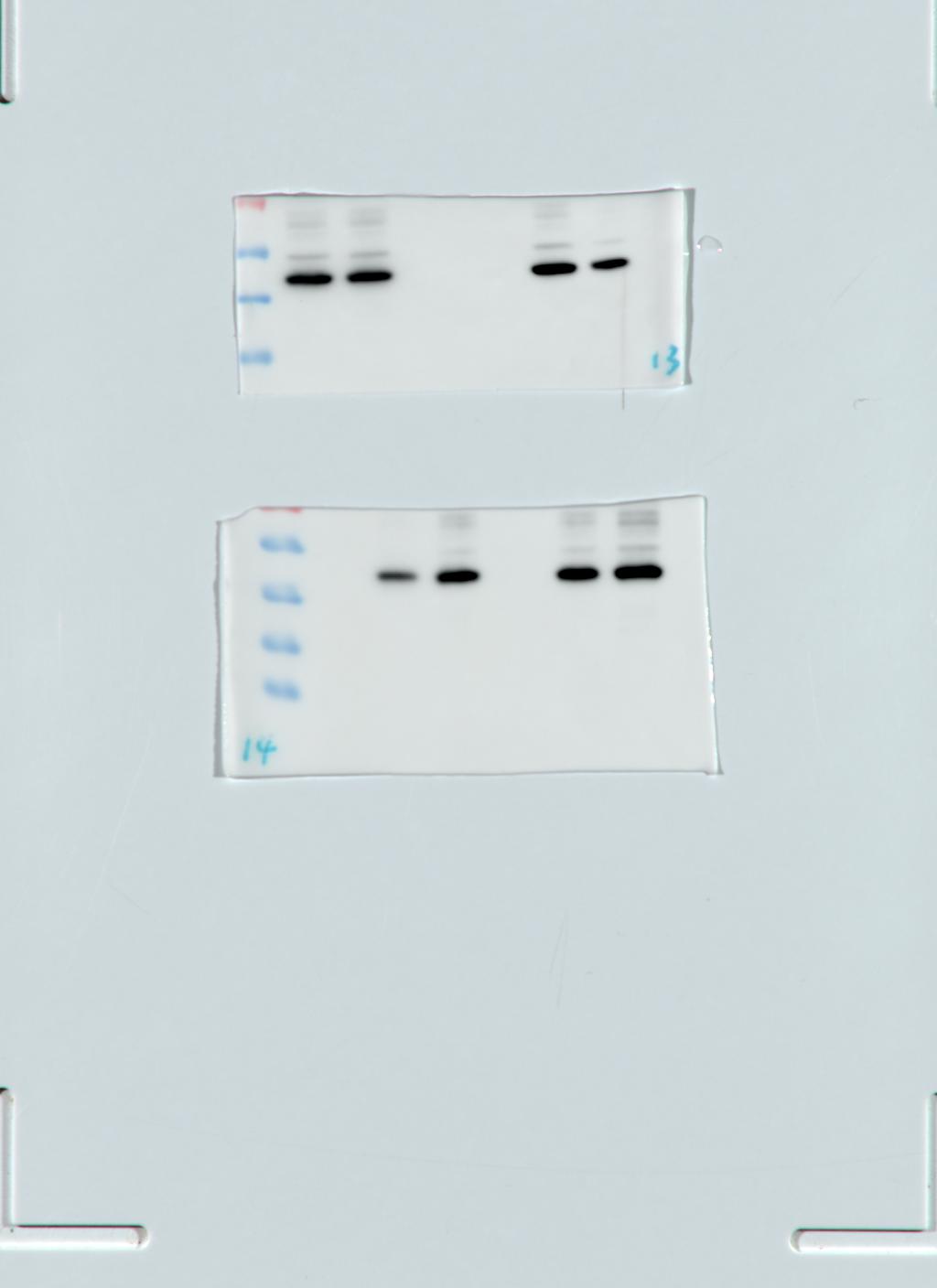

Supplement: Figure 3—figure supplement 1—source data 1. [file elife-98372-fig3-figsupp1-data1.zip › Figure 3-supplementary figure 1-data1/Figure_3-figure supplement_1_ source_data_1_ Figure_D_WSB2.jpg]

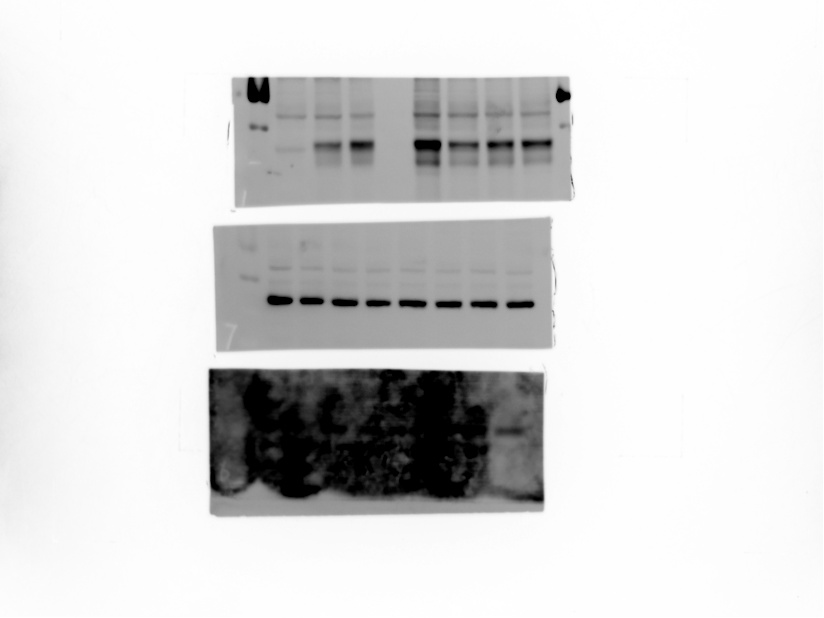

Supplement: Figure 3—figure supplement 1—source data 1. [file elife-98372-fig3-figsupp1-data1.zip › Figure 3-supplementary figure 1-data1/Figure_3-figure supplement_1_ source_data_1_ Figure_E_Actin.jpg]

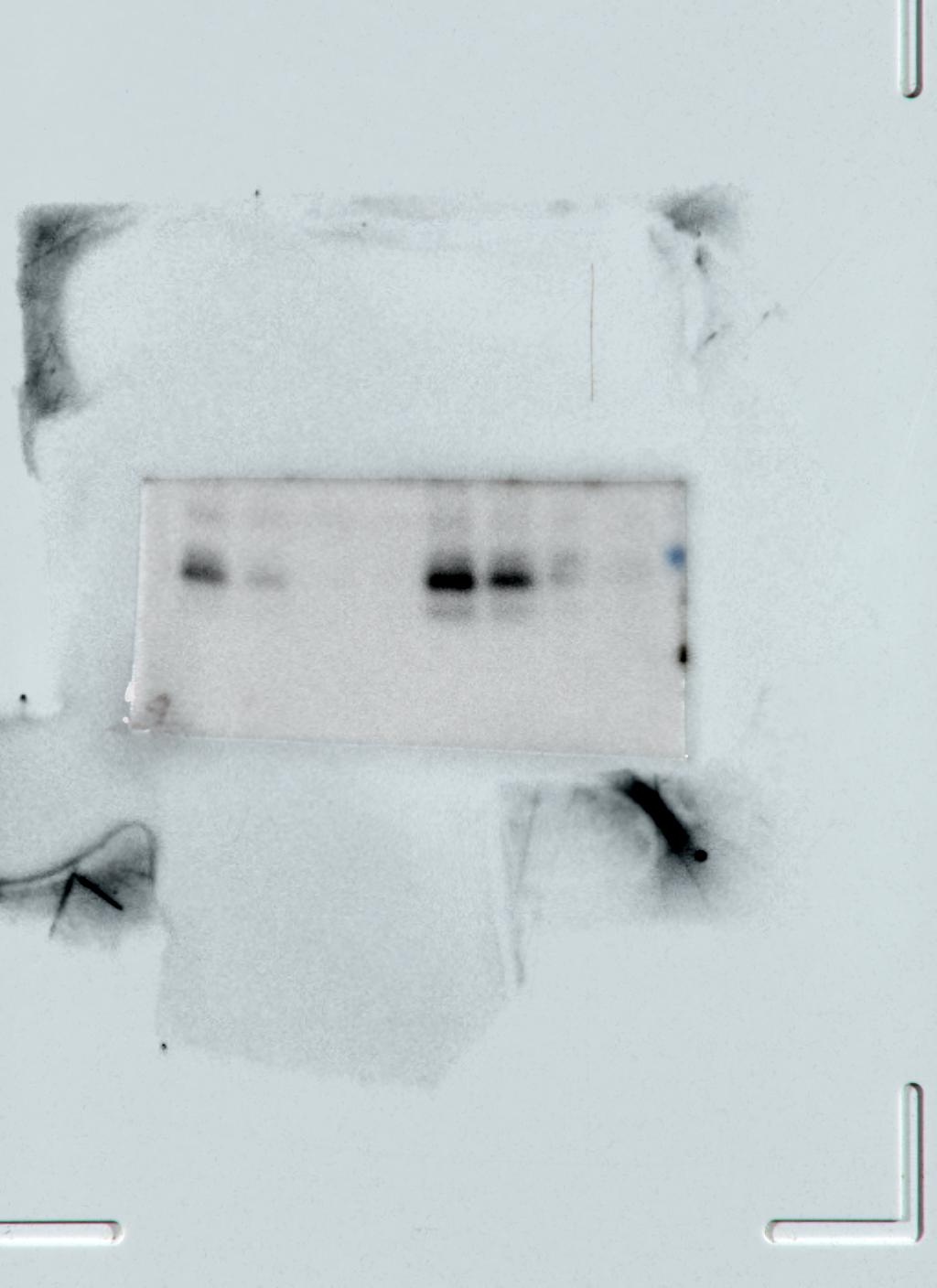

Supplement: Figure 3—figure supplement 1—source data 1. [file elife-98372-fig3-figsupp1-data1.zip › Figure 3-supplementary figure 1-data1/Figure_3-figure supplement_1_ source_data_1_ Figure_E_NOXA.jpg]

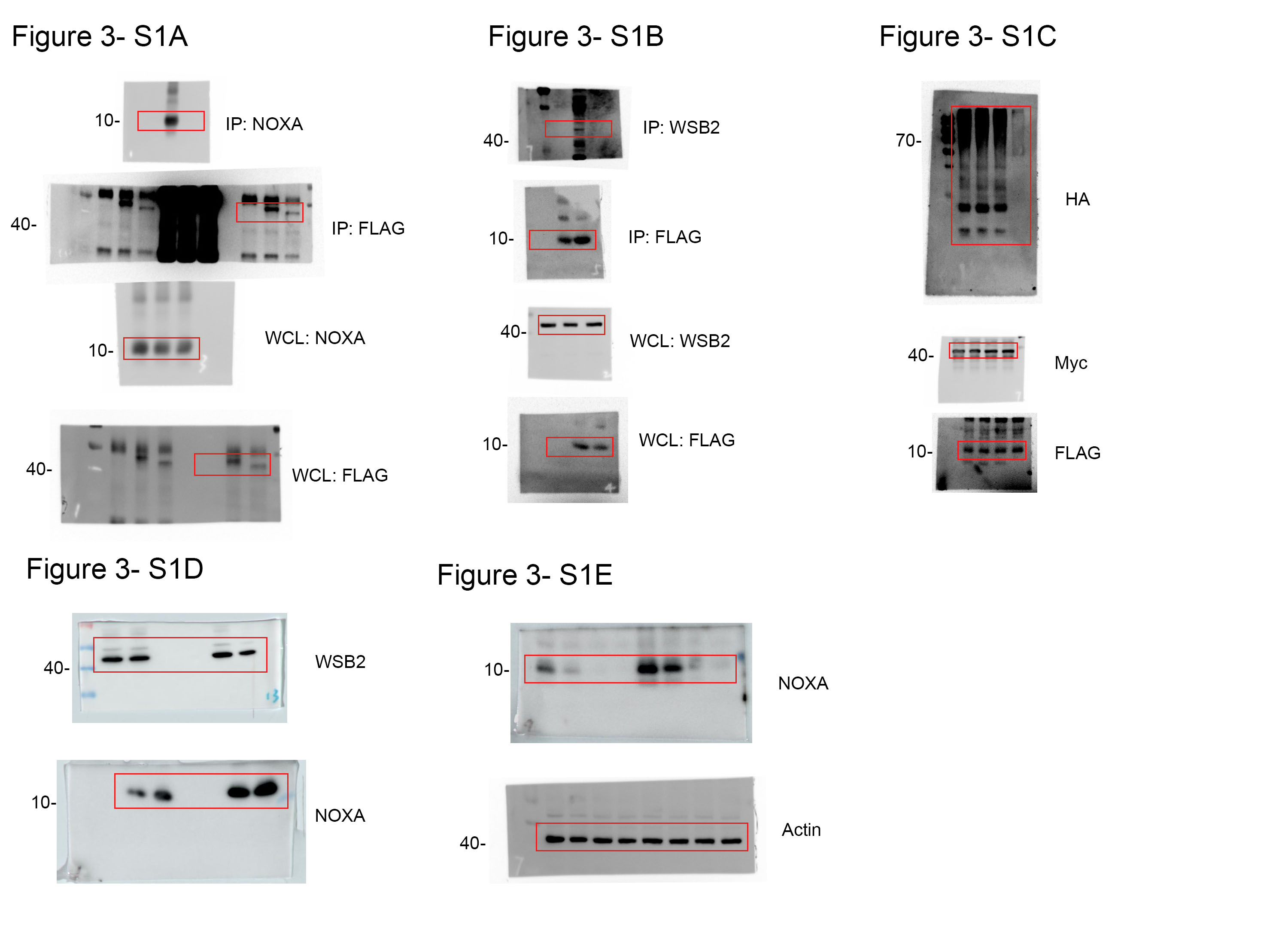

Supplement: Figure 3—figure supplement 1—source data 2. [file elife-98372-fig3-figsupp1-data2.zip › Figure 3-supplementary figure 1-data2/Figure_3_supplement figure_1_data_2.jpg]

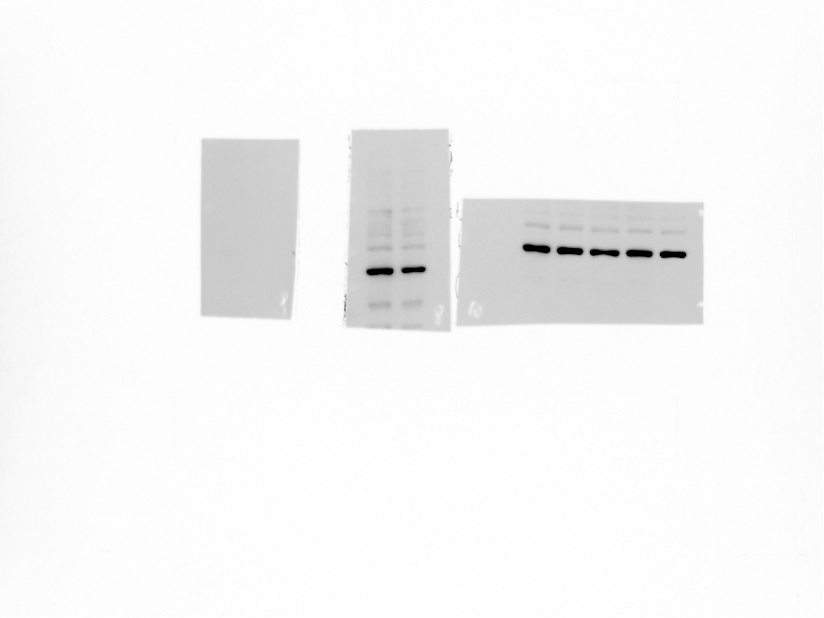

Supplement: Figure 4—source data 1. [file elife-98372-fig4-data1.zip › Figure 4-data1/Figure_4-source_data_1_ Figure_4A_Actin.jpg]

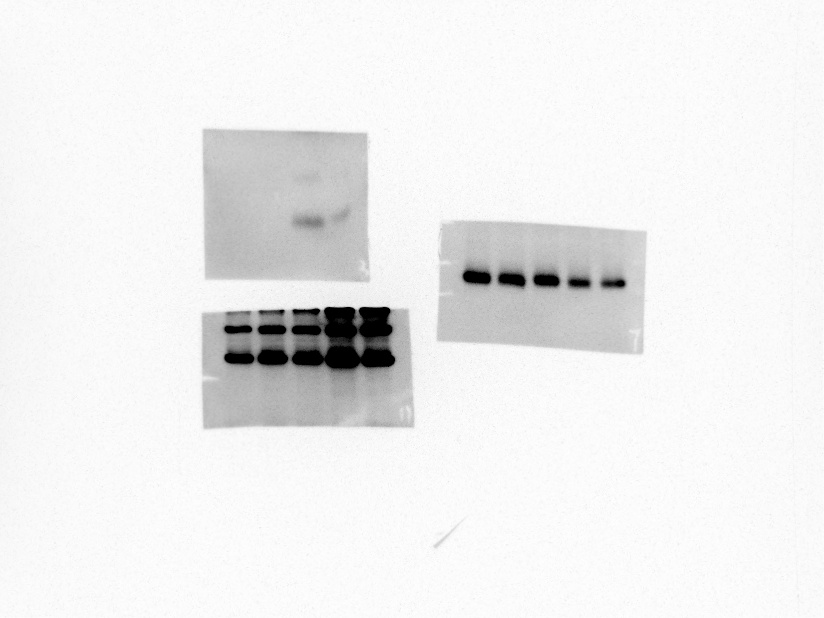

Supplement: Figure 4—source data 1. [file elife-98372-fig4-data1.zip › Figure 4-data1/Figure_4-source_data_1_ Figure_4A_CASP3.jpg]

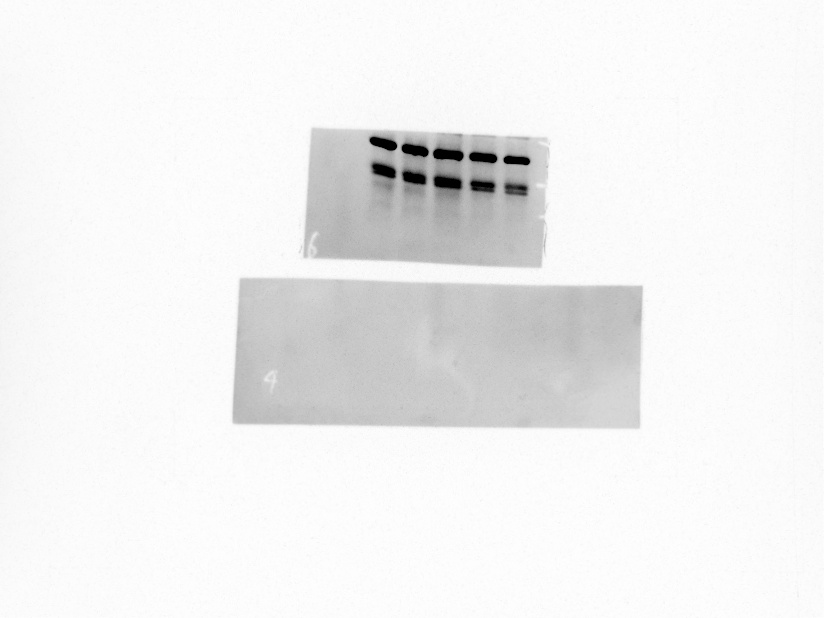

Supplement: Figure 4—source data 1. [file elife-98372-fig4-data1.zip › Figure 4-data1/Figure_4-source_data_1_ Figure_4A_CASP7.jpg]

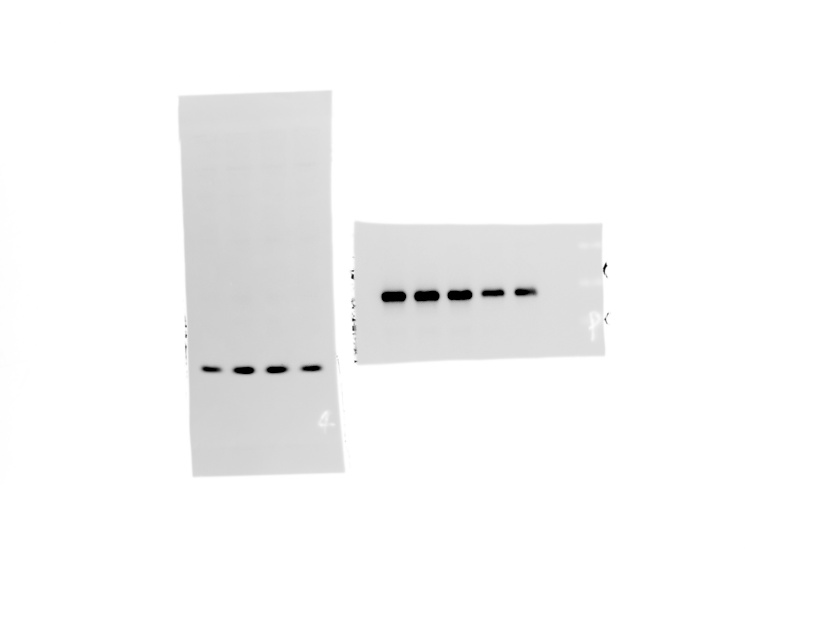

Supplement: Figure 4—source data 1. [file elife-98372-fig4-data1.zip › Figure 4-data1/Figure_4-source_data_1_ Figure_4A_CASP9.jpg]

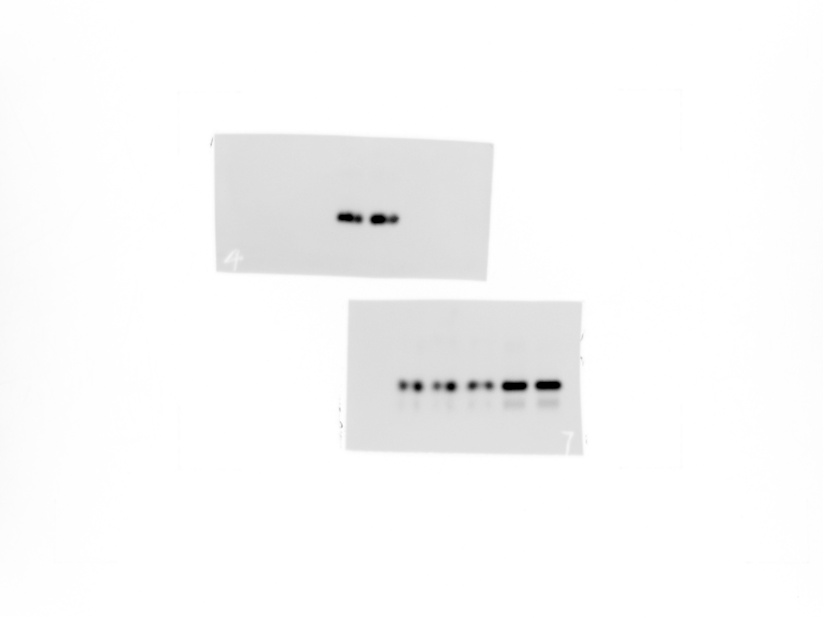

Supplement: Figure 4—source data 1. [file elife-98372-fig4-data1.zip › Figure 4-data1/Figure_4-source_data_1_ Figure_4A_cl-CASP3.jpg]

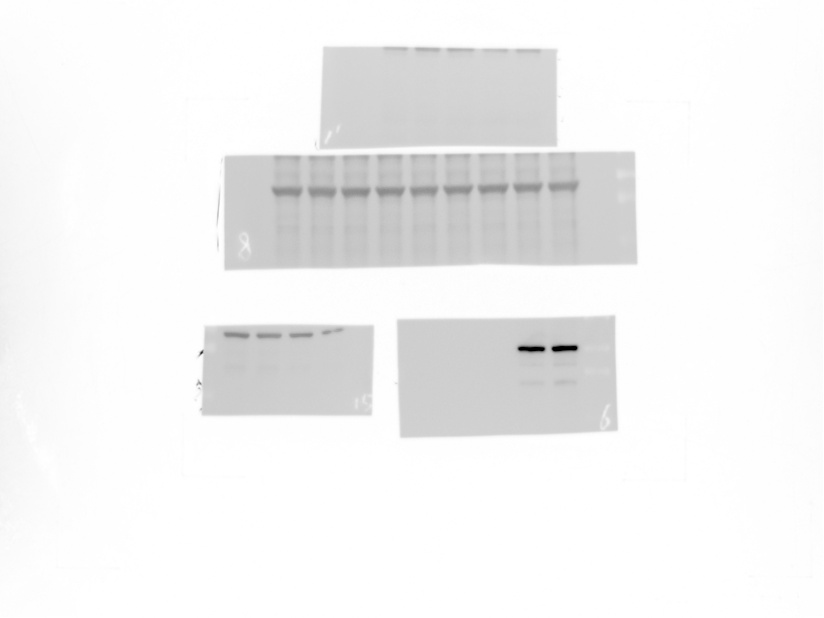

Supplement: Figure 4—source data 1. [file elife-98372-fig4-data1.zip › Figure 4-data1/Figure_4-source_data_1_ Figure_4A_cl-CASP7.jpg]

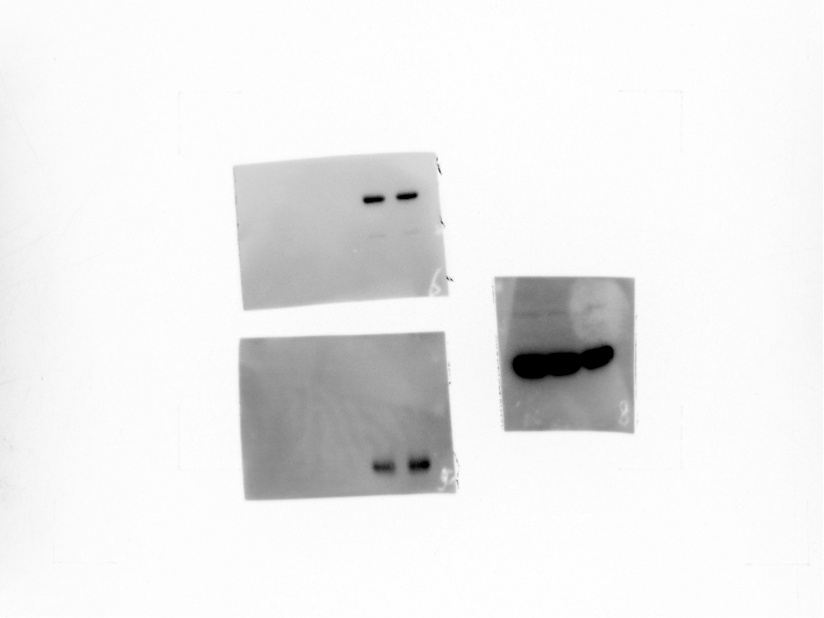

Supplement: Figure 4—source data 1. [file elife-98372-fig4-data1.zip › Figure 4-data1/Figure_4-source_data_1_ Figure_4A_cl-CASP9.jpg]

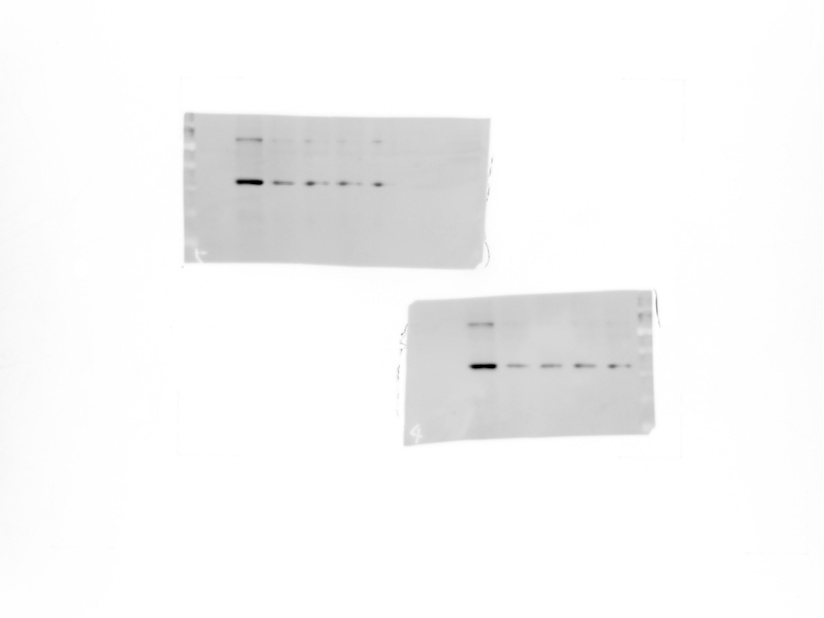

Supplement: Figure 4—source data 1. [file elife-98372-fig4-data1.zip › Figure 4-data1/Figure_4-source_data_1_ Figure_4A_MCL-1.jpg]

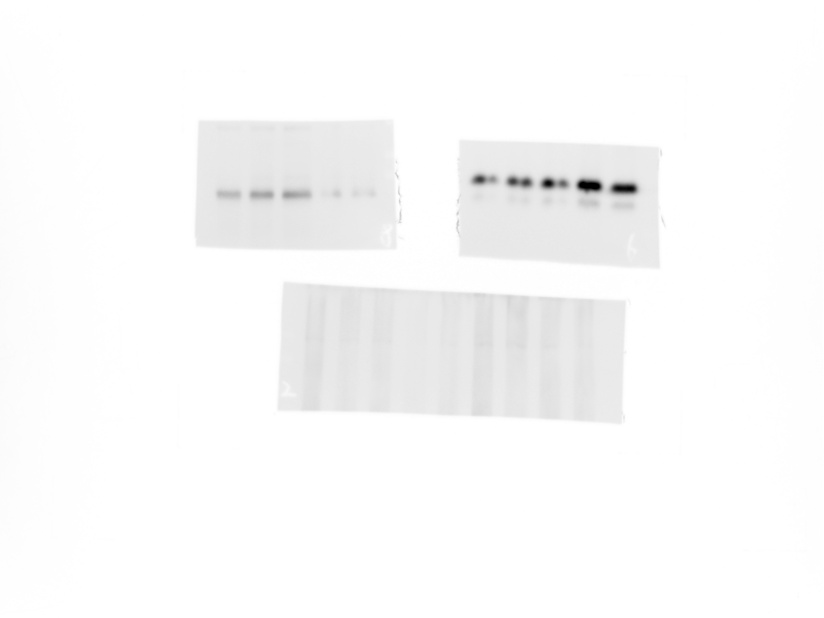

Supplement: Figure 4—source data 1. [file elife-98372-fig4-data1.zip › Figure 4-data1/Figure_4-source_data_1_ Figure_4A_NOXA.jpg]

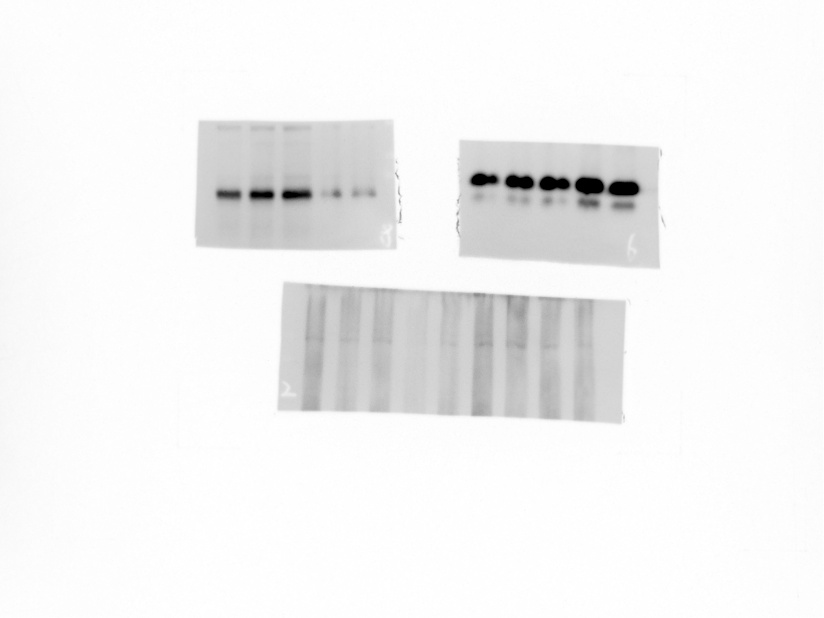

Supplement: Figure 4—source data 1. [file elife-98372-fig4-data1.zip › Figure 4-data1/Figure_4-source_data_1_ Figure_4A_WSB2.jpg]

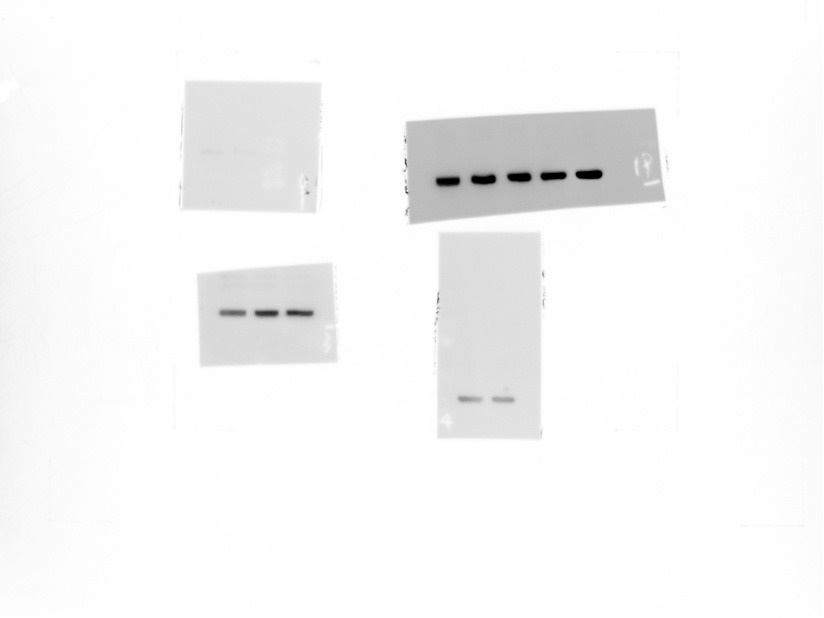

Supplement: Figure 4—source data 1. [file elife-98372-fig4-data1.zip › Figure 4-data1/Figure_4-source_data_1_ Figure_4C_Actin.jpg]

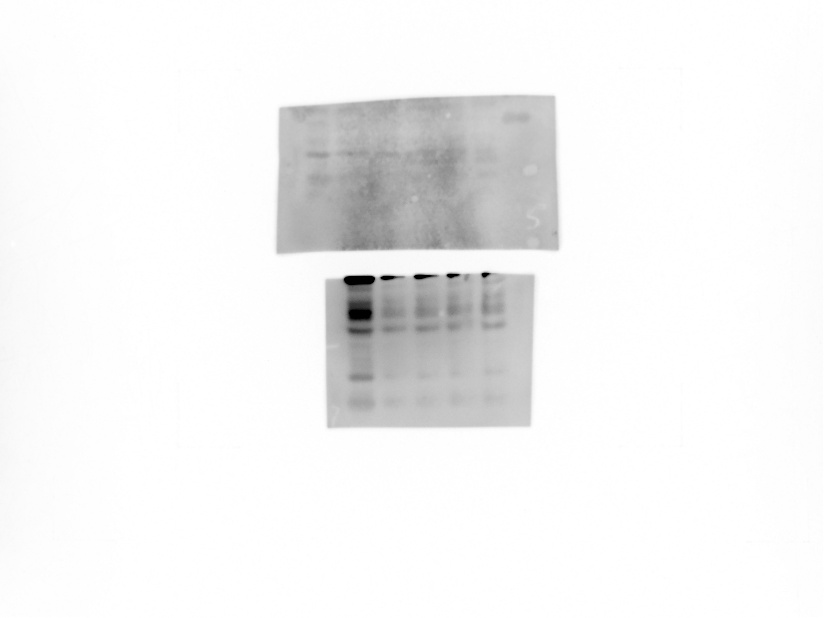

Supplement: Figure 4—source data 1. [file elife-98372-fig4-data1.zip › Figure 4-data1/Figure_4-source_data_1_ Figure_4C_BCL-XL.jpg]

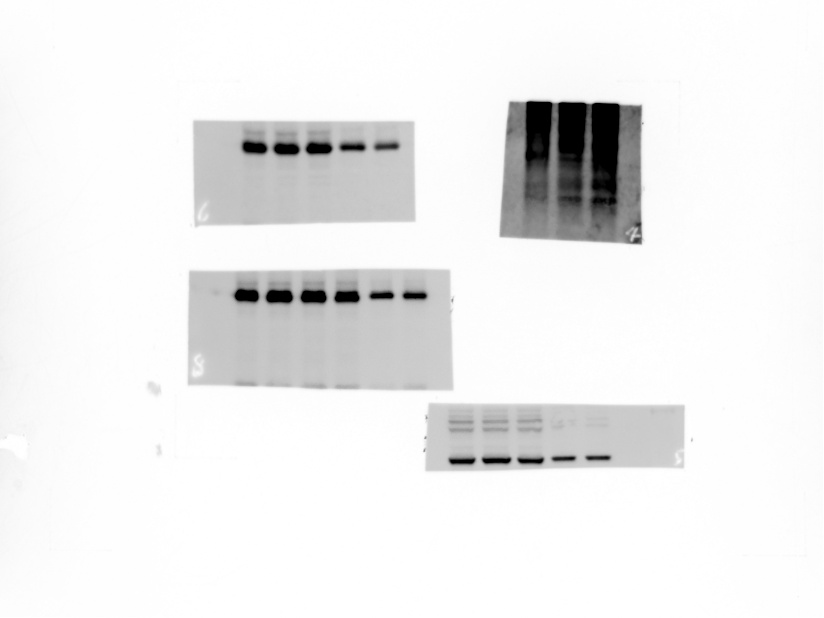

Supplement: Figure 4—source data 1. [file elife-98372-fig4-data1.zip › Figure 4-data1/Figure_4-source_data_1_ Figure_4C_CASP3.jpg]

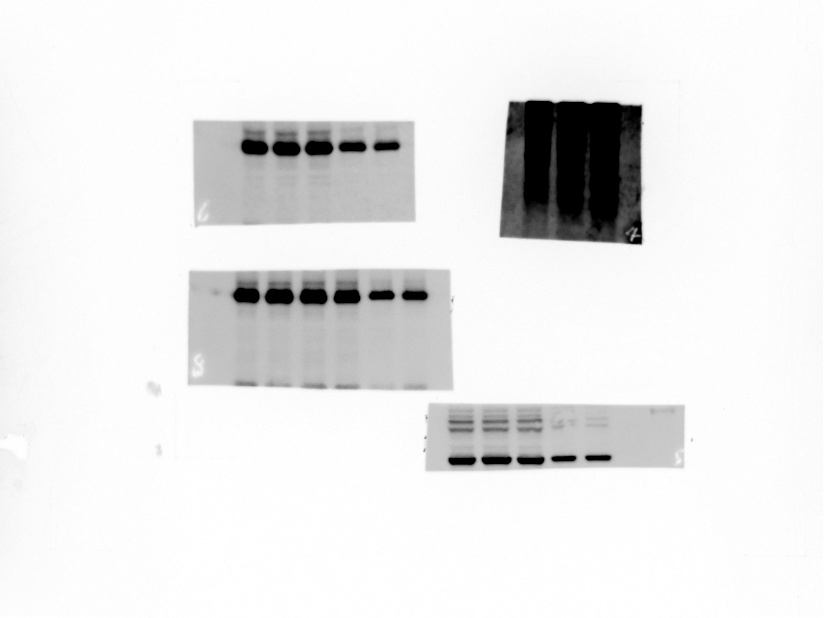

Supplement: Figure 4—source data 1. [file elife-98372-fig4-data1.zip › Figure 4-data1/Figure_4-source_data_1_ Figure_4C_CASP7.jpg]

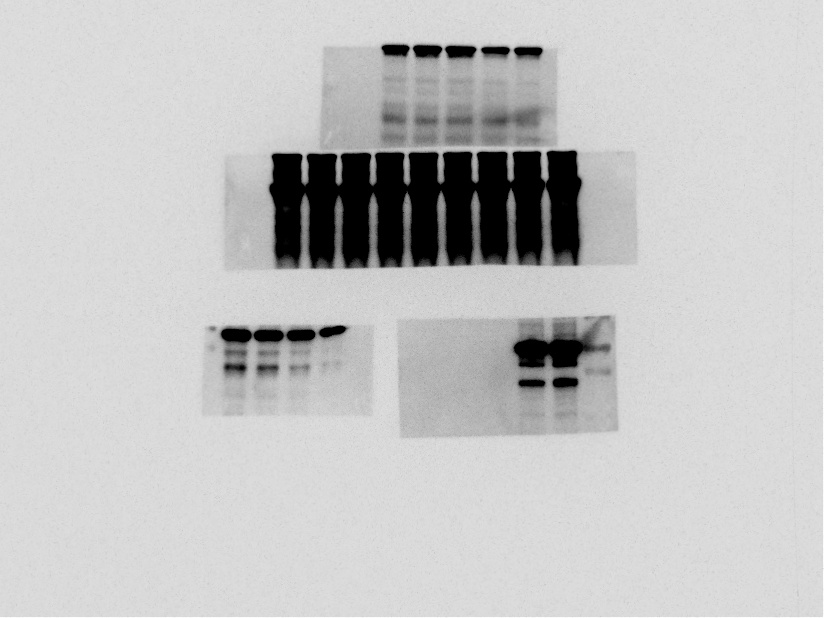

Supplement: Figure 4—source data 1. [file elife-98372-fig4-data1.zip › Figure 4-data1/Figure_4-source_data_1_ Figure_4C_CASP9.jpg]

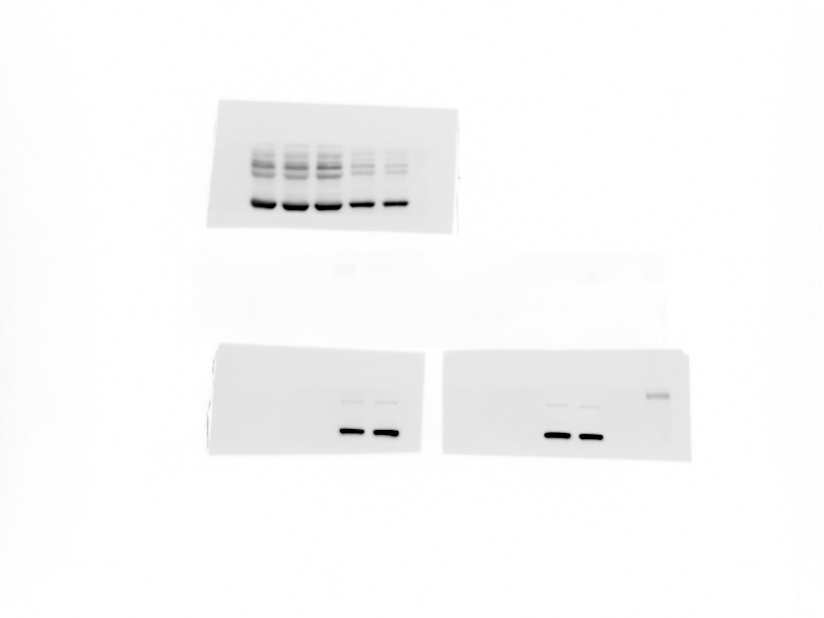

Supplement: Figure 4—source data 1. [file elife-98372-fig4-data1.zip › Figure 4-data1/Figure_4-source_data_1_ Figure_4C_cl-CASP3.jpg]

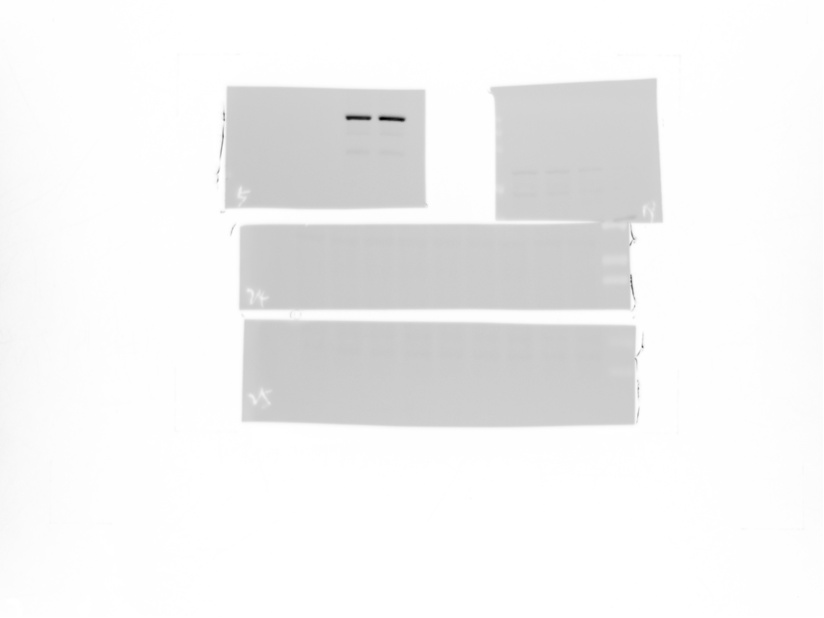

Supplement: Figure 4—source data 1. [file elife-98372-fig4-data1.zip › Figure 4-data1/Figure_4-source_data_1_ Figure_4C_cl-CASP9.jpg]

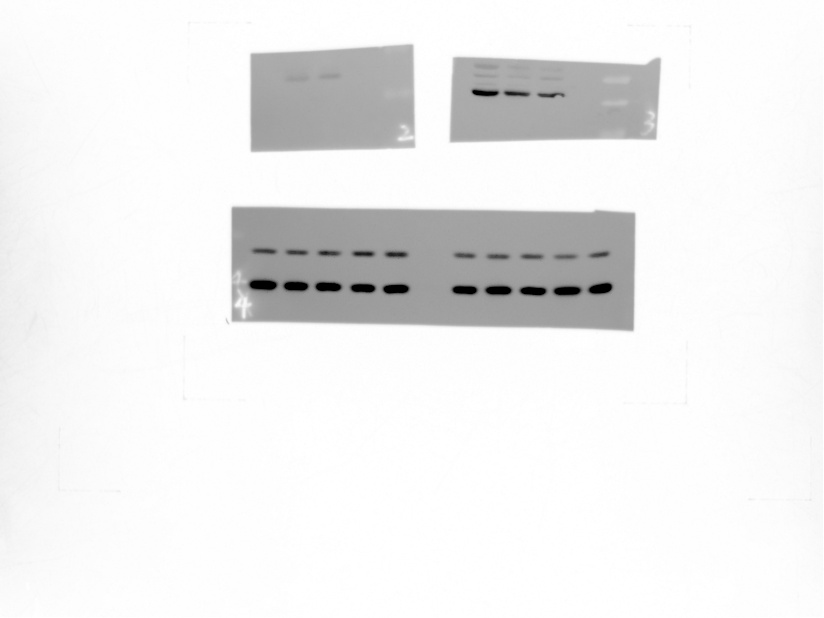

Supplement: Figure 4—source data 1. [file elife-98372-fig4-data1.zip › Figure 4-data1/Figure_4-source_data_1_ Figure_4C_MCL-1.jpg]

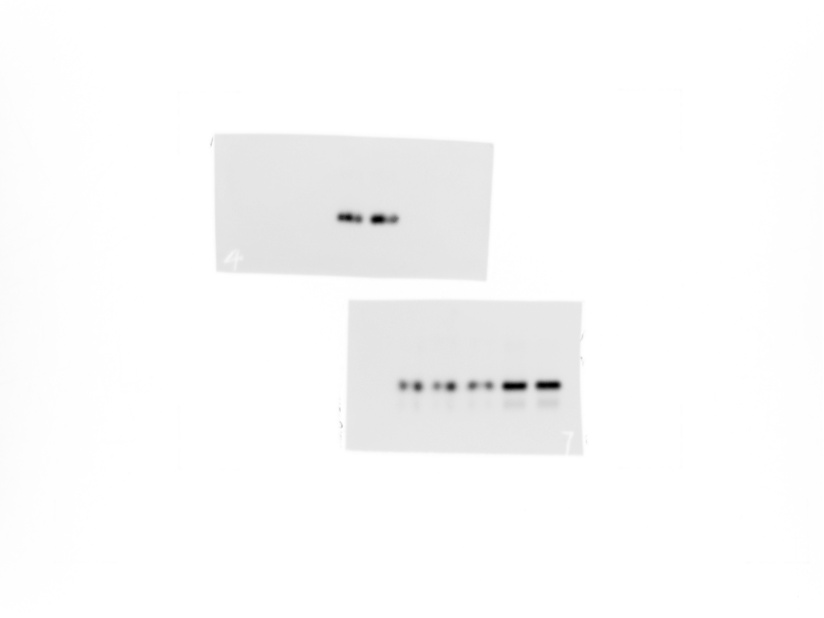

Supplement: Figure 4—source data 1. [file elife-98372-fig4-data1.zip › Figure 4-data1/Figure_4-source_data_1_ Figure_4C_NOXA.jpg]

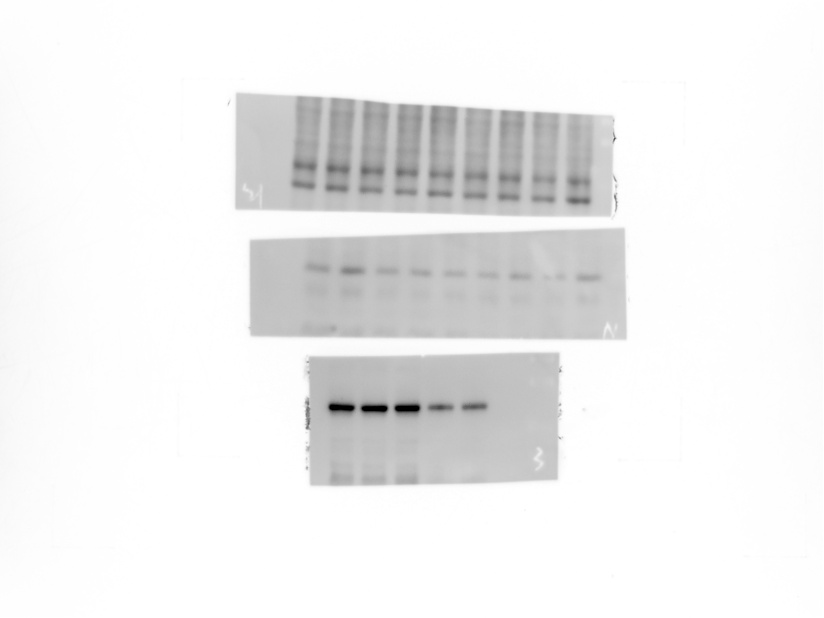

Supplement: Figure 4—source data 1. [file elife-98372-fig4-data1.zip › Figure 4-data1/Figure_4-source_data_1_ Figure_4C_WSB2.jpg]
