# Supplementary figures and images for: Targeting the WSB2–NOXA axis in cancer cells for enhanced sensitivity to BCL-2 family protein inhibitors (part 3 of 5)
Source: eLife. 2025 Jul 23;13:RP98372. doi: 10.7554/eLife.98372 (PMC12286604; doi:10.7554/eLife.98372)

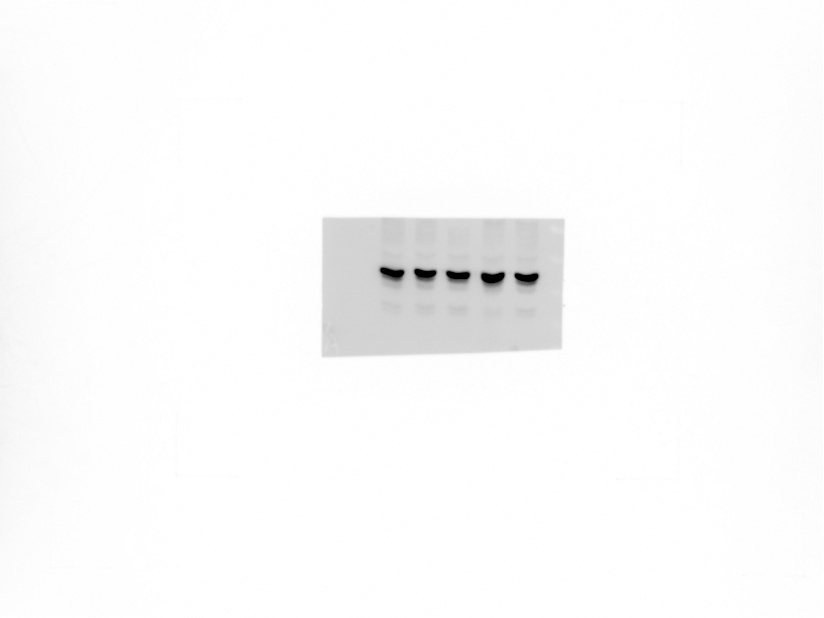

Supplement: Figure 4—source data 1. [file elife-98372-fig4-data1.zip › Figure 4-data1/Figure_4-source_data_1_ Figure_4E_Actin.jpg]

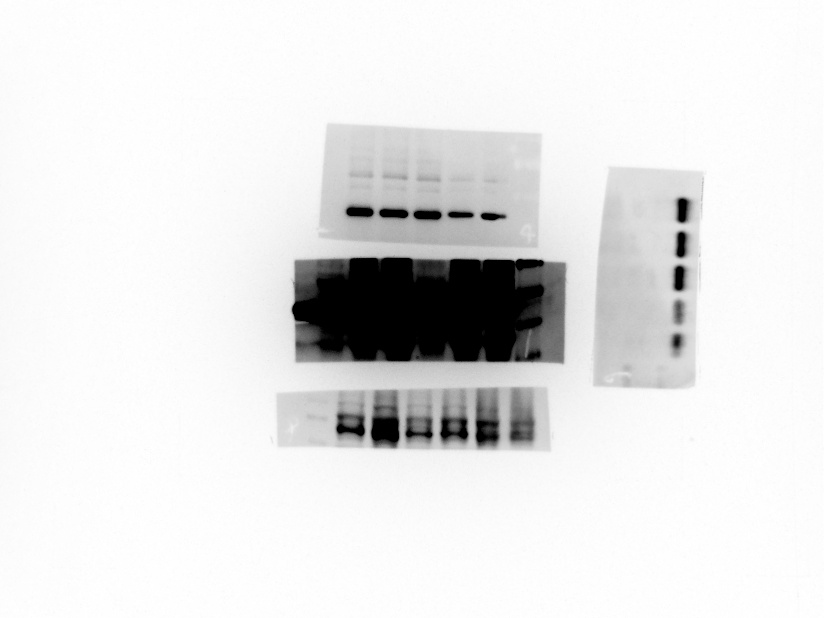

Supplement: Figure 4—source data 1. [file elife-98372-fig4-data1.zip › Figure 4-data1/Figure_4-source_data_1_ Figure_4E_CASP3.jpg]

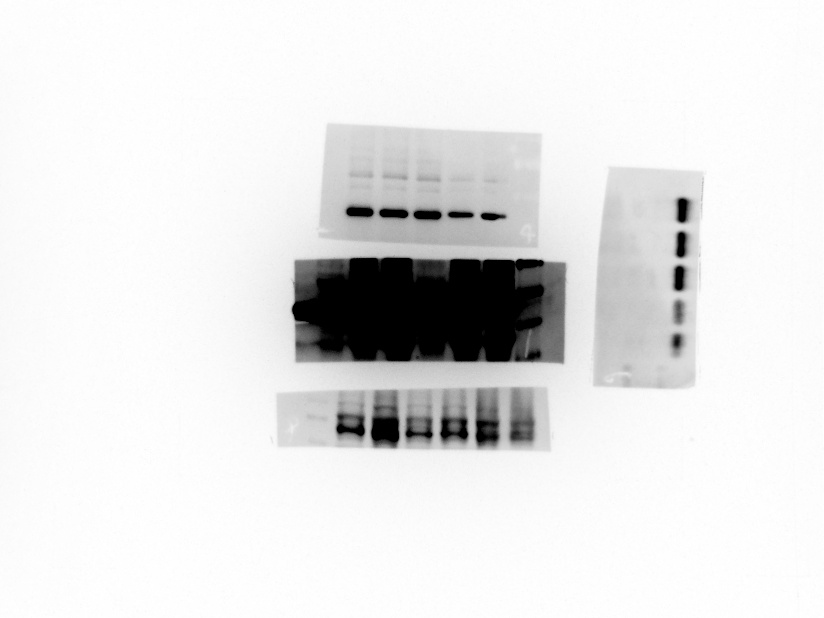

Supplement: Figure 4—source data 1. [file elife-98372-fig4-data1.zip › Figure 4-data1/Figure_4-source_data_1_ Figure_4E_CASP7.jpg]

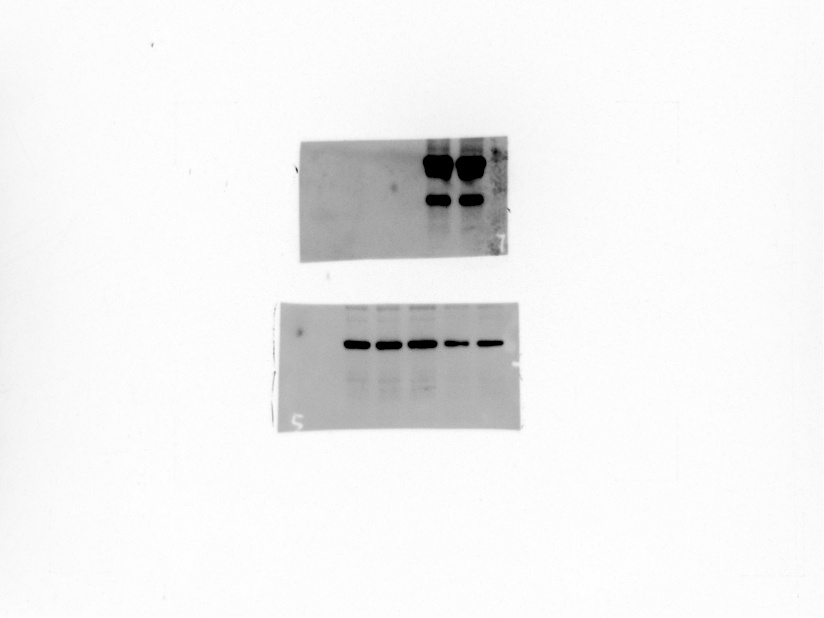

Supplement: Figure 4—source data 1. [file elife-98372-fig4-data1.zip › Figure 4-data1/Figure_4-source_data_1_ Figure_4E_CASP9.jpg]

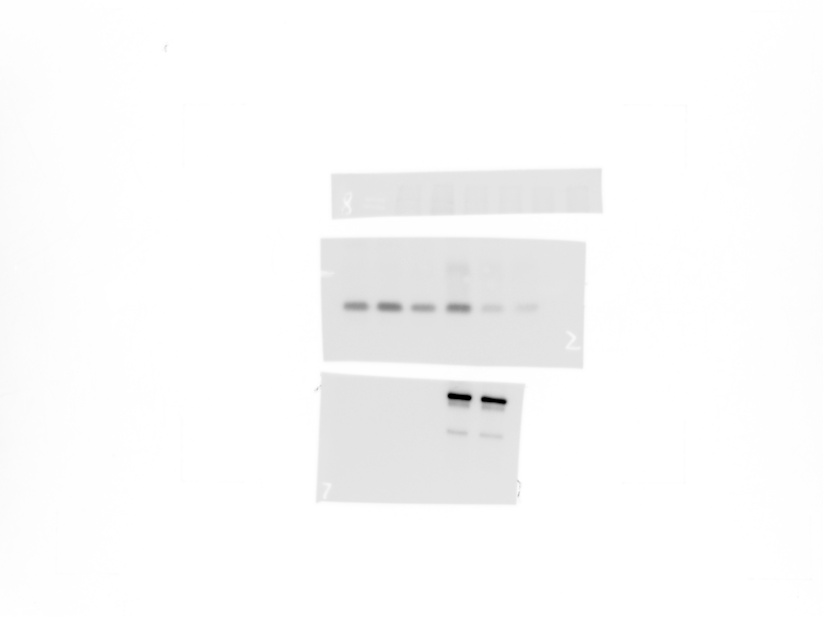

Supplement: Figure 4—source data 1. [file elife-98372-fig4-data1.zip › Figure 4-data1/Figure_4-source_data_1_ Figure_4E_cl-CASP3.jpg]

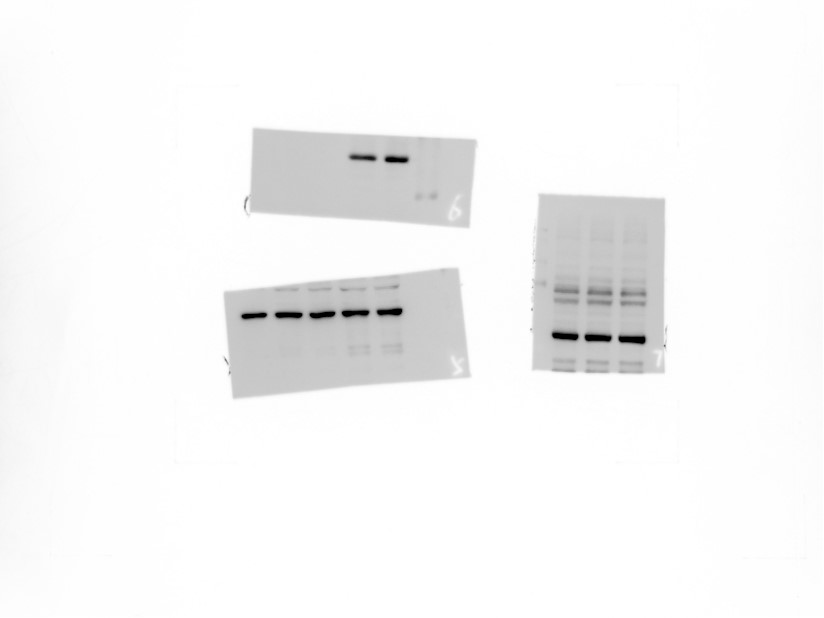

Supplement: Figure 4—source data 1. [file elife-98372-fig4-data1.zip › Figure 4-data1/Figure_4-source_data_1_ Figure_4E_cl-CASP7.jpg]

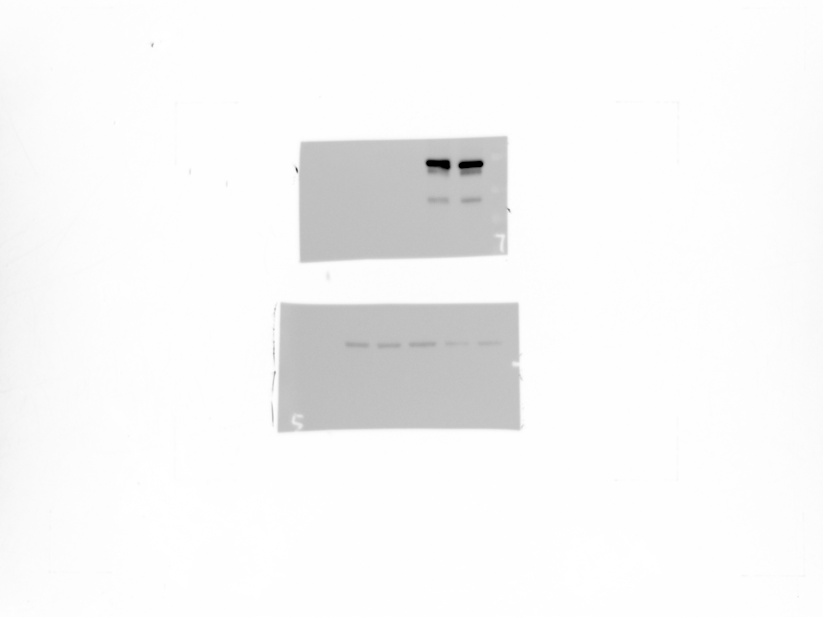

Supplement: Figure 4—source data 1. [file elife-98372-fig4-data1.zip › Figure 4-data1/Figure_4-source_data_1_ Figure_4E_cl-CASP9.jpg]

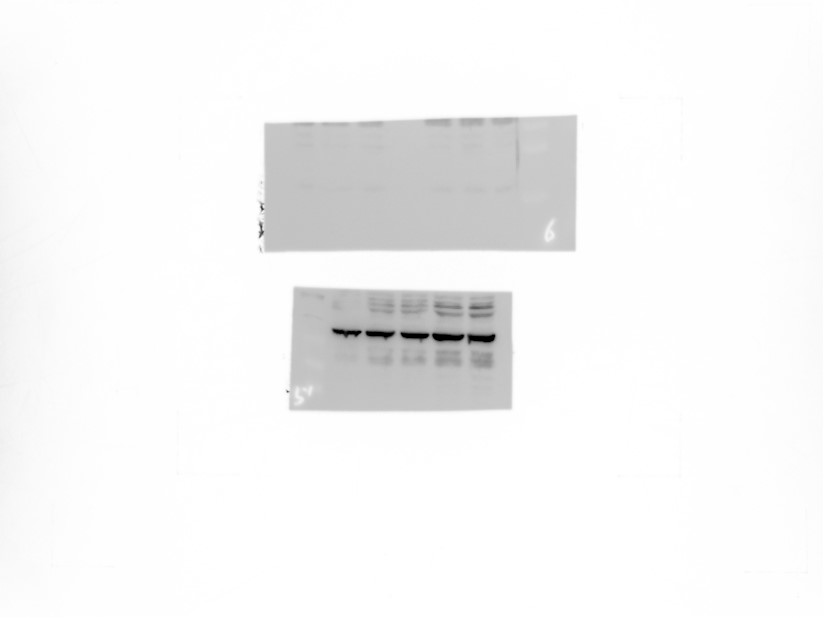

Supplement: Figure 4—source data 1. [file elife-98372-fig4-data1.zip › Figure 4-data1/Figure_4-source_data_1_ Figure_4E_MCL-1.jpg]

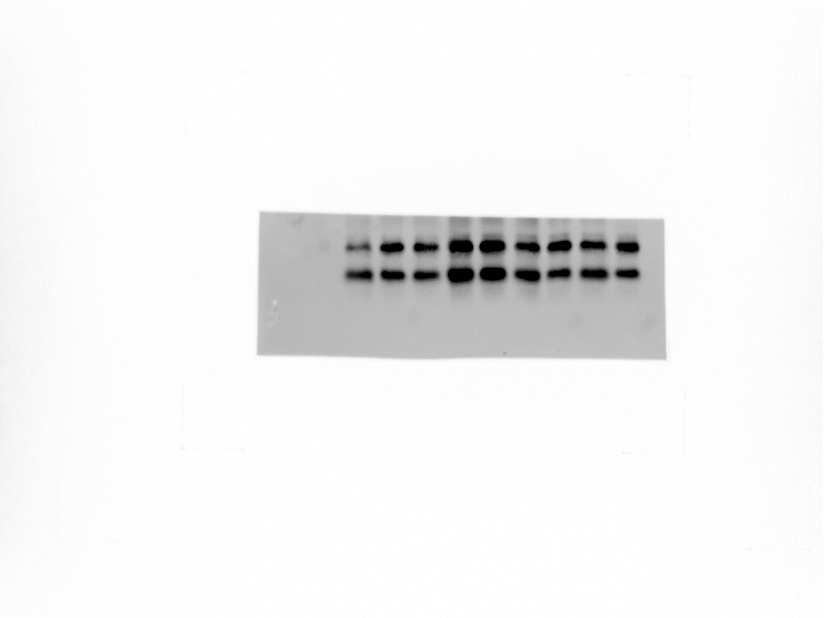

Supplement: Figure 4—source data 1. [file elife-98372-fig4-data1.zip › Figure 4-data1/Figure_4-source_data_1_ Figure_4E_NOXA.jpg]

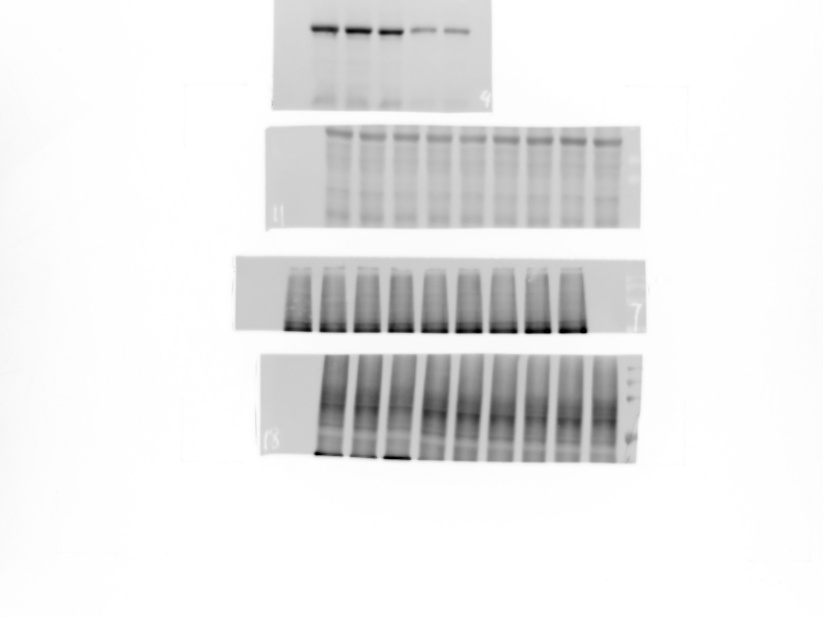

Supplement: Figure 4—source data 1. [file elife-98372-fig4-data1.zip › Figure 4-data1/Figure_4-source_data_1_ Figure_4E_WSB2.jpg]

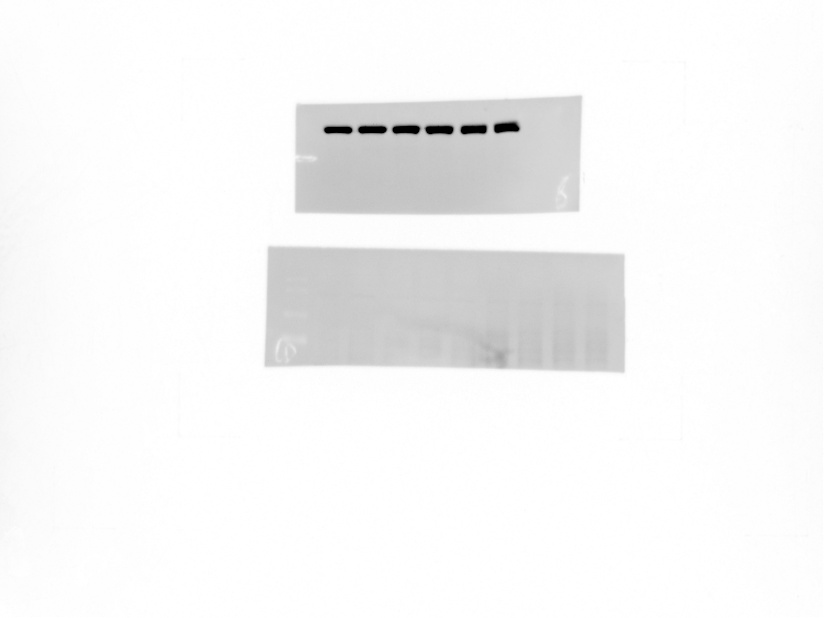

Supplement: Figure 4—source data 1. [file elife-98372-fig4-data1.zip › Figure 4-data1/Figure_4-source_data_1_ Figure_4G_Actin.jpg]

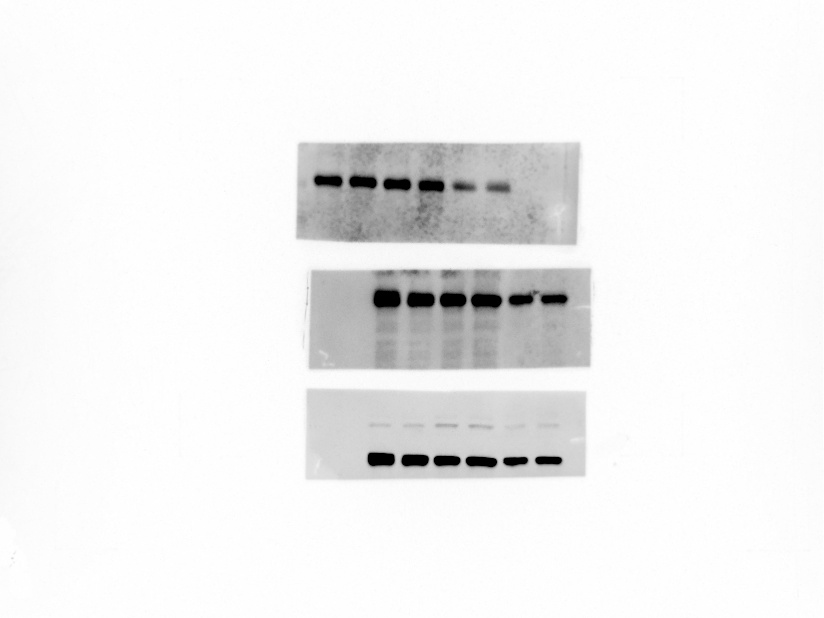

Supplement: Figure 4—source data 1. [file elife-98372-fig4-data1.zip › Figure 4-data1/Figure_4-source_data_1_ Figure_4G_CASP3.jpg]

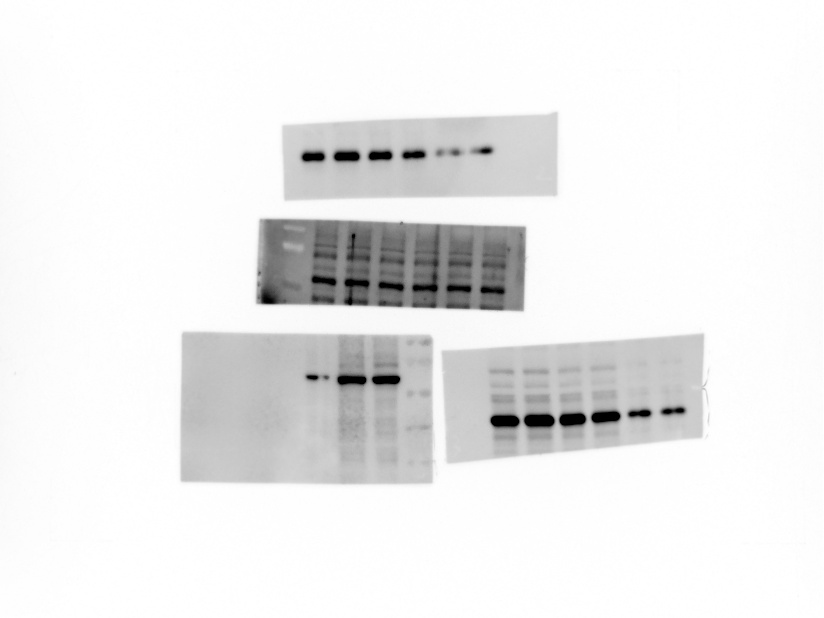

Supplement: Figure 4—source data 1. [file elife-98372-fig4-data1.zip › Figure 4-data1/Figure_4-source_data_1_ Figure_4G_CASP7.jpg]

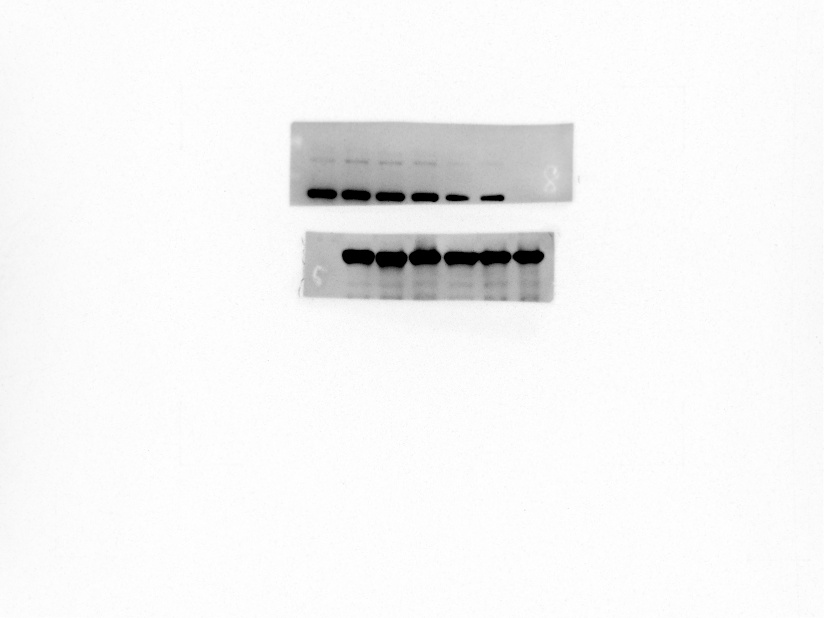

Supplement: Figure 4—source data 1. [file elife-98372-fig4-data1.zip › Figure 4-data1/Figure_4-source_data_1_ Figure_4G_CASP9.jpg]

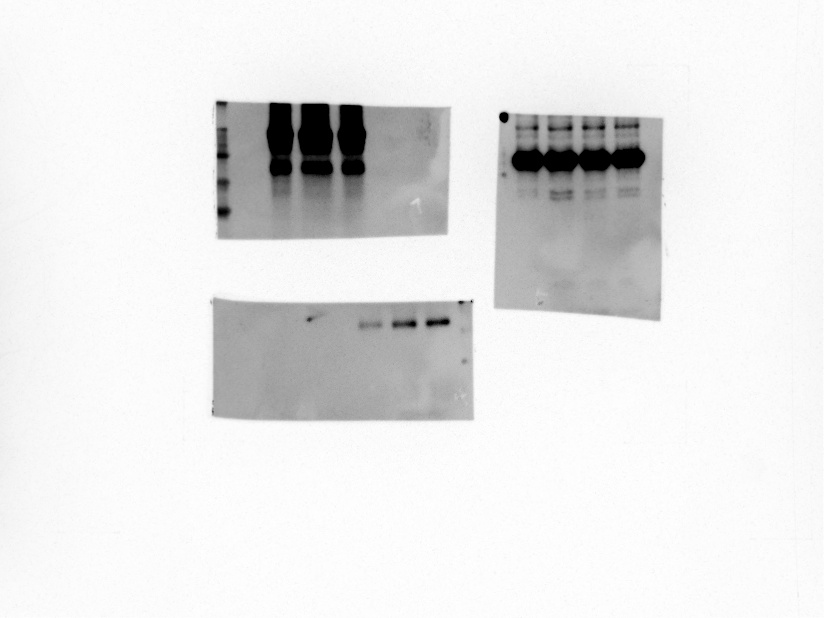

Supplement: Figure 4—source data 1. [file elife-98372-fig4-data1.zip › Figure 4-data1/Figure_4-source_data_1_ Figure_4G_cl-CASP3.jpg]

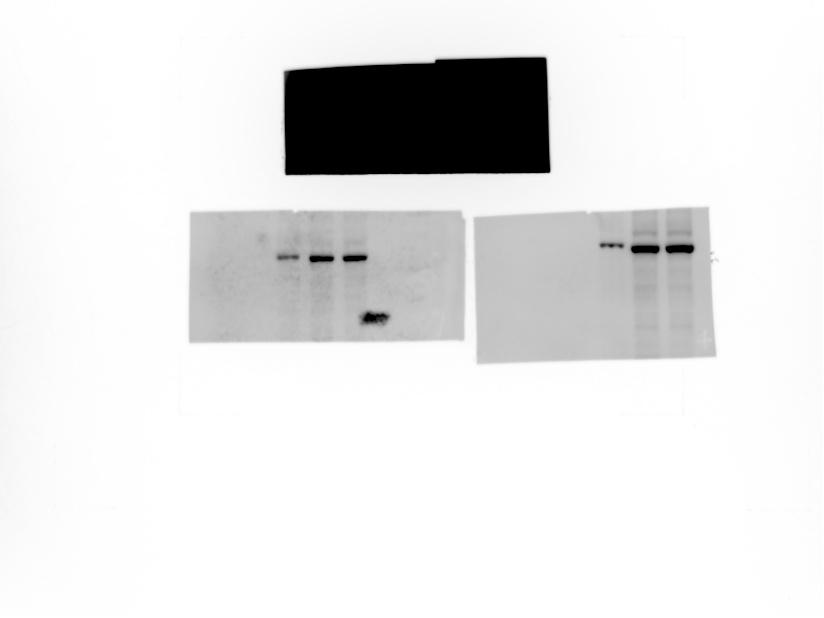

Supplement: Figure 4—source data 1. [file elife-98372-fig4-data1.zip › Figure 4-data1/Figure_4-source_data_1_ Figure_4G_cl-CASP7.jpg]

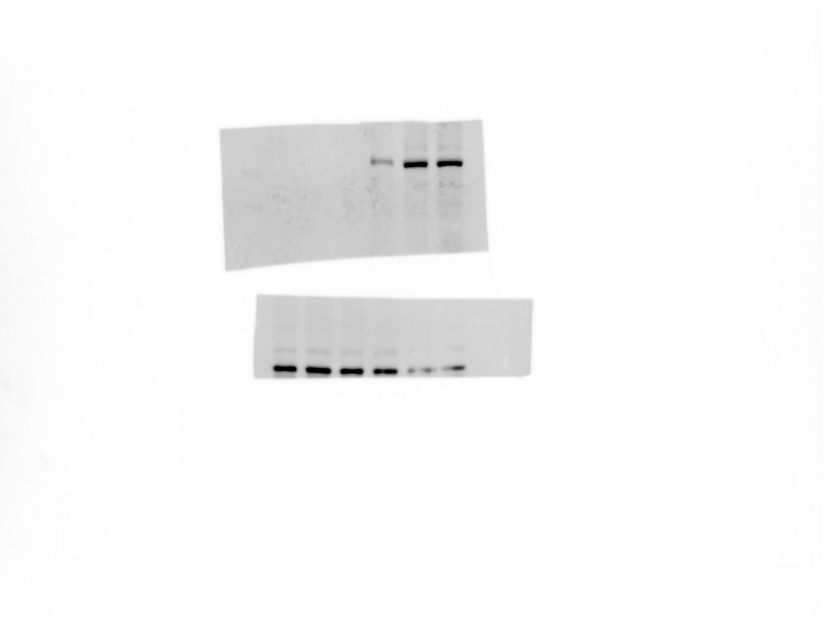

Supplement: Figure 4—source data 1. [file elife-98372-fig4-data1.zip › Figure 4-data1/Figure_4-source_data_1_ Figure_4G_cl-CASP9.jpg]

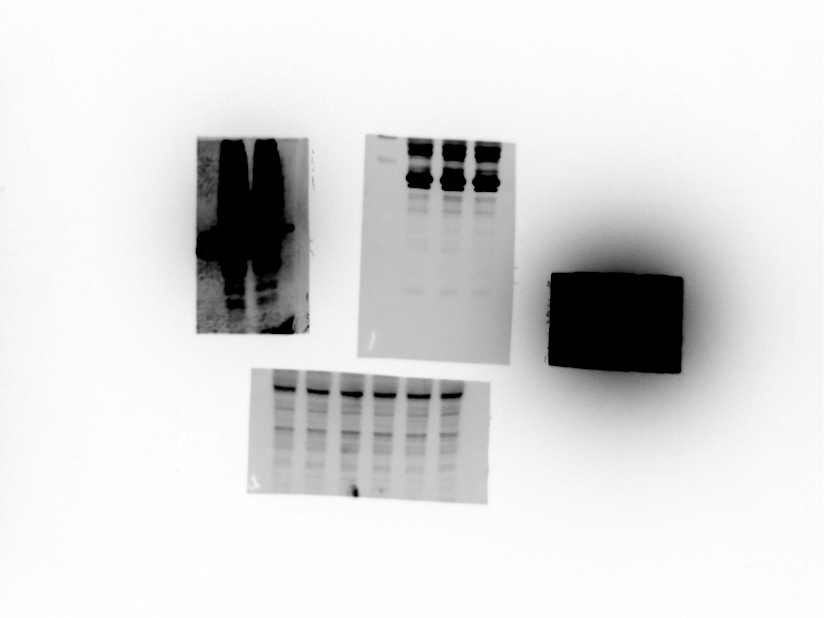

Supplement: Figure 4—source data 1. [file elife-98372-fig4-data1.zip › Figure 4-data1/Figure_4-source_data_1_ Figure_4G_MCL-1.jpg]

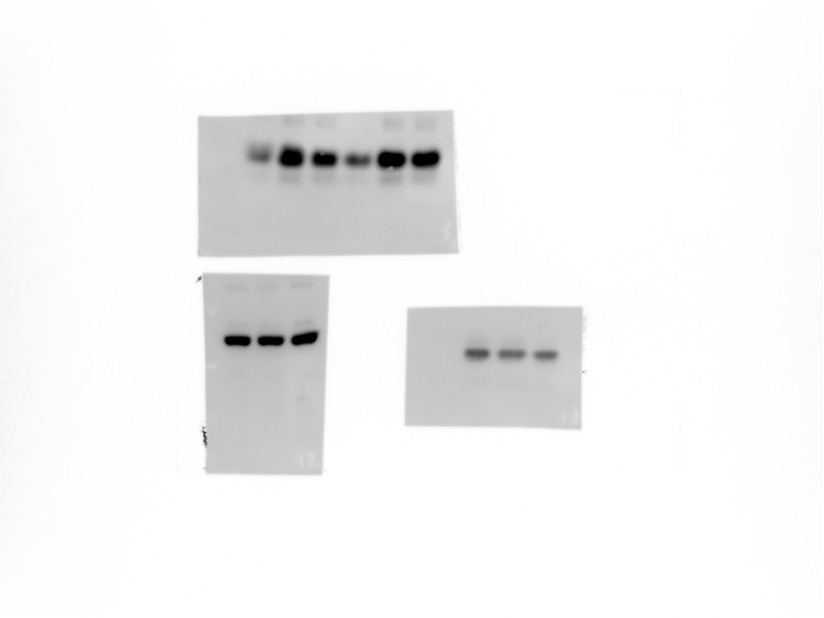

Supplement: Figure 4—source data 1. [file elife-98372-fig4-data1.zip › Figure 4-data1/Figure_4-source_data_1_ Figure_4G_NOXA.jpg]

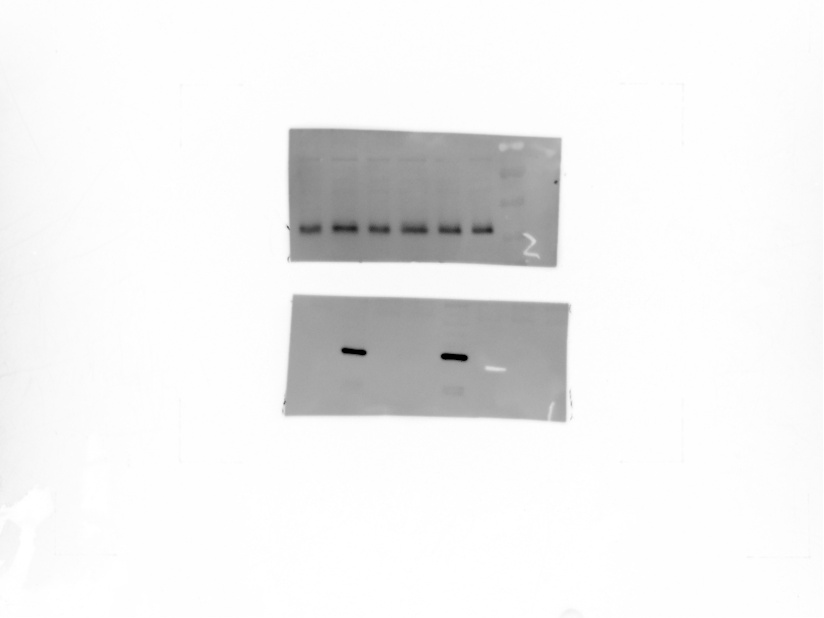

Supplement: Figure 4—source data 1. [file elife-98372-fig4-data1.zip › Figure 4-data1/Figure_4-source_data_1_ Figure_4G_WSB2.jpg]

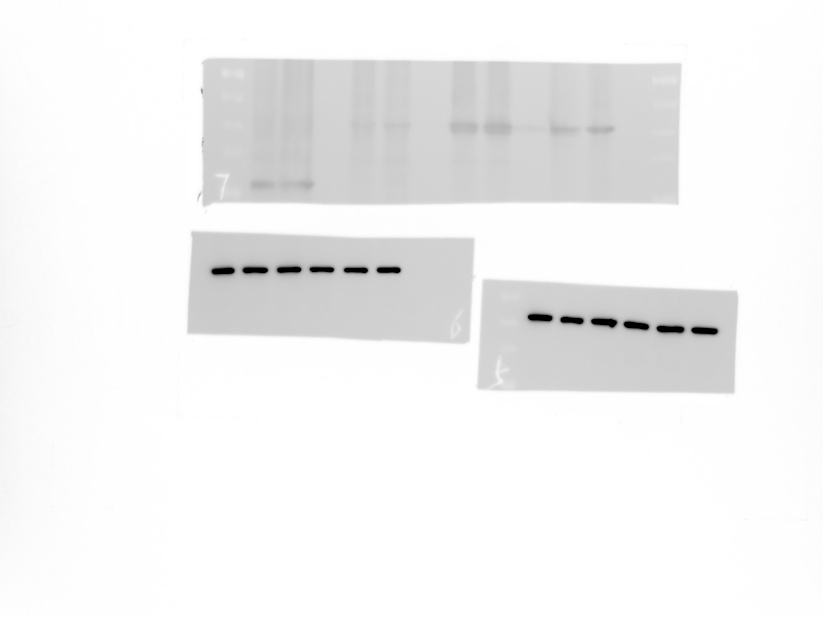

Supplement: Figure 4—source data 1. [file elife-98372-fig4-data1.zip › Figure 4-data1/Figure_4-source_data_1_ Figure_4I_Actin.jpg]

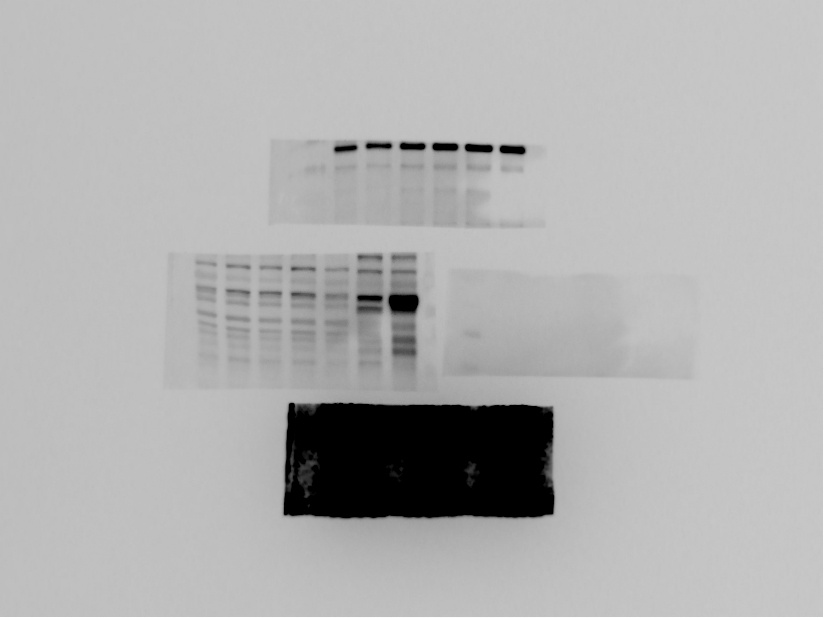

Supplement: Figure 4—source data 1. [file elife-98372-fig4-data1.zip › Figure 4-data1/Figure_4-source_data_1_ Figure_4I_CASP7.jpg]

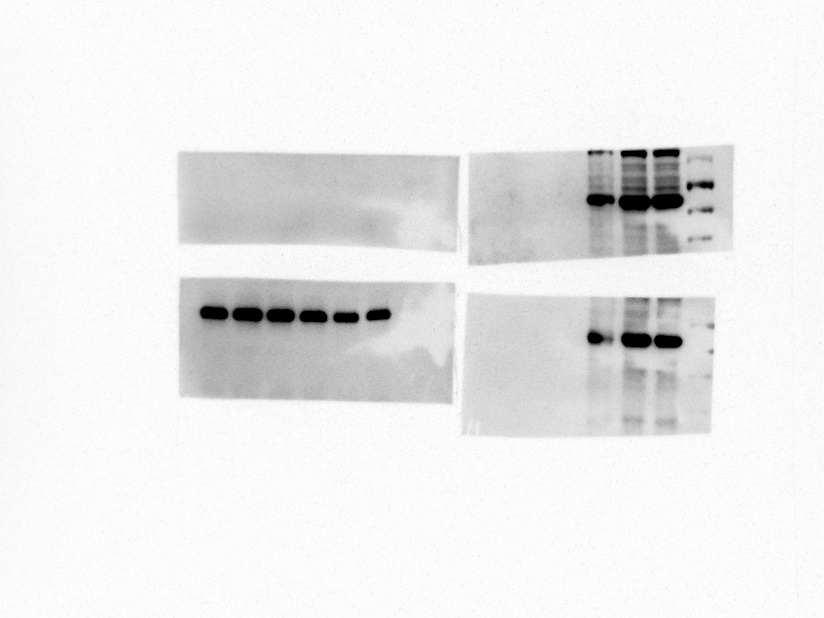

Supplement: Figure 4—source data 1. [file elife-98372-fig4-data1.zip › Figure 4-data1/Figure_4-source_data_1_ Figure_4I_CASP9.jpg]

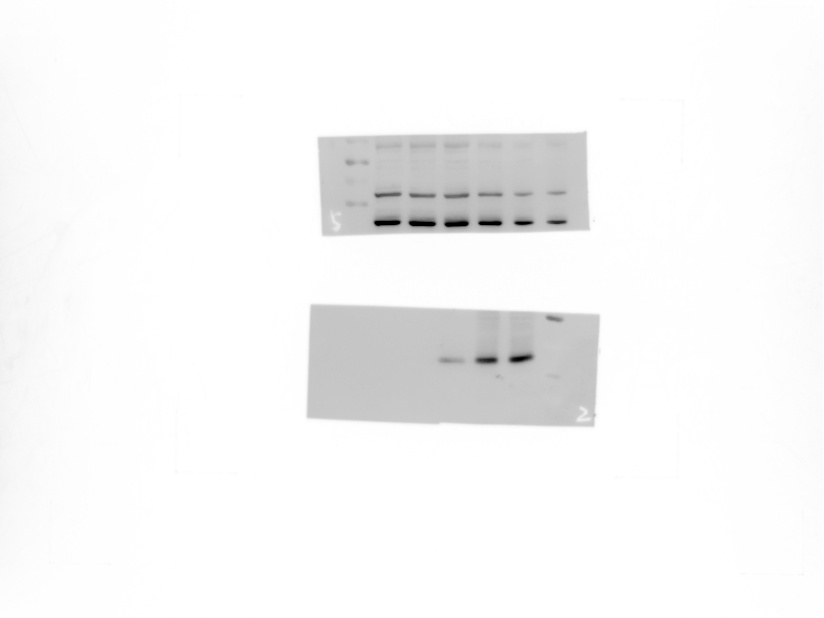

Supplement: Figure 4—source data 1. [file elife-98372-fig4-data1.zip › Figure 4-data1/Figure_4-source_data_1_ Figure_4I_cl-CASP3.jpg]

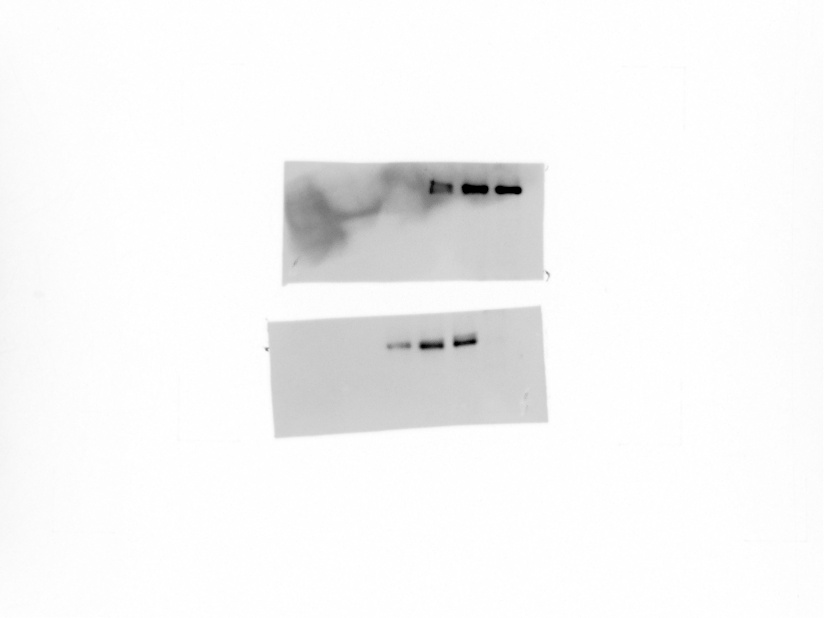

Supplement: Figure 4—source data 1. [file elife-98372-fig4-data1.zip › Figure 4-data1/Figure_4-source_data_1_ Figure_4I_cl-CASP7.jpg]

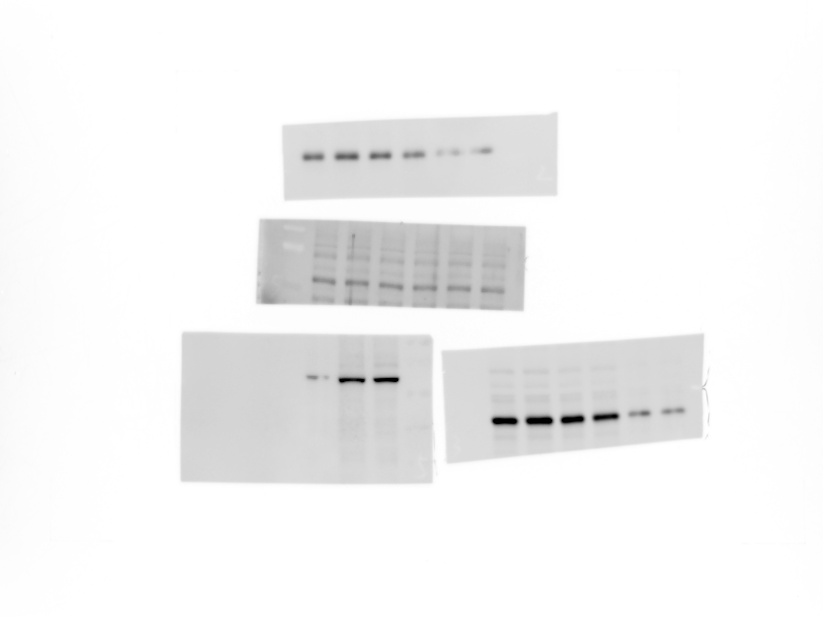

Supplement: Figure 4—source data 1. [file elife-98372-fig4-data1.zip › Figure 4-data1/Figure_4-source_data_1_ Figure_4I_cl-CASP9.jpg]

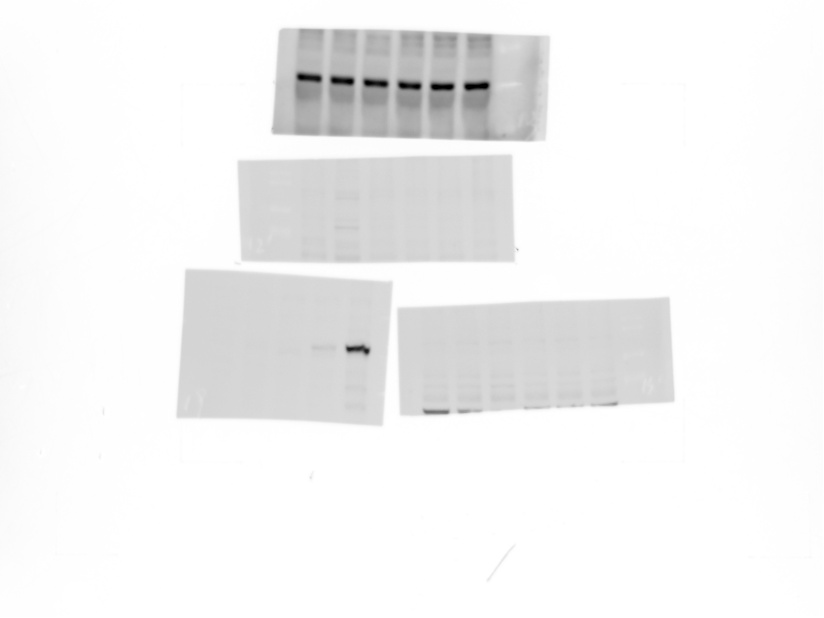

Supplement: Figure 4—source data 1. [file elife-98372-fig4-data1.zip › Figure 4-data1/Figure_4-source_data_1_ Figure_4I_MCL-1.jpg]

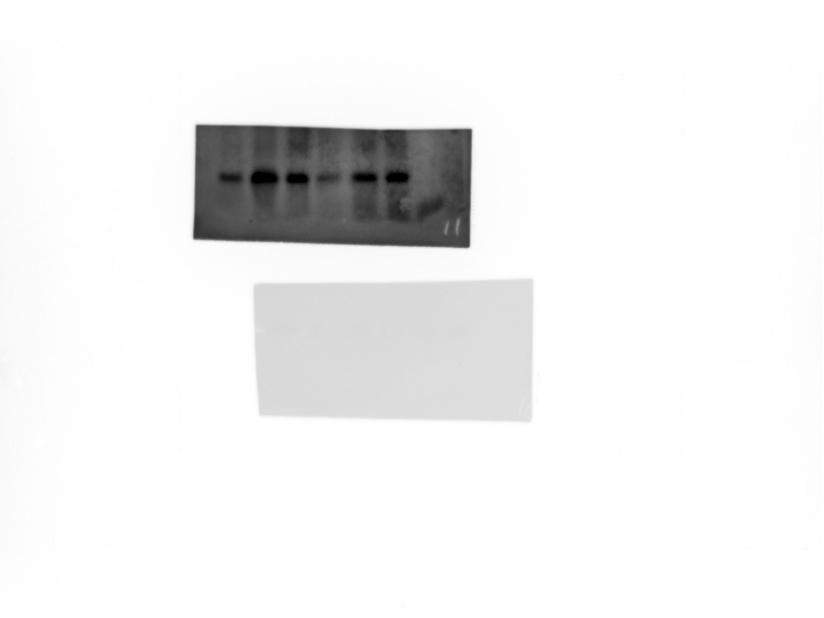

Supplement: Figure 4—source data 1. [file elife-98372-fig4-data1.zip › Figure 4-data1/Figure_4-source_data_1_ Figure_4I_NOXA.jpg]

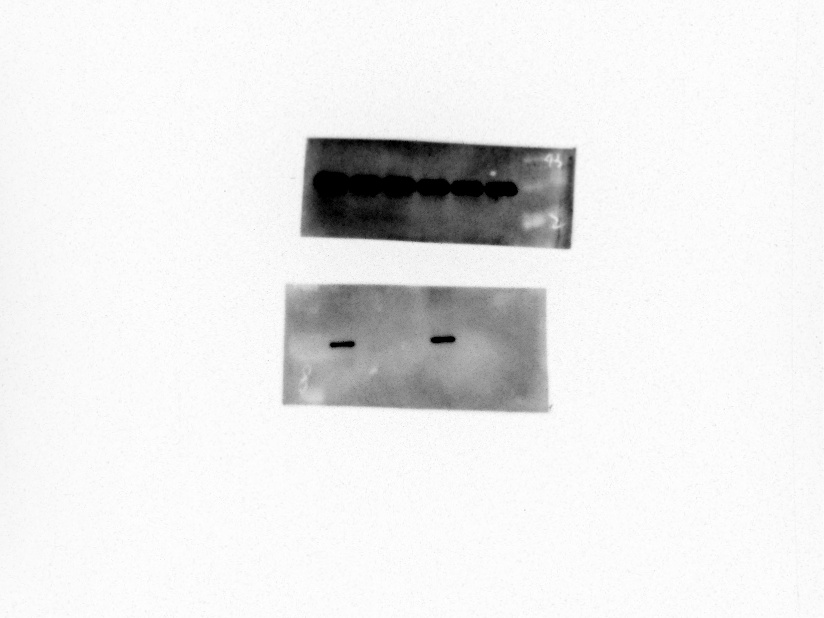

Supplement: Figure 4—source data 1. [file elife-98372-fig4-data1.zip › Figure 4-data1/Figure_4-source_data_1_ Figure_4I_WSB2.jpg]

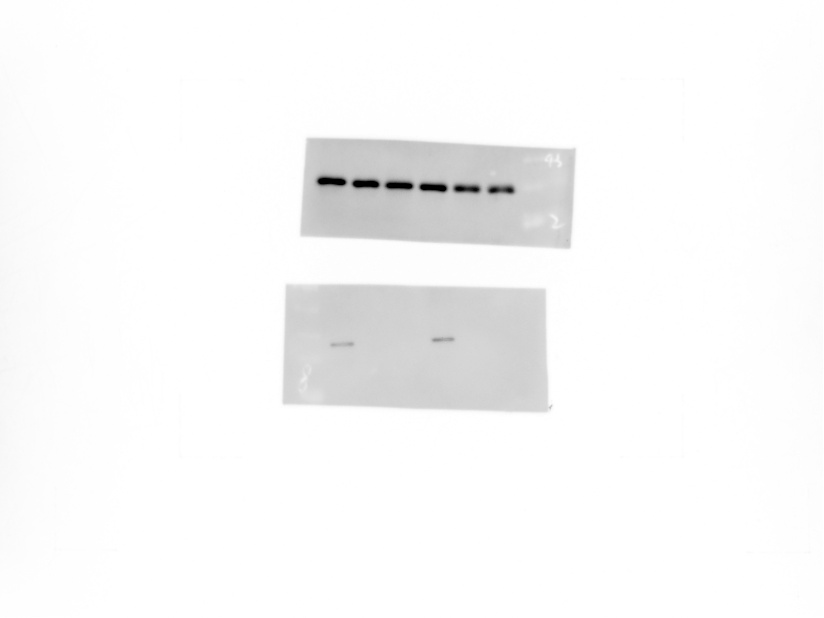

Supplement: Figure 4—source data 1. [file elife-98372-fig4-data1.zip › Figure 4-data1/Figure_4-source_data_1_ Figure_4K_CASP3.jpg]

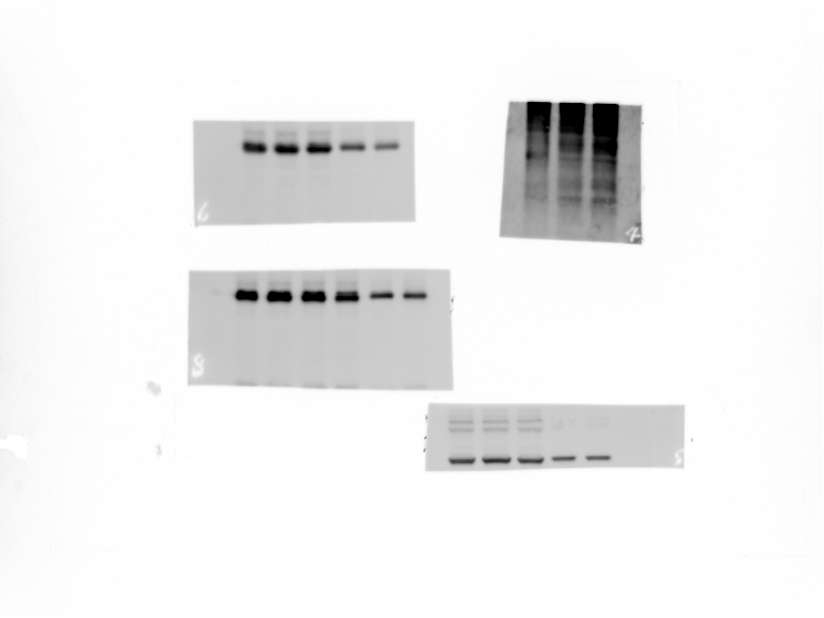

Supplement: Figure 4—source data 1. [file elife-98372-fig4-data1.zip › Figure 4-data1/Figure_4-source_data_1_ Figure_4K_CASP7.jpg]

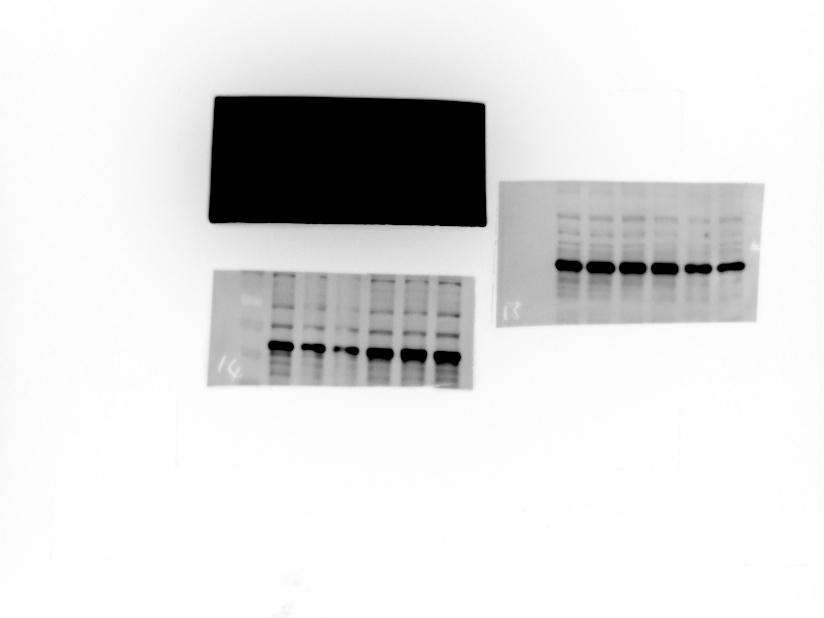

Supplement: Figure 4—source data 1. [file elife-98372-fig4-data1.zip › Figure 4-data1/Figure_4-source_data_1_ Figure_4K_CASP9.jpg]

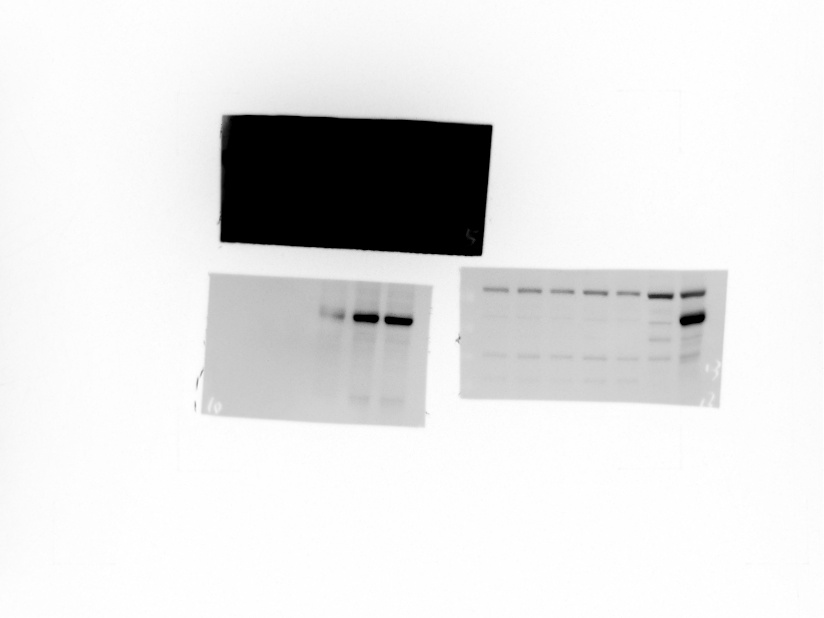

Supplement: Figure 4—source data 1. [file elife-98372-fig4-data1.zip › Figure 4-data1/Figure_4-source_data_1_ Figure_4K_cl-CASP3.jpg]

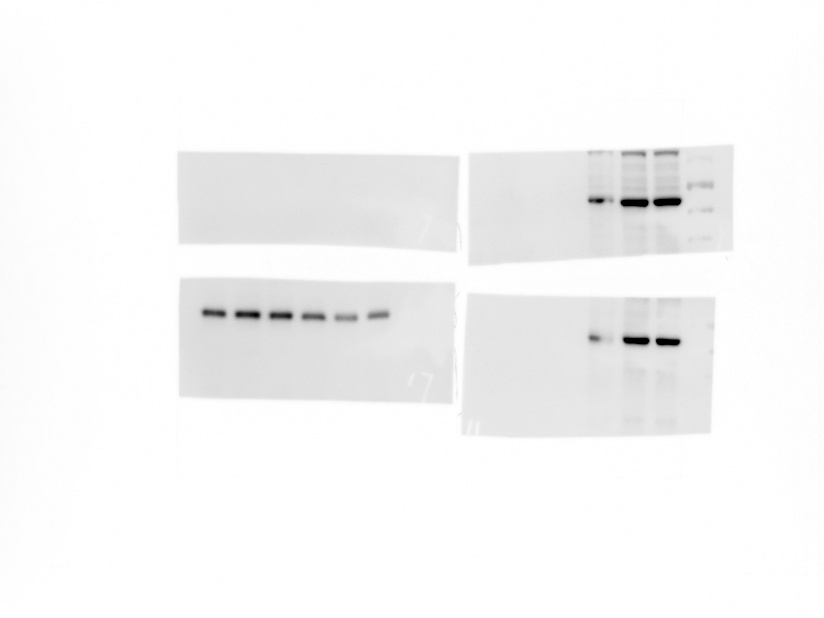

Supplement: Figure 4—source data 1. [file elife-98372-fig4-data1.zip › Figure 4-data1/Figure_4-source_data_1_ Figure_4K_cl-CASP7.jpg]

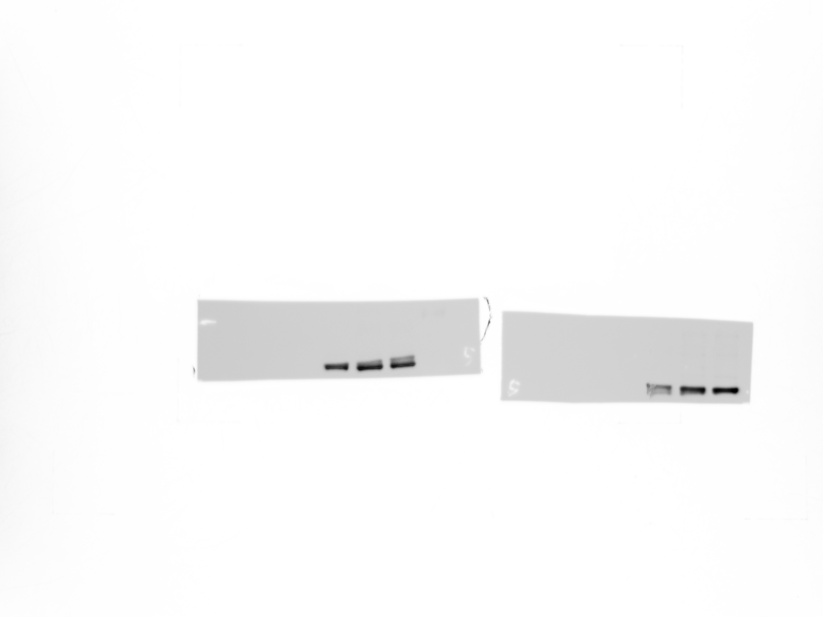

Supplement: Figure 4—source data 1. [file elife-98372-fig4-data1.zip › Figure 4-data1/Figure_4-source_data_1_ Figure_4K_cl-CASP9.jpg]

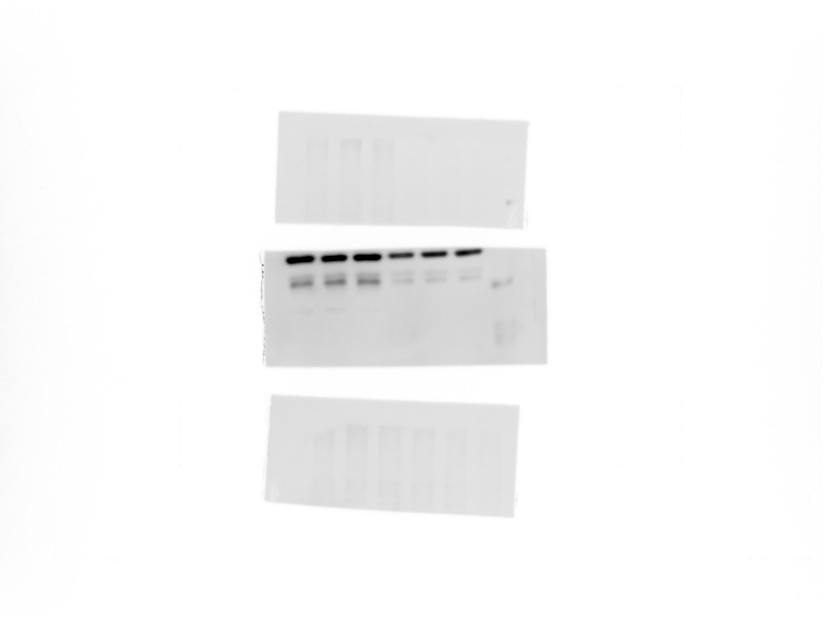

Supplement: Figure 4—source data 1. [file elife-98372-fig4-data1.zip › Figure 4-data1/Figure_4-source_data_1_ Figure_4K_MCL-1.jpg]

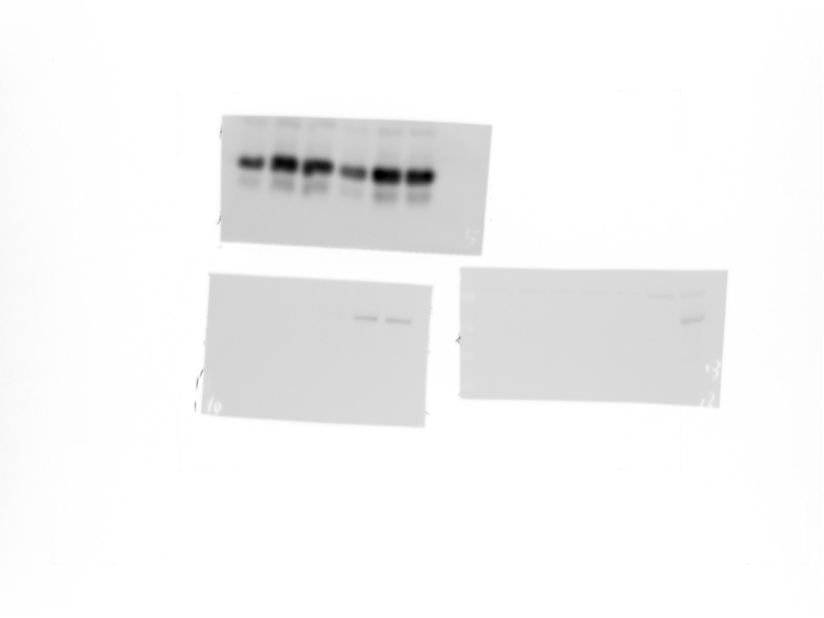

Supplement: Figure 4—source data 1. [file elife-98372-fig4-data1.zip › Figure 4-data1/Figure_4-source_data_1_ Figure_4K_NOXA.jpg]

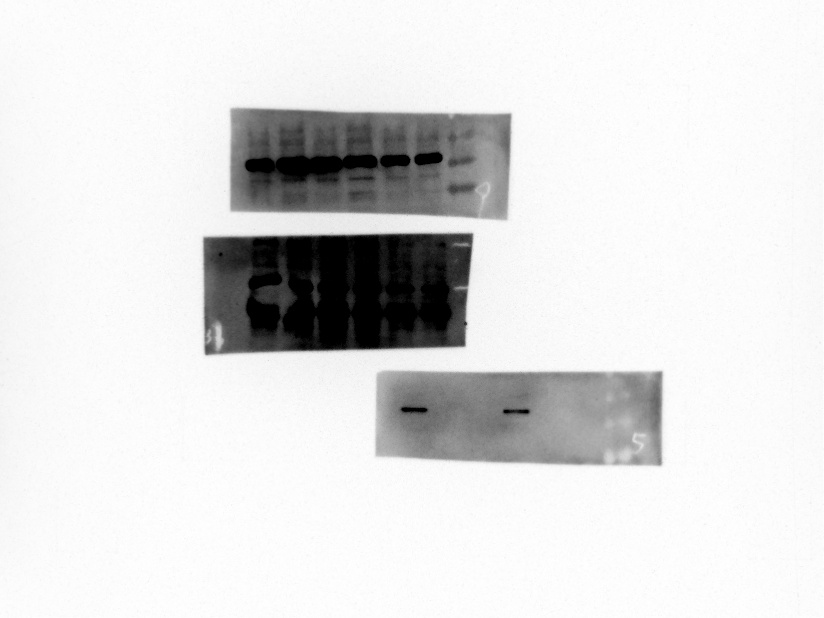

Supplement: Figure 4—source data 1. [file elife-98372-fig4-data1.zip › Figure 4-data1/Figure_4-source_data_1_ Figure_4K_WSB2.jpg]

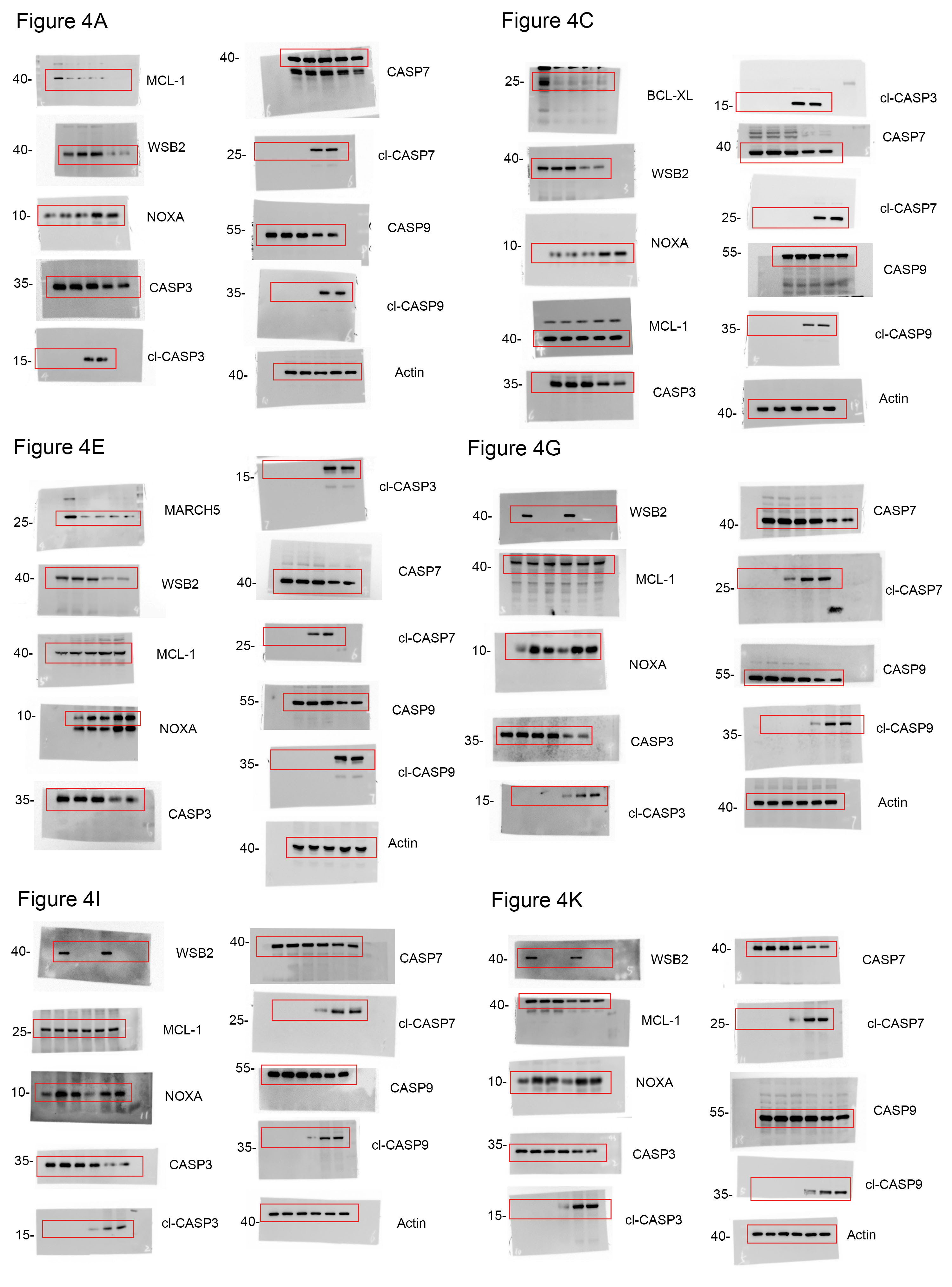

Supplement: Figure 4—source data 2. [file elife-98372-fig4-data2.zip › Figure 4-data2/Figure_4_data_2.jpg]

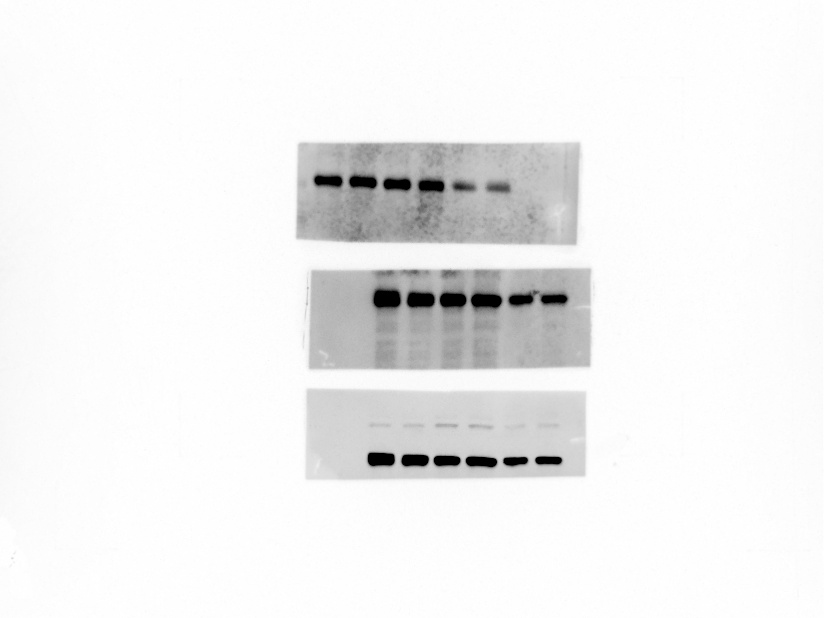

Supplement: Figure 4—figure supplement 1—source data 1. [file elife-98372-fig4-figsupp1-data1.zip › Figure 4-supplementary figure 1-data1/Figure_3-figure supplement_1_ source_data_1_ Figure_3A_CASP3.jpg]

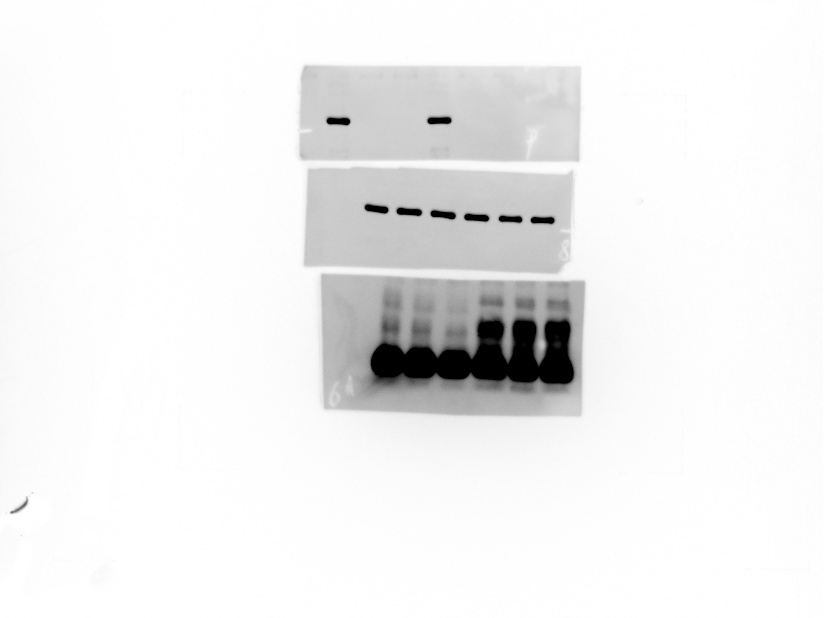

Supplement: Figure 4—figure supplement 1—source data 1. [file elife-98372-fig4-figsupp1-data1.zip › Figure 4-supplementary figure 1-data1/Figure_4-figure supplement_1_ source_data_1_ Figure_A_Actin.jpg]

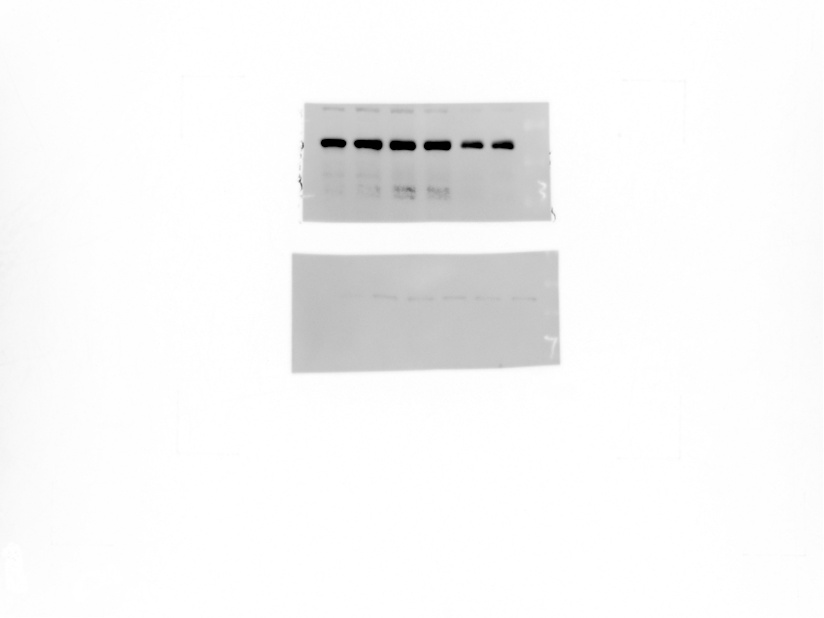

Supplement: Figure 4—figure supplement 1—source data 1. [file elife-98372-fig4-figsupp1-data1.zip › Figure 4-supplementary figure 1-data1/Figure_4-figure supplement_1_ source_data_1_ Figure_A_CASP7.jpg]

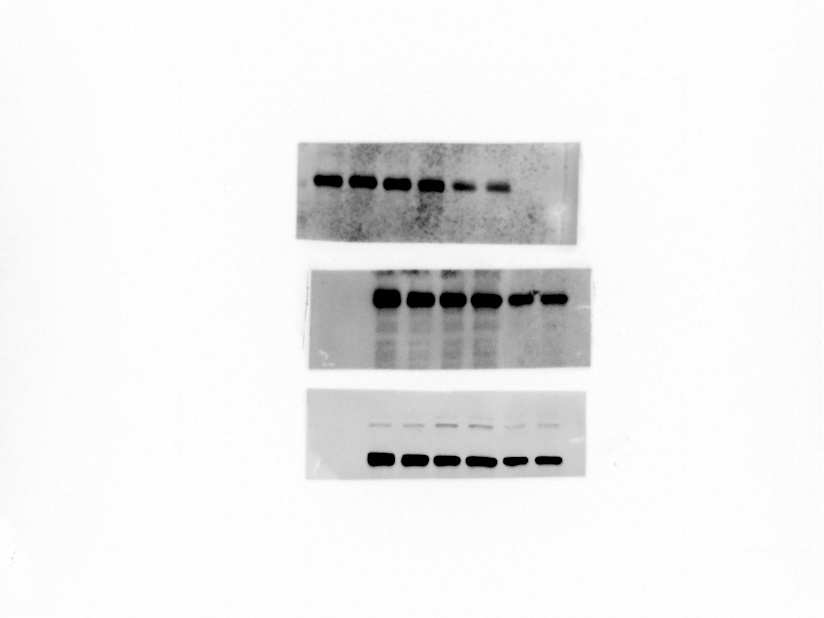

Supplement: Figure 4—figure supplement 1—source data 1. [file elife-98372-fig4-figsupp1-data1.zip › Figure 4-supplementary figure 1-data1/Figure_4-figure supplement_1_ source_data_1_ Figure_A_CASP9.jpg]

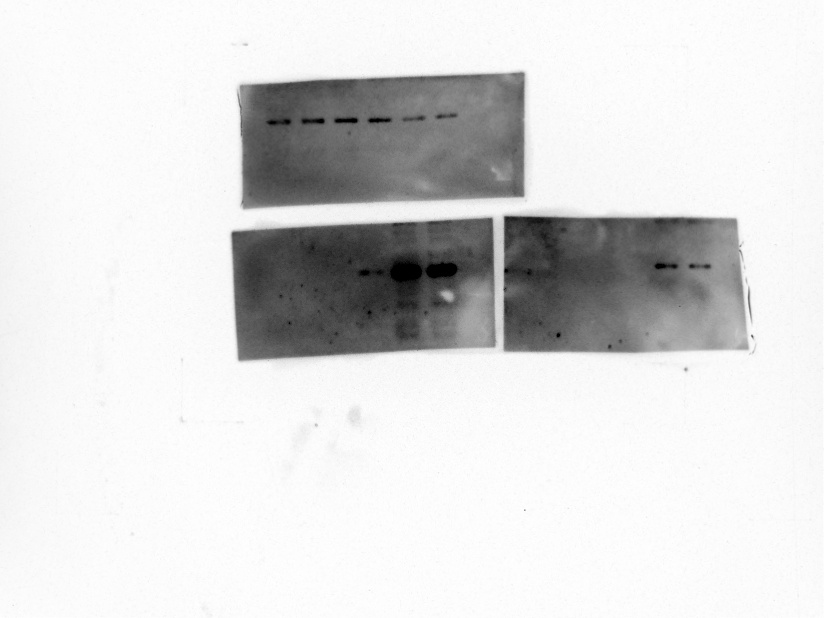

Supplement: Figure 4—figure supplement 1—source data 1. [file elife-98372-fig4-figsupp1-data1.zip › Figure 4-supplementary figure 1-data1/Figure_4-figure supplement_1_ source_data_1_ Figure_A_cl-CASP3.jpg]

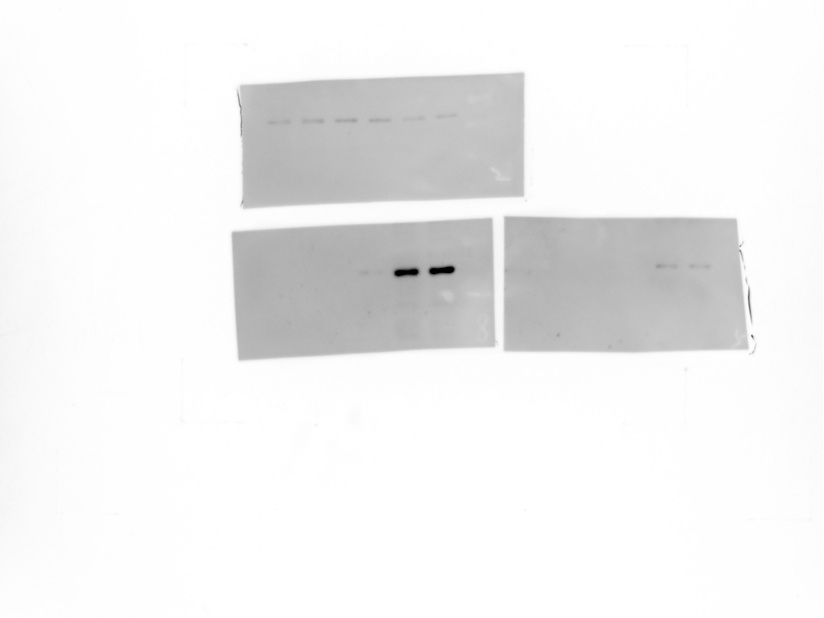

Supplement: Figure 4—figure supplement 1—source data 1. [file elife-98372-fig4-figsupp1-data1.zip › Figure 4-supplementary figure 1-data1/Figure_4-figure supplement_1_ source_data_1_ Figure_A_cl-CASP7.jpg]

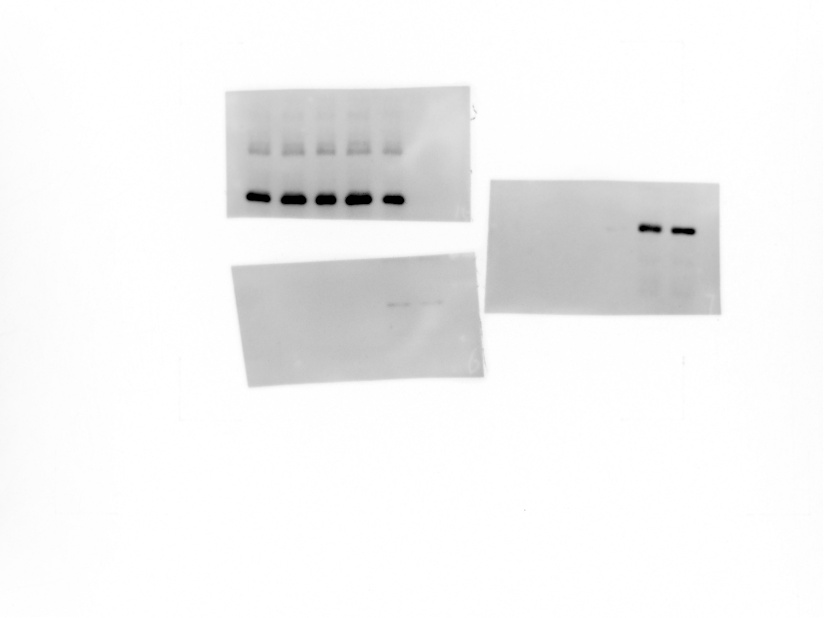

Supplement: Figure 4—figure supplement 1—source data 1. [file elife-98372-fig4-figsupp1-data1.zip › Figure 4-supplementary figure 1-data1/Figure_4-figure supplement_1_ source_data_1_ Figure_A_cl-CASP9.jpg]

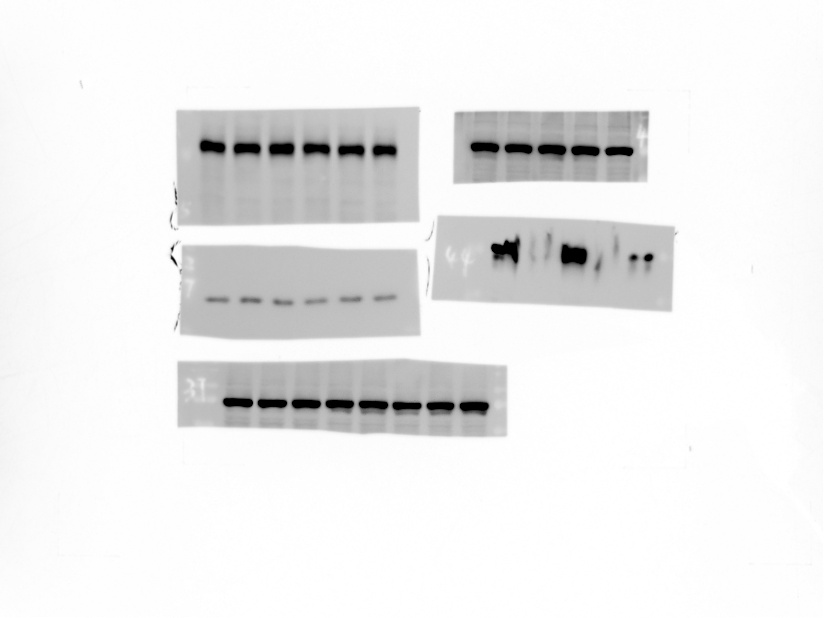

Supplement: Figure 4—figure supplement 1—source data 1. [file elife-98372-fig4-figsupp1-data1.zip › Figure 4-supplementary figure 1-data1/Figure_4-figure supplement_1_ source_data_1_ Figure_A_MCL-1.jpg]

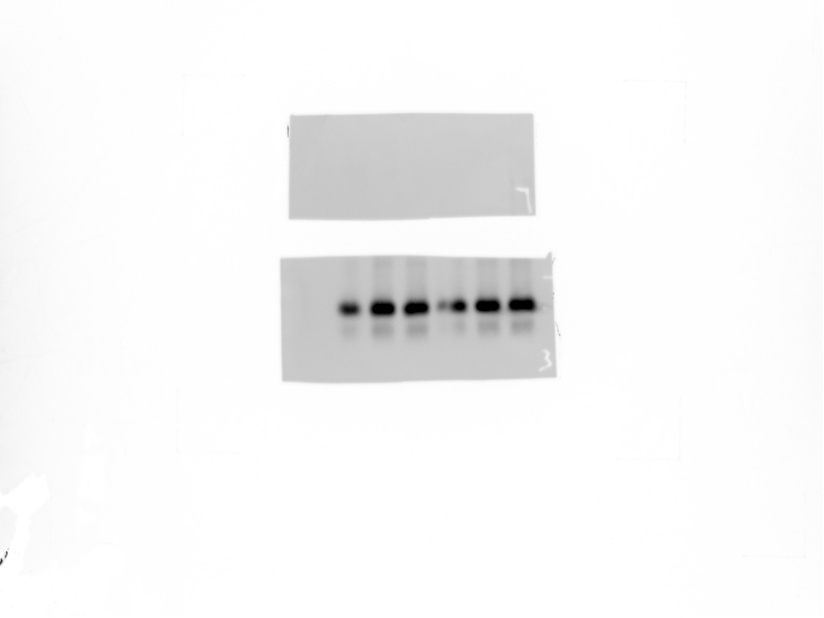

Supplement: Figure 4—figure supplement 1—source data 1. [file elife-98372-fig4-figsupp1-data1.zip › Figure 4-supplementary figure 1-data1/Figure_4-figure supplement_1_ source_data_1_ Figure_A_NOXA.jpg]

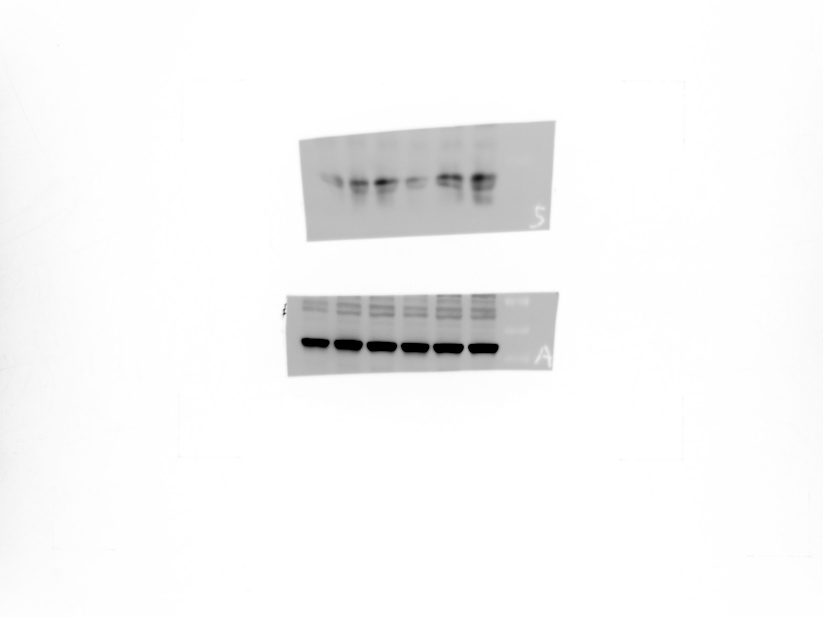

Supplement: Figure 4—figure supplement 1—source data 1. [file elife-98372-fig4-figsupp1-data1.zip › Figure 4-supplementary figure 1-data1/Figure_4-figure supplement_1_ source_data_1_ Figure_C_Actin.jpg]

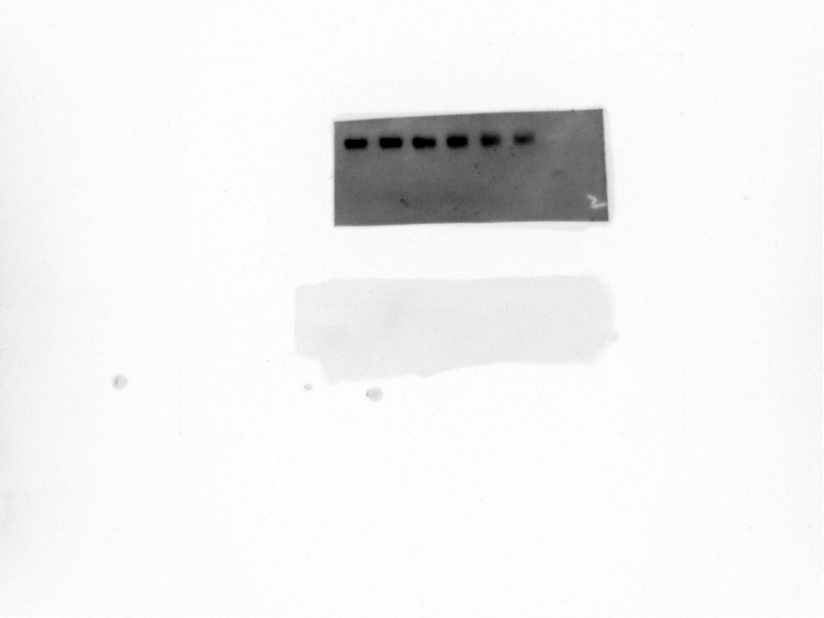

Supplement: Figure 4—figure supplement 1—source data 1. [file elife-98372-fig4-figsupp1-data1.zip › Figure 4-supplementary figure 1-data1/Figure_4-figure supplement_1_ source_data_1_ Figure_C_CASP3.jpg]

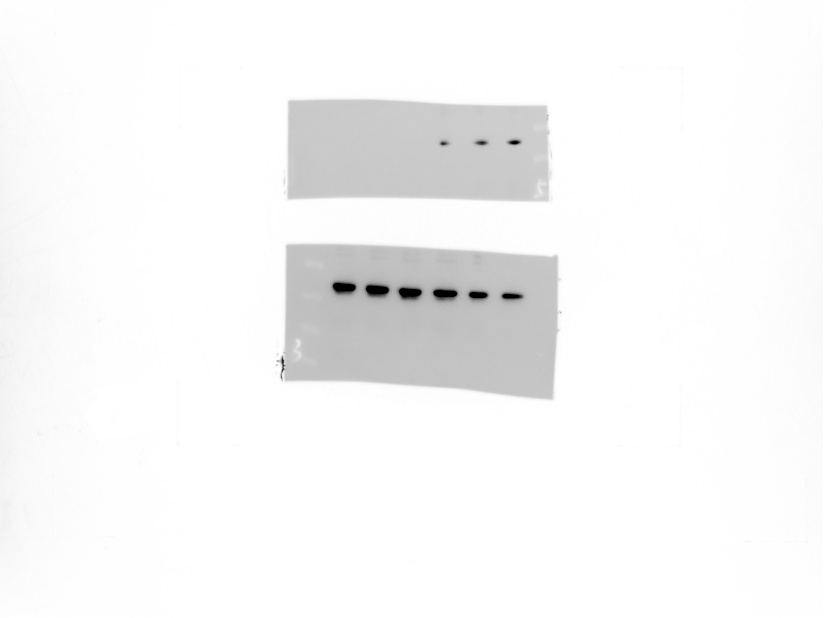

Supplement: Figure 4—figure supplement 1—source data 1. [file elife-98372-fig4-figsupp1-data1.zip › Figure 4-supplementary figure 1-data1/Figure_4-figure supplement_1_ source_data_1_ Figure_C_CASP7.jpg]

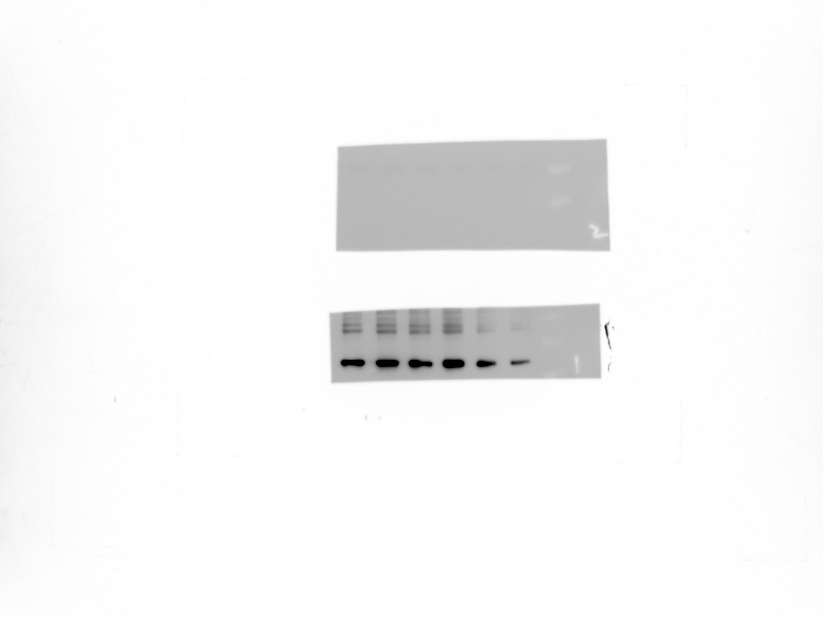

Supplement: Figure 4—figure supplement 1—source data 1. [file elife-98372-fig4-figsupp1-data1.zip › Figure 4-supplementary figure 1-data1/Figure_4-figure supplement_1_ source_data_1_ Figure_C_CASP9.jpg]

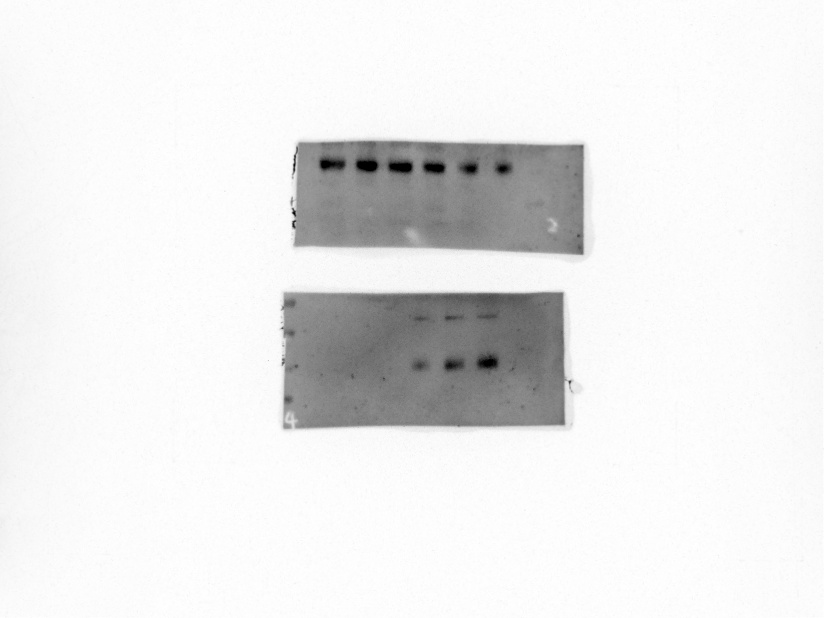

Supplement: Figure 4—figure supplement 1—source data 1. [file elife-98372-fig4-figsupp1-data1.zip › Figure 4-supplementary figure 1-data1/Figure_4-figure supplement_1_ source_data_1_ Figure_C_cl-CASP3.jpg]

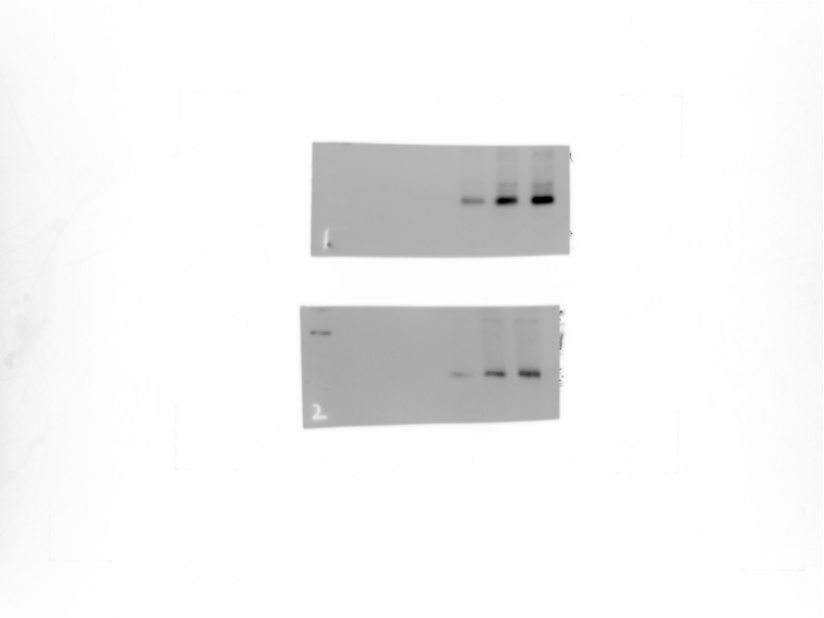

Supplement: Figure 4—figure supplement 1—source data 1. [file elife-98372-fig4-figsupp1-data1.zip › Figure 4-supplementary figure 1-data1/Figure_4-figure supplement_1_ source_data_1_ Figure_C_cl-CASP7.jpg]

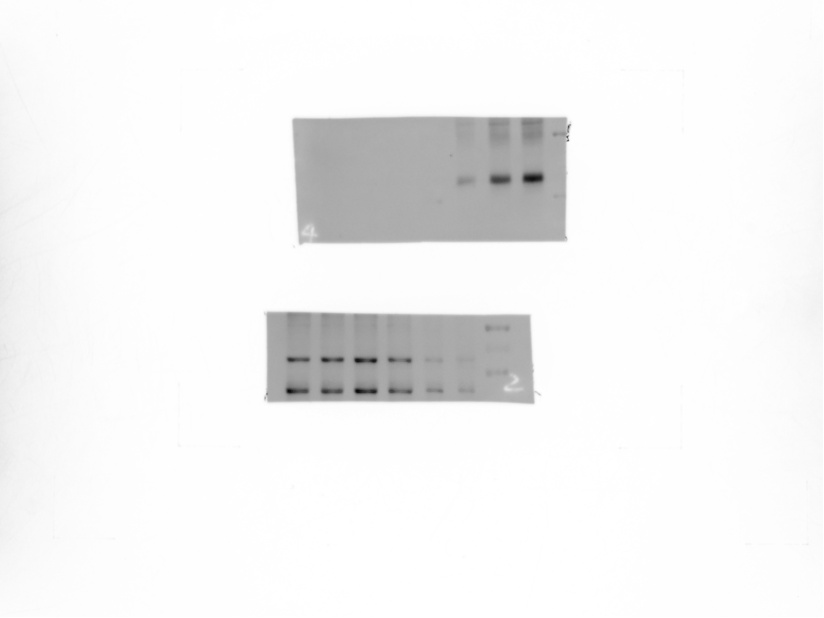

Supplement: Figure 4—figure supplement 1—source data 1. [file elife-98372-fig4-figsupp1-data1.zip › Figure 4-supplementary figure 1-data1/Figure_4-figure supplement_1_ source_data_1_ Figure_C_cl-CASP9.jpg]

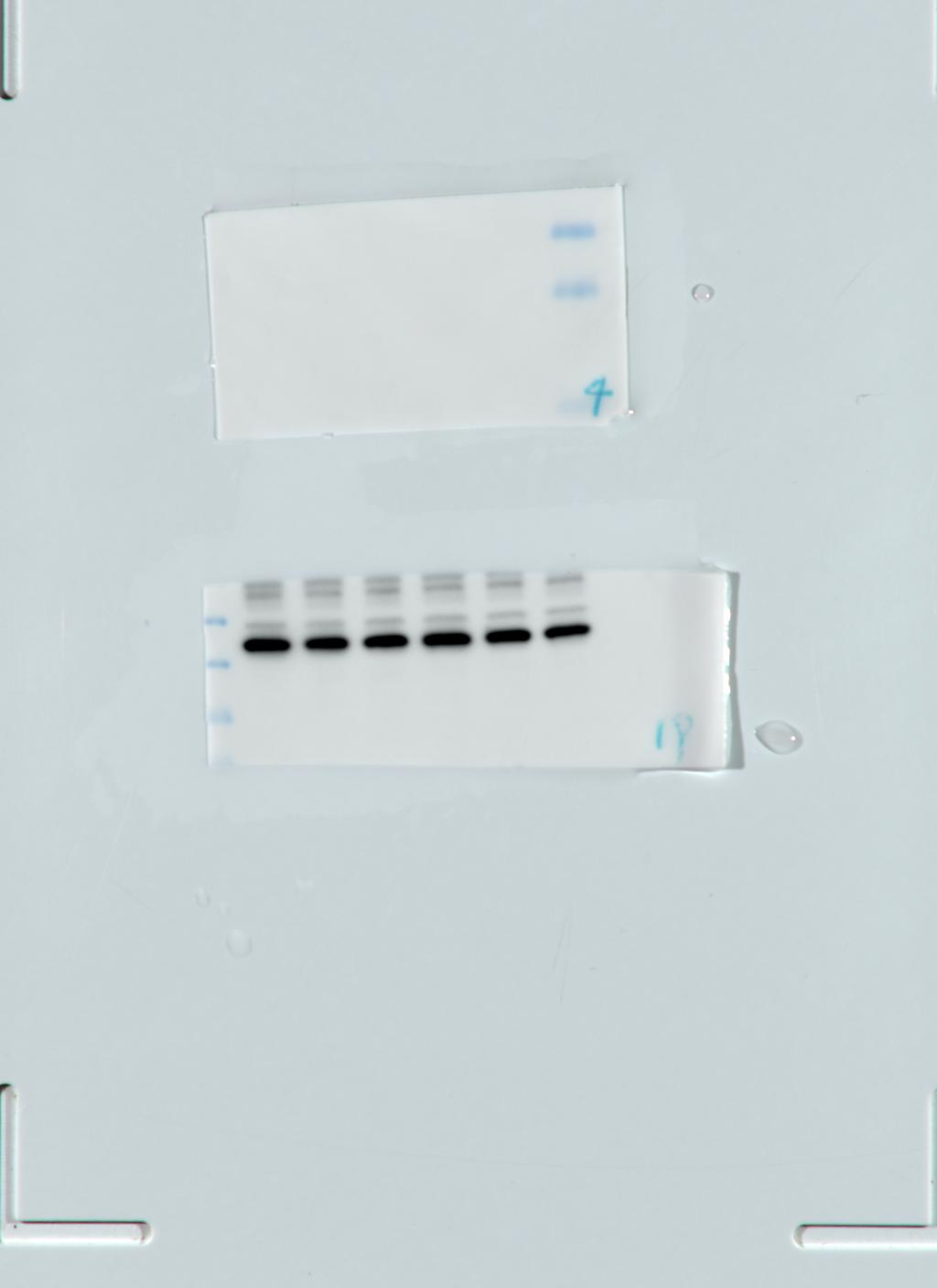

Supplement: Figure 4—figure supplement 1—source data 1. [file elife-98372-fig4-figsupp1-data1.zip › Figure 4-supplementary figure 1-data1/Figure_4-figure supplement_1_ source_data_1_ Figure_C_MCL1.jpg]

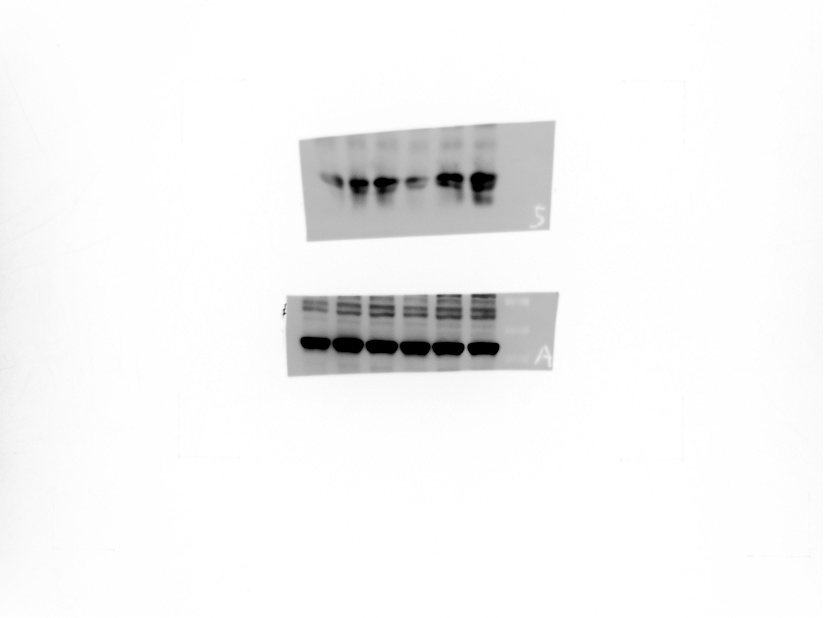

Supplement: Figure 4—figure supplement 1—source data 1. [file elife-98372-fig4-figsupp1-data1.zip › Figure 4-supplementary figure 1-data1/Figure_4-figure supplement_1_ source_data_1_ Figure_C_NOXA.jpg]

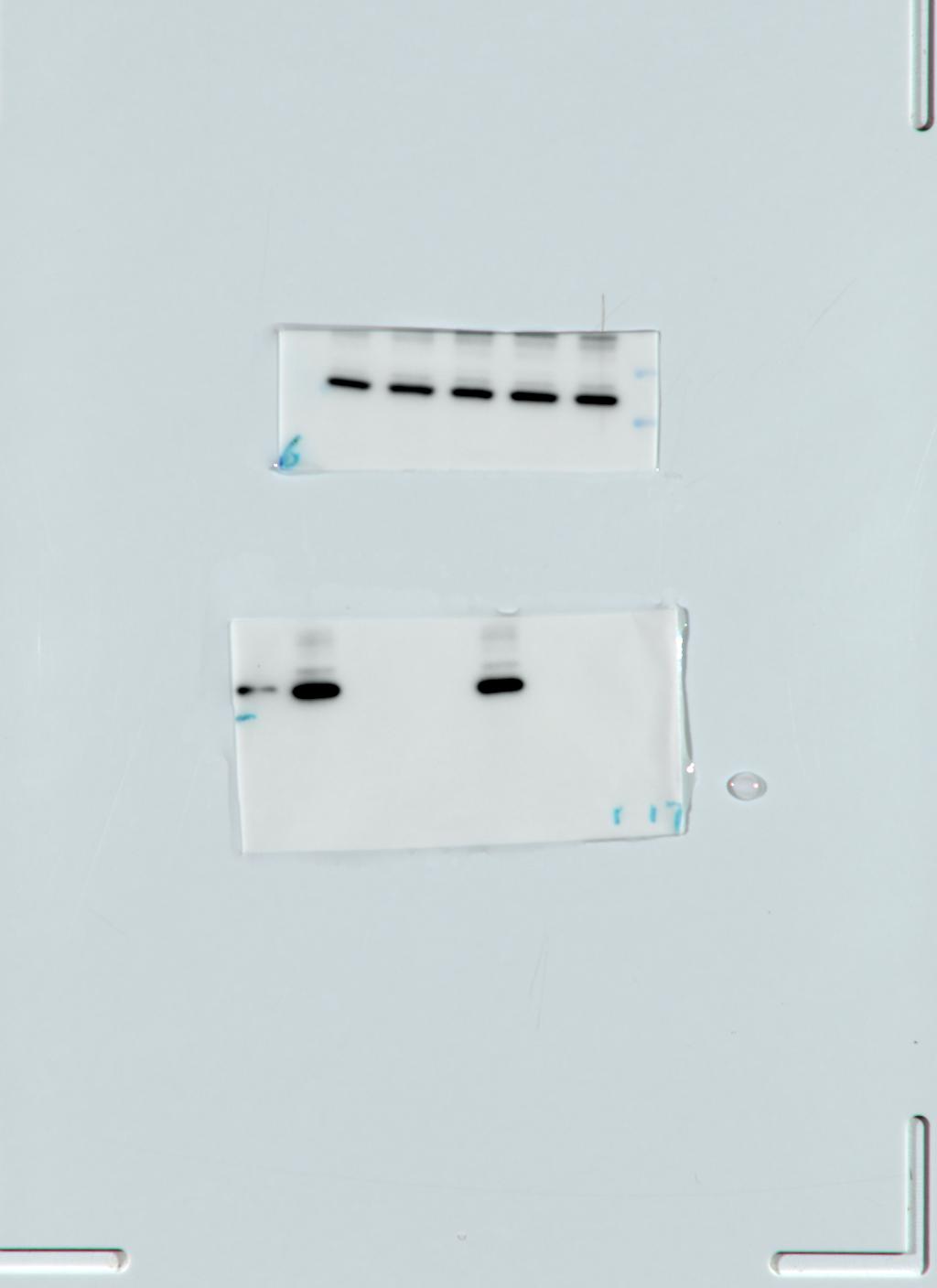

Supplement: Figure 4—figure supplement 1—source data 1. [file elife-98372-fig4-figsupp1-data1.zip › Figure 4-supplementary figure 1-data1/Figure_4-figure supplement_1_ source_data_1_ Figure_C_WSB2.jpg]

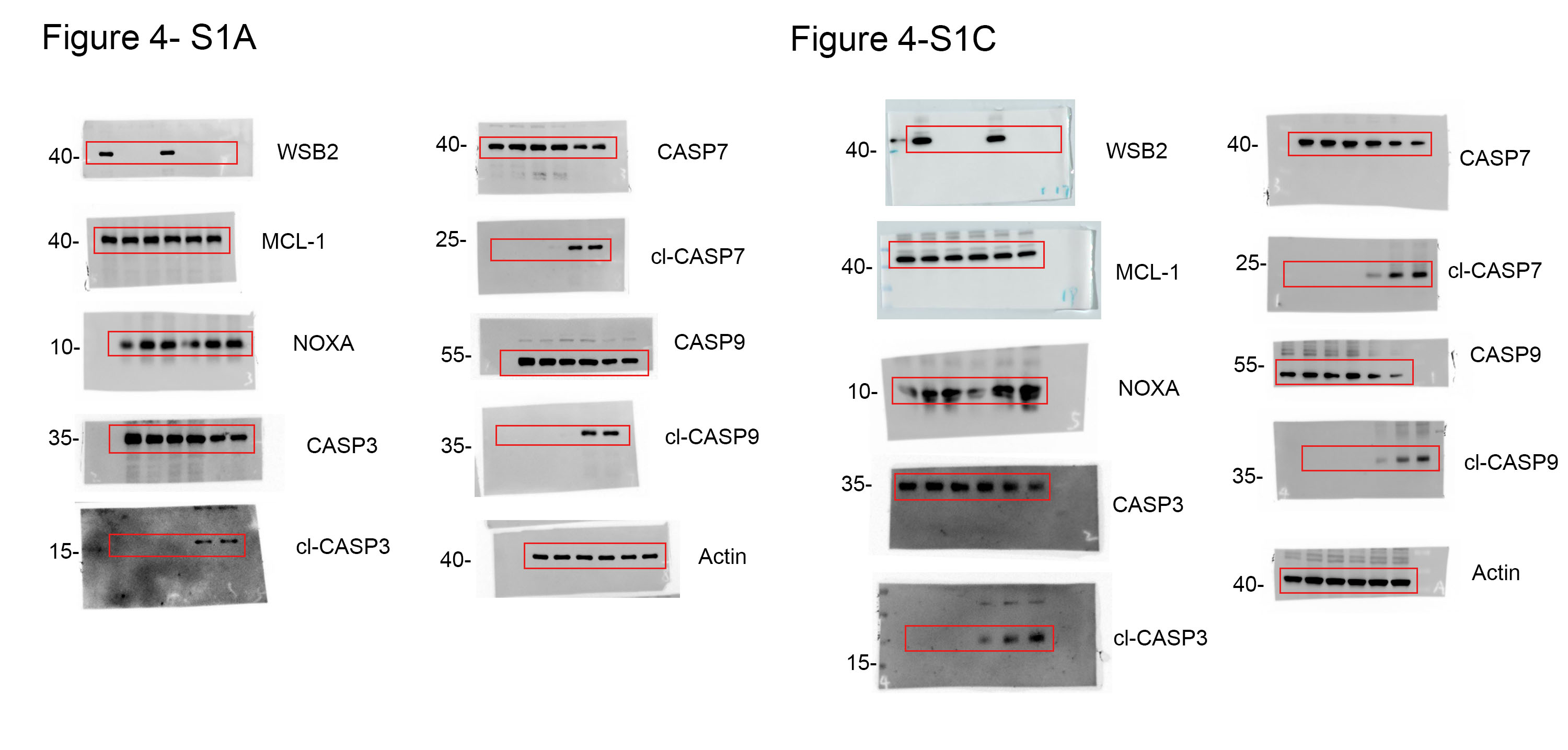

Supplement: Figure 4—figure supplement 1—source data 2. [file elife-98372-fig4-figsupp1-data2.zip › Figure 4-supplementary figure 1-data2/Figure_4-figure supplement_1_ data_2.jpg]

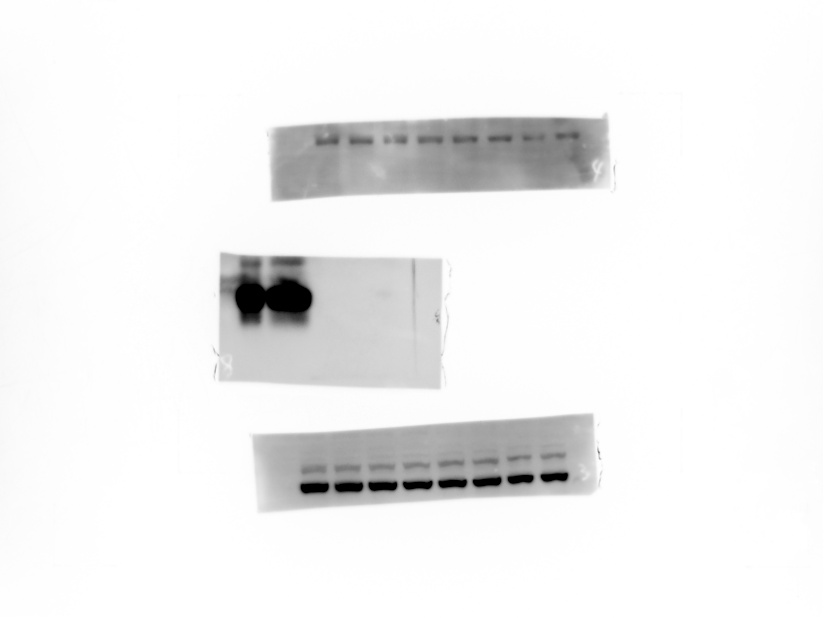

Supplement: Figure 5—source data 1. [file elife-98372-fig5-data1.zip › Figure 5-data1/Figure_5-source_data_1_ Figure_5A_Actin.jpg]

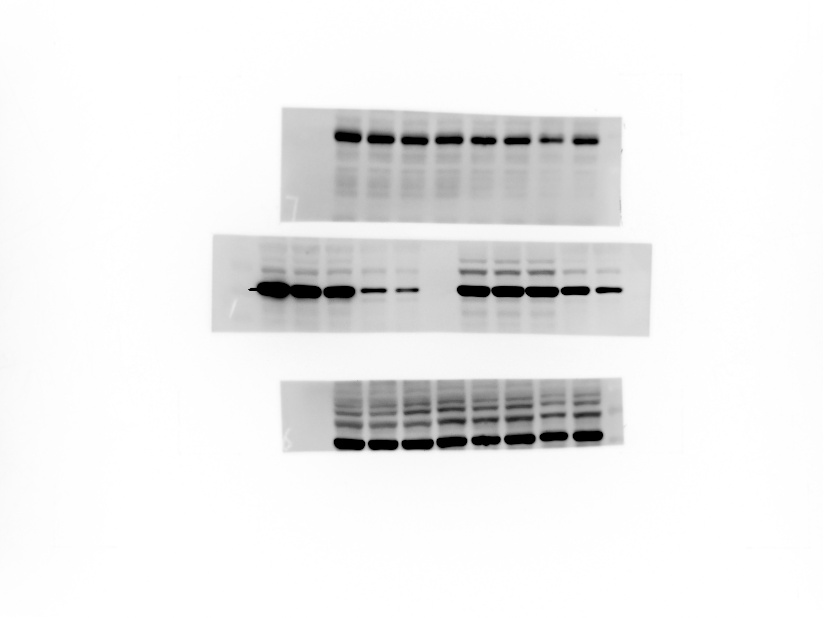

Supplement: Figure 5—source data 1. [file elife-98372-fig5-data1.zip › Figure 5-data1/Figure_5-source_data_1_ Figure_5A_CASP3.jpg]

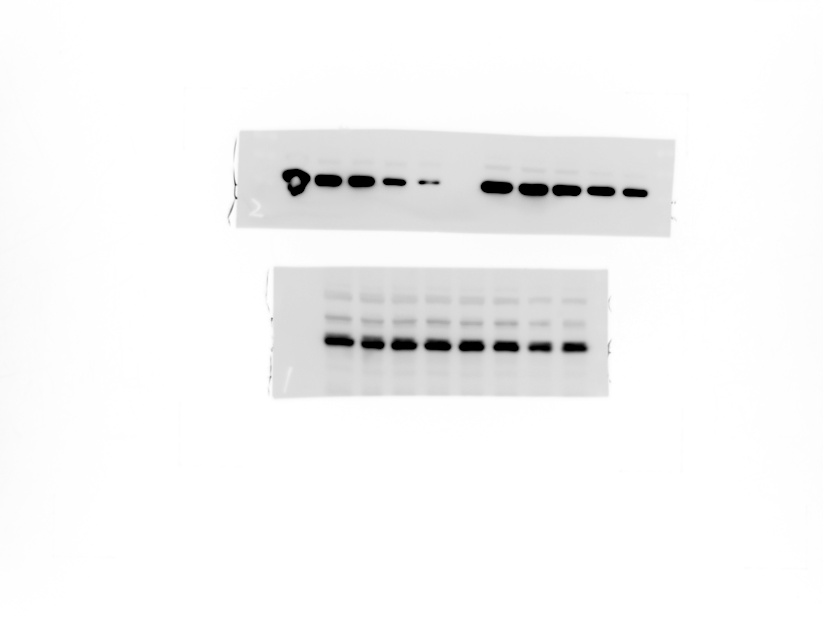

Supplement: Figure 5—source data 1. [file elife-98372-fig5-data1.zip › Figure 5-data1/Figure_5-source_data_1_ Figure_5A_CASP7.jpg]

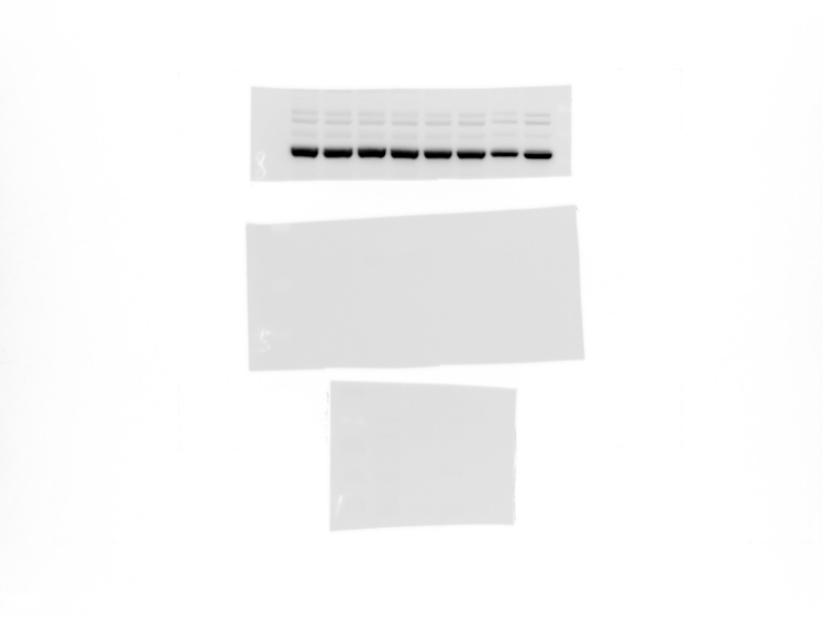

Supplement: Figure 5—source data 1. [file elife-98372-fig5-data1.zip › Figure 5-data1/Figure_5-source_data_1_ Figure_5A_CASP9.jpg]

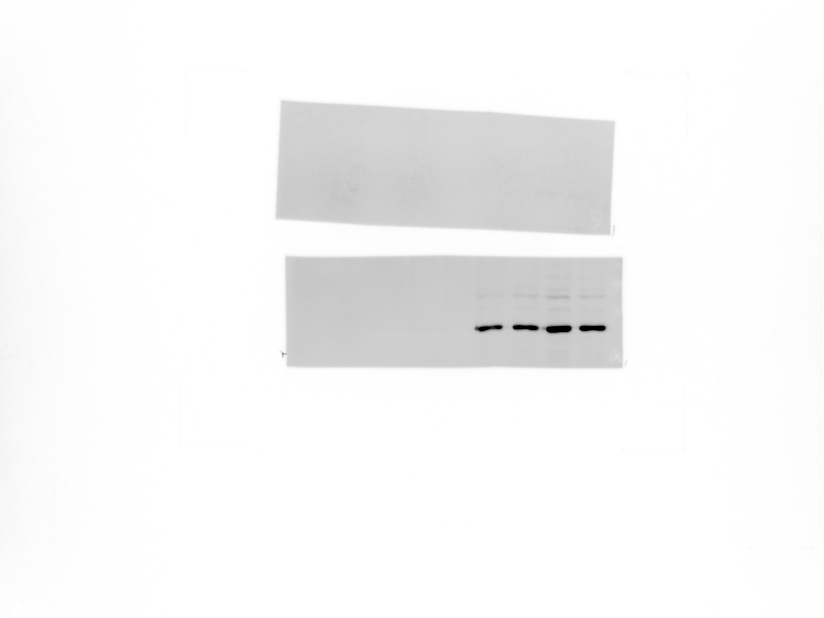

Supplement: Figure 5—source data 1. [file elife-98372-fig5-data1.zip › Figure 5-data1/Figure_5-source_data_1_ Figure_5A_cl-CASP3.jpg]

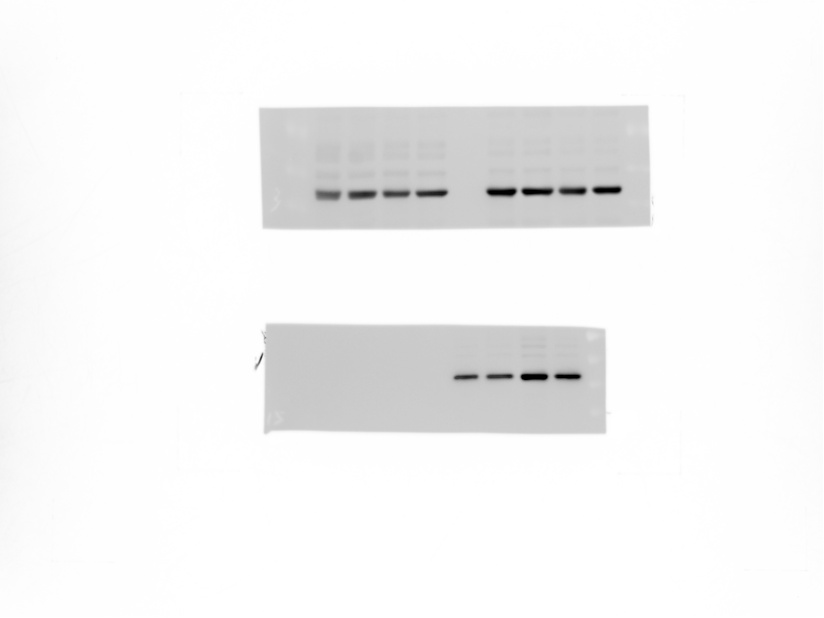

Supplement: Figure 5—source data 1. [file elife-98372-fig5-data1.zip › Figure 5-data1/Figure_5-source_data_1_ Figure_5A_cl-CASP7.jpg]

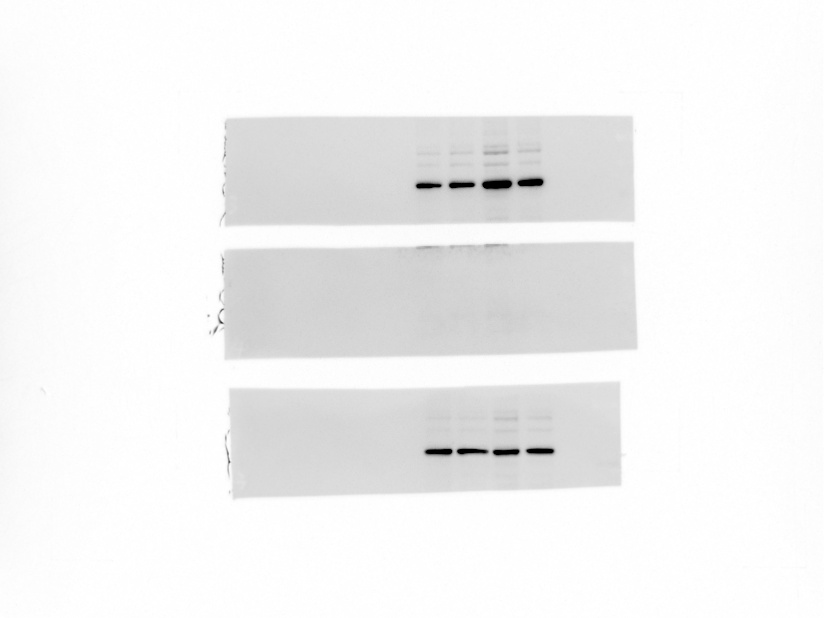

Supplement: Figure 5—source data 1. [file elife-98372-fig5-data1.zip › Figure 5-data1/Figure_5-source_data_1_ Figure_5A_cl-CASP9.jpg]

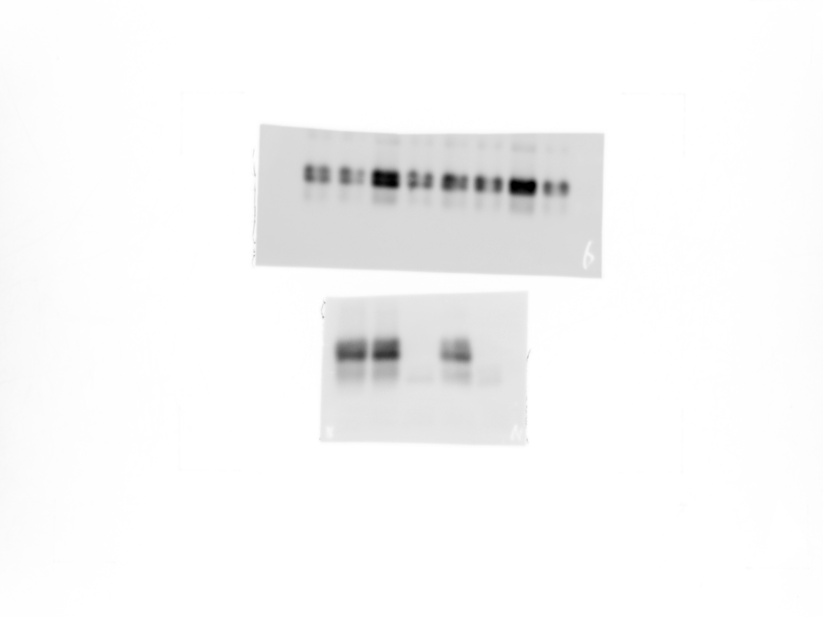

Supplement: Figure 5—source data 1. [file elife-98372-fig5-data1.zip › Figure 5-data1/Figure_5-source_data_1_ Figure_5A_NOXA.jpg]

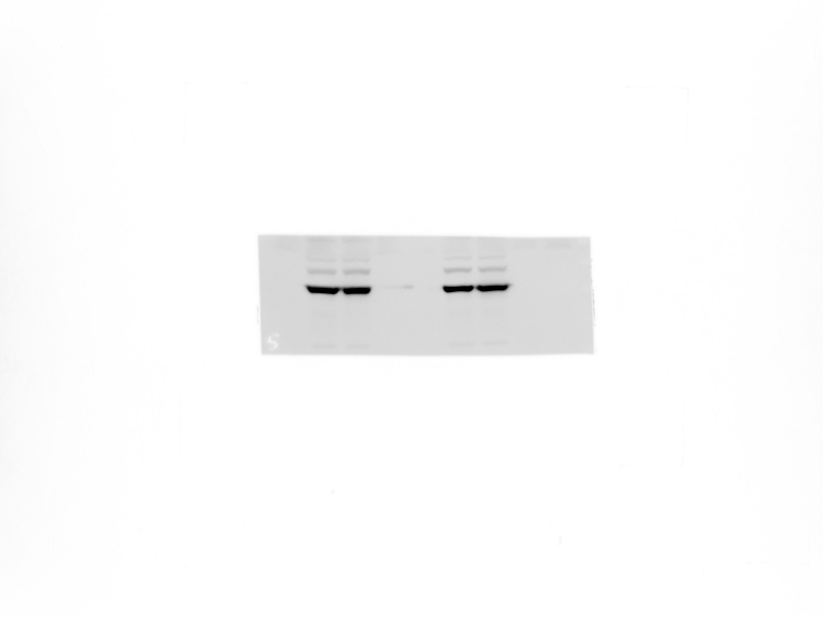

Supplement: Figure 5—source data 1. [file elife-98372-fig5-data1.zip › Figure 5-data1/Figure_5-source_data_1_ Figure_5A_WSB2.jpg]

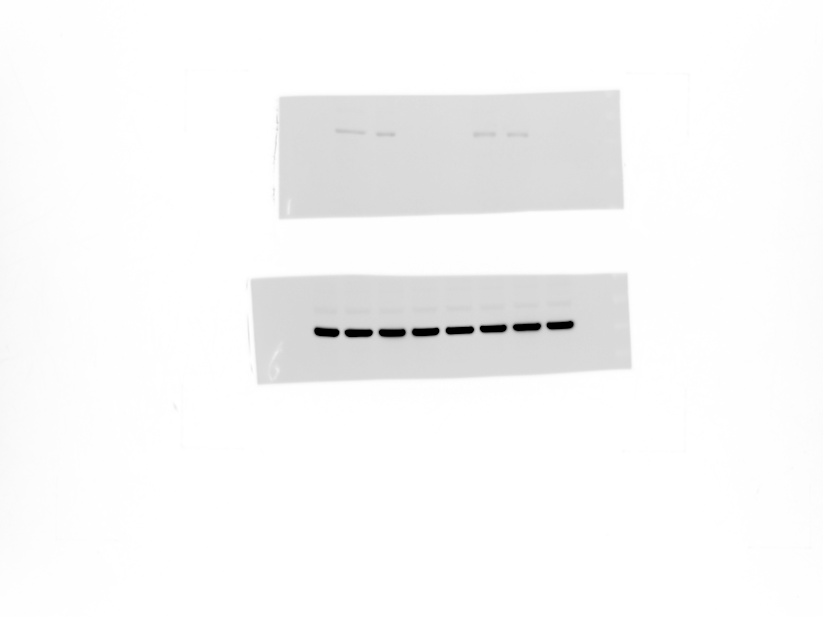

Supplement: Figure 5—source data 1. [file elife-98372-fig5-data1.zip › Figure 5-data1/Figure_5-source_data_1_ Figure_5C_Actin.jpg]

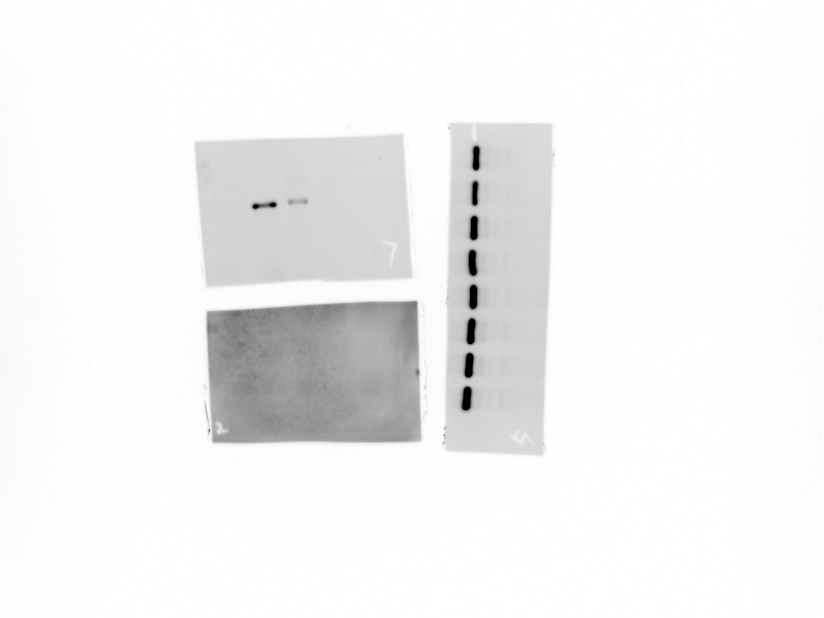

Supplement: Figure 5—source data 1. [file elife-98372-fig5-data1.zip › Figure 5-data1/Figure_5-source_data_1_ Figure_5C_CASP3.jpg]

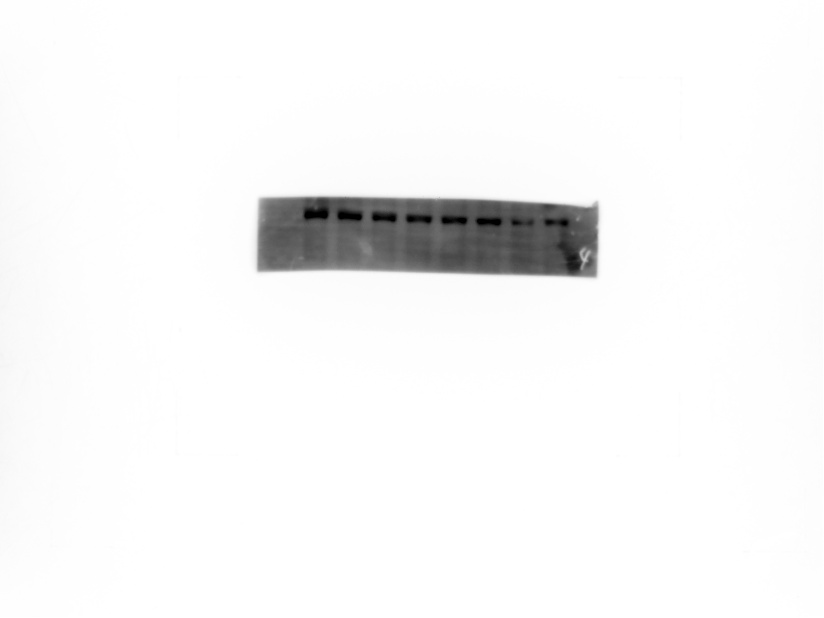

Supplement: Figure 5—source data 1. [file elife-98372-fig5-data1.zip › Figure 5-data1/Figure_5-source_data_1_ Figure_5C_CASP7.jpg]

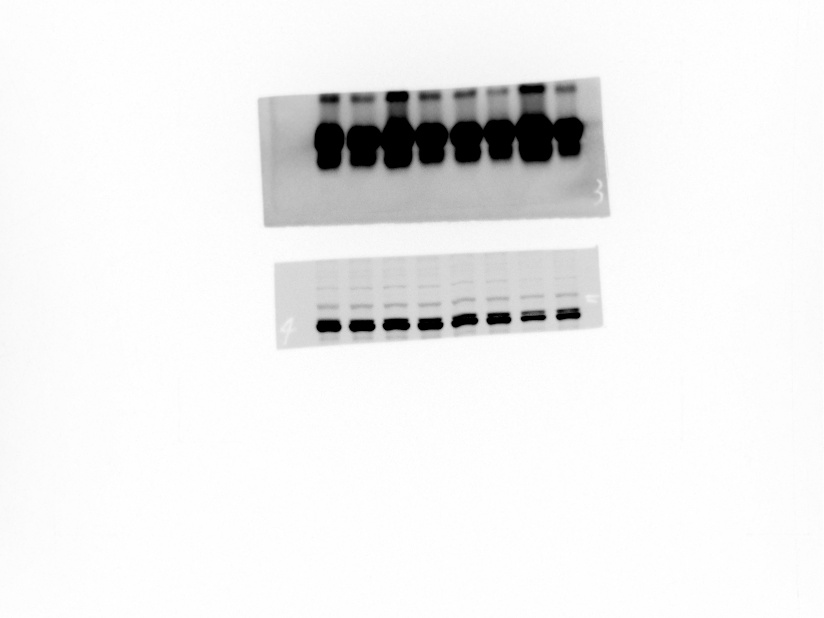

Supplement: Figure 5—source data 1. [file elife-98372-fig5-data1.zip › Figure 5-data1/Figure_5-source_data_1_ Figure_5C_CASP9.jpg]

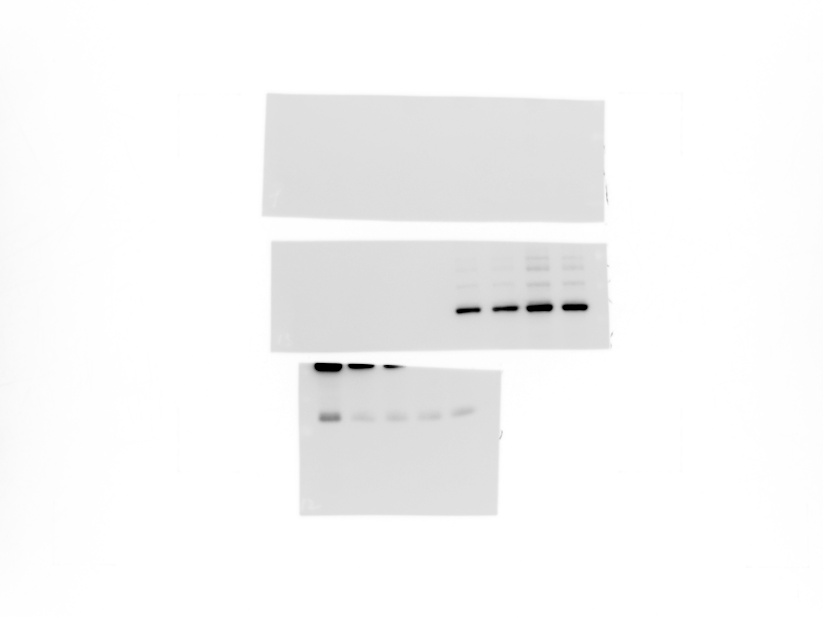

Supplement: Figure 5—source data 1. [file elife-98372-fig5-data1.zip › Figure 5-data1/Figure_5-source_data_1_ Figure_5C_cl-CASP3.jpg]

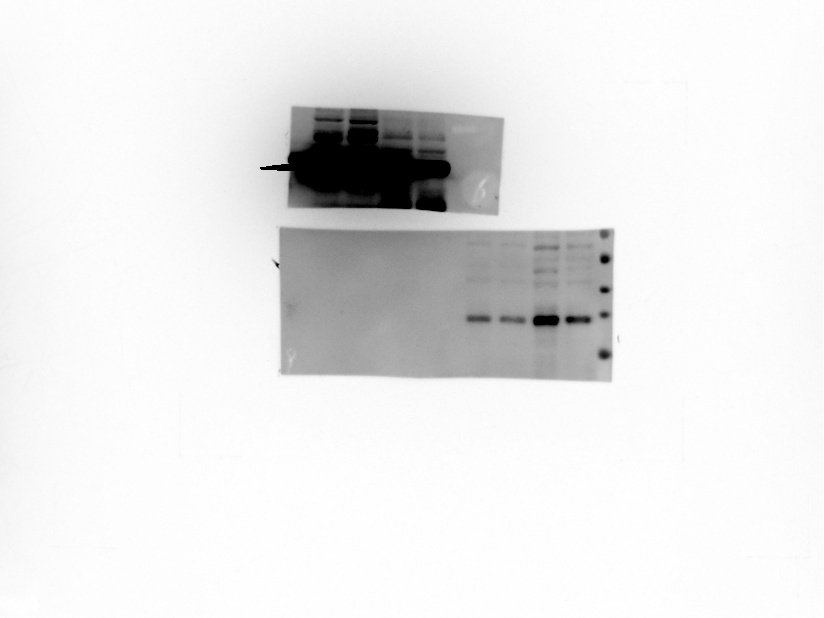

Supplement: Figure 5—source data 1. [file elife-98372-fig5-data1.zip › Figure 5-data1/Figure_5-source_data_1_ Figure_5C_cl-CASP7.jpg]

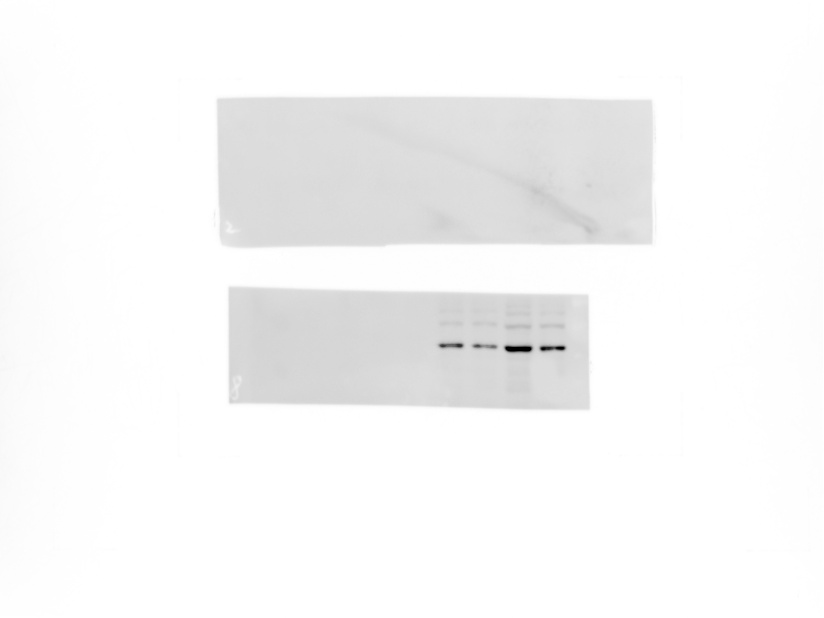

Supplement: Figure 5—source data 1. [file elife-98372-fig5-data1.zip › Figure 5-data1/Figure_5-source_data_1_ Figure_5C_cl-CASP9.jpg]

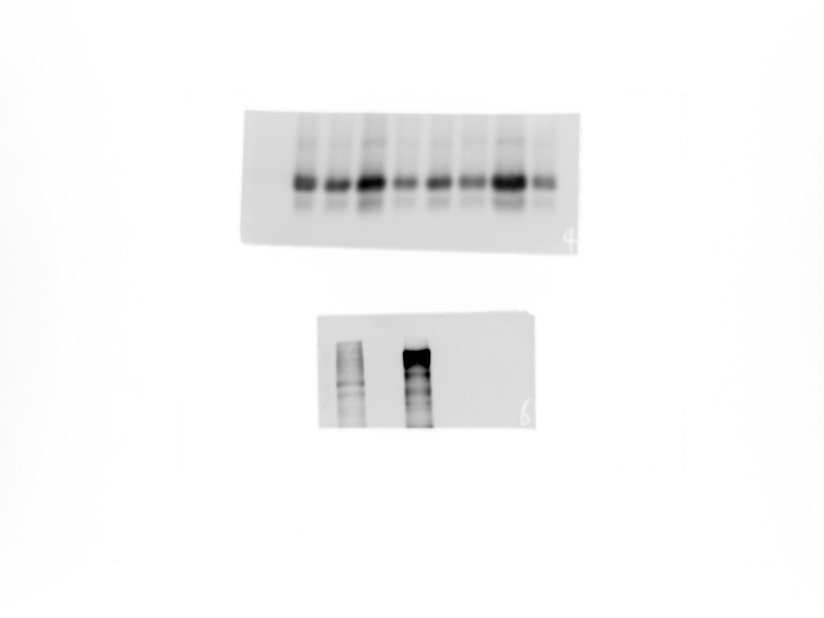

Supplement: Figure 5—source data 1. [file elife-98372-fig5-data1.zip › Figure 5-data1/Figure_5-source_data_1_ Figure_5C_NOXA.jpg]

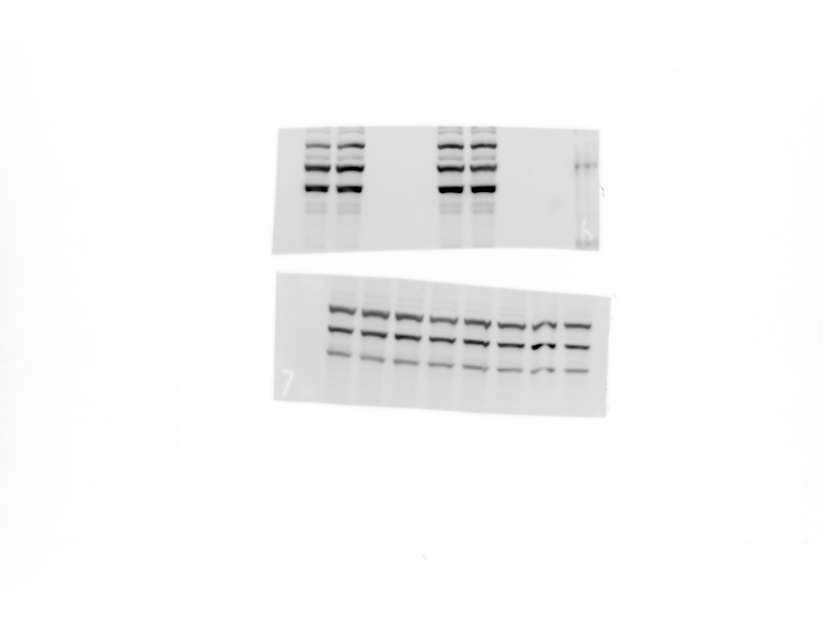

Supplement: Figure 5—source data 1. [file elife-98372-fig5-data1.zip › Figure 5-data1/Figure_5-source_data_1_ Figure_5C_WSB2.jpg]

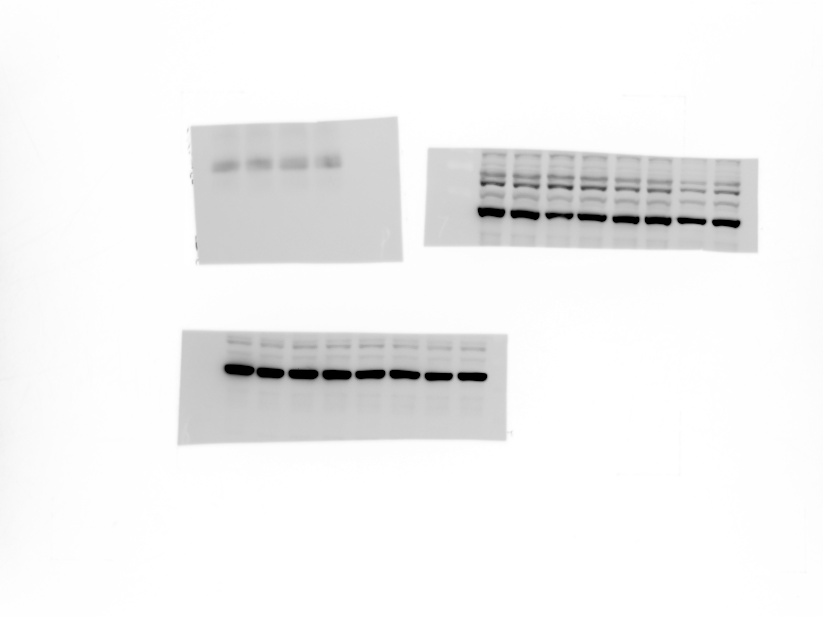

Supplement: Figure 5—source data 1. [file elife-98372-fig5-data1.zip › Figure 5-data1/Figure_5-source_data_1_ Figure_5E_Actin.jpg]

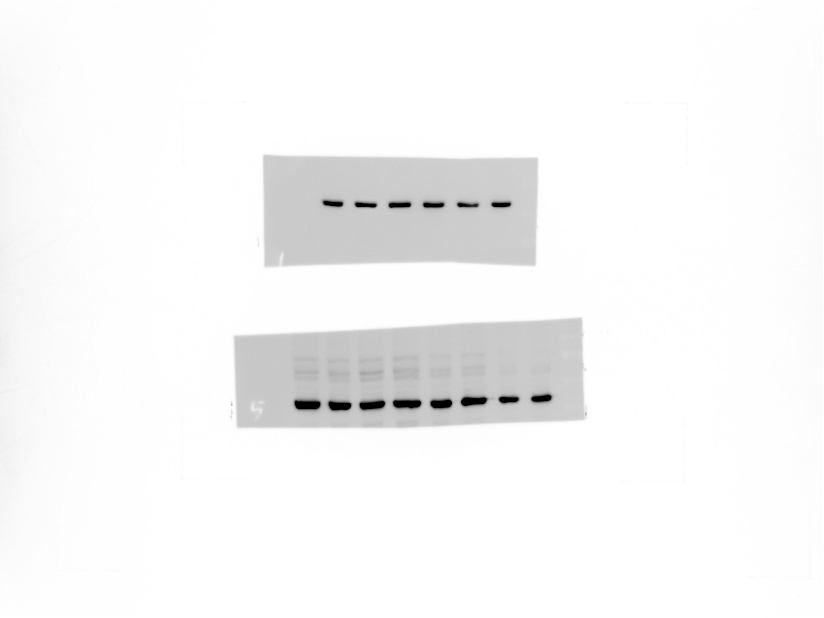

Supplement: Figure 5—source data 1. [file elife-98372-fig5-data1.zip › Figure 5-data1/Figure_5-source_data_1_ Figure_5E_CASP3.jpg]

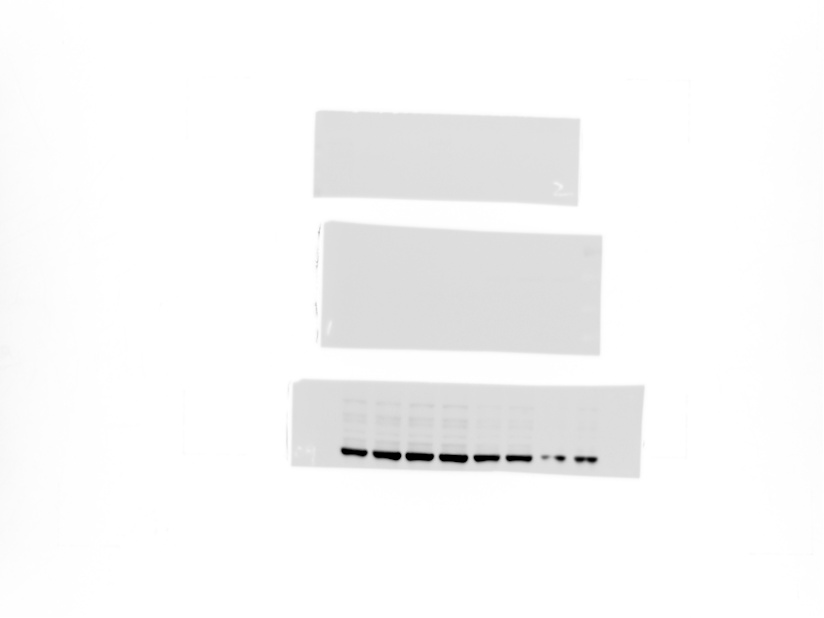

Supplement: Figure 5—source data 1. [file elife-98372-fig5-data1.zip › Figure 5-data1/Figure_5-source_data_1_ Figure_5E_CASP7.jpg]

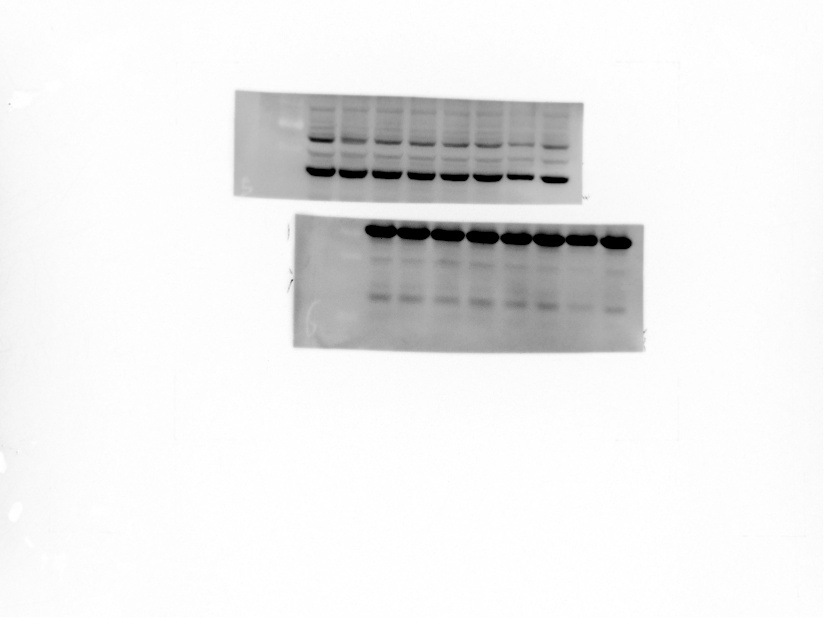

Supplement: Figure 5—source data 1. [file elife-98372-fig5-data1.zip › Figure 5-data1/Figure_5-source_data_1_ Figure_5E_CASP9.jpg]

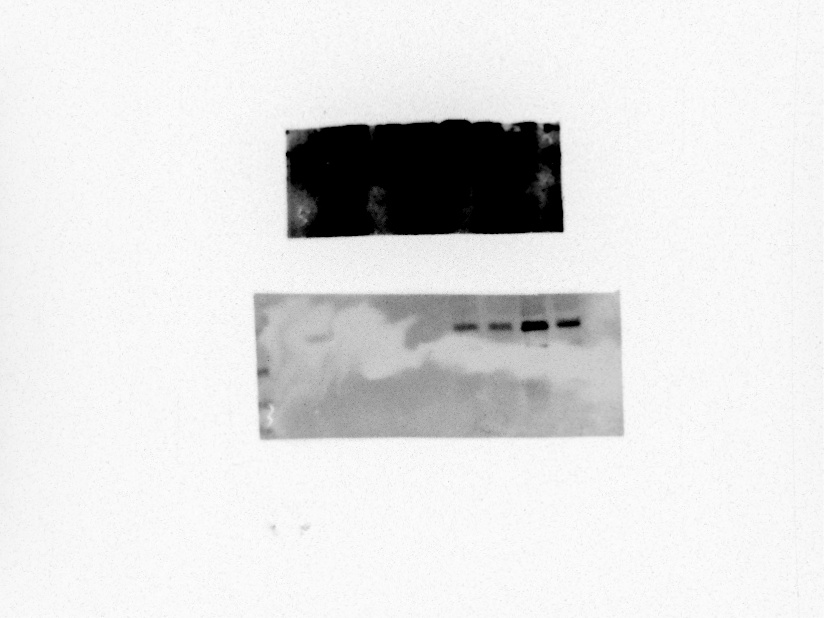

Supplement: Figure 5—source data 1. [file elife-98372-fig5-data1.zip › Figure 5-data1/Figure_5-source_data_1_ Figure_5E_cl-CASP3.jpg]

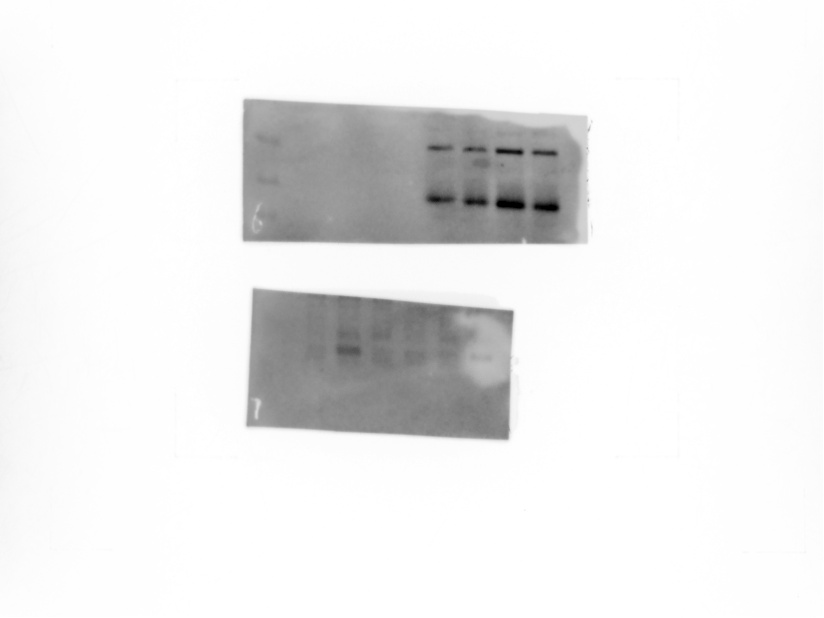

Supplement: Figure 5—source data 1. [file elife-98372-fig5-data1.zip › Figure 5-data1/Figure_5-source_data_1_ Figure_5E_cl-CASP7.jpg]

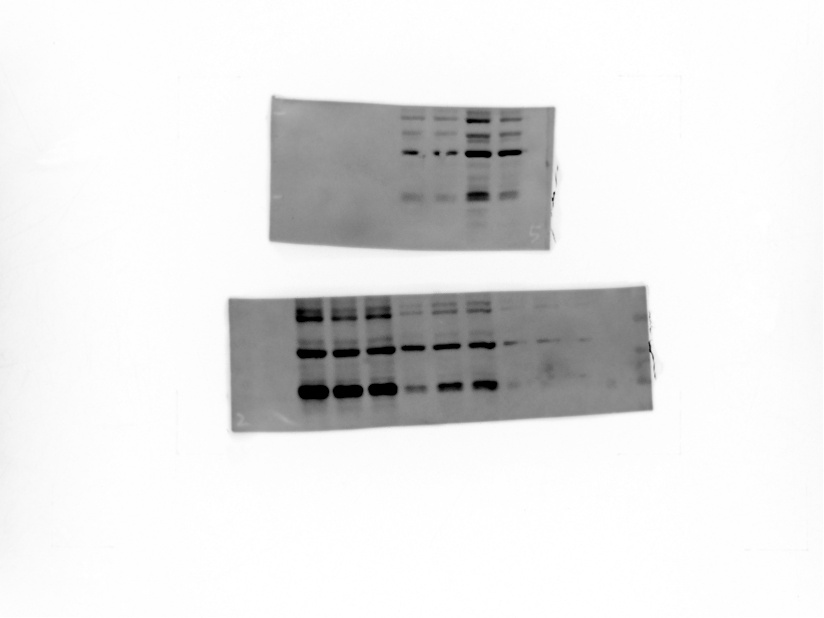

Supplement: Figure 5—source data 1. [file elife-98372-fig5-data1.zip › Figure 5-data1/Figure_5-source_data_1_ Figure_5E_cl-CASP9.jpg]

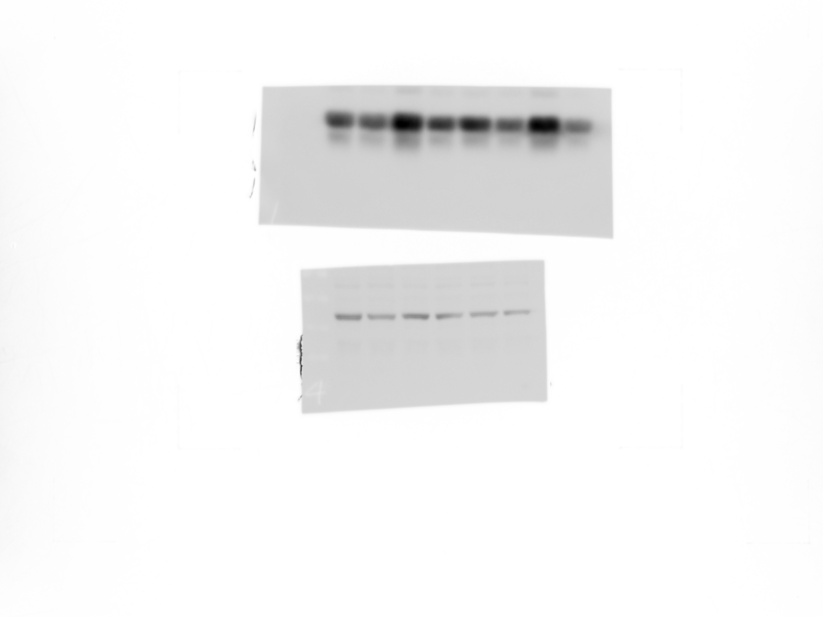

Supplement: Figure 5—source data 1. [file elife-98372-fig5-data1.zip › Figure 5-data1/Figure_5-source_data_1_ Figure_5E_NOXA.jpg]

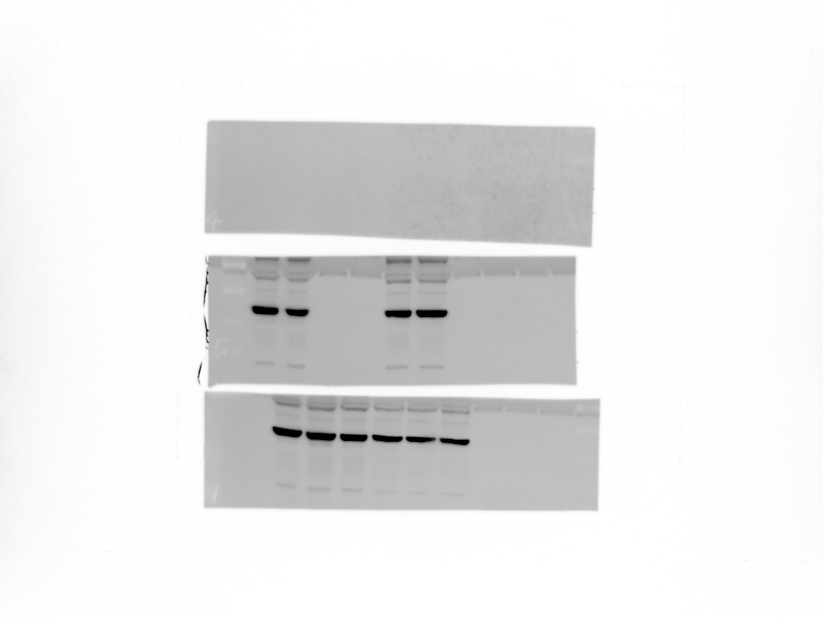

Supplement: Figure 5—source data 1. [file elife-98372-fig5-data1.zip › Figure 5-data1/Figure_5-source_data_1_ Figure_5E_WSB2.jpg]

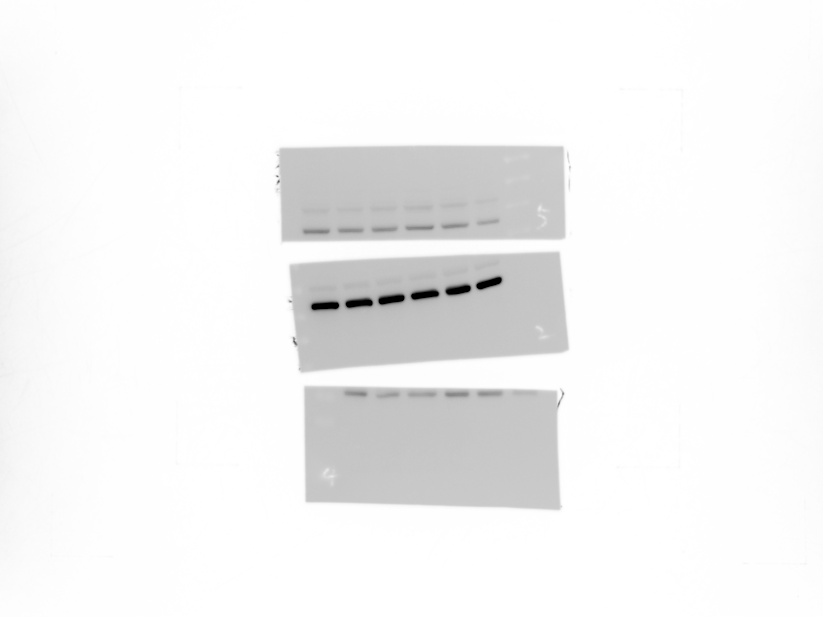

Supplement: Figure 5—source data 1. [file elife-98372-fig5-data1.zip › Figure 5-data1/Figure_5-source_data_1_ Figure_5G_Actin.jpg]

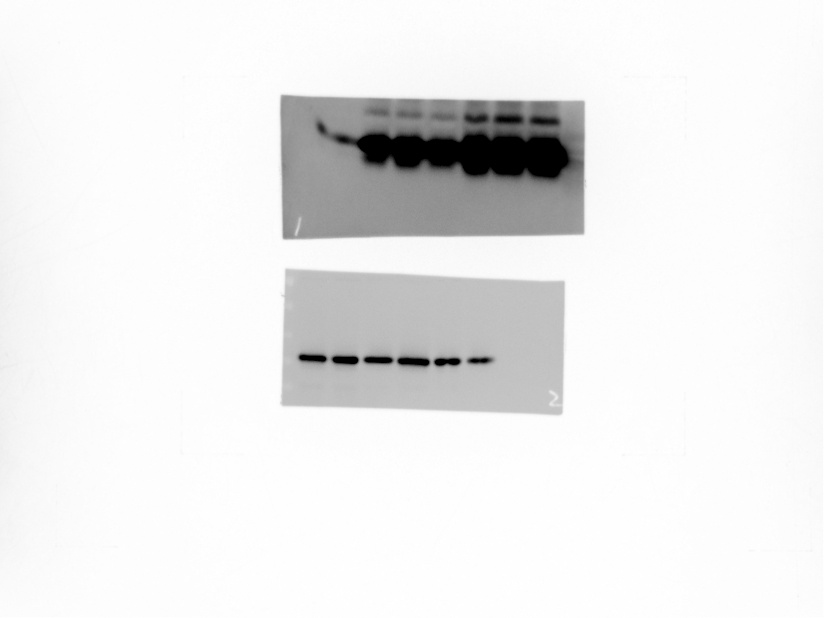

Supplement: Figure 5—source data 1. [file elife-98372-fig5-data1.zip › Figure 5-data1/Figure_5-source_data_1_ Figure_5G_CASP3.jpg]

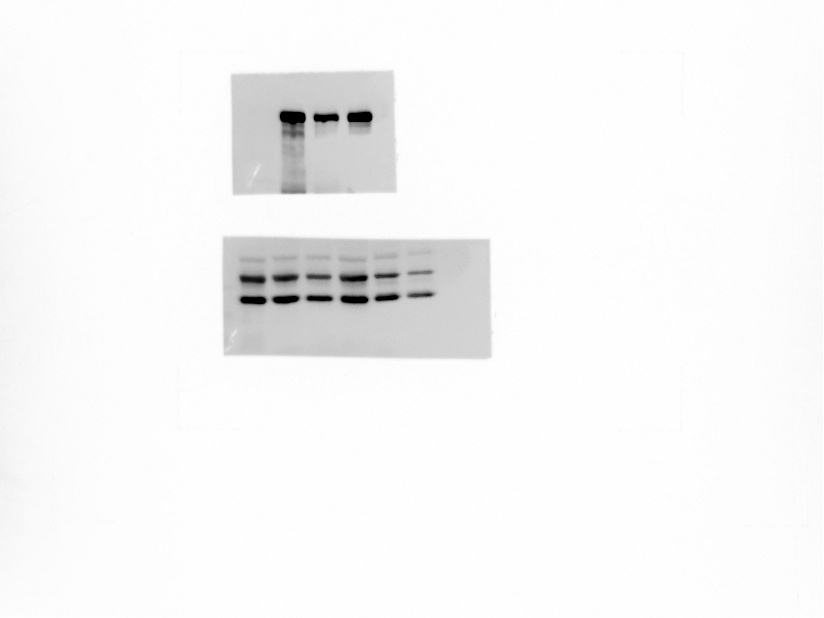

Supplement: Figure 5—source data 1. [file elife-98372-fig5-data1.zip › Figure 5-data1/Figure_5-source_data_1_ Figure_5G_CASP7.jpg]

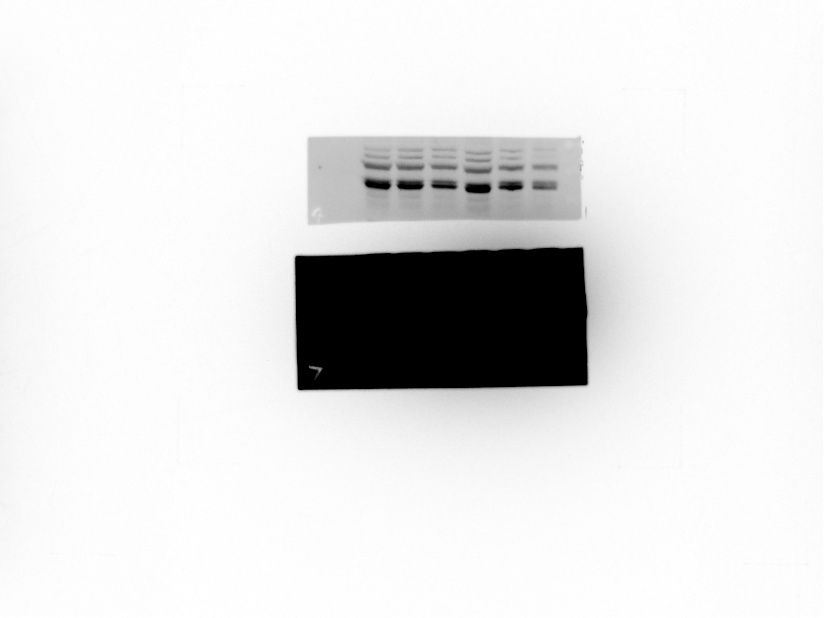

Supplement: Figure 5—source data 1. [file elife-98372-fig5-data1.zip › Figure 5-data1/Figure_5-source_data_1_ Figure_5G_CASP9.jpg]

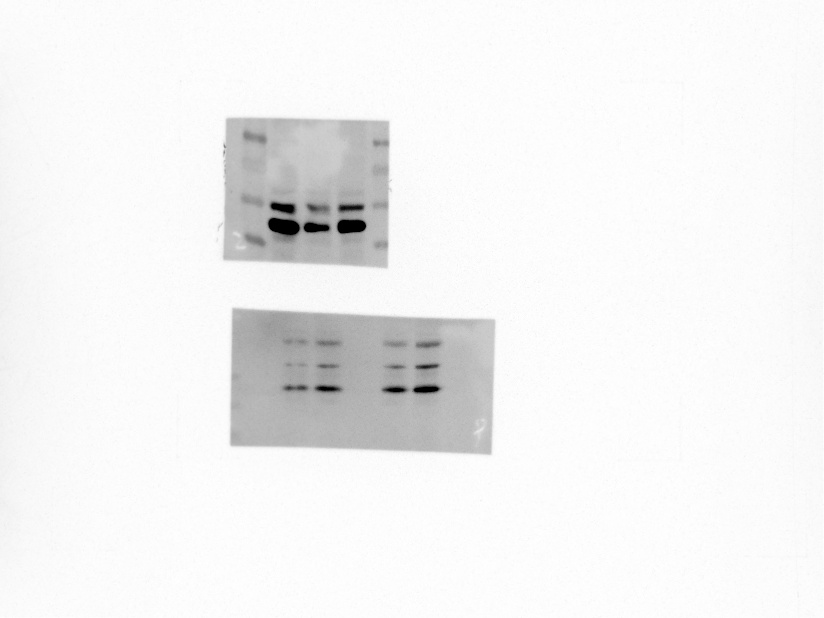

Supplement: Figure 5—source data 1. [file elife-98372-fig5-data1.zip › Figure 5-data1/Figure_5-source_data_1_ Figure_5G_cl-CASP3.jpg]

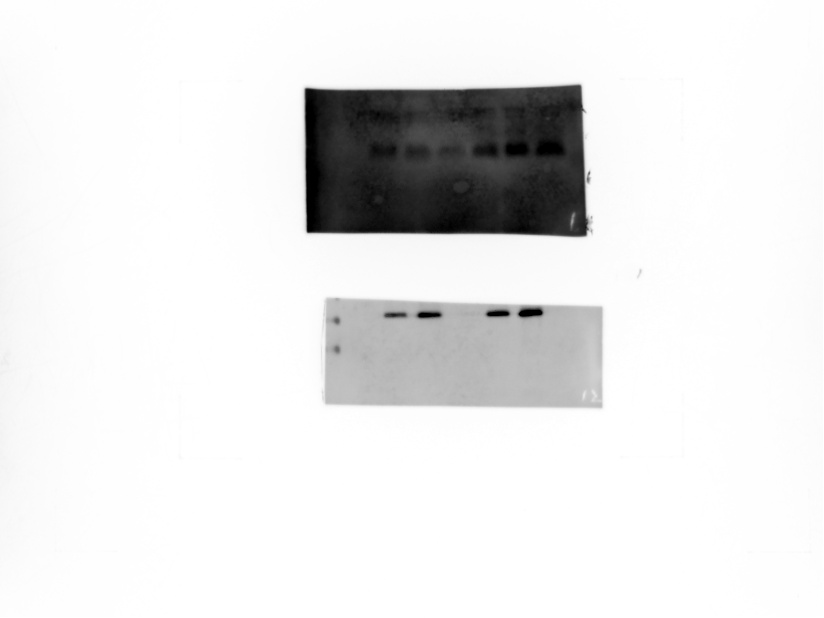

Supplement: Figure 5—source data 1. [file elife-98372-fig5-data1.zip › Figure 5-data1/Figure_5-source_data_1_ Figure_5G_cl-CASP7.jpg]

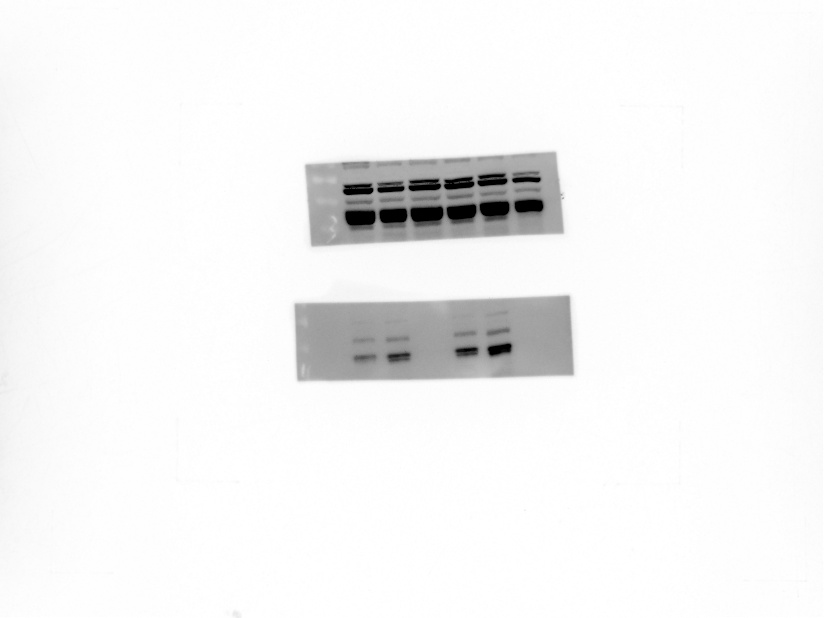

Supplement: Figure 5—source data 1. [file elife-98372-fig5-data1.zip › Figure 5-data1/Figure_5-source_data_1_ Figure_5G_cl-CASP9.jpg]

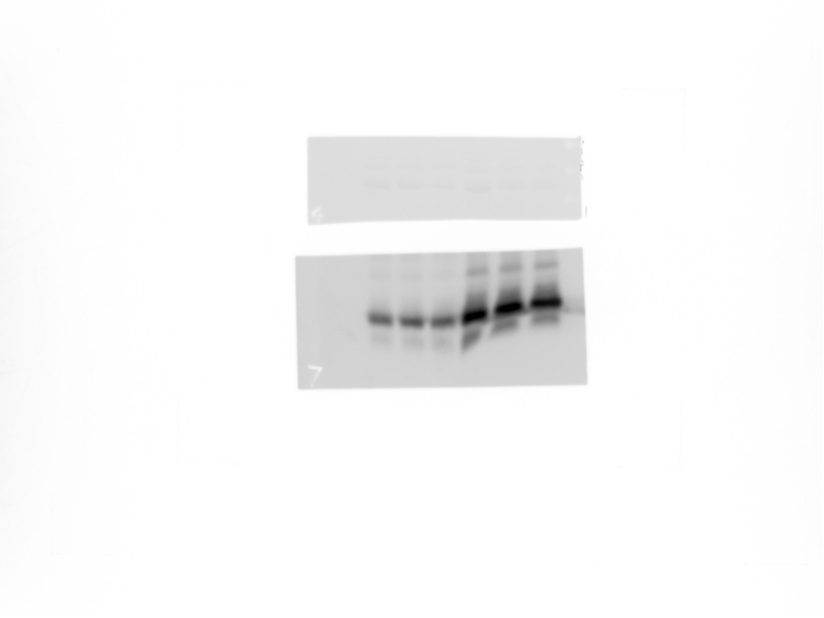

Supplement: Figure 5—source data 1. [file elife-98372-fig5-data1.zip › Figure 5-data1/Figure_5-source_data_1_ Figure_5G_NOXA.jpg]

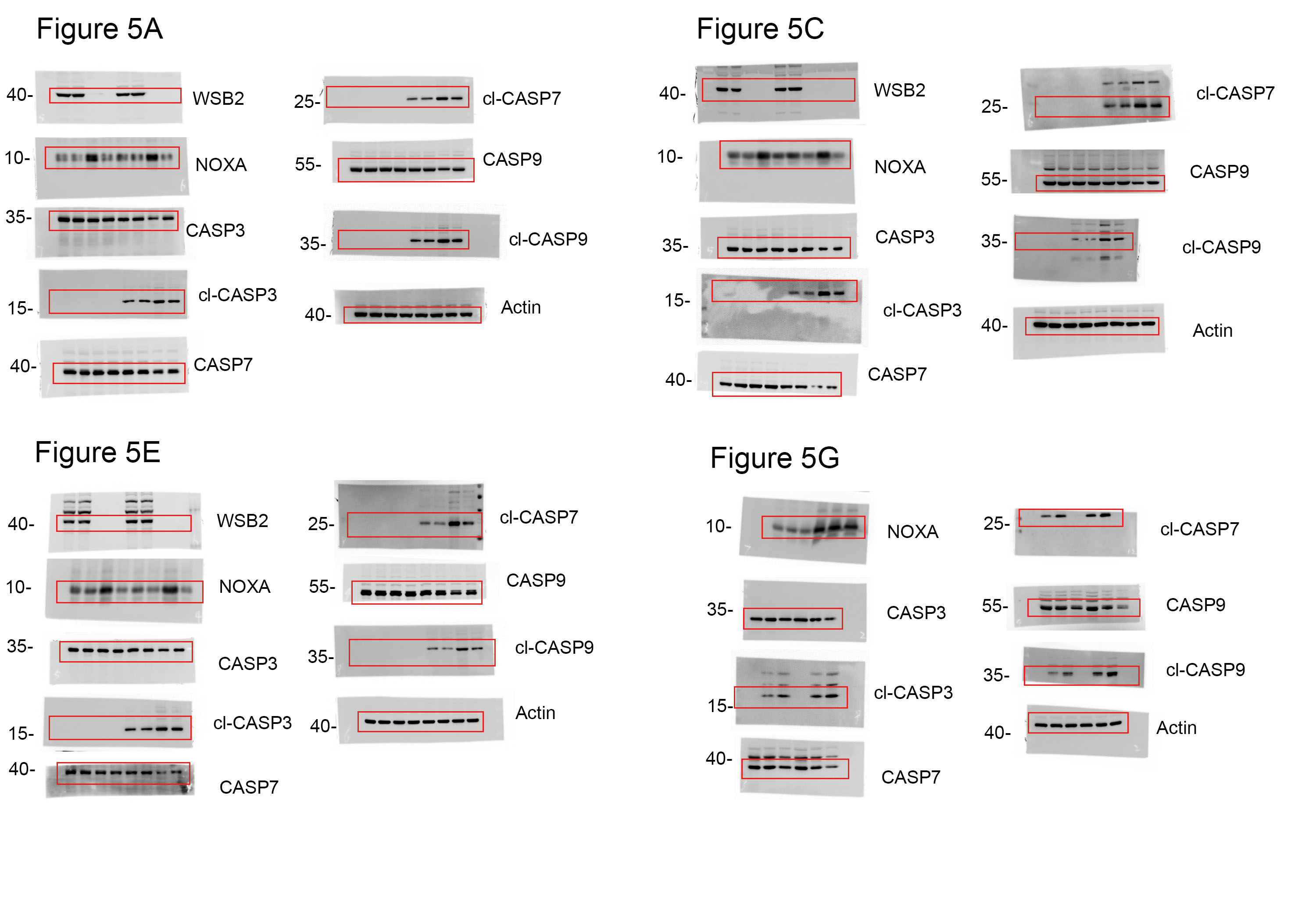

Supplement: Figure 5—source data 2. [file elife-98372-fig5-data2.zip › Figure 5-data2/Figure_5_data_2 .jpg]

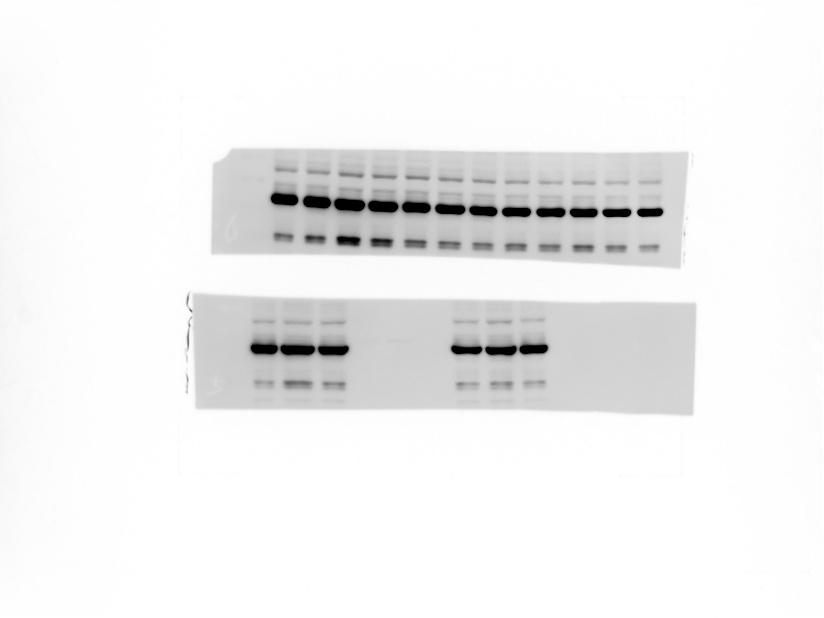

Supplement: Figure 6—source data 1. [file elife-98372-fig6-data1.zip › Figure 6-Source Data 1/Figure_6-source_data_1_ Figure_6A_Actin.jpg]

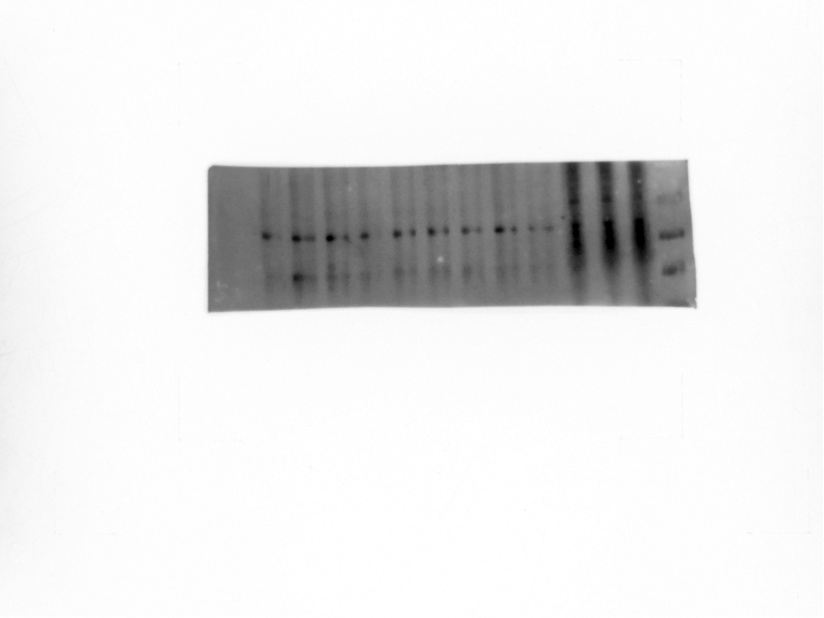

Supplement: Figure 6—source data 1. [file elife-98372-fig6-data1.zip › Figure 6-Source Data 1/Figure_6-source_data_1_ Figure_6A_cl-CASP3.jpg]

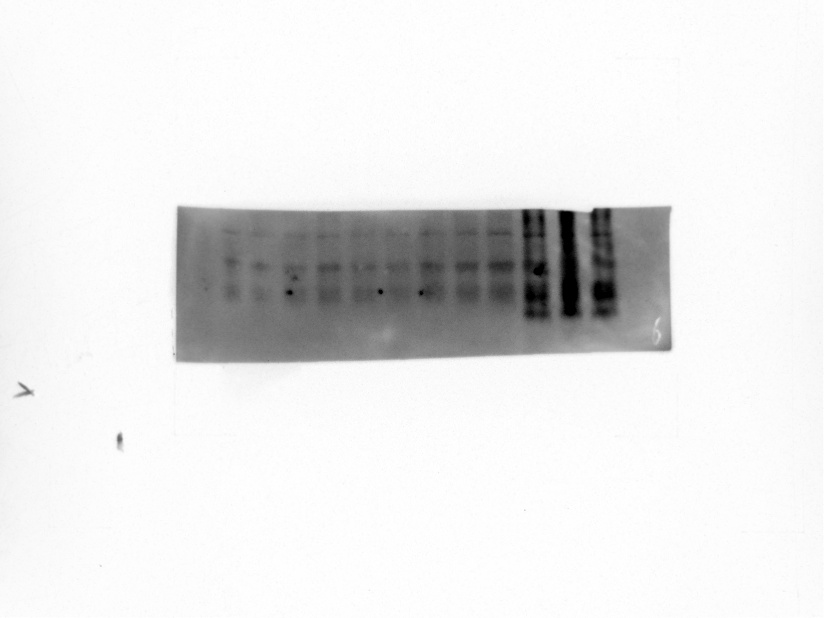

Supplement: Figure 6—source data 1. [file elife-98372-fig6-data1.zip › Figure 6-Source Data 1/Figure_6-source_data_1_ Figure_6A_cl-CASP7.jpg]

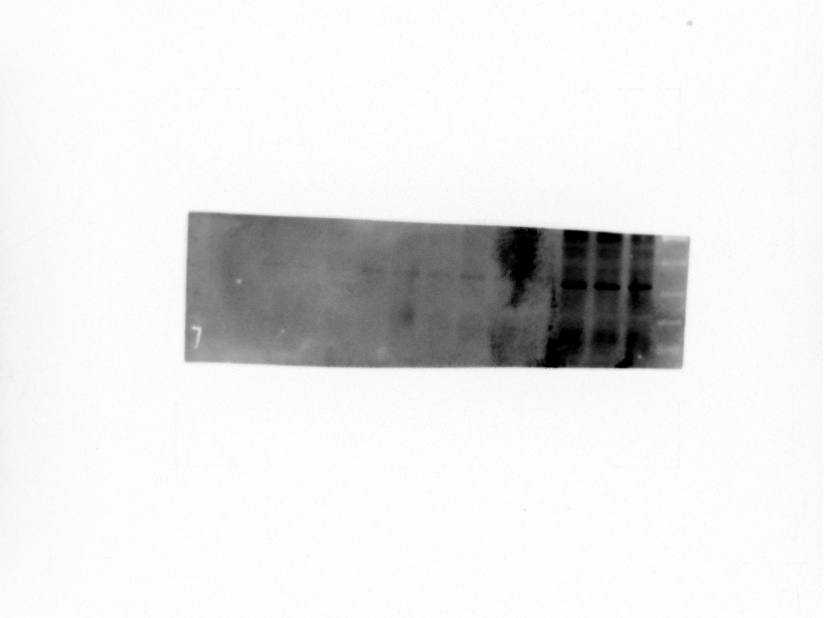

Supplement: Figure 6—source data 1. [file elife-98372-fig6-data1.zip › Figure 6-Source Data 1/Figure_6-source_data_1_ Figure_6A_cl-CASP9.jpg]

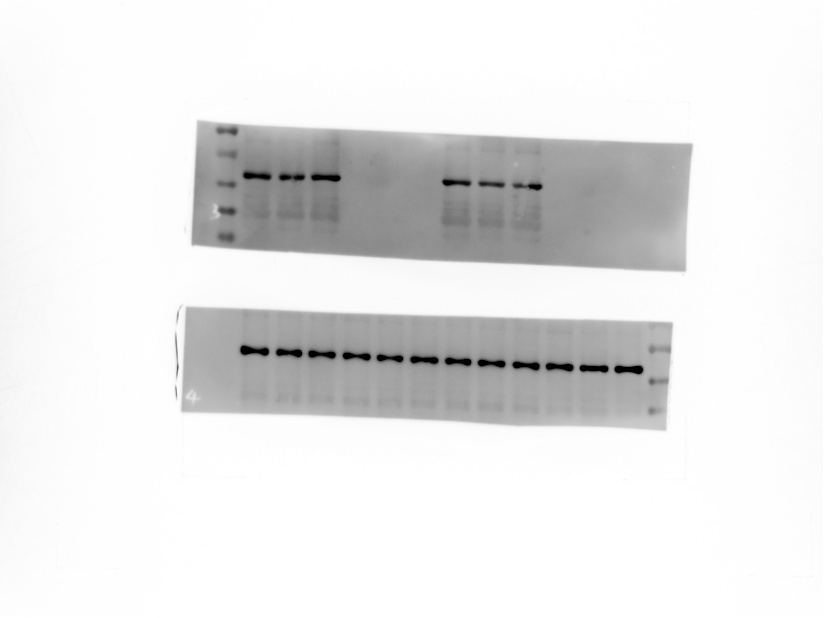

Supplement: Figure 6—source data 1. [file elife-98372-fig6-data1.zip › Figure 6-Source Data 1/Figure_6-source_data_1_ Figure_6B_Actin.jpg]
