# Supplementary figures and images for: Targeting the WSB2–NOXA axis in cancer cells for enhanced sensitivity to BCL-2 family protein inhibitors (part 4 of 5)
Source: eLife. 2025 Jul 23;13:RP98372. doi: 10.7554/eLife.98372 (PMC12286604; doi:10.7554/eLife.98372)

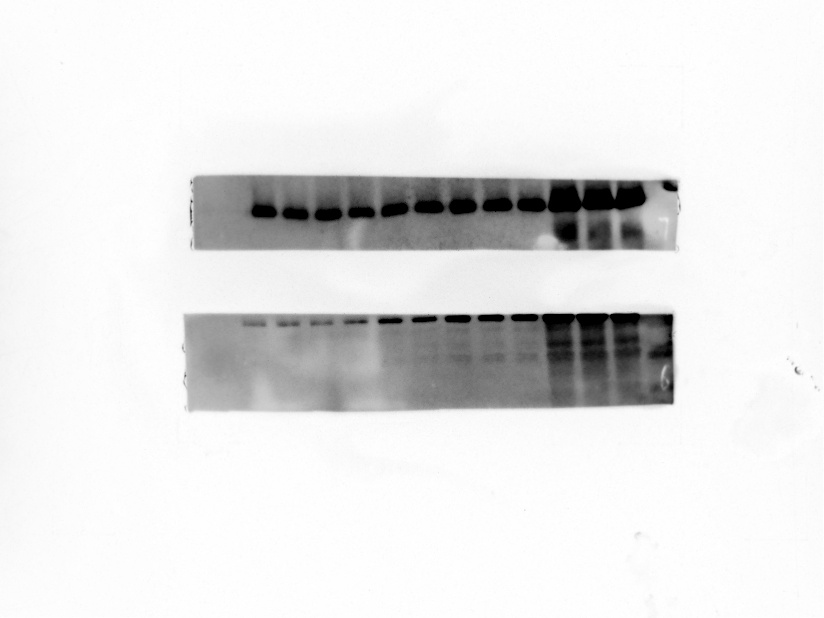

Supplement: Figure 6—source data 1. [file elife-98372-fig6-data1.zip › Figure 6-Source Data 1/Figure_6-source_data_1_ Figure_6B_cl-CASP3.jpg]

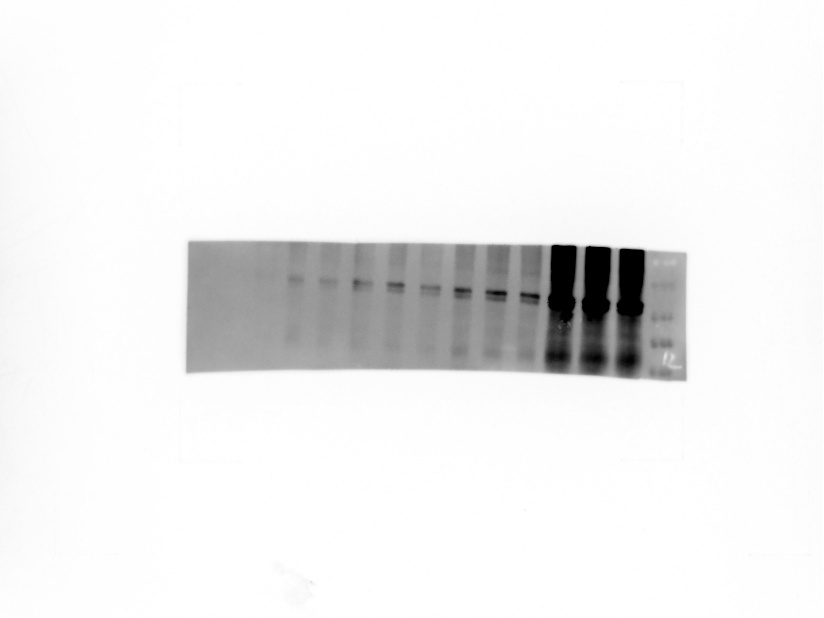

Supplement: Figure 6—source data 1. [file elife-98372-fig6-data1.zip › Figure 6-Source Data 1/Figure_6-source_data_1_ Figure_6B_cl-CASP7.jpg]

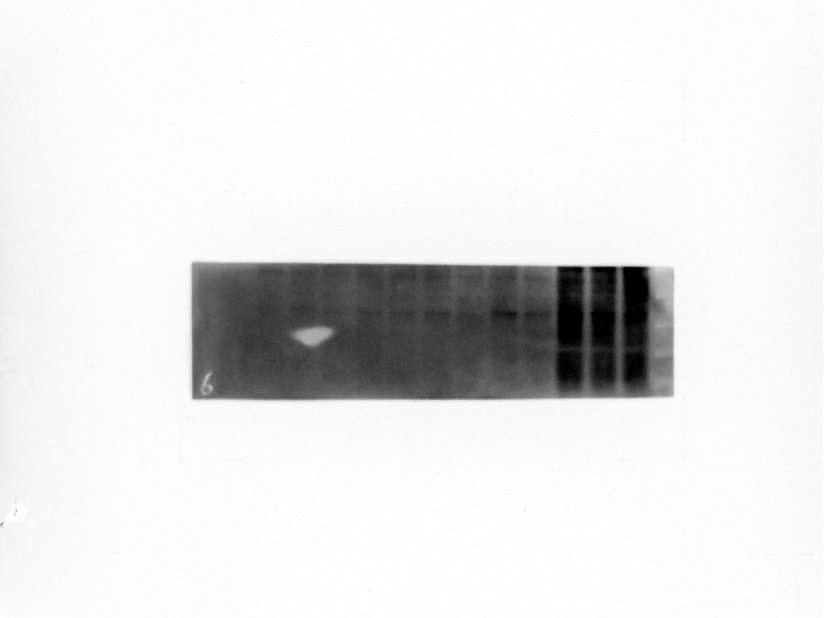

Supplement: Figure 6—source data 1. [file elife-98372-fig6-data1.zip › Figure 6-Source Data 1/Figure_6-source_data_1_ Figure_6B_cl-CASP9.jpg]

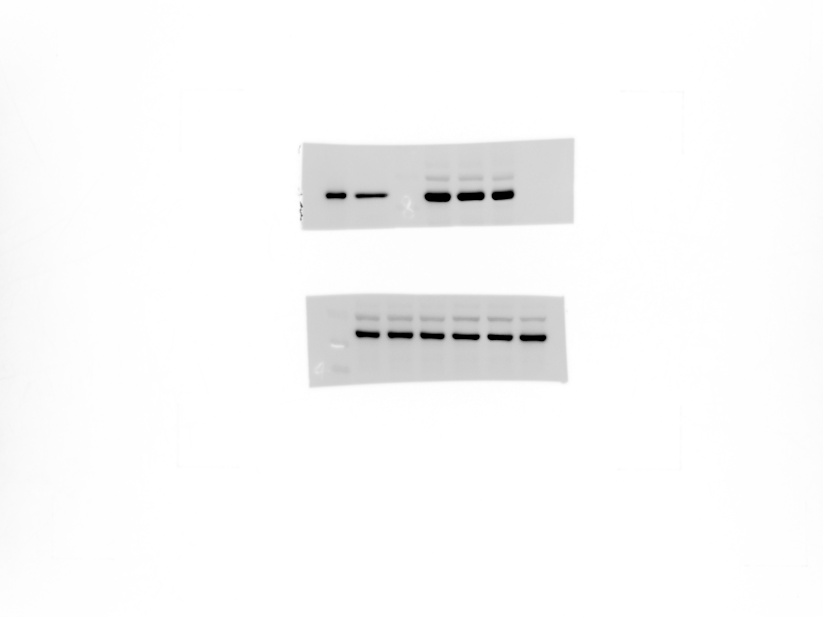

Supplement: Figure 6—source data 1. [file elife-98372-fig6-data1.zip › Figure 6-Source Data 1/Figure_6-source_data_1_ Figure_6F_Actin.jpg]

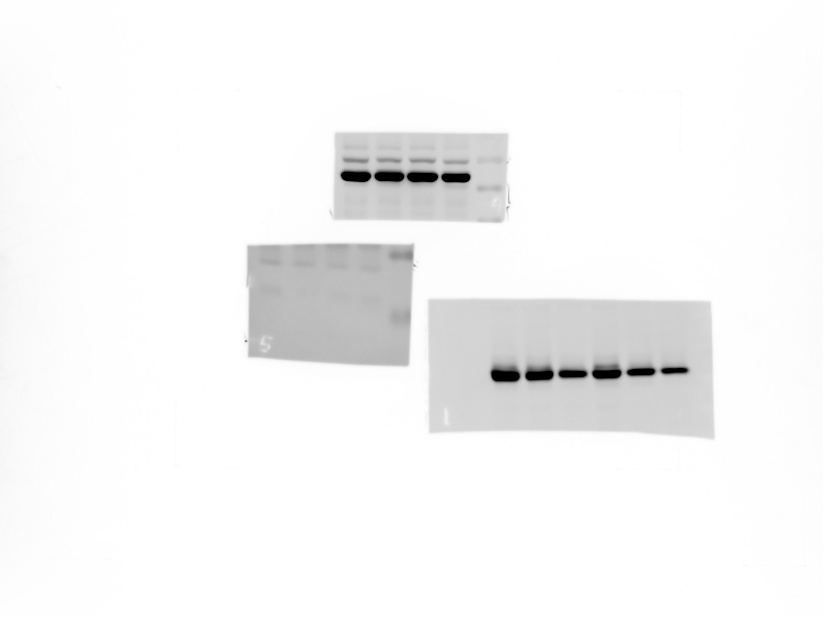

Supplement: Figure 6—source data 1. [file elife-98372-fig6-data1.zip › Figure 6-Source Data 1/Figure_6-source_data_1_ Figure_6F_CASP3.jpg]

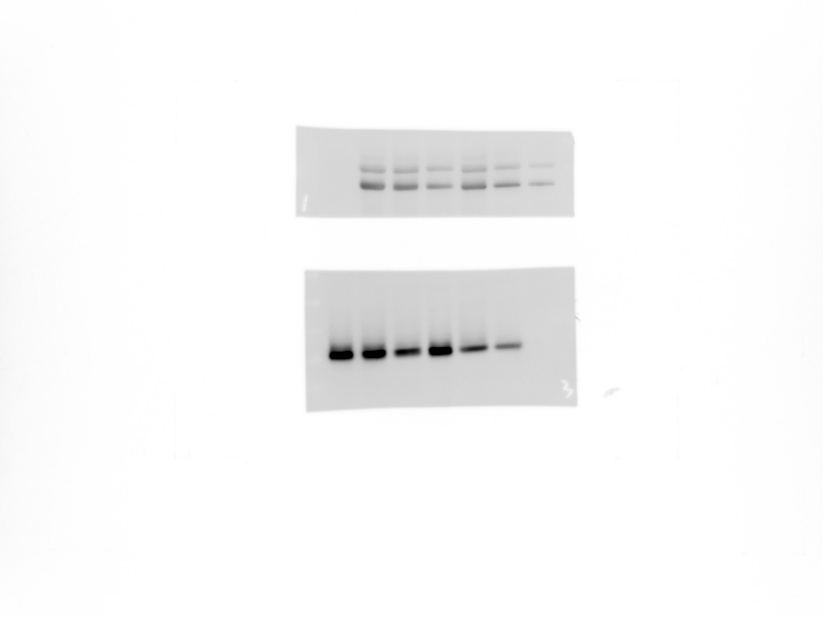

Supplement: Figure 6—source data 1. [file elife-98372-fig6-data1.zip › Figure 6-Source Data 1/Figure_6-source_data_1_ Figure_6F_CASP7.jpg]

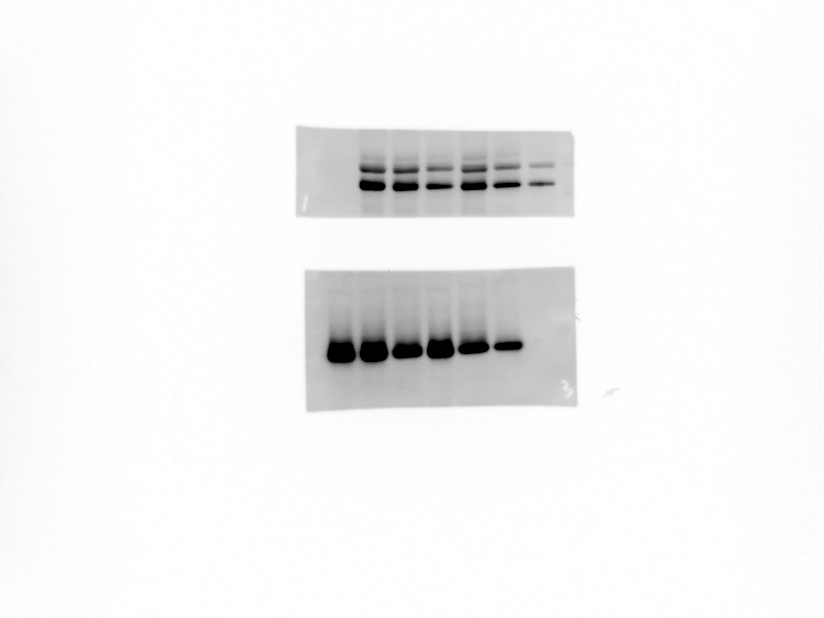

Supplement: Figure 6—source data 1. [file elife-98372-fig6-data1.zip › Figure 6-Source Data 1/Figure_6-source_data_1_ Figure_6F_CASP9.jpg]

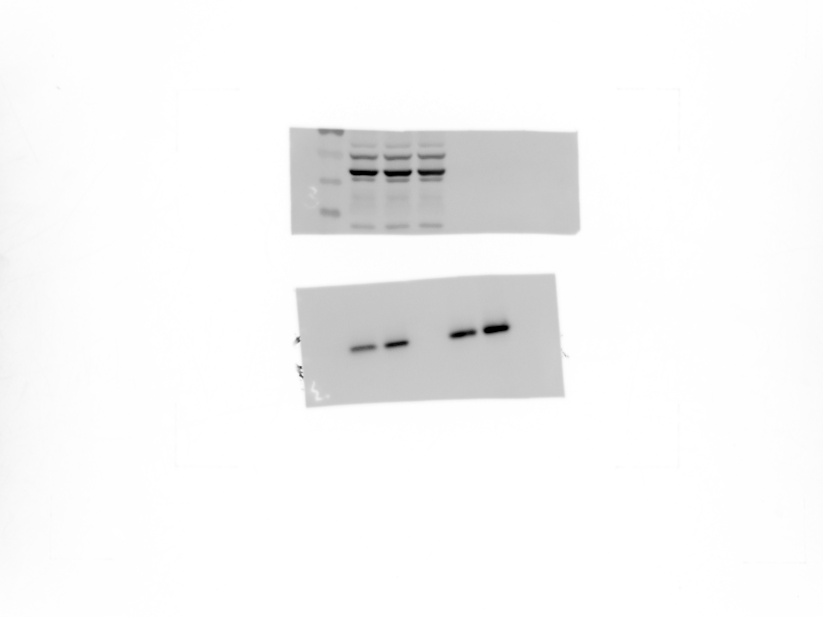

Supplement: Figure 6—source data 1. [file elife-98372-fig6-data1.zip › Figure 6-Source Data 1/Figure_6-source_data_1_ Figure_6F_cl-CASP3.jpg]

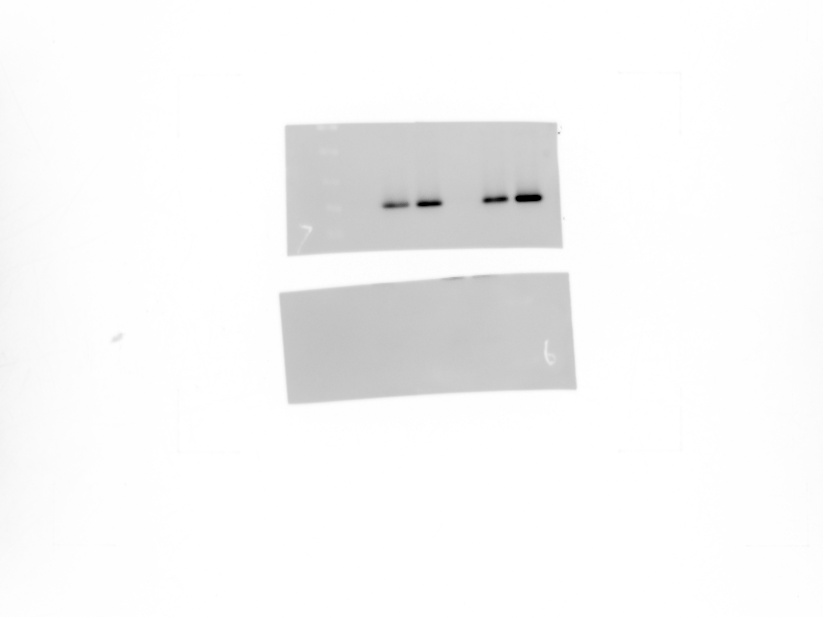

Supplement: Figure 6—source data 1. [file elife-98372-fig6-data1.zip › Figure 6-Source Data 1/Figure_6-source_data_1_ Figure_6F_cl-CASP7.jpg]

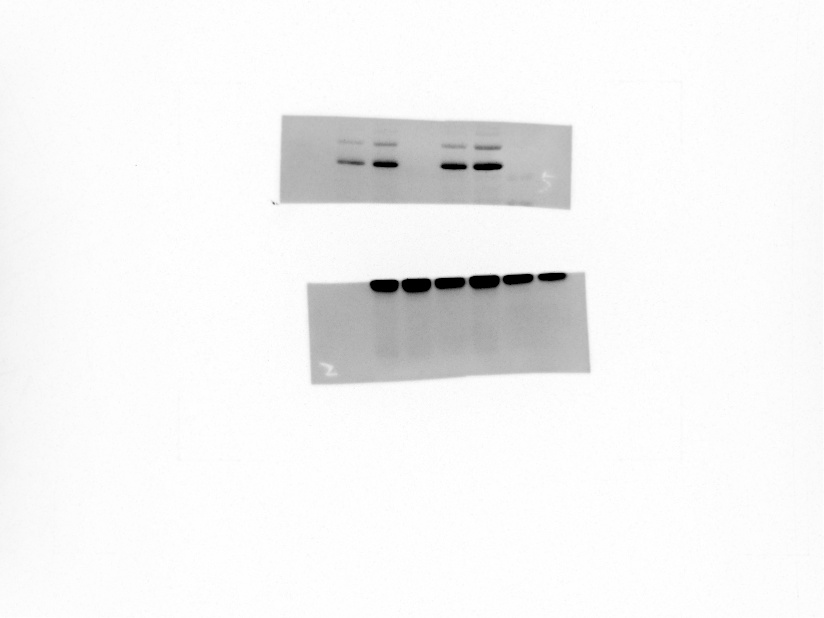

Supplement: Figure 6—source data 1. [file elife-98372-fig6-data1.zip › Figure 6-Source Data 1/Figure_6-source_data_1_ Figure_6F_cl-CASP9.jpg]

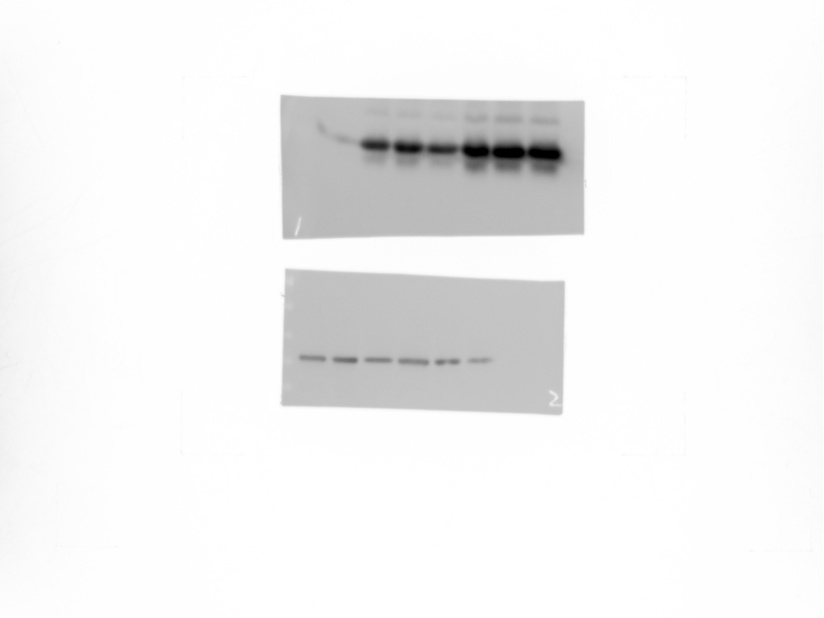

Supplement: Figure 6—source data 1. [file elife-98372-fig6-data1.zip › Figure 6-Source Data 1/Figure_6-source_data_1_ Figure_6F_NOXA.jpg]

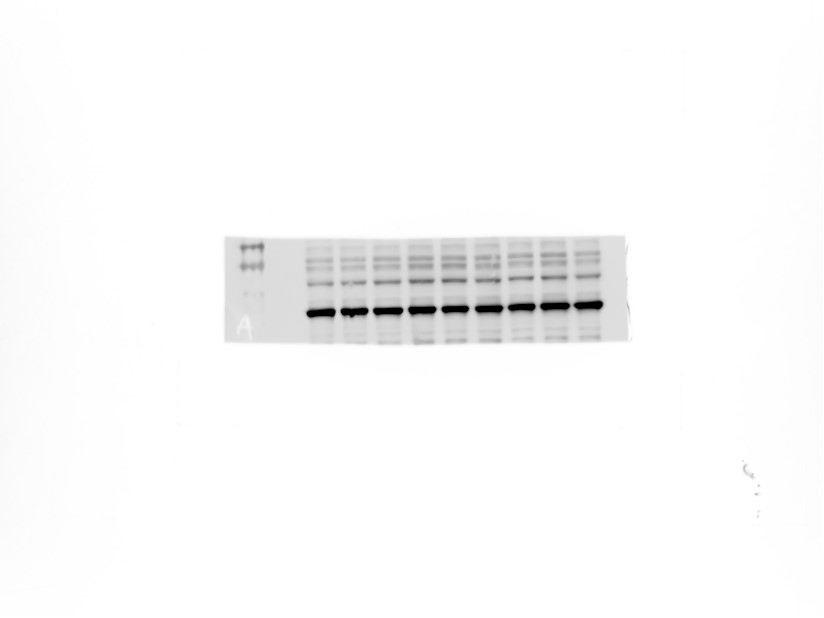

Supplement: Figure 6—source data 1. [file elife-98372-fig6-data1.zip › Figure 6-Source Data 1/Figure_6-source_data_1_ Figure_6H_Actin.jpg]

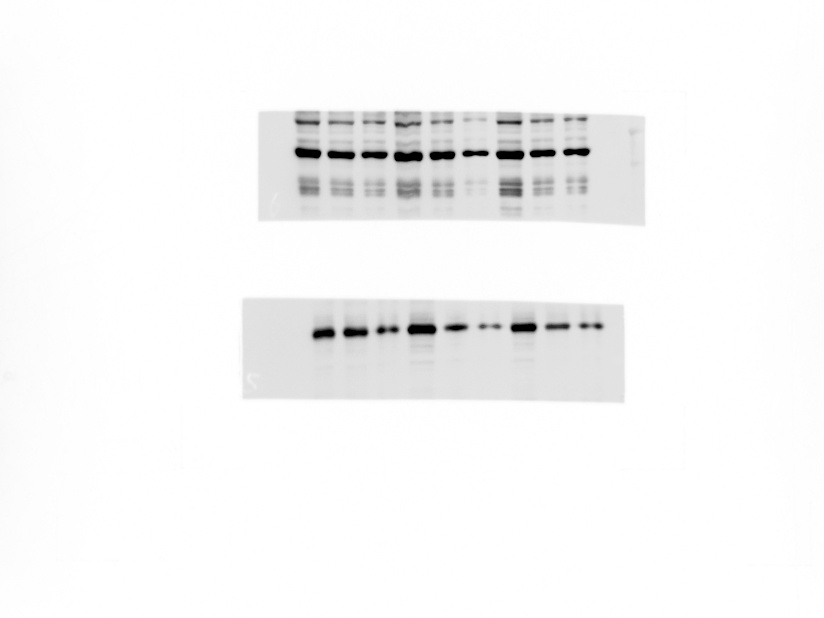

Supplement: Figure 6—source data 1. [file elife-98372-fig6-data1.zip › Figure 6-Source Data 1/Figure_6-source_data_1_ Figure_6H_CASP3.jpg]

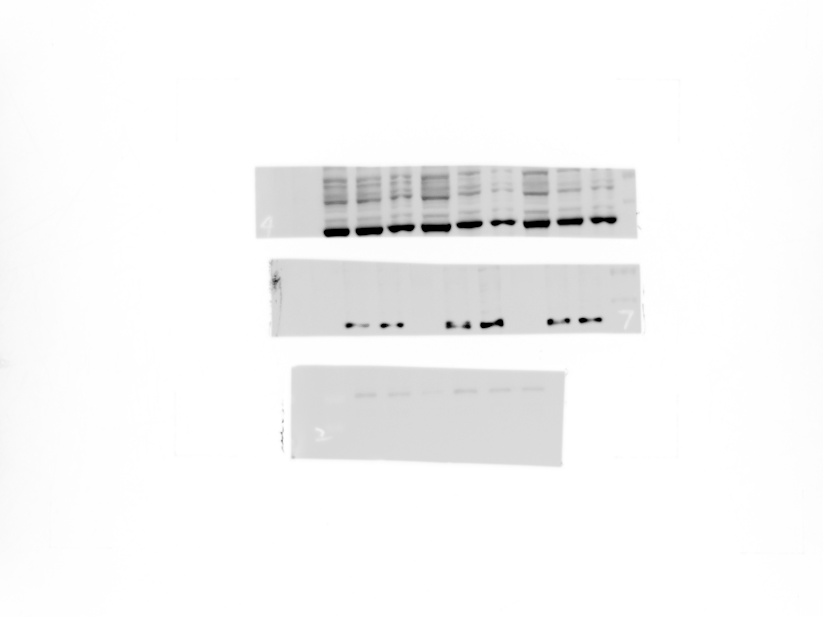

Supplement: Figure 6—source data 1. [file elife-98372-fig6-data1.zip › Figure 6-Source Data 1/Figure_6-source_data_1_ Figure_6H_CASP9.jpg]

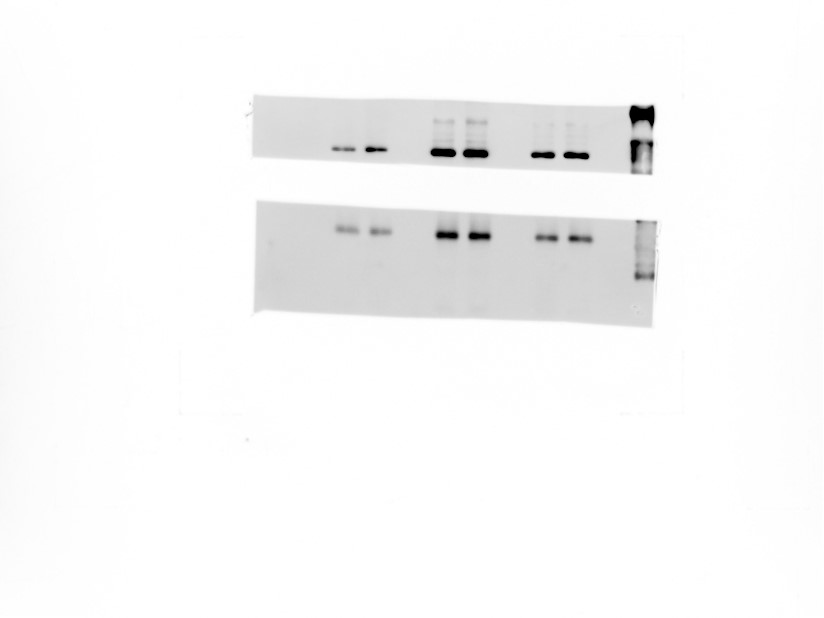

Supplement: Figure 6—source data 1. [file elife-98372-fig6-data1.zip › Figure 6-Source Data 1/Figure_6-source_data_1_ Figure_6H_cl-CASP3.jpg]

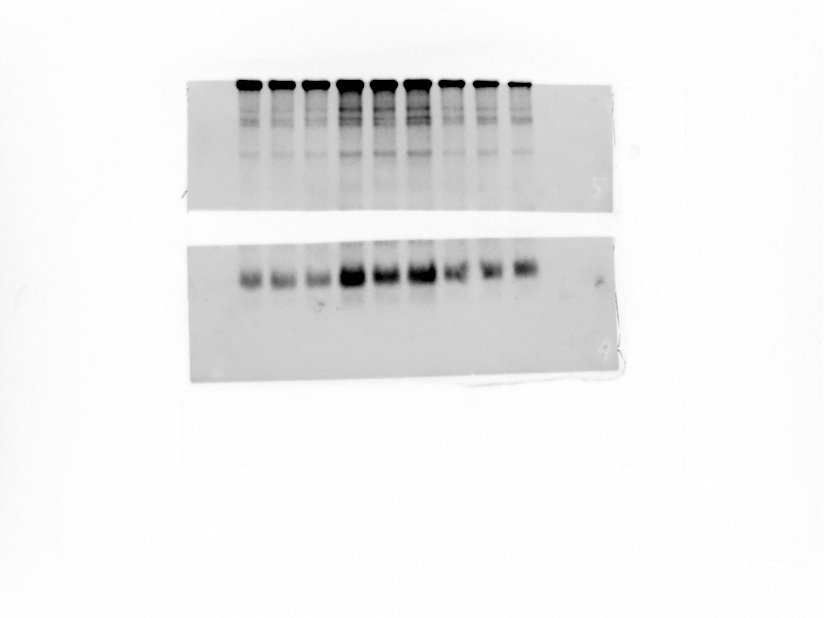

Supplement: Figure 6—source data 1. [file elife-98372-fig6-data1.zip › Figure 6-Source Data 1/Figure_6-source_data_1_ Figure_6H_NOXA.jpg]

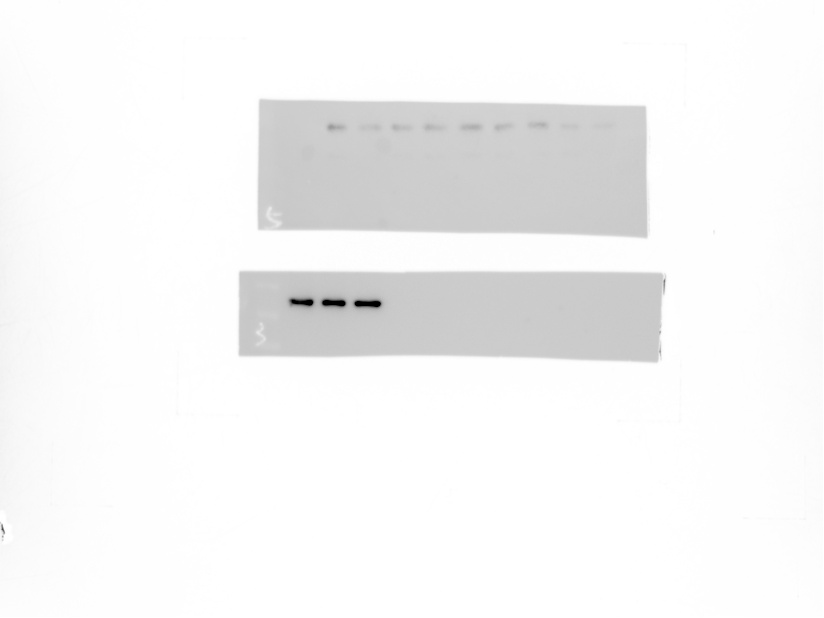

Supplement: Figure 6—source data 1. [file elife-98372-fig6-data1.zip › Figure 6-Source Data 1/Figure_6-source_data_1_ Figure_6H_WSB2.jpg]

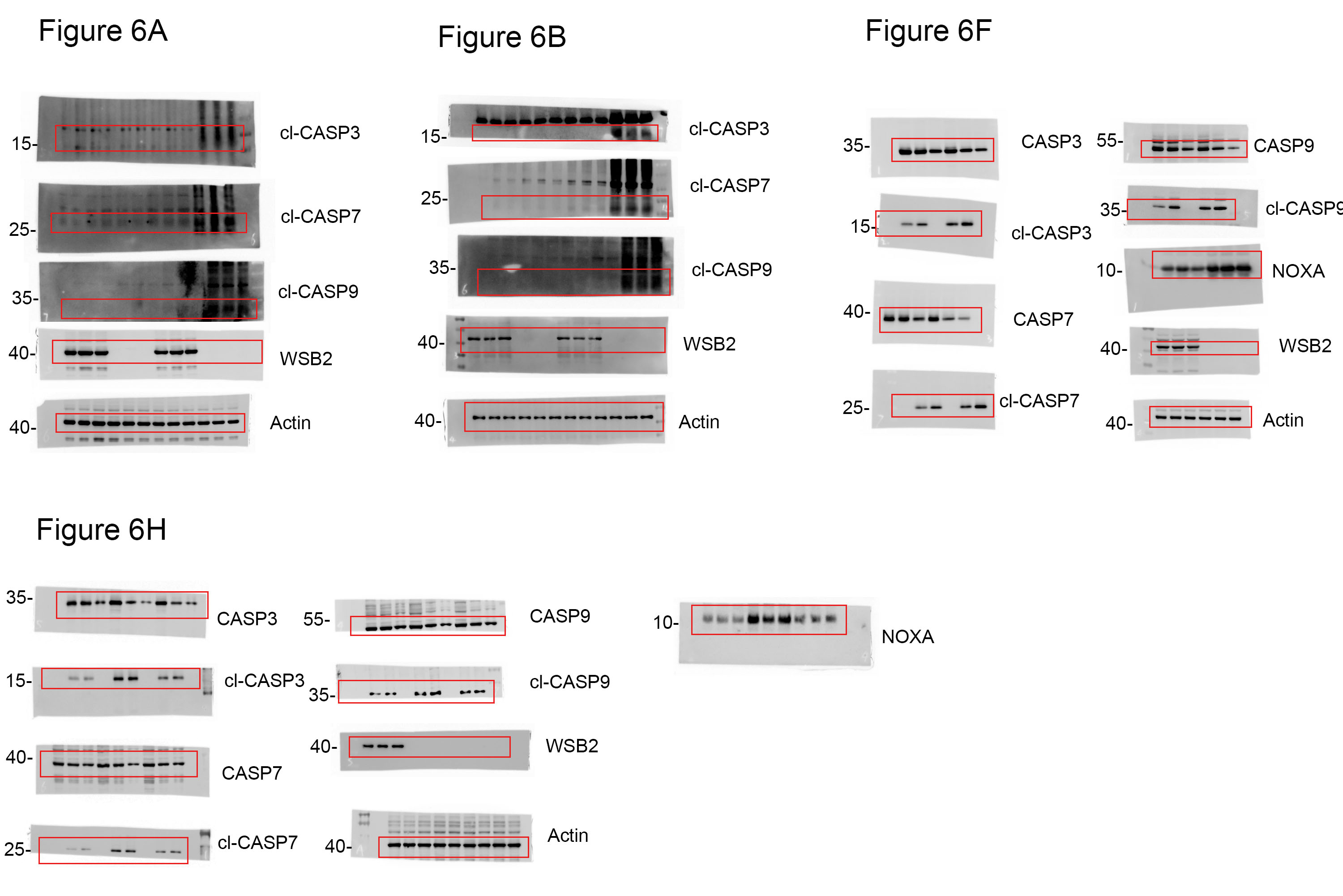

Supplement: Figure 6—source data 2. [file elife-98372-fig6-data2.zip › Figure 6-data2/Figure_6_data_2 .jpg]

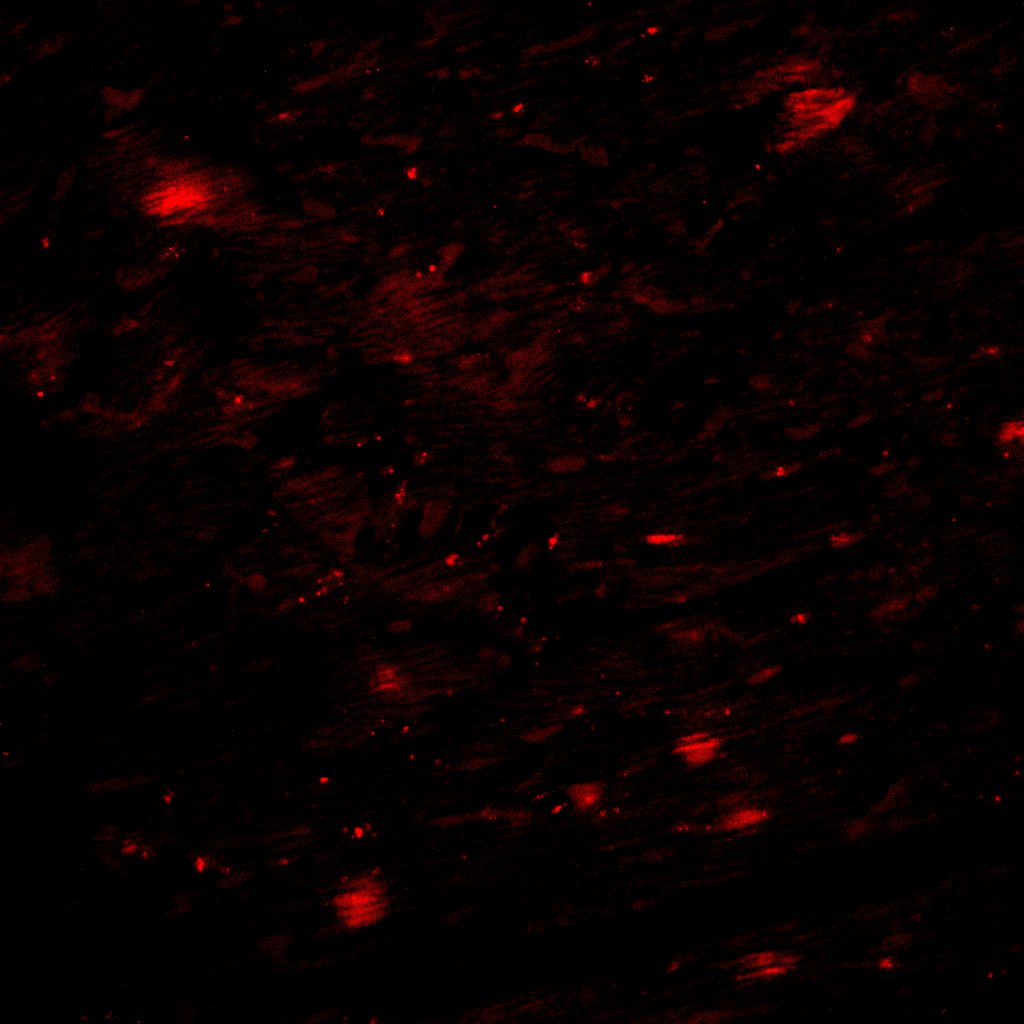

Supplement: Figure 6—source data 3. [file elife-98372-fig6-data3.zip › Figure 6-Source Data 3/Figure_6-source_data_1_Figure_6C_Heart_homo_ABT-199_cl-CASP3(cl-CASP3).jpg]

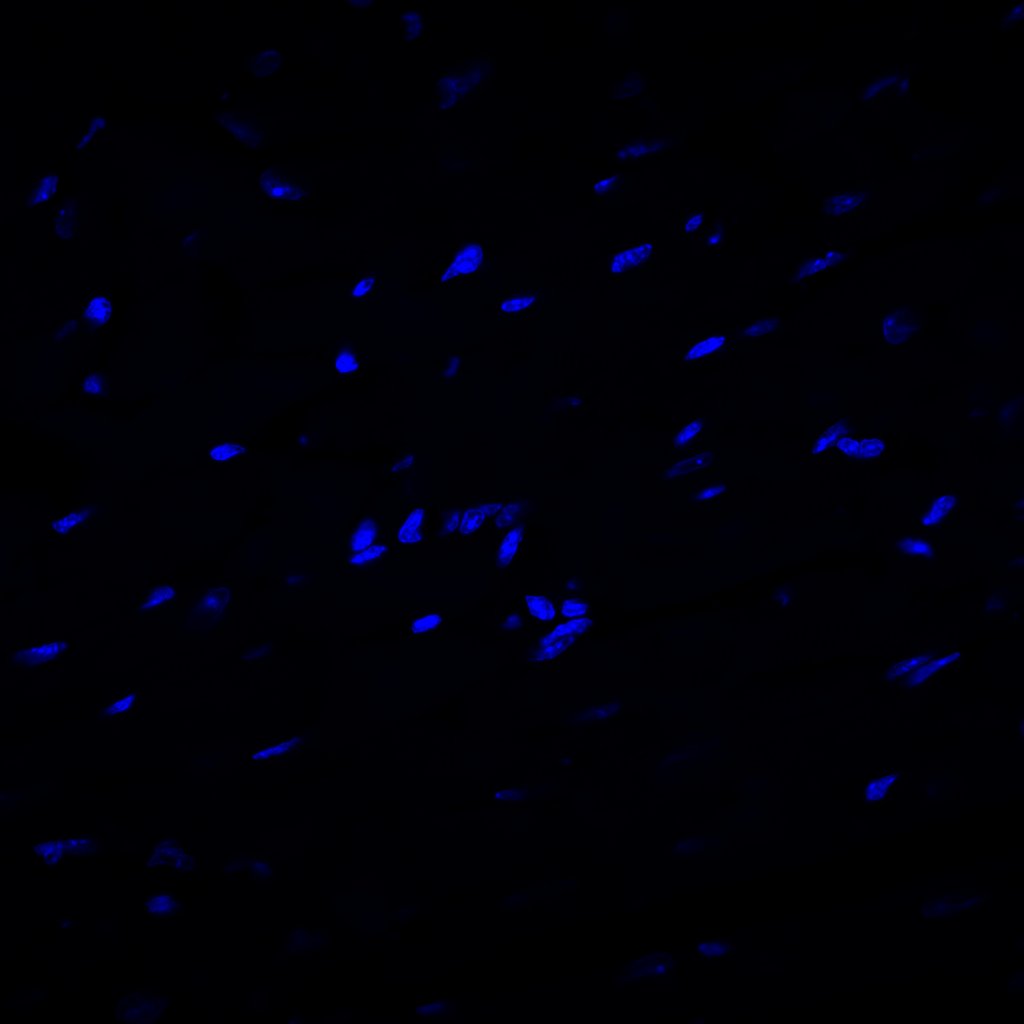

Supplement: Figure 6—source data 3. [file elife-98372-fig6-data3.zip › Figure 6-Source Data 3/Figure_6-source_data_1_Figure_6C_Heart_homo_ABT-199_cl-CASP3(DAPI).jpg]

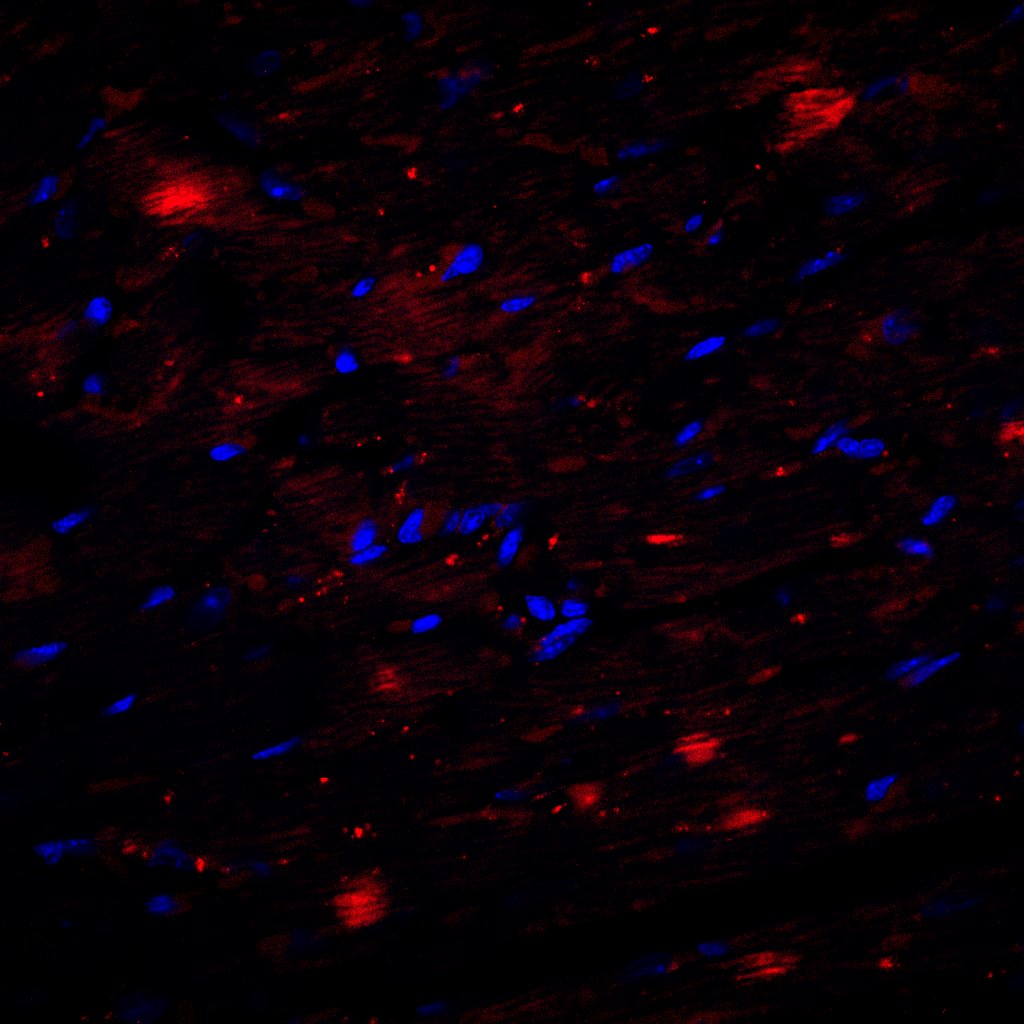

Supplement: Figure 6—source data 3. [file elife-98372-fig6-data3.zip › Figure 6-Source Data 3/Figure_6-source_data_1_Figure_6C_Heart_homo_ABT-199_cl-CASP3(Merge).jpg]

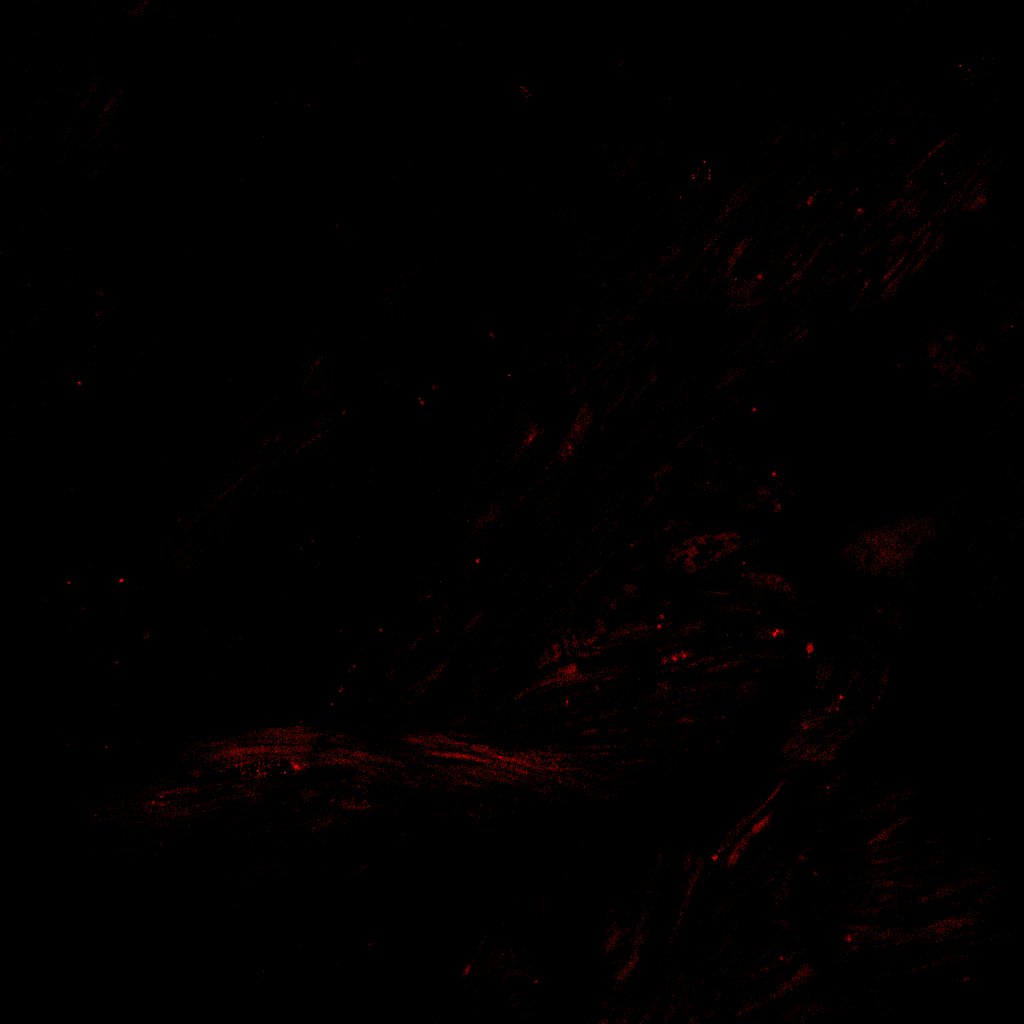

Supplement: Figure 6—source data 3. [file elife-98372-fig6-data3.zip › Figure 6-Source Data 3/Figure_6-source_data_1_Figure_6C_Heart_homo_cl-CASP3(cl-CASP3).jpg]

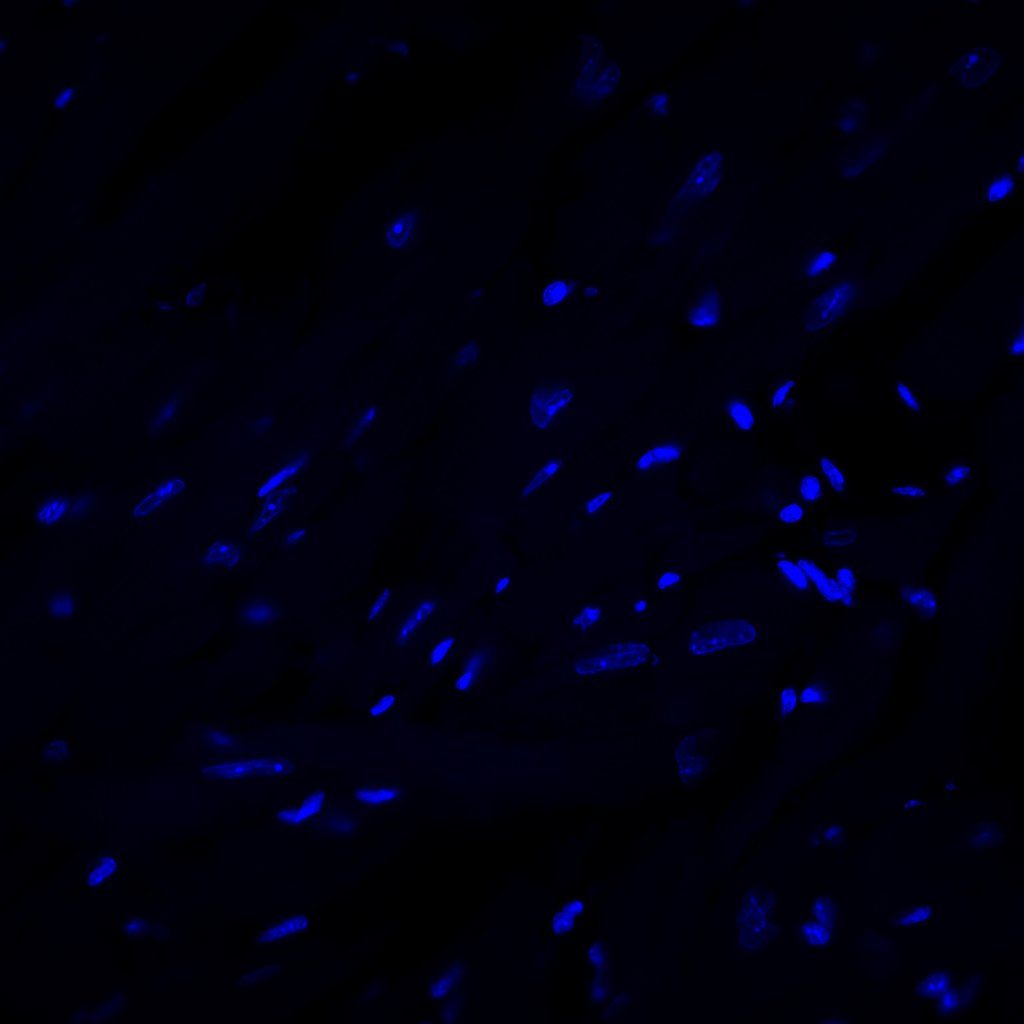

Supplement: Figure 6—source data 3. [file elife-98372-fig6-data3.zip › Figure 6-Source Data 3/Figure_6-source_data_1_Figure_6C_Heart_homo_cl-CASP3(DAPI).jpg]

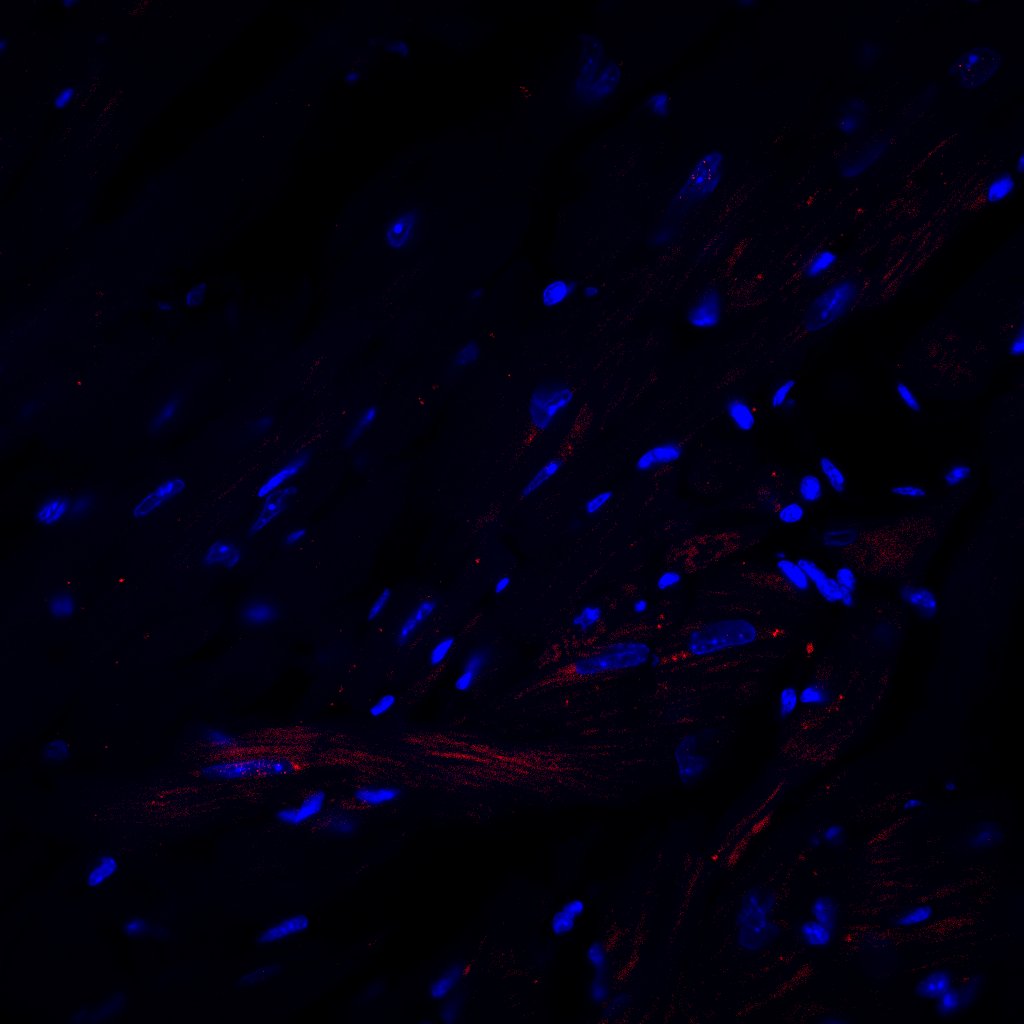

Supplement: Figure 6—source data 3. [file elife-98372-fig6-data3.zip › Figure 6-Source Data 3/Figure_6-source_data_1_Figure_6C_Heart_homo_cl-CASP3(Merge).jpg]

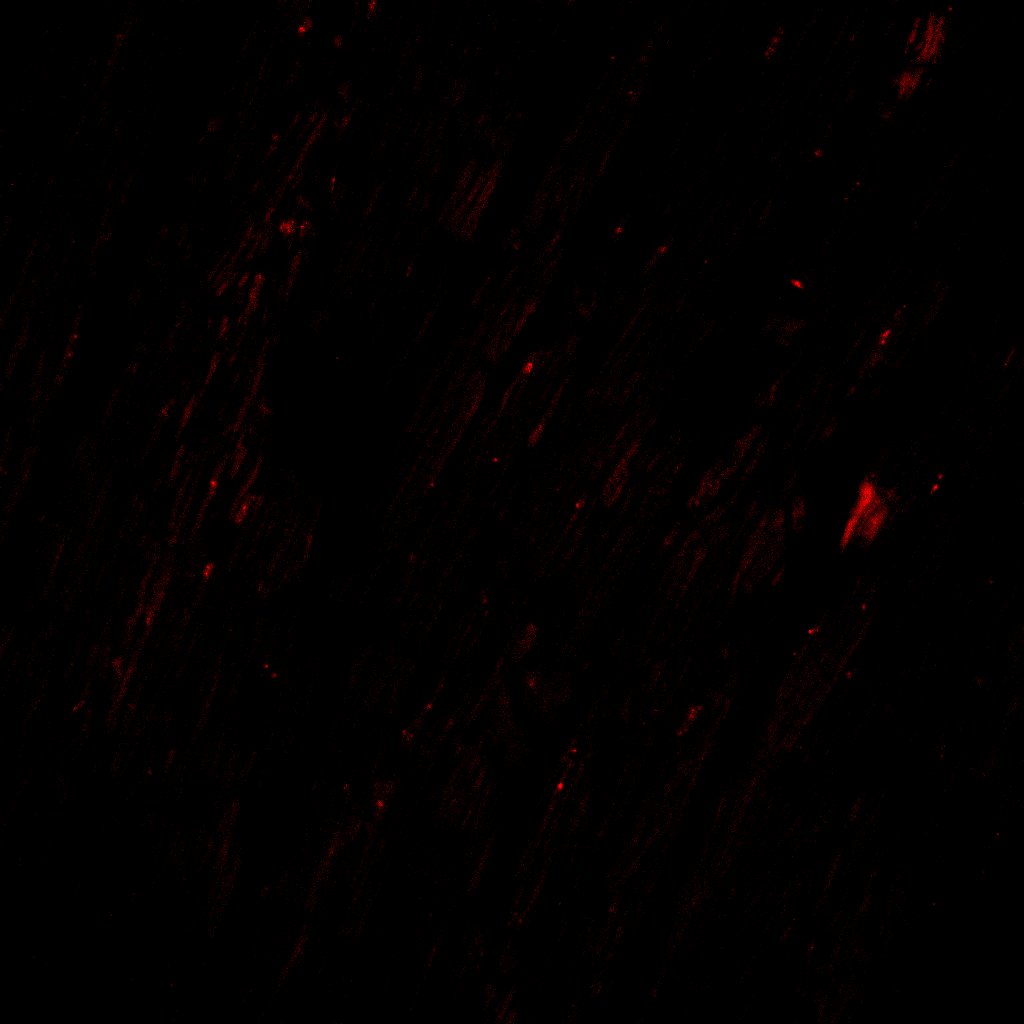

Supplement: Figure 6—source data 3. [file elife-98372-fig6-data3.zip › Figure 6-Source Data 3/Figure_6-source_data_1_Figure_6C_Heart_WT_ABT-199_cl-CASP3(cl-CASP3).jpg]

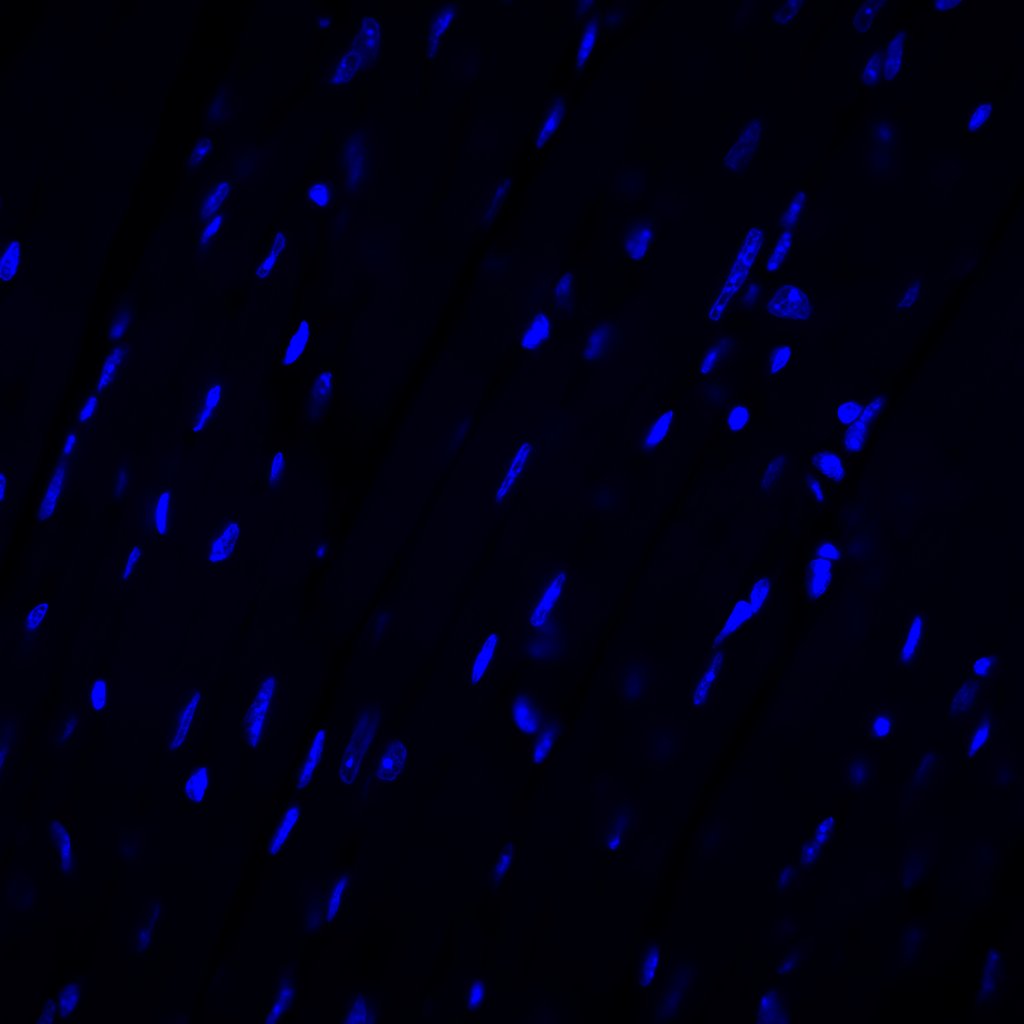

Supplement: Figure 6—source data 3. [file elife-98372-fig6-data3.zip › Figure 6-Source Data 3/Figure_6-source_data_1_Figure_6C_Heart_WT_ABT-199_cl-CASP3(DAPI).jpg]

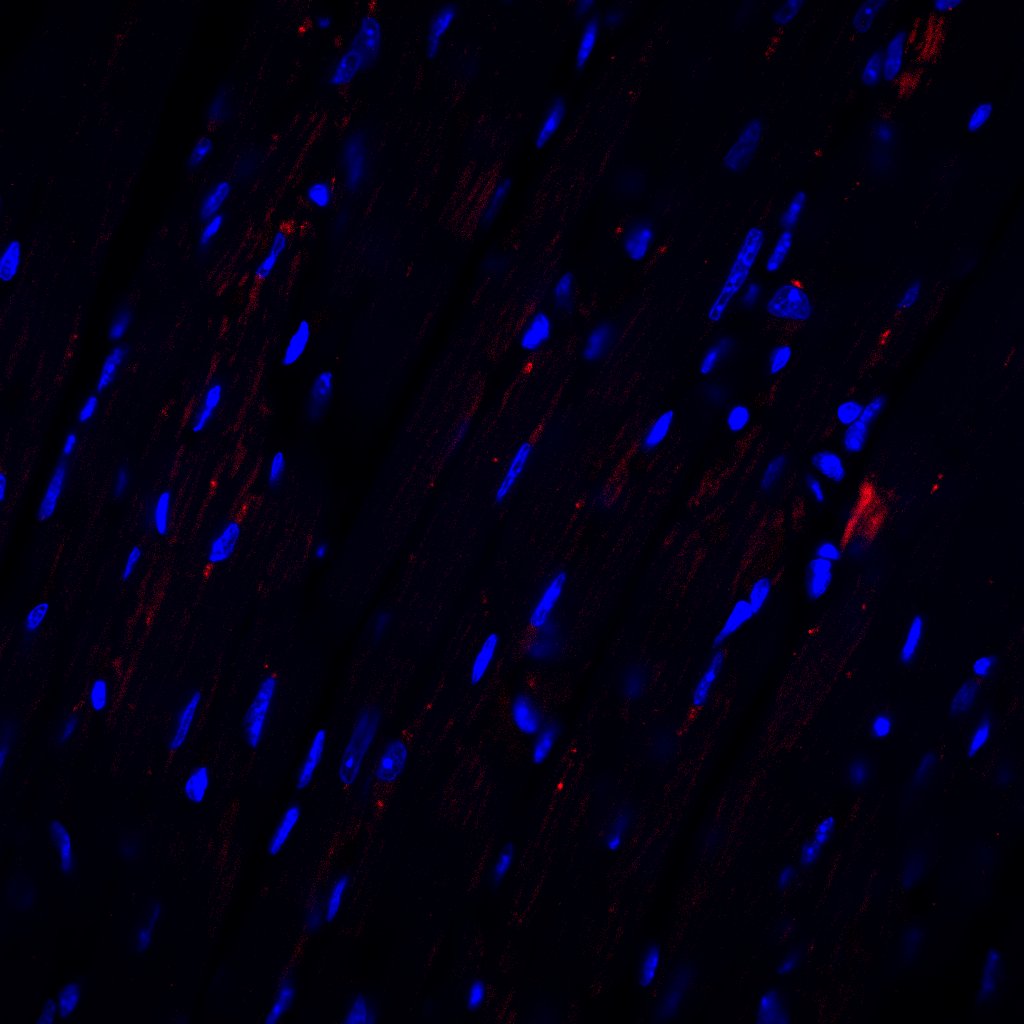

Supplement: Figure 6—source data 3. [file elife-98372-fig6-data3.zip › Figure 6-Source Data 3/Figure_6-source_data_1_Figure_6C_Heart_WT_ABT-199_cl-CASP3(Merge).jpg]

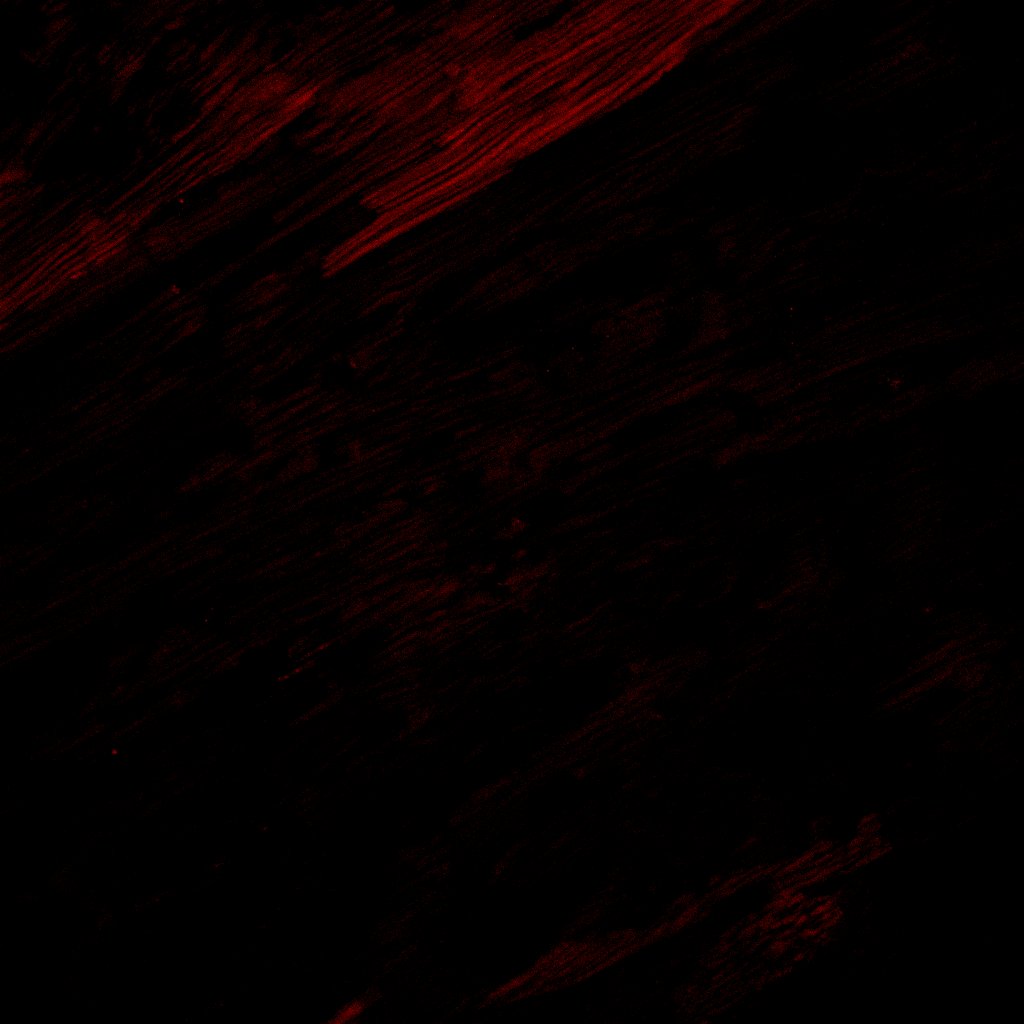

Supplement: Figure 6—source data 3. [file elife-98372-fig6-data3.zip › Figure 6-Source Data 3/Figure_6-source_data_1_Figure_6C_Heart_WT_cl-CASP3(cl-CASP3).jpg]

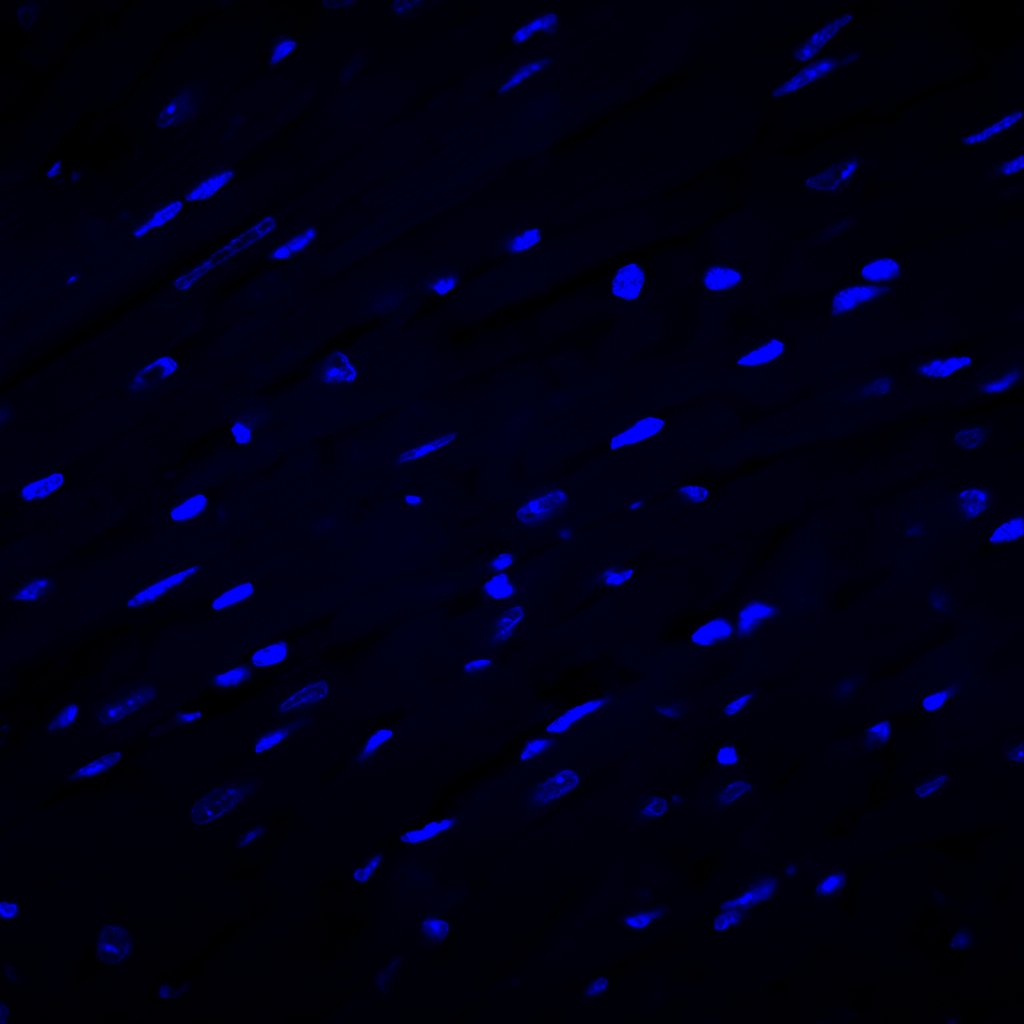

Supplement: Figure 6—source data 3. [file elife-98372-fig6-data3.zip › Figure 6-Source Data 3/Figure_6-source_data_1_Figure_6C_Heart_WT_cl-CASP3(DAPI).jpg]

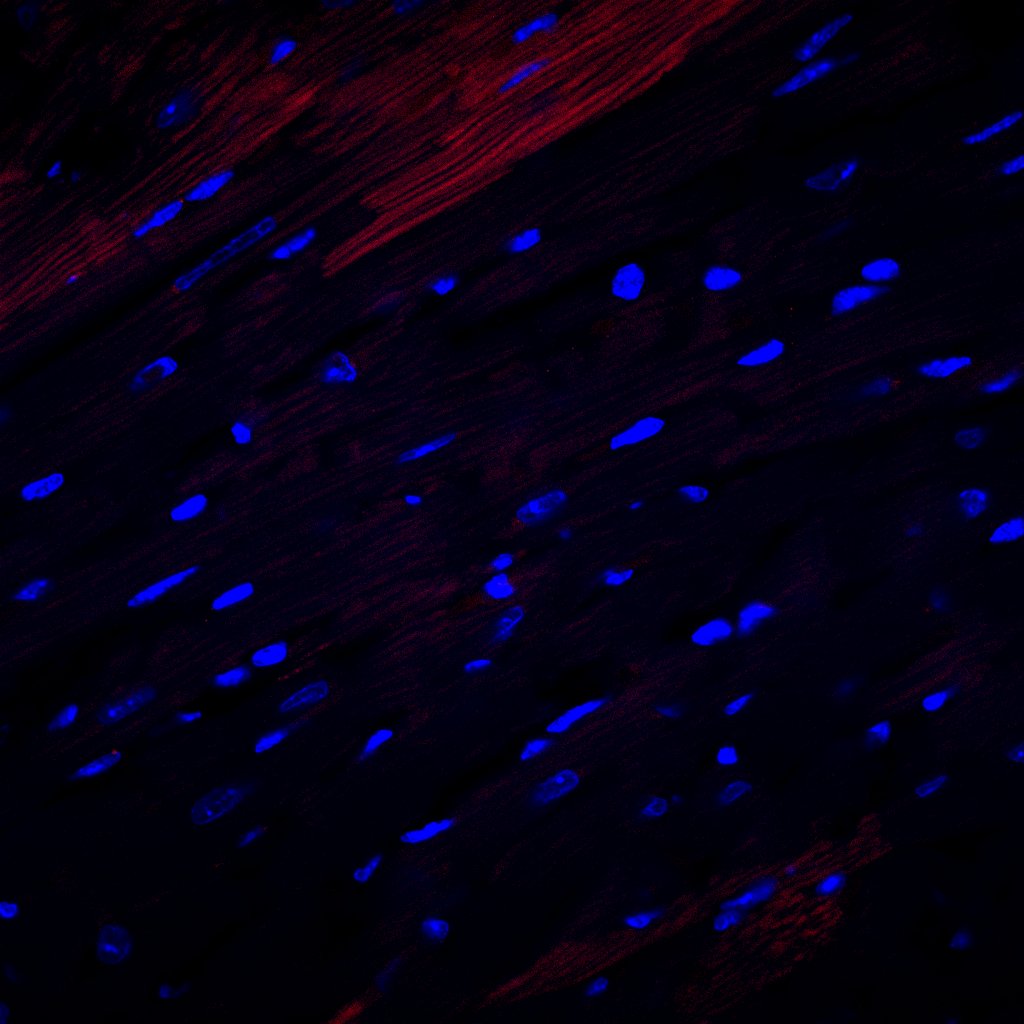

Supplement: Figure 6—source data 3. [file elife-98372-fig6-data3.zip › Figure 6-Source Data 3/Figure_6-source_data_1_Figure_6C_Heart_WT_cl-CASP3(Merge).jpg]

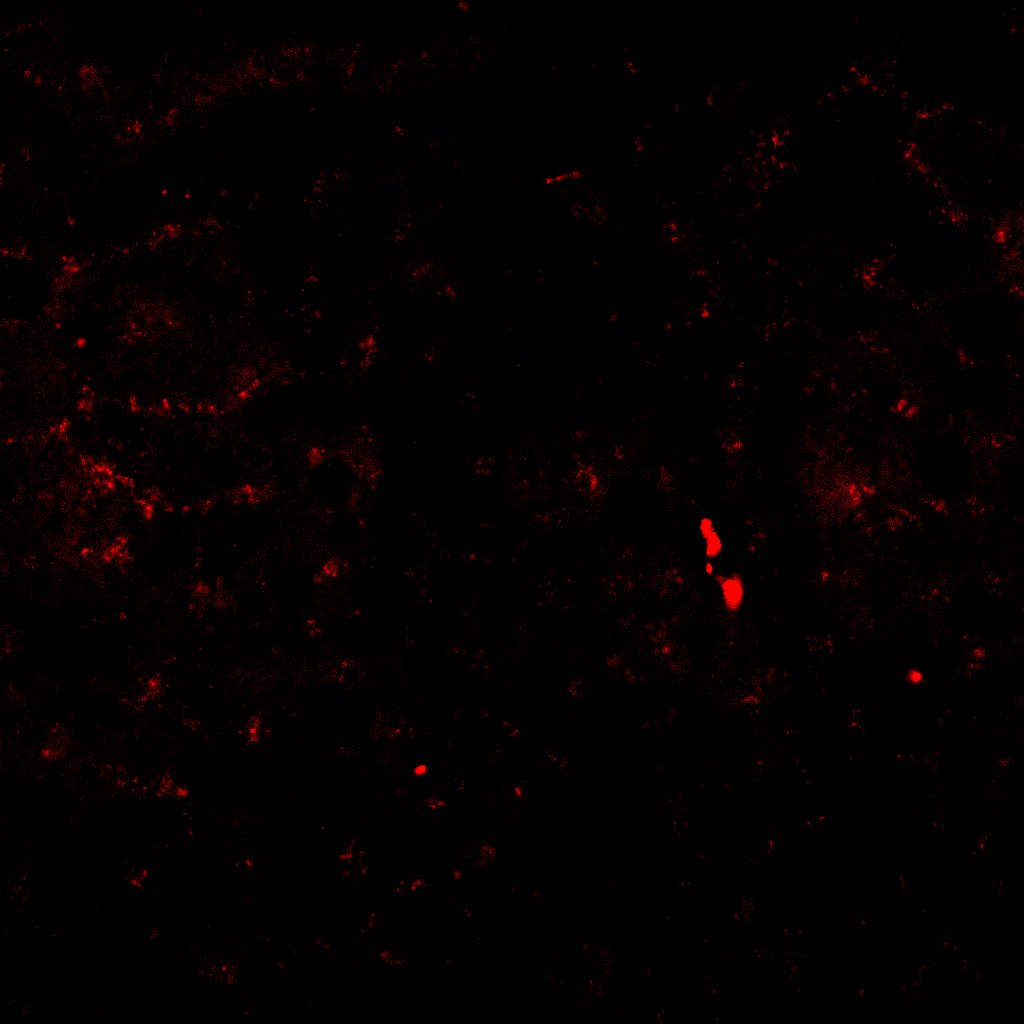

Supplement: Figure 6—source data 3. [file elife-98372-fig6-data3.zip › Figure 6-Source Data 3/Figure_6-source_data_1_Figure_6C_Liver_homo_ABT-199_cl-CASP3(cl-CASP3).jpg]

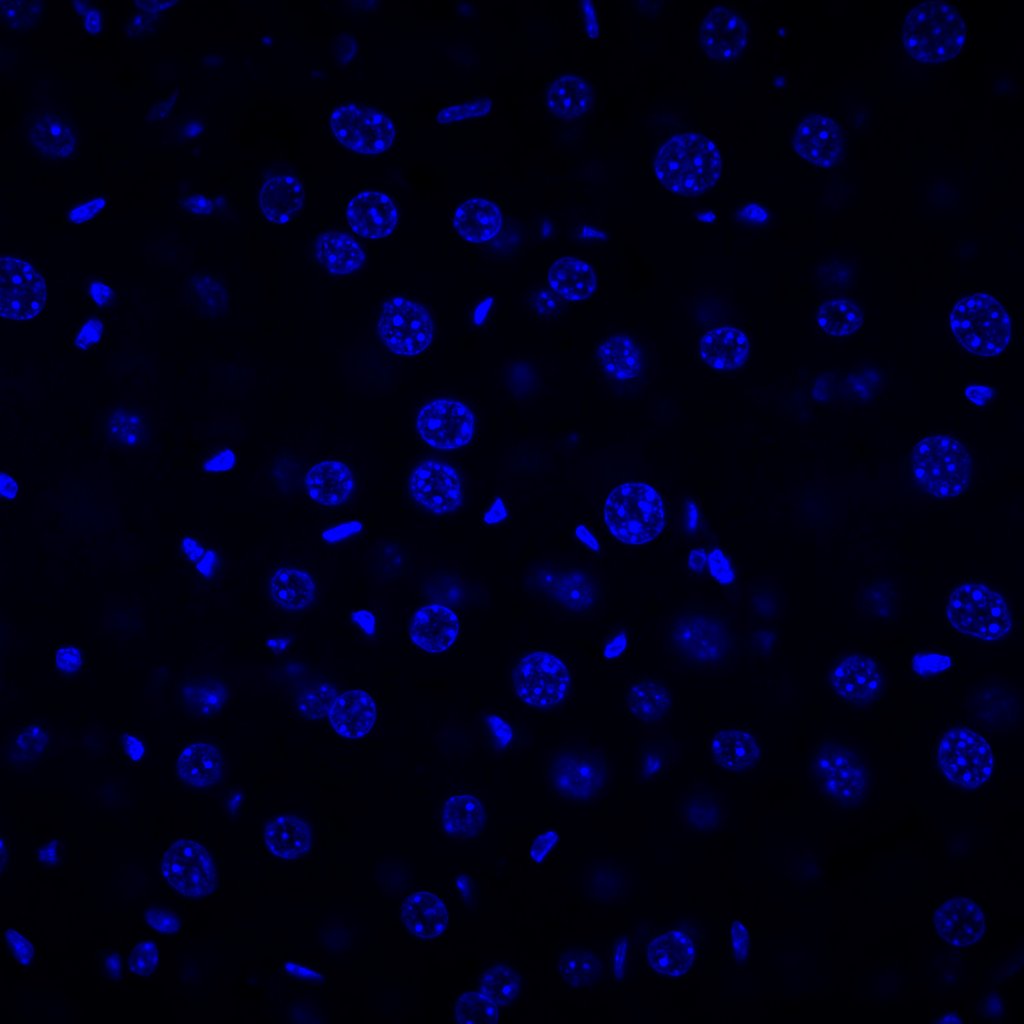

Supplement: Figure 6—source data 3. [file elife-98372-fig6-data3.zip › Figure 6-Source Data 3/Figure_6-source_data_1_Figure_6C_Liver_homo_ABT-199_cl-CASP3(DAPI).jpg]

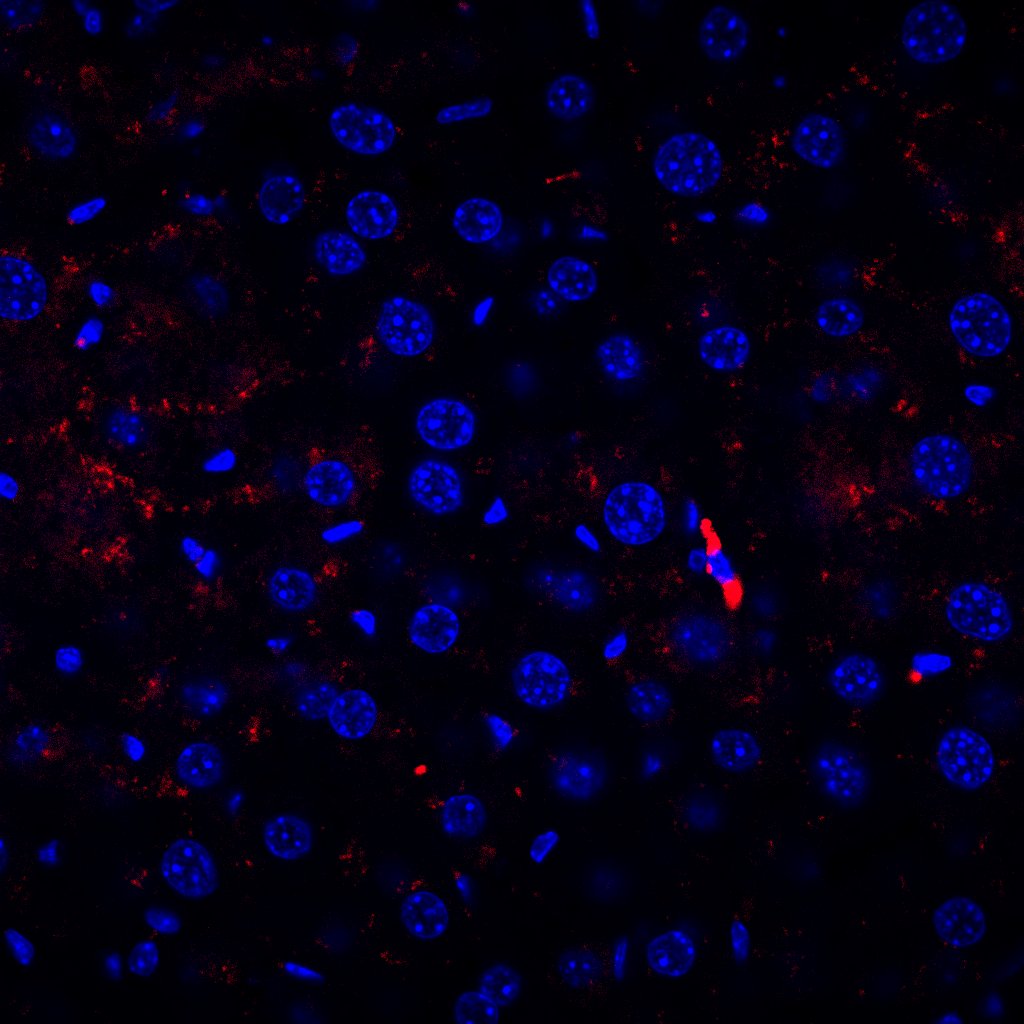

Supplement: Figure 6—source data 3. [file elife-98372-fig6-data3.zip › Figure 6-Source Data 3/Figure_6-source_data_1_Figure_6C_Liver_homo_ABT-199_cl-CASP3(Merge).jpg]

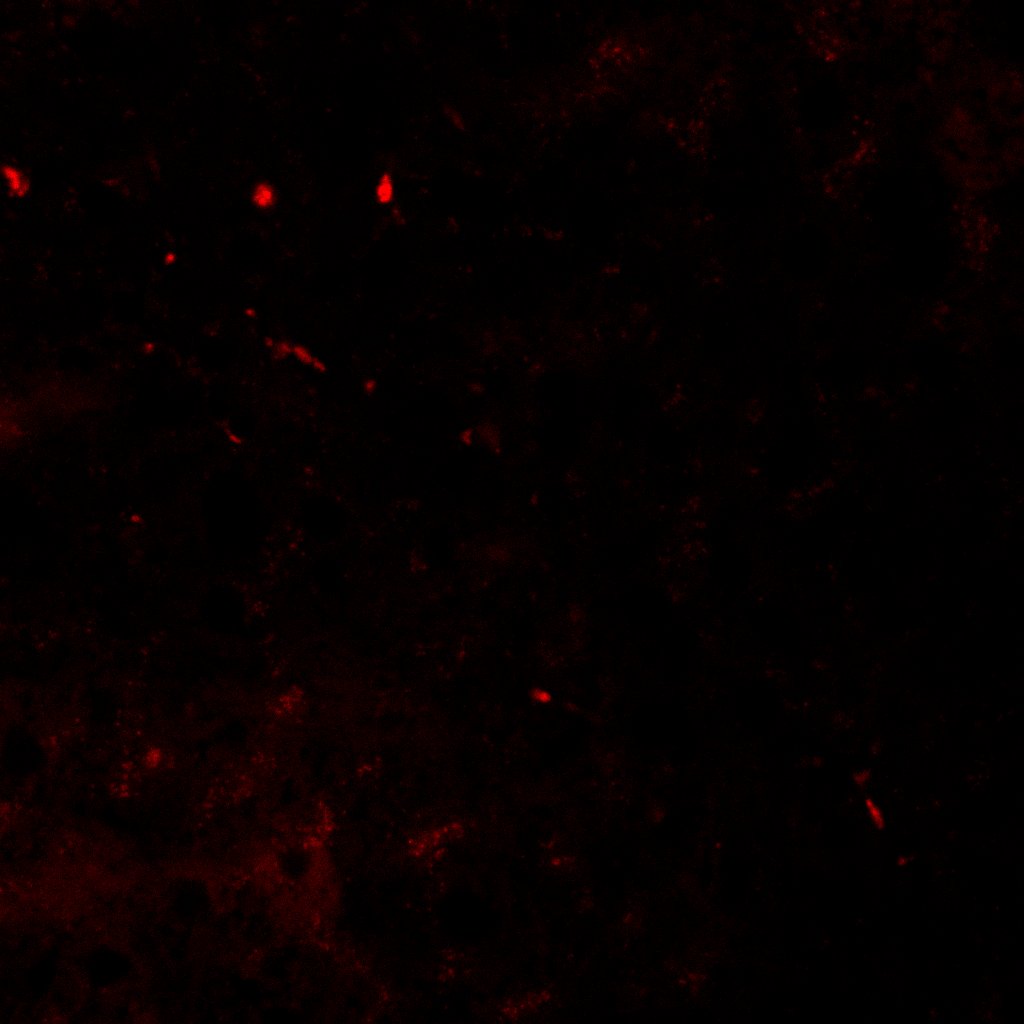

Supplement: Figure 6—source data 3. [file elife-98372-fig6-data3.zip › Figure 6-Source Data 3/Figure_6-source_data_1_Figure_6C_Liver_homo_cl-CASP3(cl-CASP3).jpg]

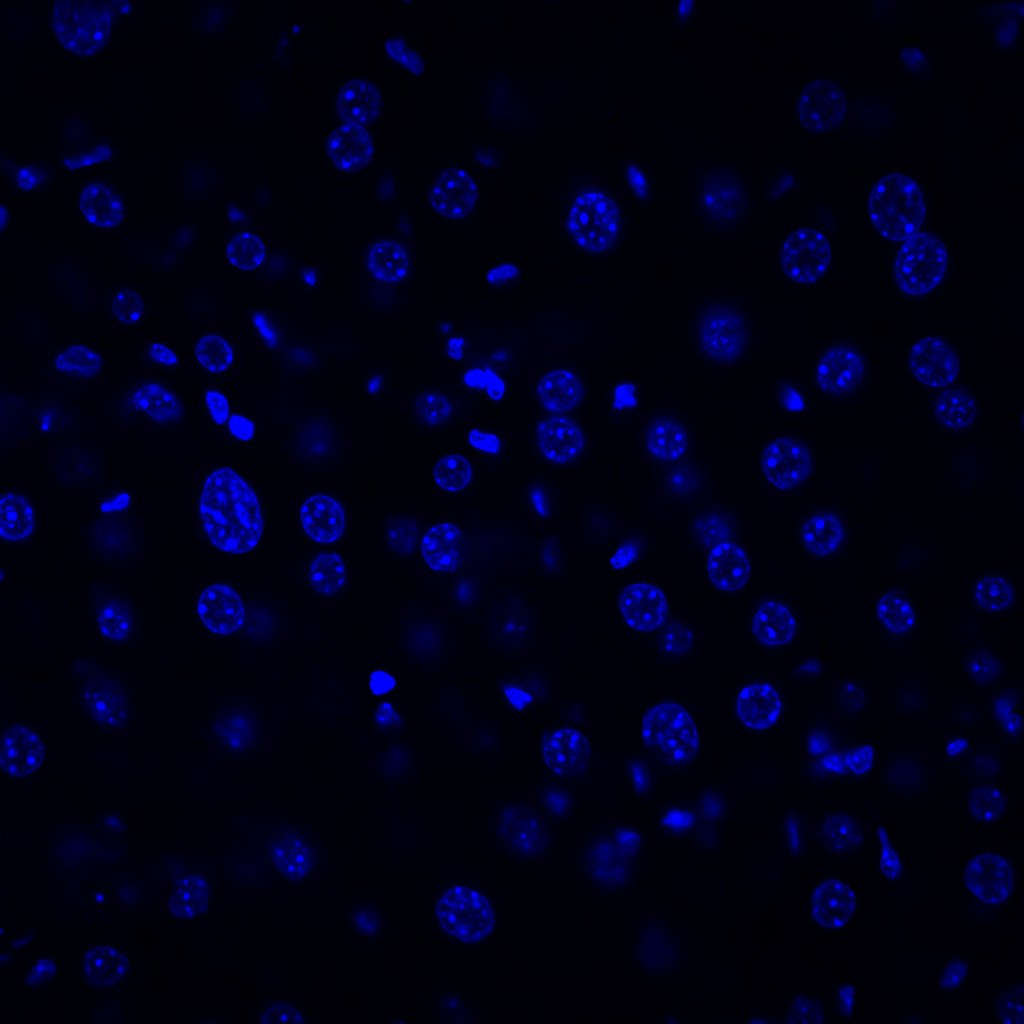

Supplement: Figure 6—source data 3. [file elife-98372-fig6-data3.zip › Figure 6-Source Data 3/Figure_6-source_data_1_Figure_6C_Liver_homo_cl-CASP3(DAPI).jpg]

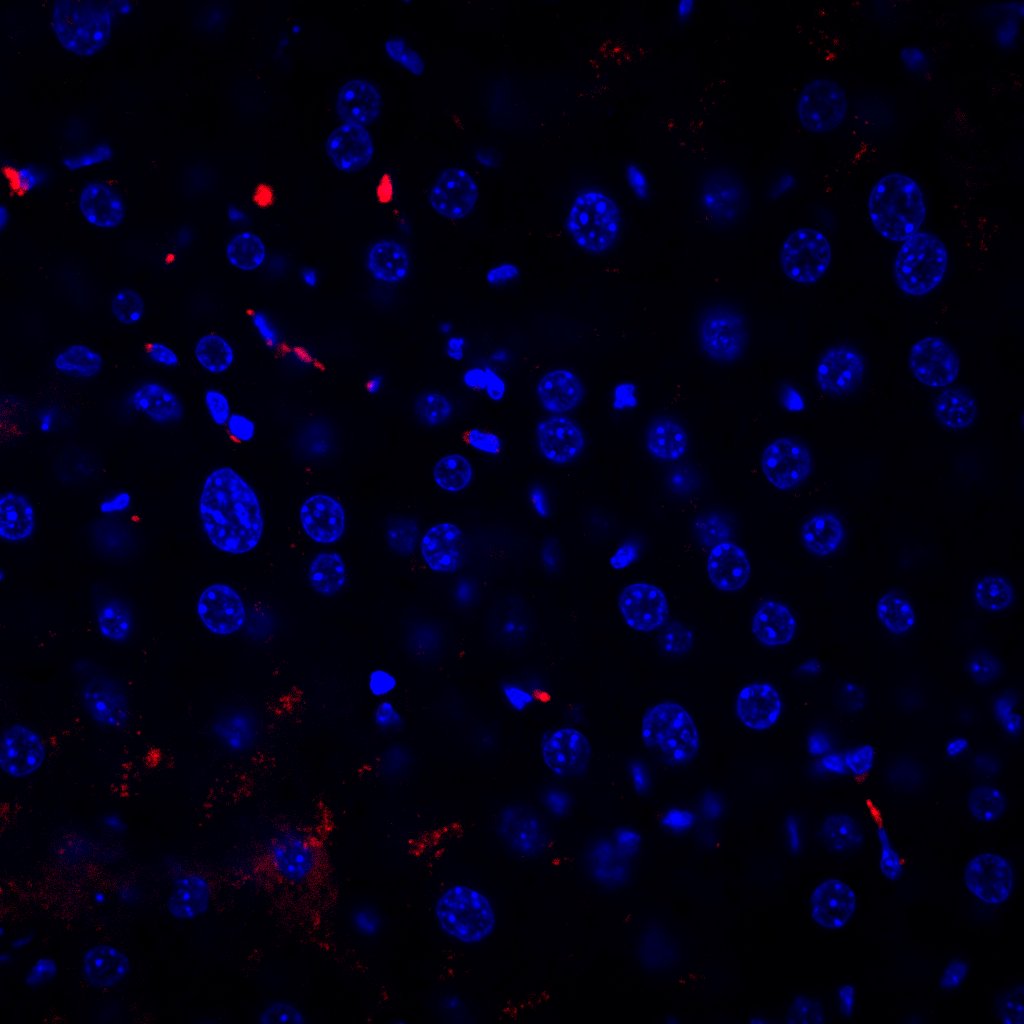

Supplement: Figure 6—source data 3. [file elife-98372-fig6-data3.zip › Figure 6-Source Data 3/Figure_6-source_data_1_Figure_6C_Liver_homo_cl-CASP3(Merge).jpg]

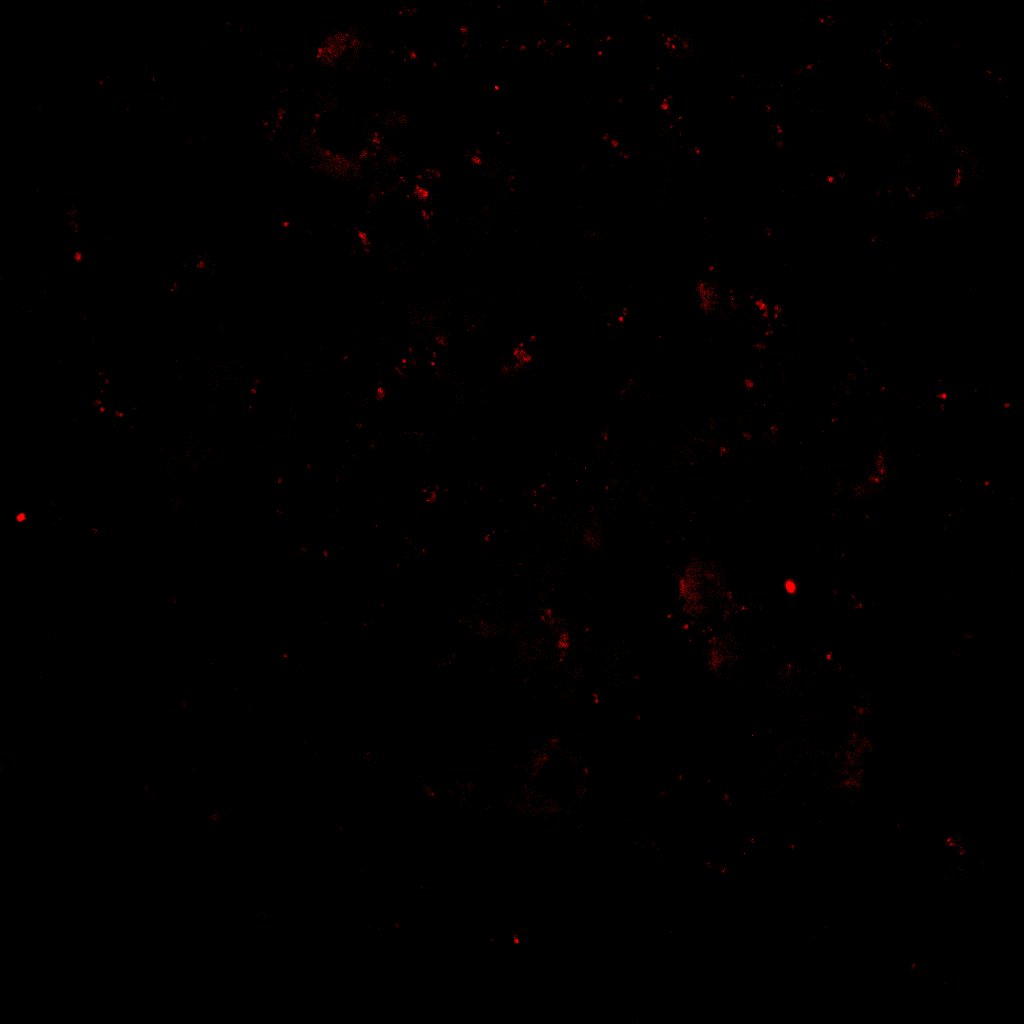

Supplement: Figure 6—source data 3. [file elife-98372-fig6-data3.zip › Figure 6-Source Data 3/Figure_6-source_data_1_Figure_6C_Liver_WT_ABT-199_cl-CASP3(cl-CASP3).jpg]

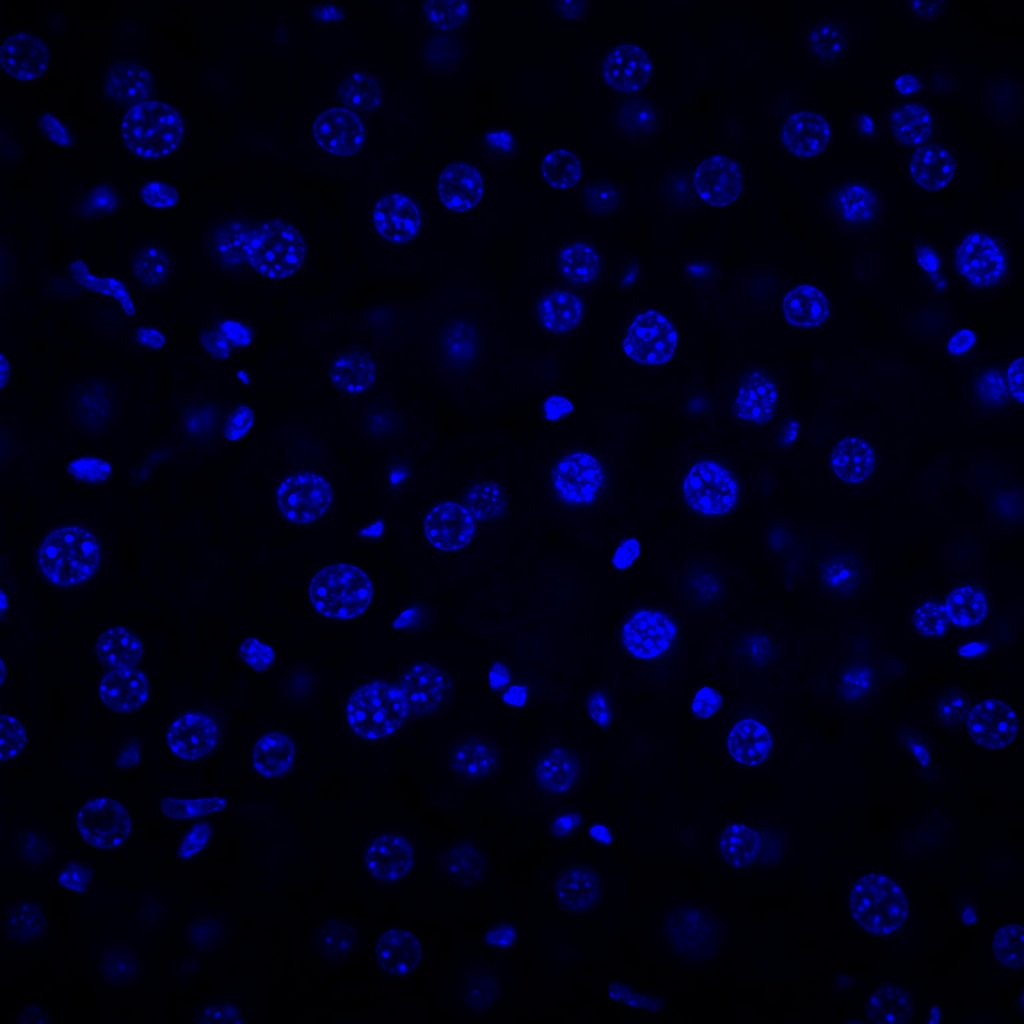

Supplement: Figure 6—source data 3. [file elife-98372-fig6-data3.zip › Figure 6-Source Data 3/Figure_6-source_data_1_Figure_6C_Liver_WT_ABT-199_cl-CASP3(DAPI).jpg]

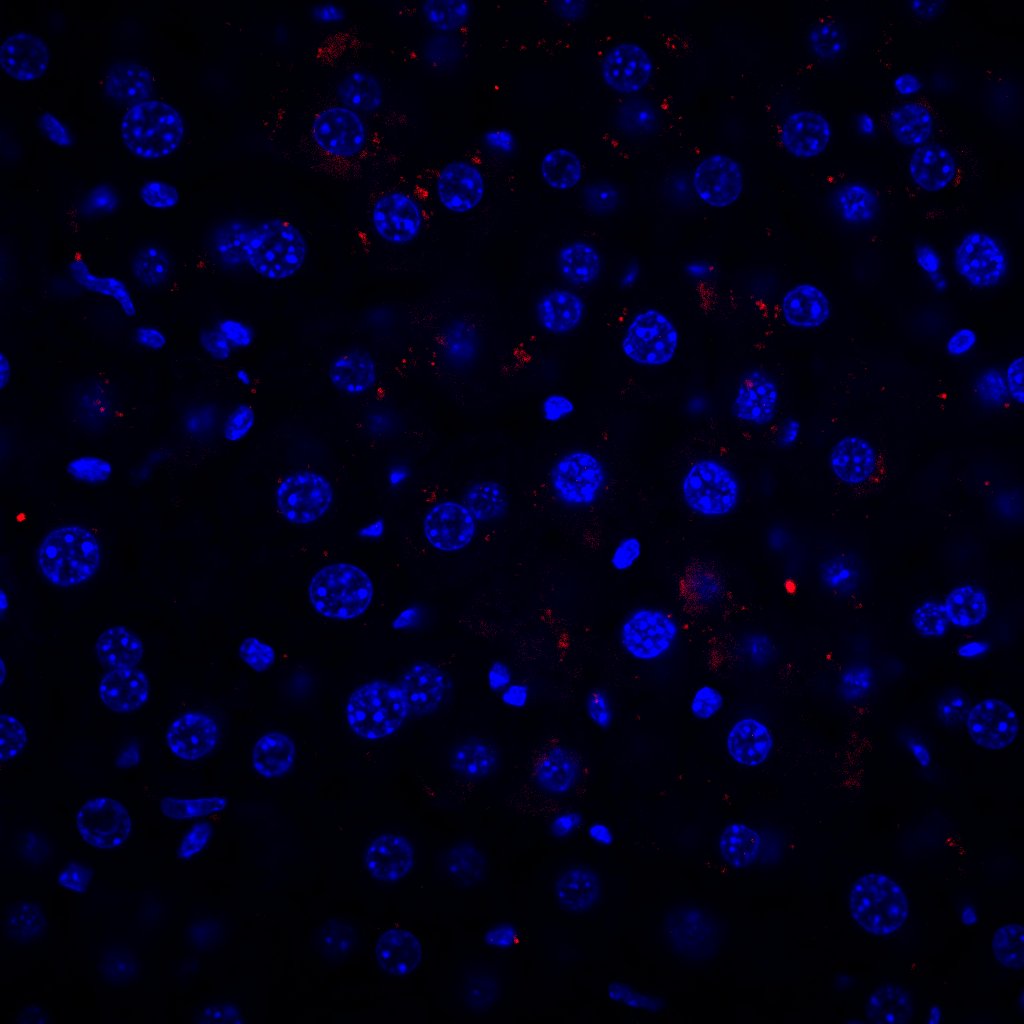

Supplement: Figure 6—source data 3. [file elife-98372-fig6-data3.zip › Figure 6-Source Data 3/Figure_6-source_data_1_Figure_6C_Liver_WT_ABT-199_cl-CASP3(Merge).jpg]

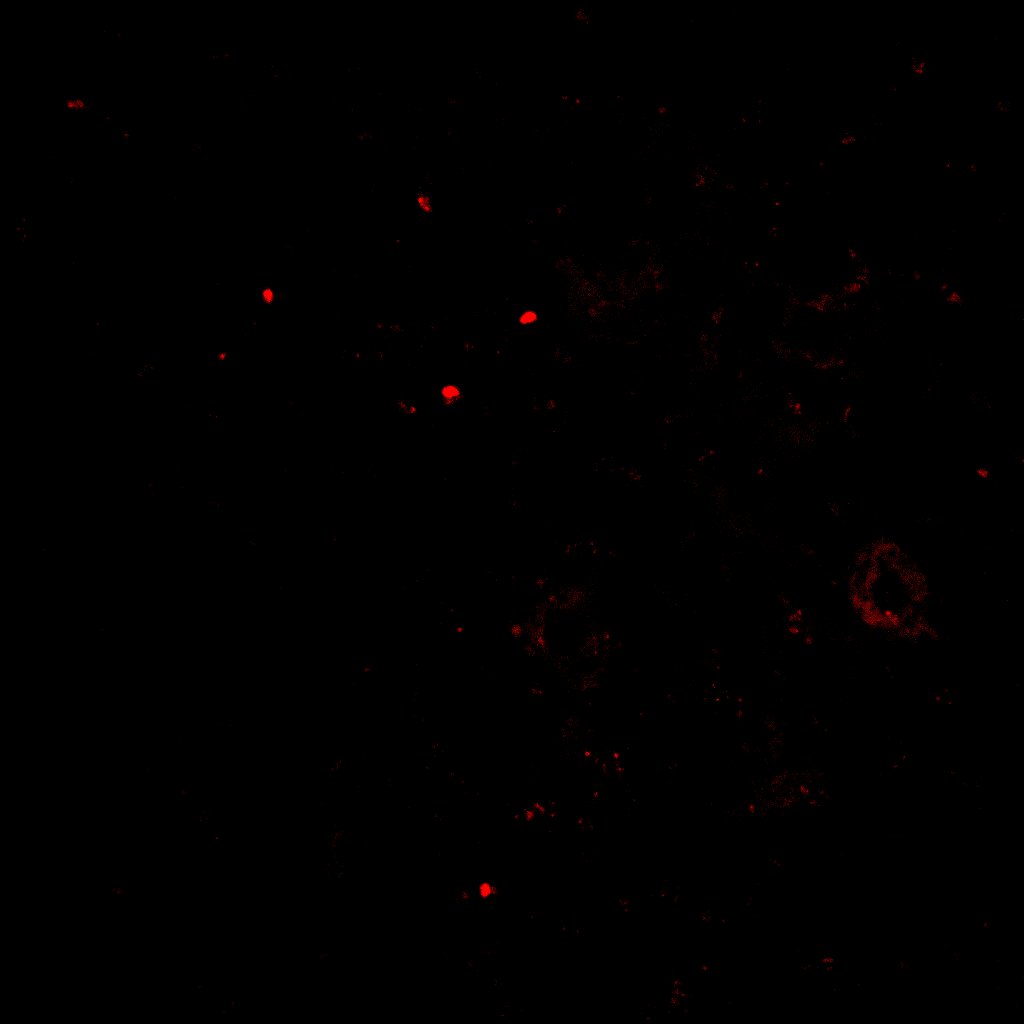

Supplement: Figure 6—source data 3. [file elife-98372-fig6-data3.zip › Figure 6-Source Data 3/Figure_6-source_data_1_Figure_6C_Liver_WT_cl-CASP3(cl-CASP3).jpg]

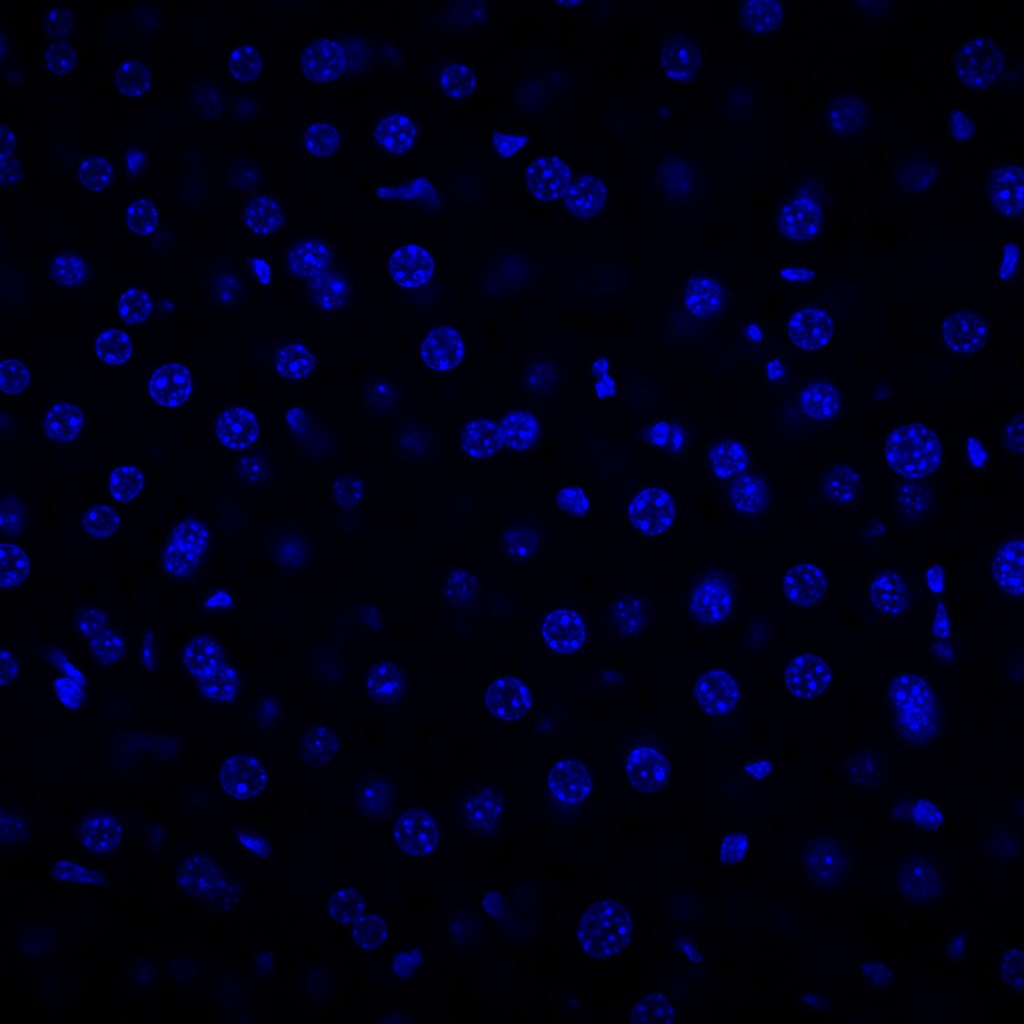

Supplement: Figure 6—source data 3. [file elife-98372-fig6-data3.zip › Figure 6-Source Data 3/Figure_6-source_data_1_Figure_6C_Liver_WT_cl-CASP3(DAPI).jpg]

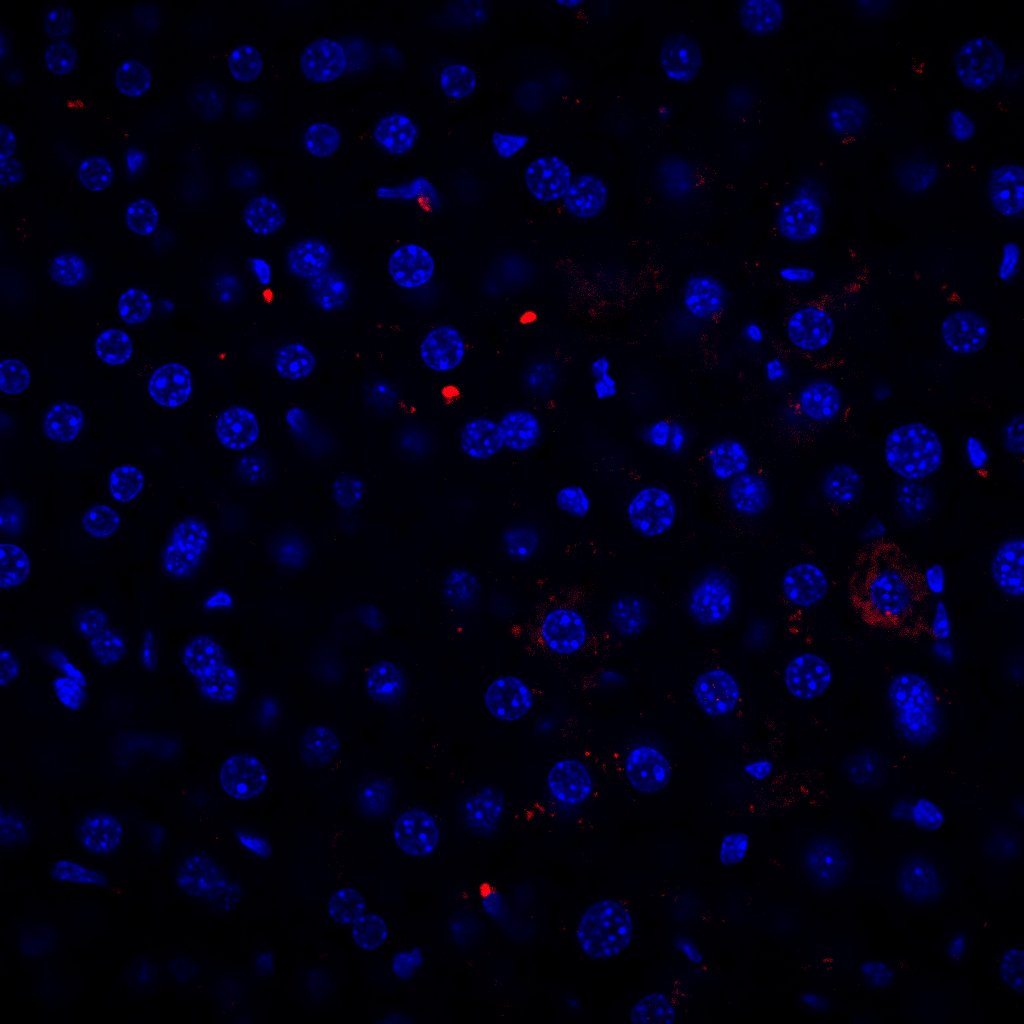

Supplement: Figure 6—source data 3. [file elife-98372-fig6-data3.zip › Figure 6-Source Data 3/Figure_6-source_data_1_Figure_6C_Liver_WT_cl-CASP3(Merge).jpg]

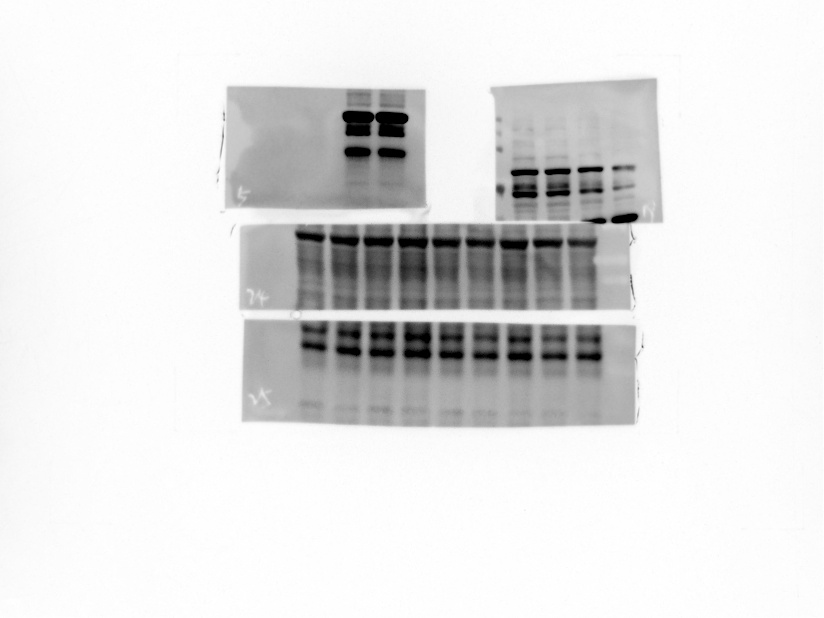

Supplement: Figure 6—figure supplement 1—source data 1. [file elife-98372-fig6-figsupp1-data1.zip › Figure 6-supplementary figure 1-data1/Figure_6-figure supplement_1_ source_data_1_ Figure_D_CASP3(Brain).jpg]

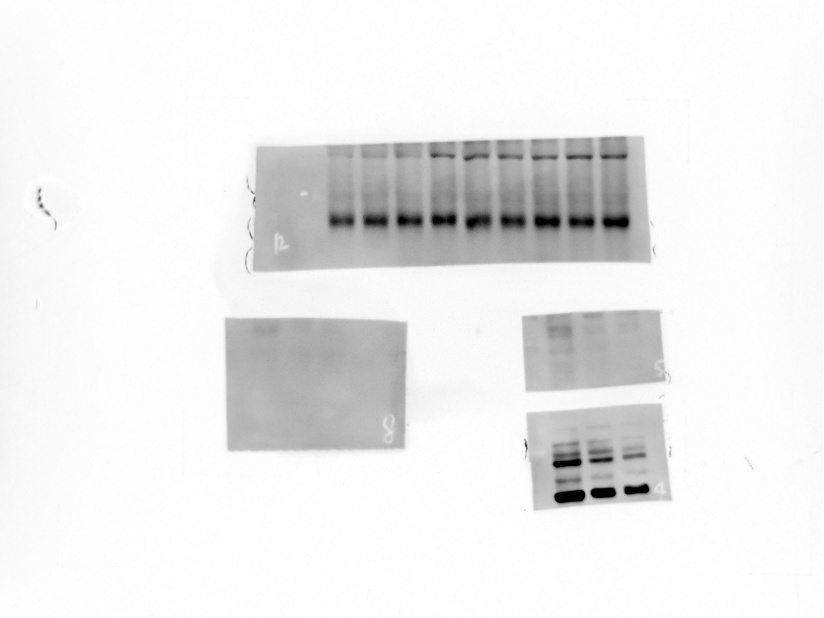

Supplement: Figure 6—figure supplement 1—source data 1. [file elife-98372-fig6-figsupp1-data1.zip › Figure 6-supplementary figure 1-data1/Figure_6-figure supplement_1_ source_data_1_ Figure_D_CASP3(Heart).jpg]

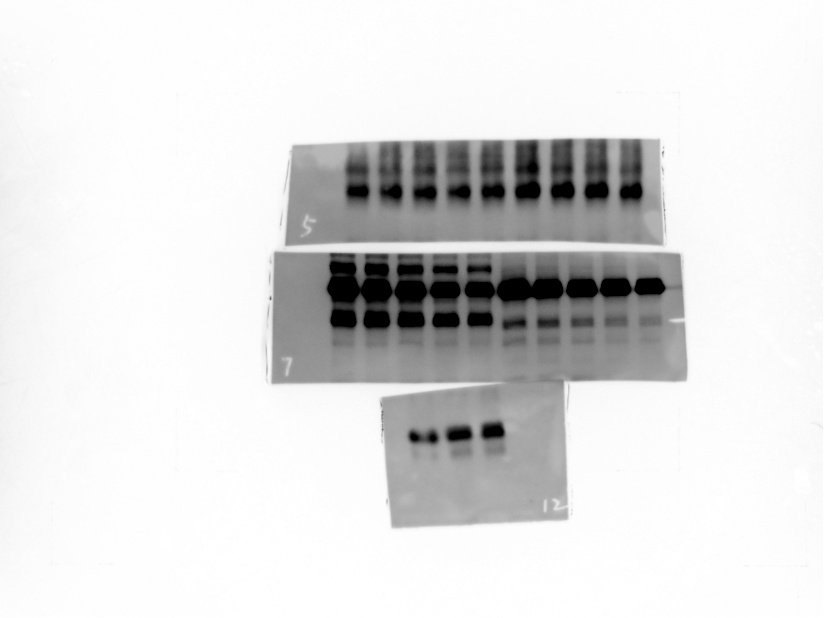

Supplement: Figure 6—figure supplement 1—source data 1. [file elife-98372-fig6-figsupp1-data1.zip › Figure 6-supplementary figure 1-data1/Figure_6-figure supplement_1_ source_data_1_ Figure_D_CASP3(Kidney).jpg]

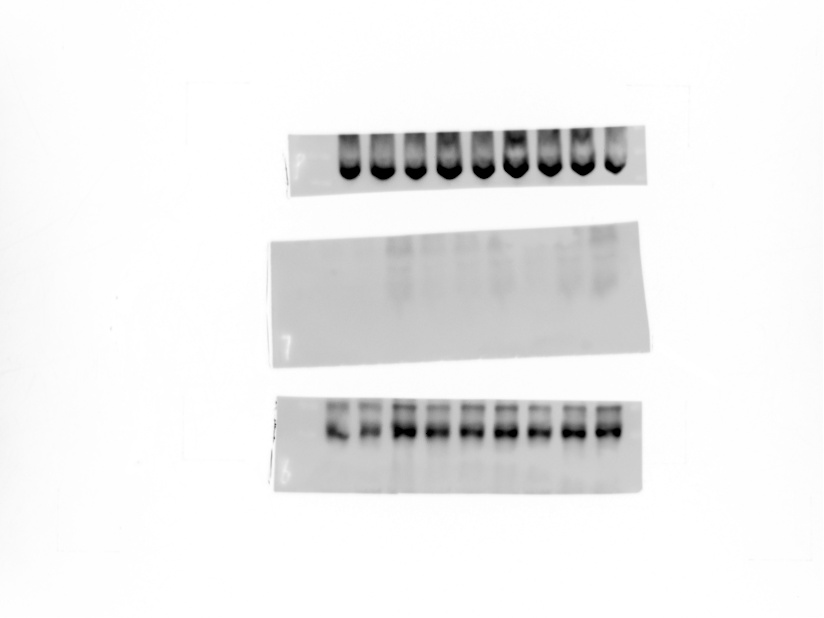

Supplement: Figure 6—figure supplement 1—source data 1. [file elife-98372-fig6-figsupp1-data1.zip › Figure 6-supplementary figure 1-data1/Figure_6-figure supplement_1_ source_data_1_ Figure_D_CASP3(Liver).jpg]

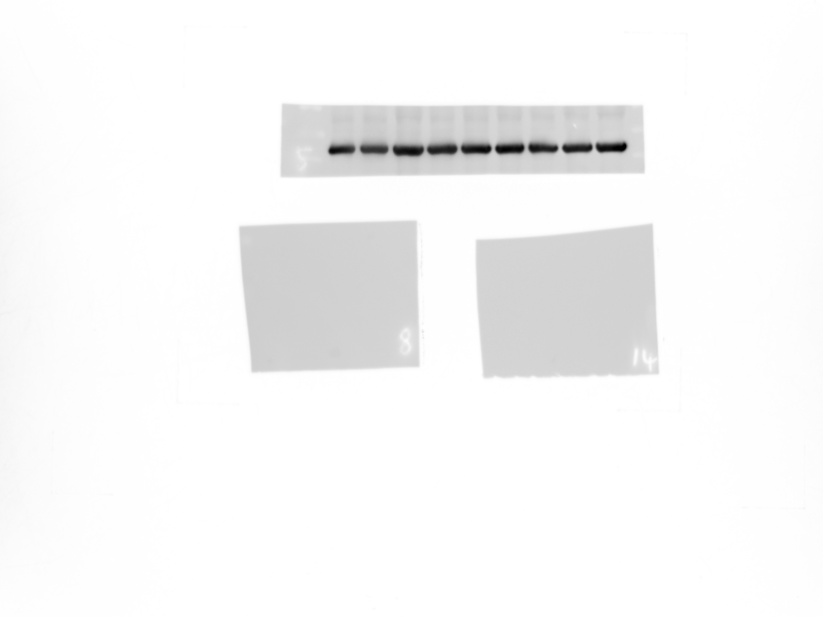

Supplement: Figure 6—figure supplement 1—source data 1. [file elife-98372-fig6-figsupp1-data1.zip › Figure 6-supplementary figure 1-data1/Figure_6-figure supplement_1_ source_data_1_ Figure_D_CASP3(Lung).jpg]

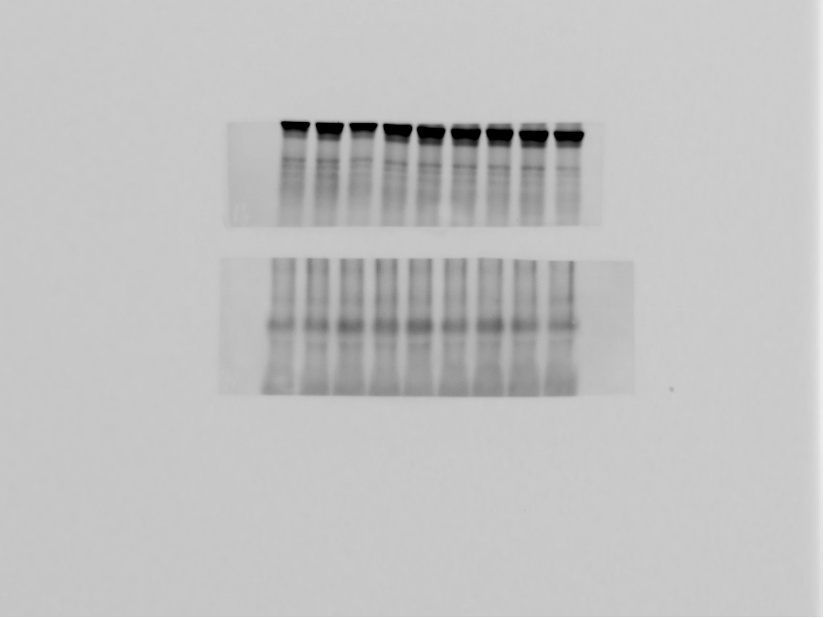

Supplement: Figure 6—figure supplement 1—source data 1. [file elife-98372-fig6-figsupp1-data1.zip › Figure 6-supplementary figure 1-data1/Figure_6-figure supplement_1_ source_data_1_ Figure_D_CASP7(Brain).jpg]

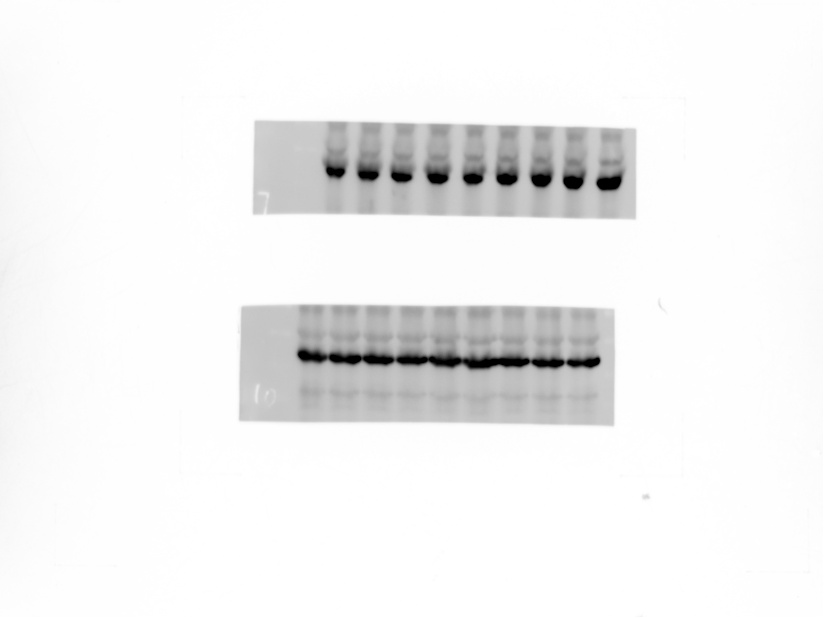

Supplement: Figure 6—figure supplement 1—source data 1. [file elife-98372-fig6-figsupp1-data1.zip › Figure 6-supplementary figure 1-data1/Figure_6-figure supplement_1_ source_data_1_ Figure_D_CASP7(Heart).jpg]

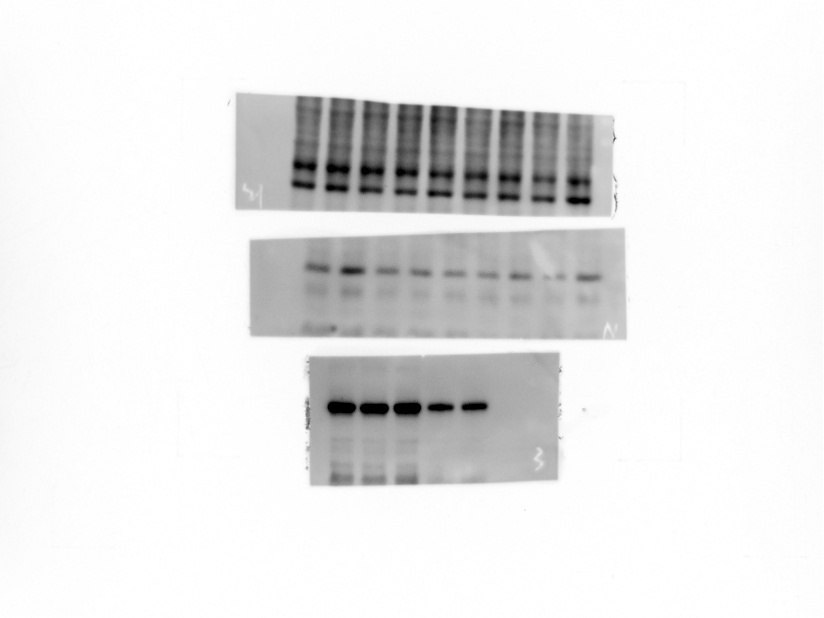

Supplement: Figure 6—figure supplement 1—source data 1. [file elife-98372-fig6-figsupp1-data1.zip › Figure 6-supplementary figure 1-data1/Figure_6-figure supplement_1_ source_data_1_ Figure_D_CASP7(Kidney).jpg]

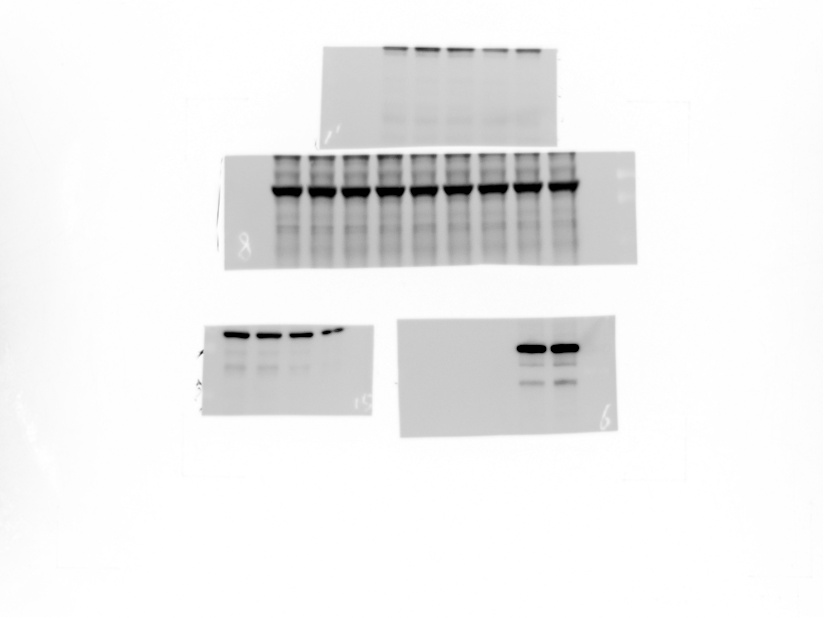

Supplement: Figure 6—figure supplement 1—source data 1. [file elife-98372-fig6-figsupp1-data1.zip › Figure 6-supplementary figure 1-data1/Figure_6-figure supplement_1_ source_data_1_ Figure_D_CASP7(Liver).jpg]

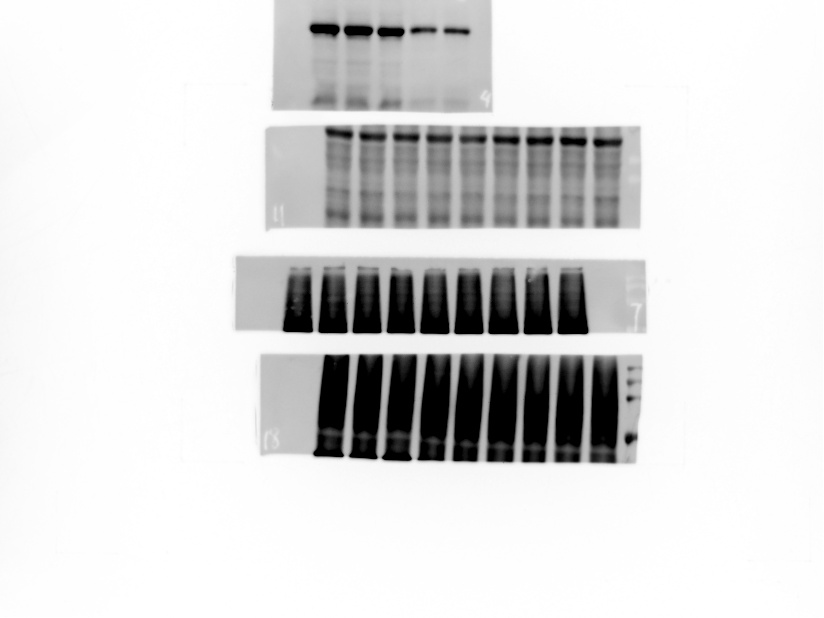

Supplement: Figure 6—figure supplement 1—source data 1. [file elife-98372-fig6-figsupp1-data1.zip › Figure 6-supplementary figure 1-data1/Figure_6-figure supplement_1_ source_data_1_ Figure_D_CASP7(Lung).jpg]

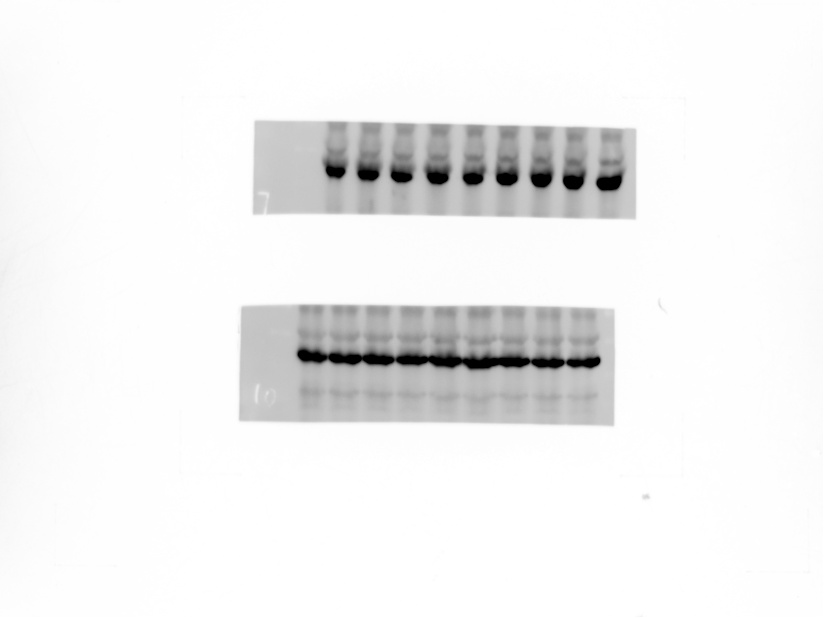

Supplement: Figure 6—figure supplement 1—source data 1. [file elife-98372-fig6-figsupp1-data1.zip › Figure 6-supplementary figure 1-data1/Figure_6-figure supplement_1_ source_data_1_ Figure_D_CASP9(Heart).jpg]

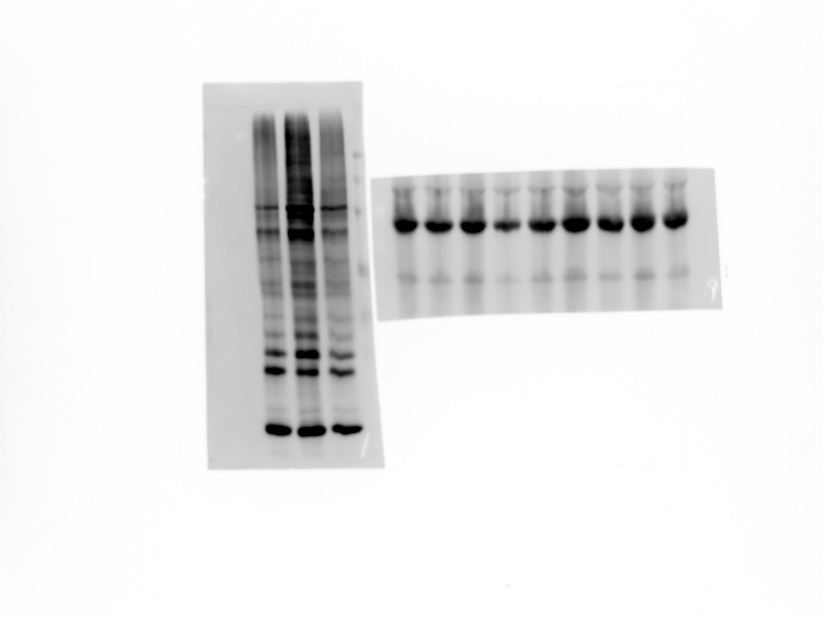

Supplement: Figure 6—figure supplement 1—source data 1. [file elife-98372-fig6-figsupp1-data1.zip › Figure 6-supplementary figure 1-data1/Figure_6-figure supplement_1_ source_data_1_ Figure_D_CASP9(Kidney).jpg]

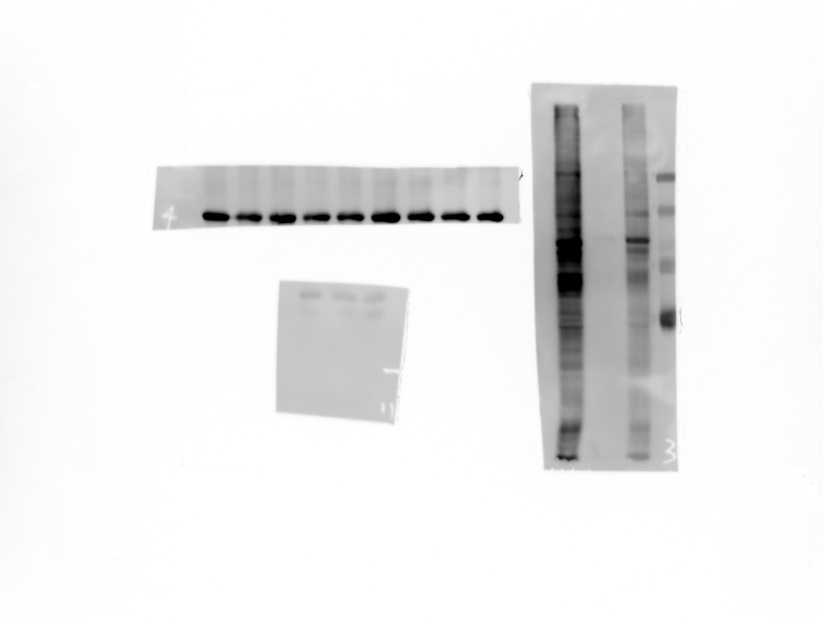

Supplement: Figure 6—figure supplement 1—source data 1. [file elife-98372-fig6-figsupp1-data1.zip › Figure 6-supplementary figure 1-data1/Figure_6-figure supplement_1_ source_data_1_ Figure_D_CASP9(Lung).jpg]

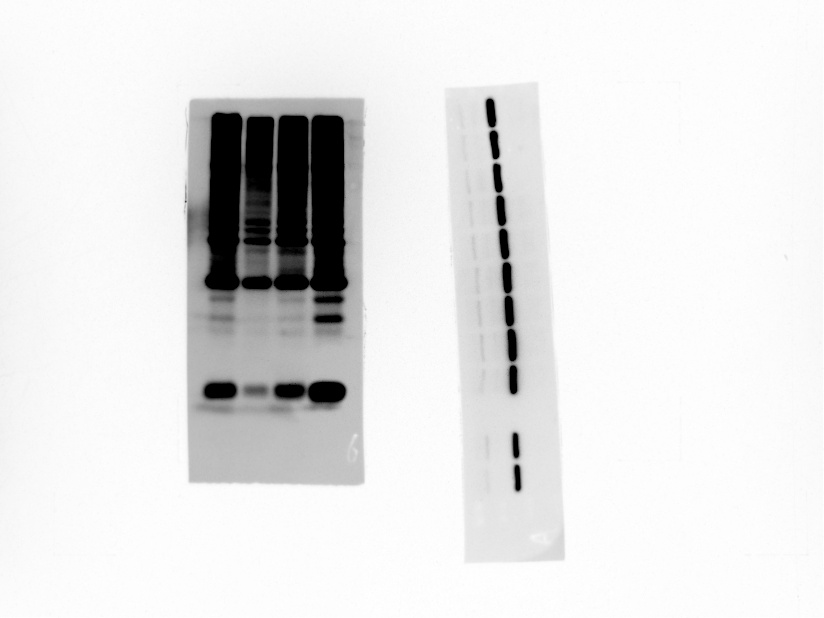

Supplement: Figure 6—figure supplement 1—source data 1. [file elife-98372-fig6-figsupp1-data1.zip › Figure 6-supplementary figure 1-data1/Figure_6-figure supplement_1_ source_data_1_ Figure_D_GAPDH(Brain).jpg]

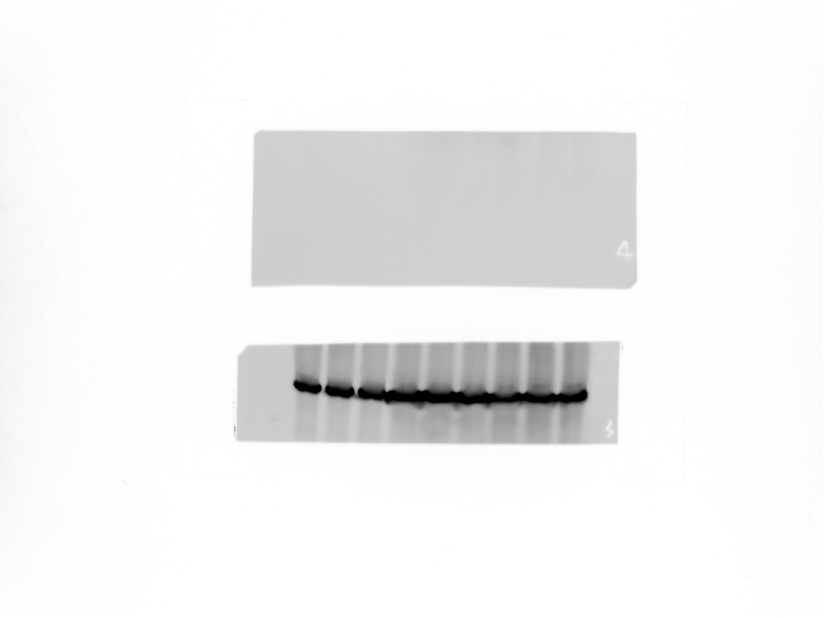

Supplement: Figure 6—figure supplement 1—source data 1. [file elife-98372-fig6-figsupp1-data1.zip › Figure 6-supplementary figure 1-data1/Figure_6-figure supplement_1_ source_data_1_ Figure_D_GAPDH(Heart).jpg]

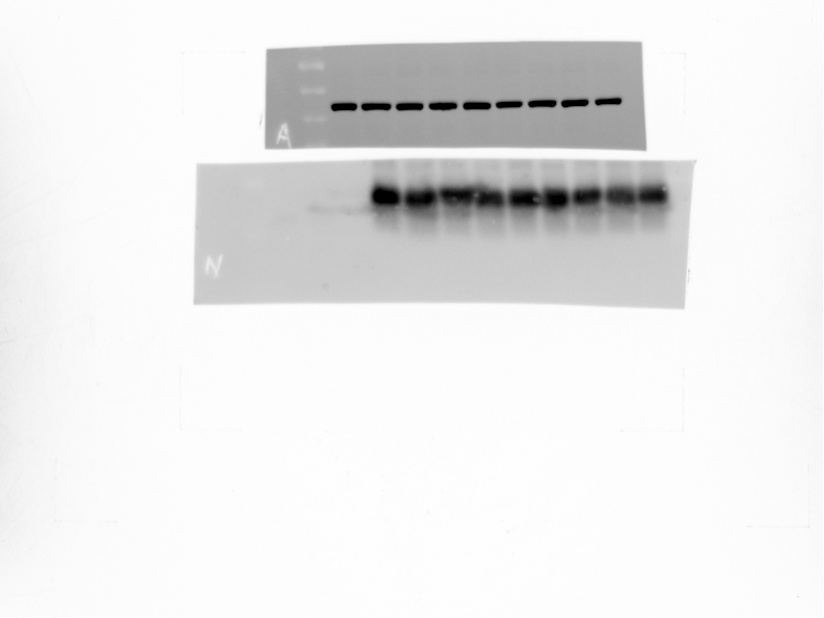

Supplement: Figure 6—figure supplement 1—source data 1. [file elife-98372-fig6-figsupp1-data1.zip › Figure 6-supplementary figure 1-data1/Figure_6-figure supplement_1_ source_data_1_ Figure_D_GAPDH(Kidney).jpg]

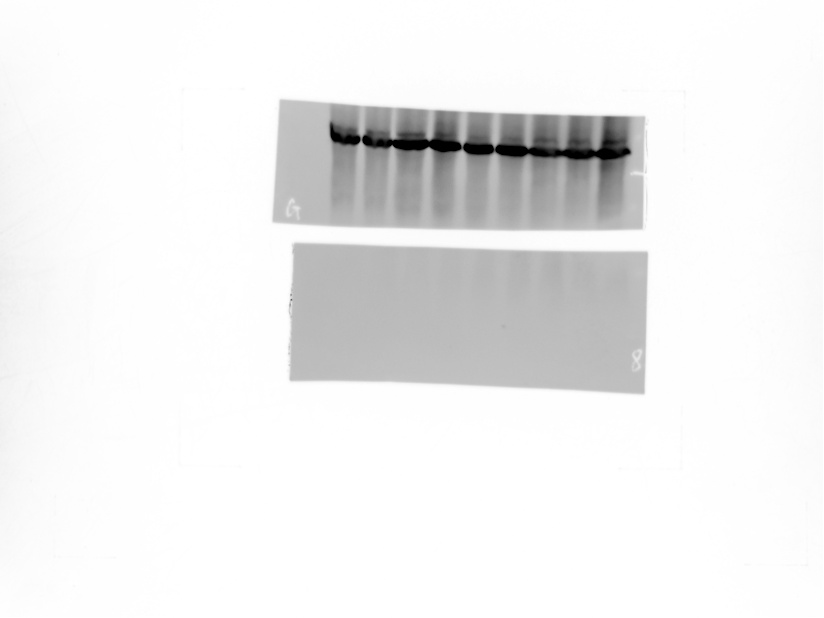

Supplement: Figure 6—figure supplement 1—source data 1. [file elife-98372-fig6-figsupp1-data1.zip › Figure 6-supplementary figure 1-data1/Figure_6-figure supplement_1_ source_data_1_ Figure_D_GAPDH(Liver).jpg]

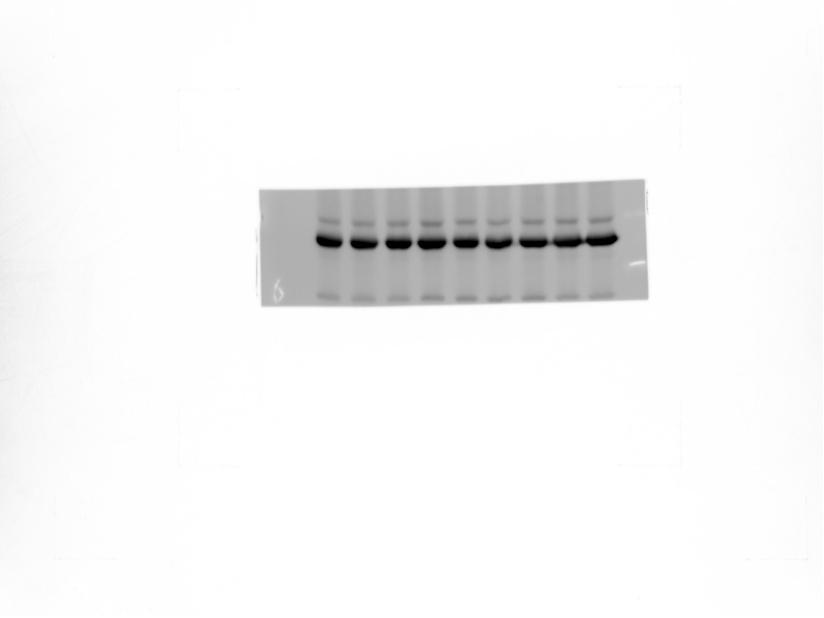

Supplement: Figure 6—figure supplement 1—source data 1. [file elife-98372-fig6-figsupp1-data1.zip › Figure 6-supplementary figure 1-data1/Figure_6-figure supplement_1_ source_data_1_ Figure_D_GAPDH(Lung).jpg]

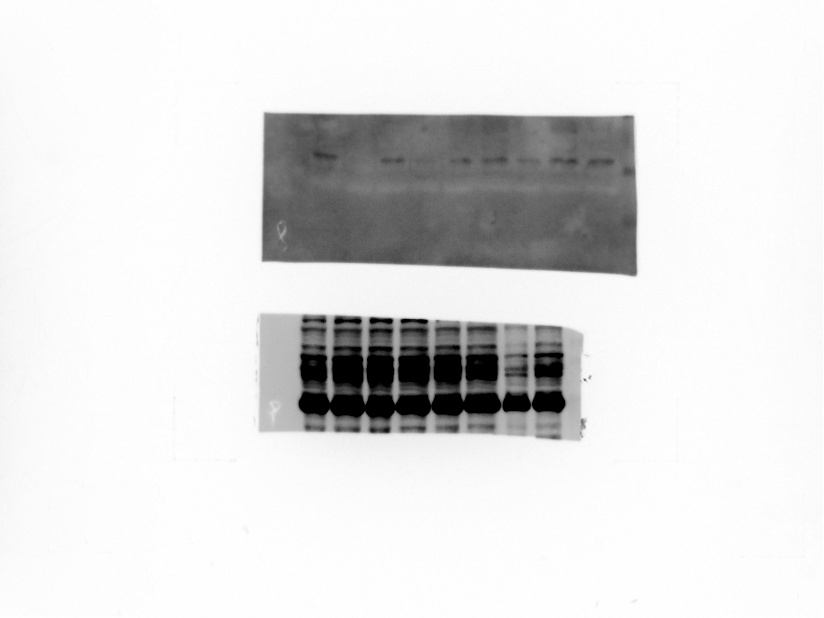

Supplement: Figure 6—figure supplement 1—source data 1. [file elife-98372-fig6-figsupp1-data1.zip › Figure 6-supplementary figure 1-data1/Figure_6-figure supplement_1_ source_data_1_ Figure_D_NOXA(Brain).jpg]

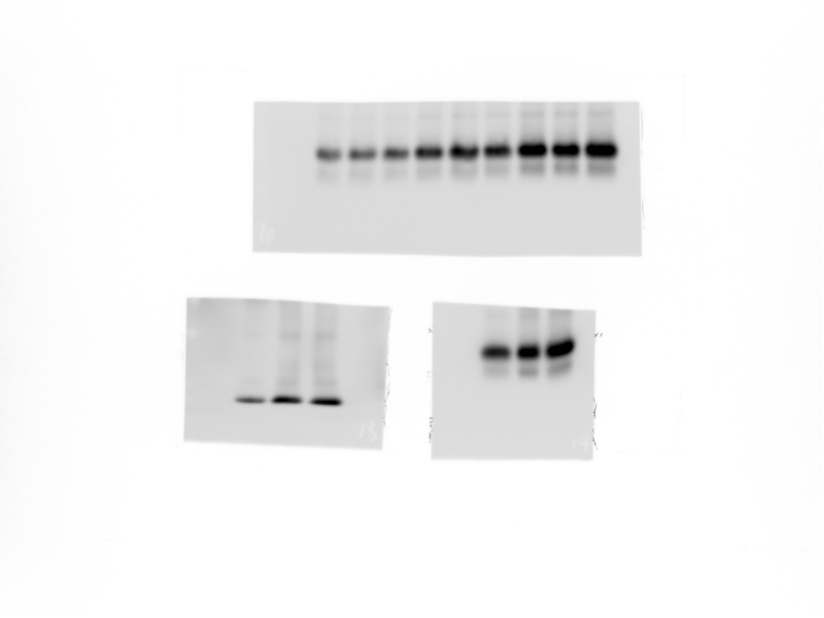

Supplement: Figure 6—figure supplement 1—source data 1. [file elife-98372-fig6-figsupp1-data1.zip › Figure 6-supplementary figure 1-data1/Figure_6-figure supplement_1_ source_data_1_ Figure_D_NOXA(Heart).jpg]

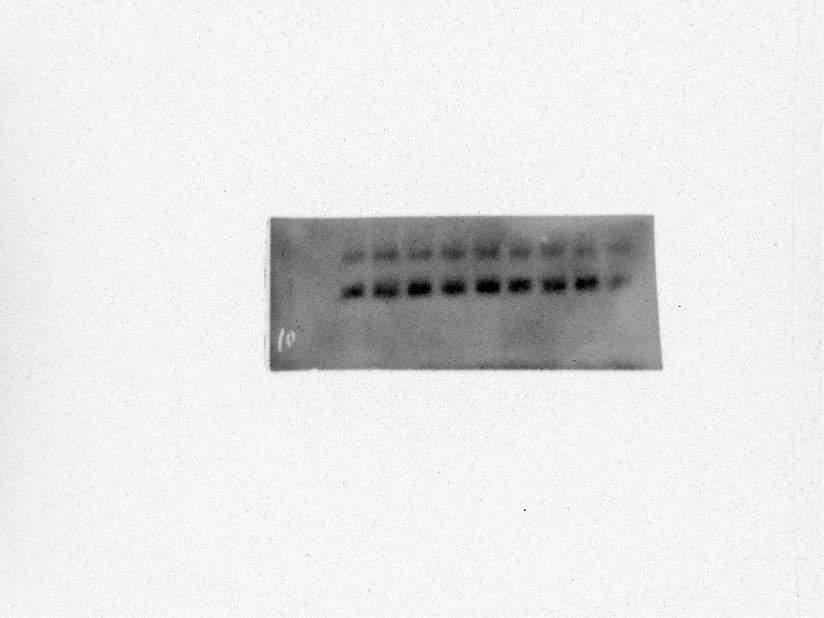

Supplement: Figure 6—figure supplement 1—source data 1. [file elife-98372-fig6-figsupp1-data1.zip › Figure 6-supplementary figure 1-data1/Figure_6-figure supplement_1_ source_data_1_ Figure_D_NOXA(Kidney).jpg]

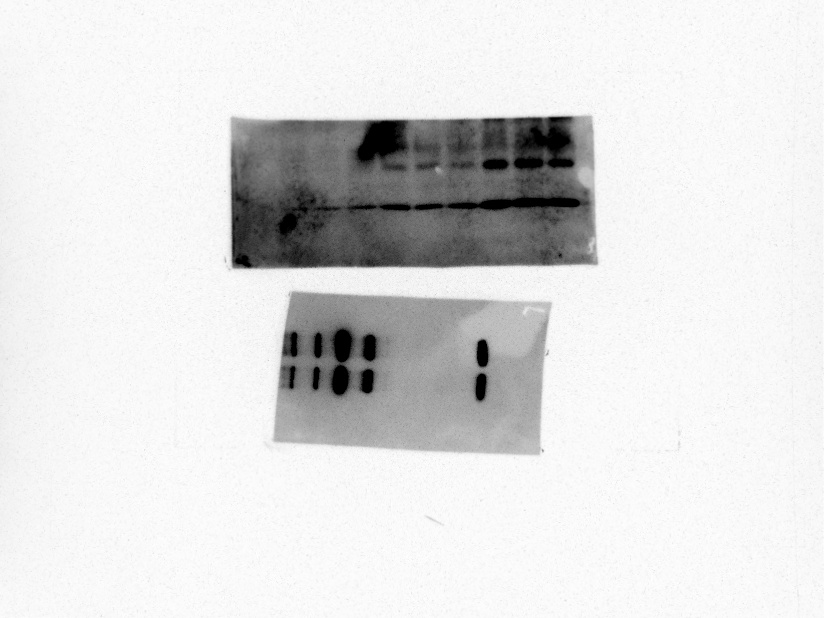

Supplement: Figure 6—figure supplement 1—source data 1. [file elife-98372-fig6-figsupp1-data1.zip › Figure 6-supplementary figure 1-data1/Figure_6-figure supplement_1_ source_data_1_ Figure_D_NOXA(Liver).jpg]

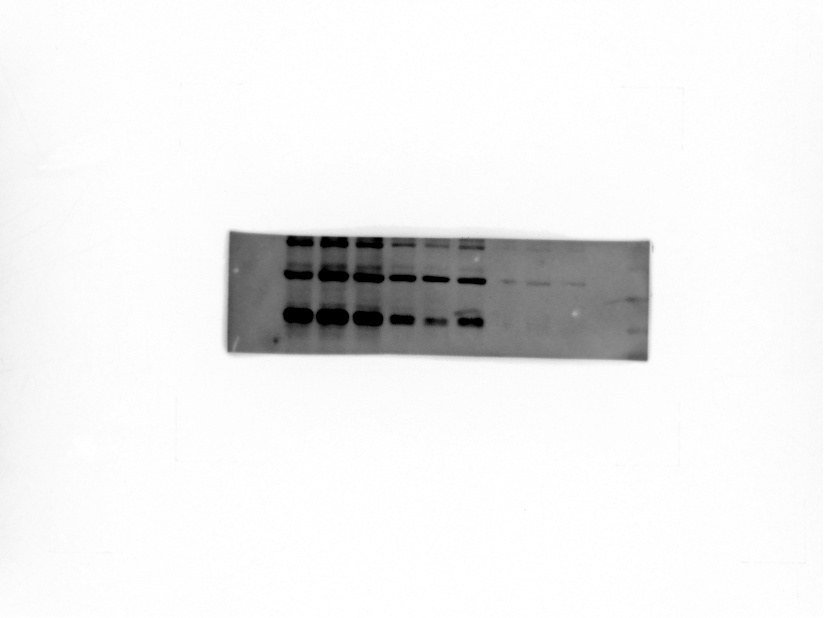

Supplement: Figure 6—figure supplement 1—source data 1. [file elife-98372-fig6-figsupp1-data1.zip › Figure 6-supplementary figure 1-data1/Figure_6-figure supplement_1_ source_data_1_ Figure_D_WSB2(Brain).jpg]

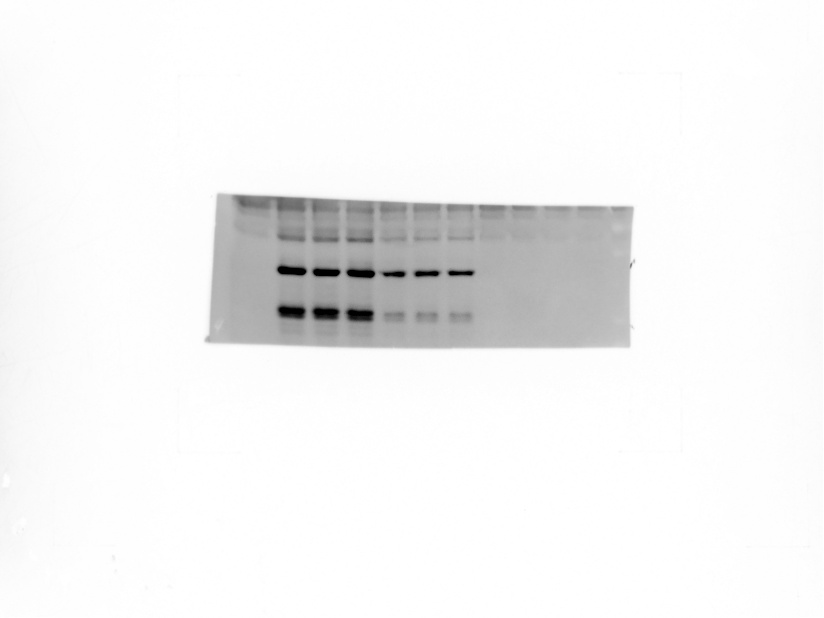

Supplement: Figure 6—figure supplement 1—source data 1. [file elife-98372-fig6-figsupp1-data1.zip › Figure 6-supplementary figure 1-data1/Figure_6-figure supplement_1_ source_data_1_ Figure_D_WSB2(Heart).jpg]

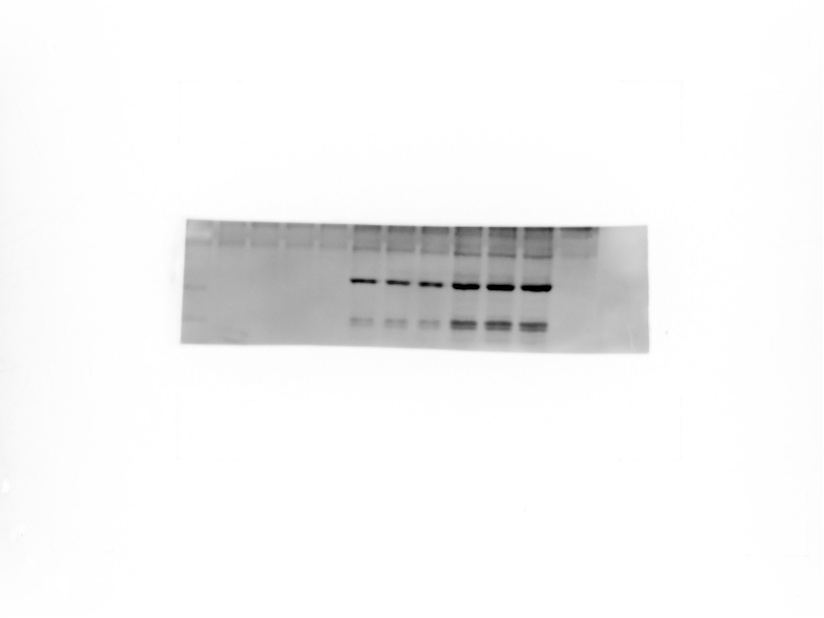

Supplement: Figure 6—figure supplement 1—source data 1. [file elife-98372-fig6-figsupp1-data1.zip › Figure 6-supplementary figure 1-data1/Figure_6-figure supplement_1_ source_data_1_ Figure_D_WSB2(Kidney).jpg]

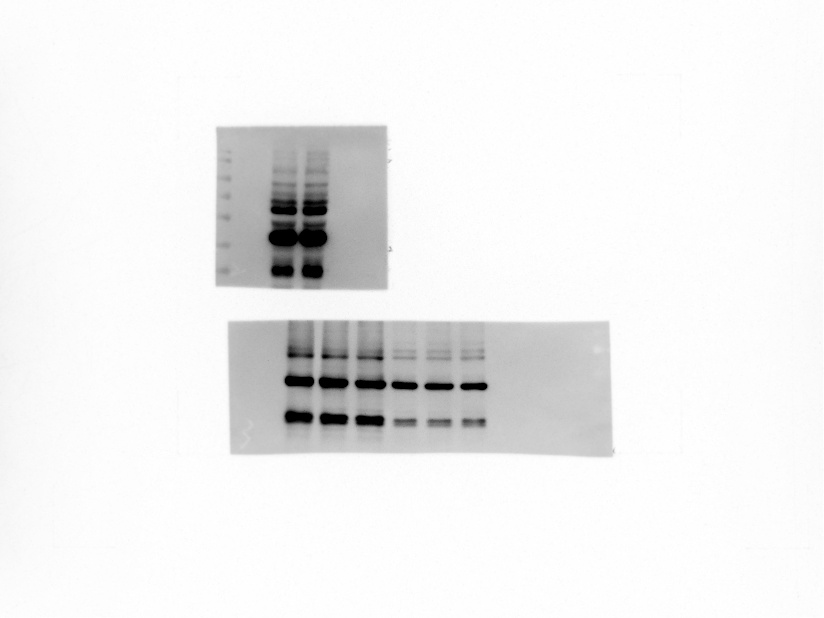

Supplement: Figure 6—figure supplement 1—source data 1. [file elife-98372-fig6-figsupp1-data1.zip › Figure 6-supplementary figure 1-data1/Figure_6-figure supplement_1_ source_data_1_ Figure_D_WSB2(Lung).jpg]

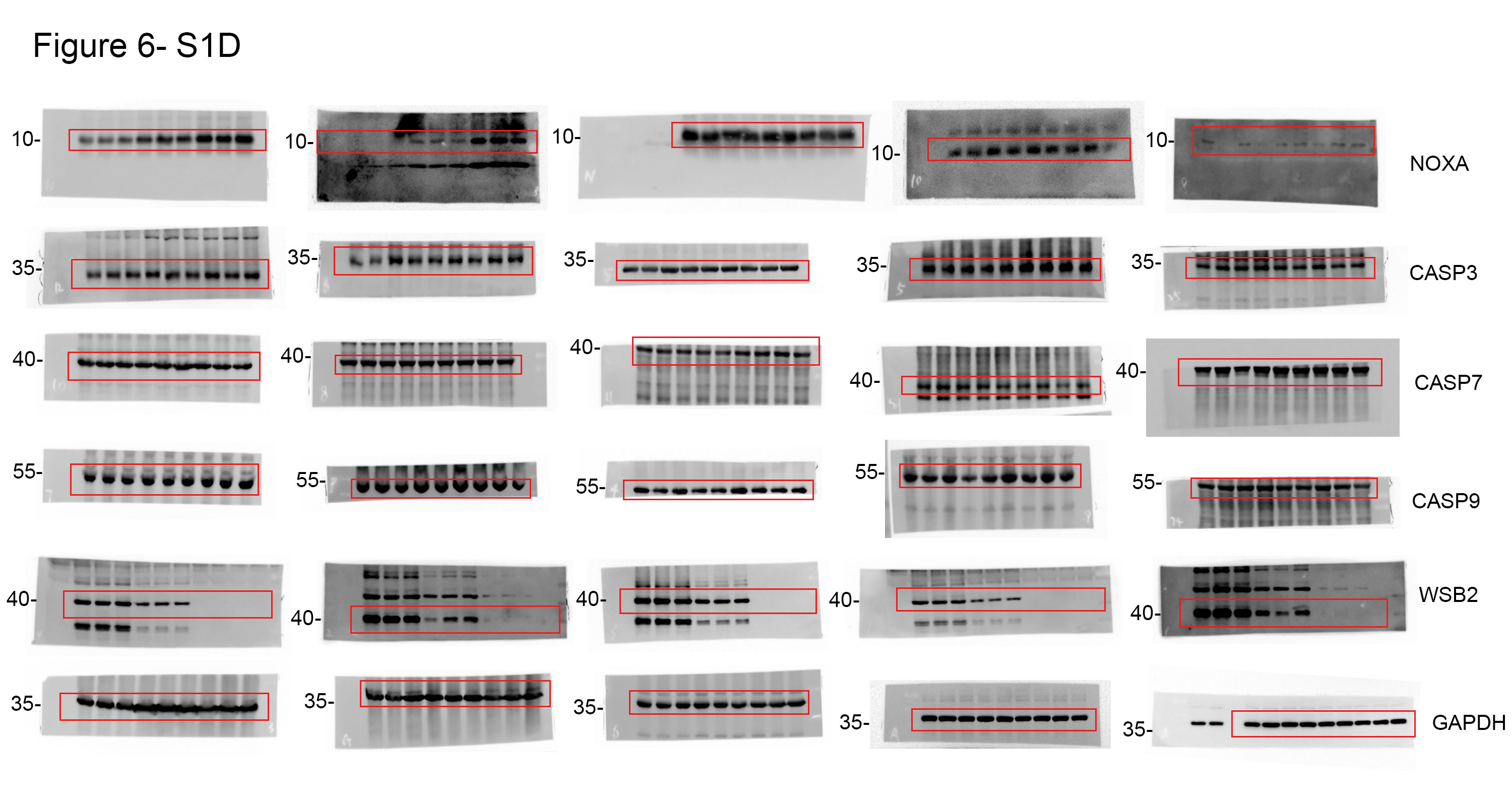

Supplement: Figure 6—figure supplement 1—source data 2. [file elife-98372-fig6-figsupp1-data2.zip › Figure 6-supplementary figure 1-data2/Figure_6_supplement figure_1_data_2.jpg]

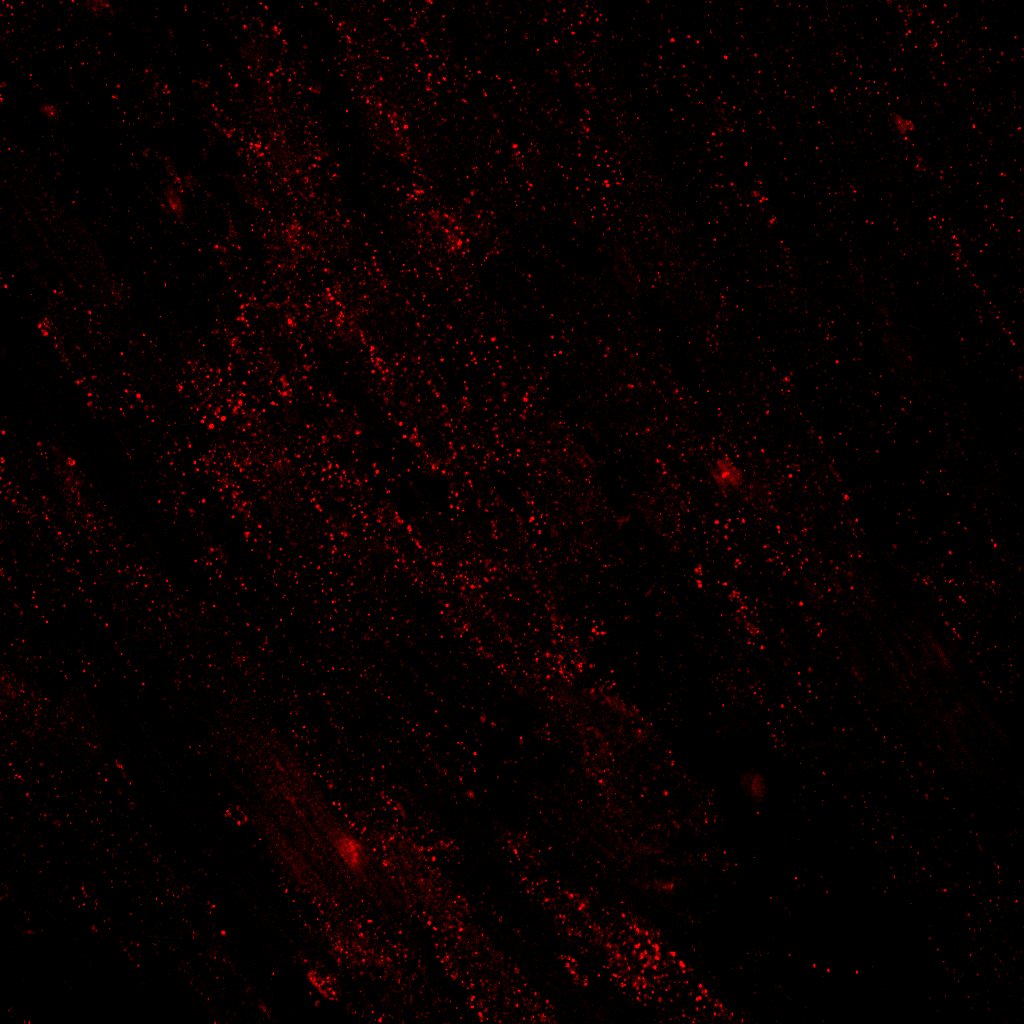

Supplement: Figure 6—figure supplement 2—source data 1. [file elife-98372-fig6-figsupp2-data1.zip › Figure 6-supplementary figure 2-data1/Figure_6-figure supplement_2_source_data_1_Figure_A_Heart_homo_ABT-199_cl-CASP7(cl-CASP7).jpg]

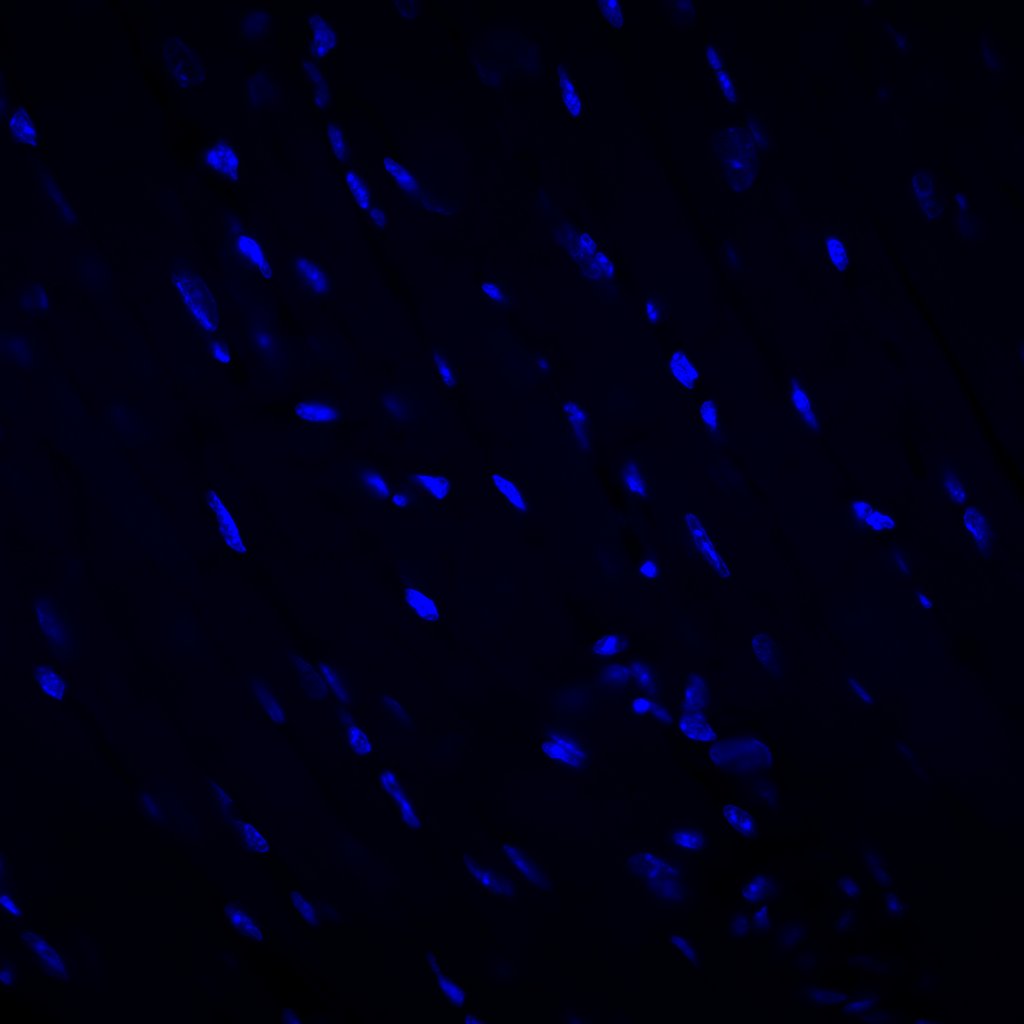

Supplement: Figure 6—figure supplement 2—source data 1. [file elife-98372-fig6-figsupp2-data1.zip › Figure 6-supplementary figure 2-data1/Figure_6-figure supplement_2_source_data_1_Figure_A_Heart_homo_ABT-199_cl-CASP7(DAPI).jpg]

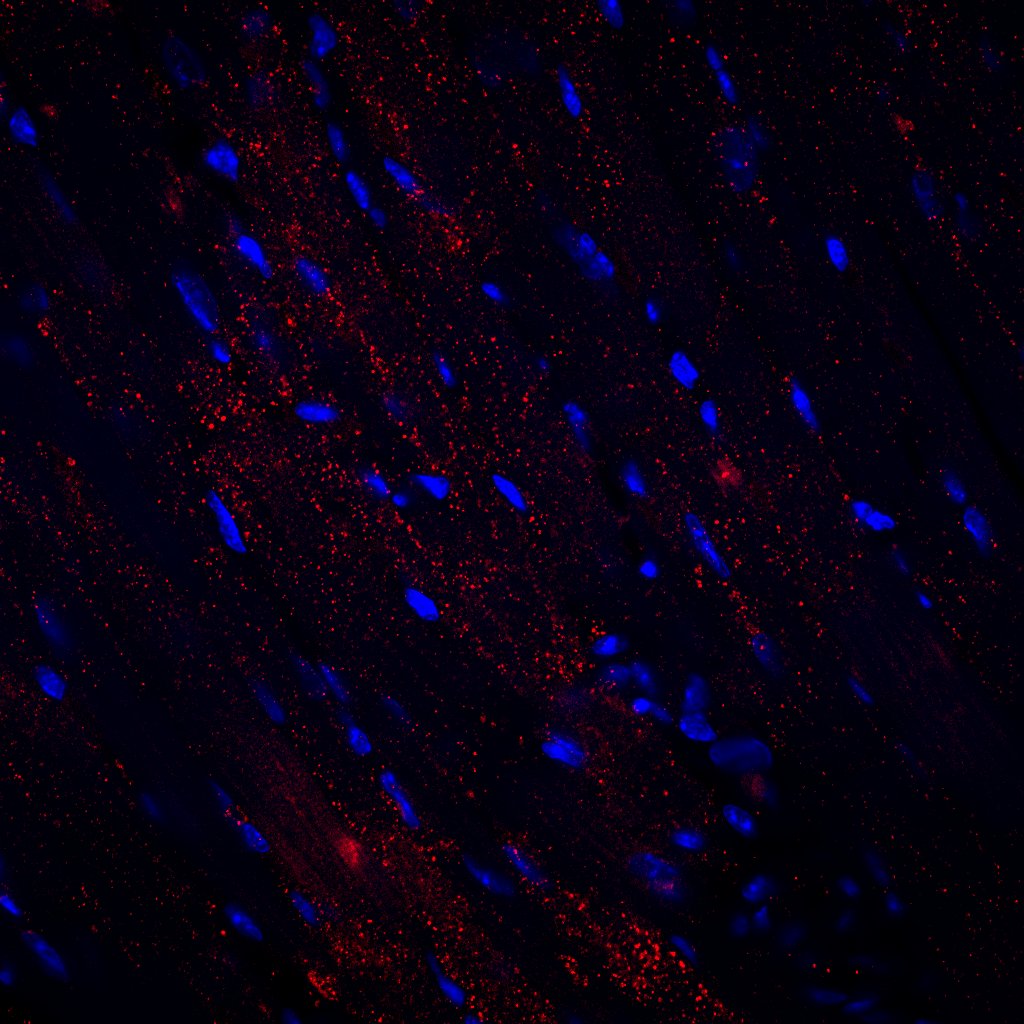

Supplement: Figure 6—figure supplement 2—source data 1. [file elife-98372-fig6-figsupp2-data1.zip › Figure 6-supplementary figure 2-data1/Figure_6-figure supplement_2_source_data_1_Figure_A_Heart_homo_ABT-199_cl-CASP7(Merge).jpg]

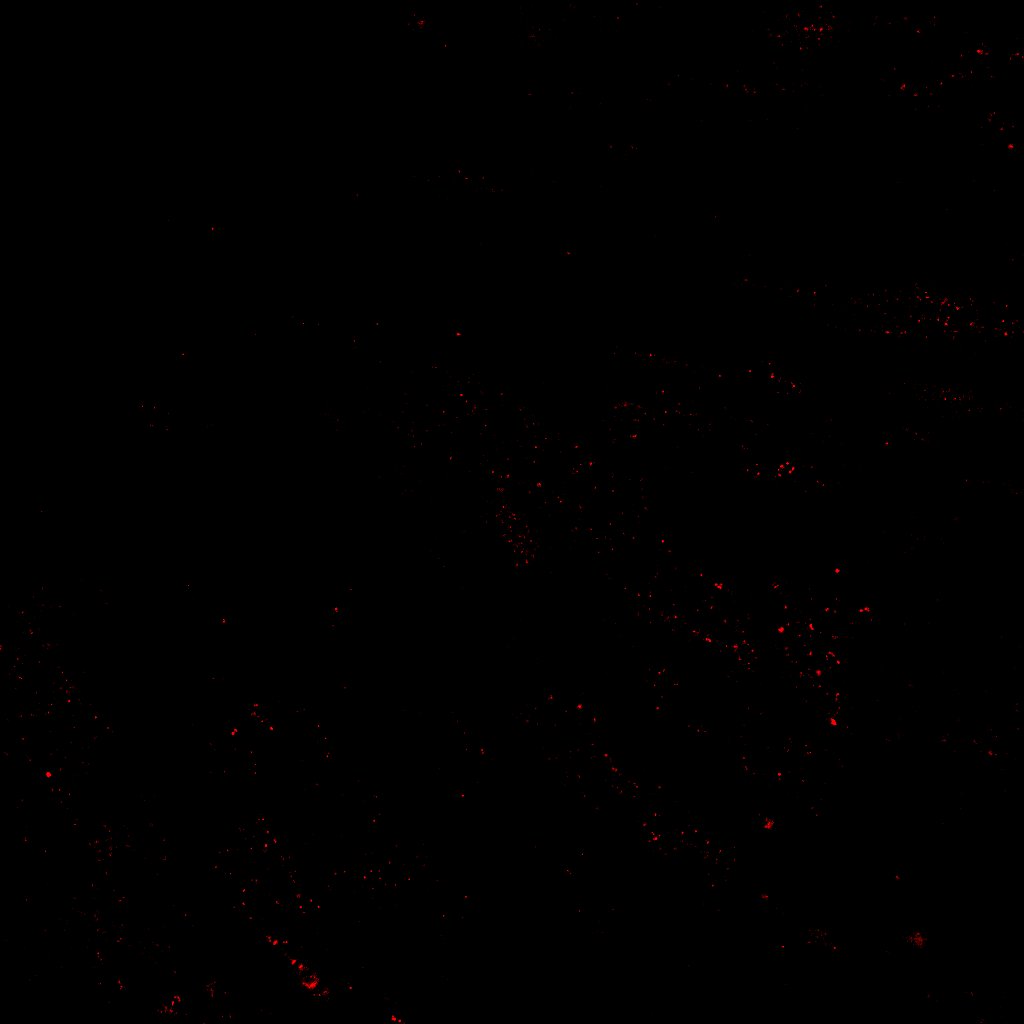

Supplement: Figure 6—figure supplement 2—source data 1. [file elife-98372-fig6-figsupp2-data1.zip › Figure 6-supplementary figure 2-data1/Figure_6-figure supplement_2_source_data_1_Figure_A_Heart_homo_cl-CASP7(cl-CASP7).jpg]

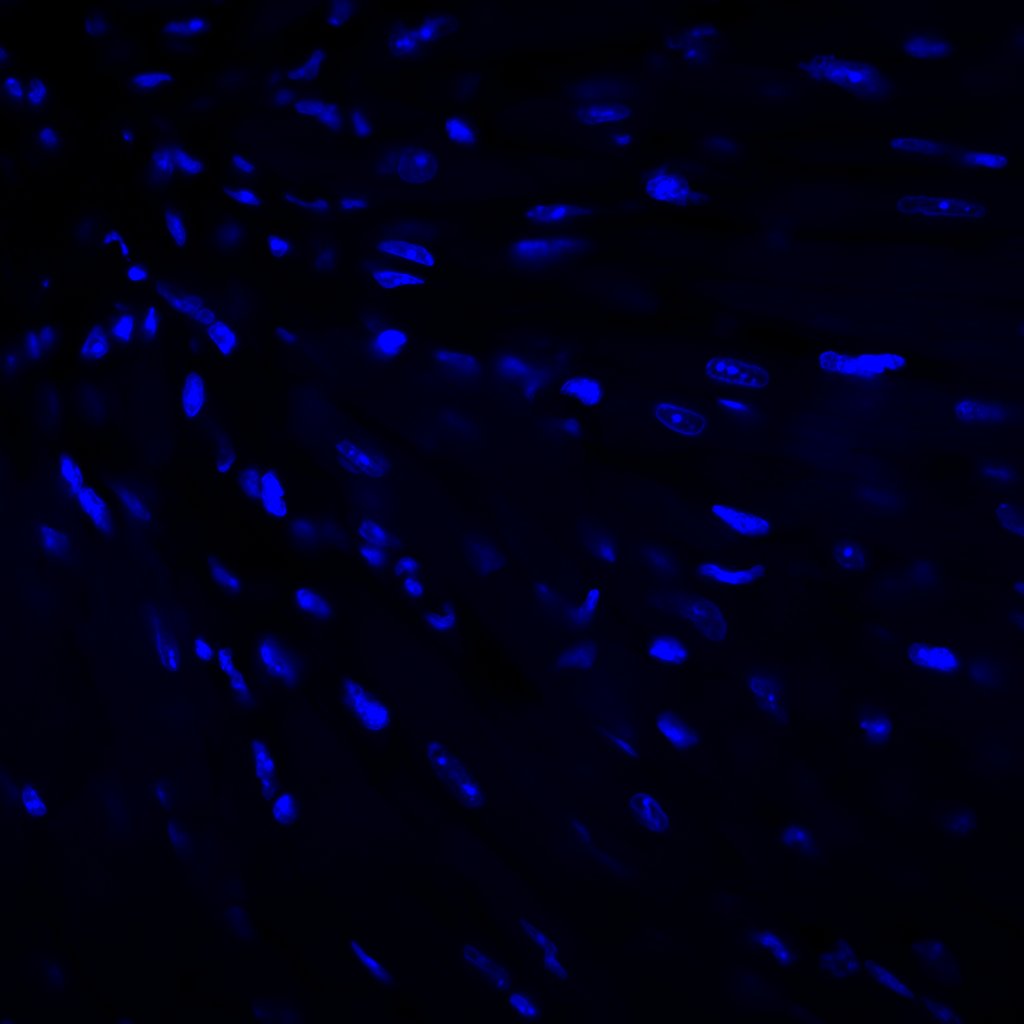

Supplement: Figure 6—figure supplement 2—source data 1. [file elife-98372-fig6-figsupp2-data1.zip › Figure 6-supplementary figure 2-data1/Figure_6-figure supplement_2_source_data_1_Figure_A_Heart_homo_cl-CASP7(DAPI).jpg]

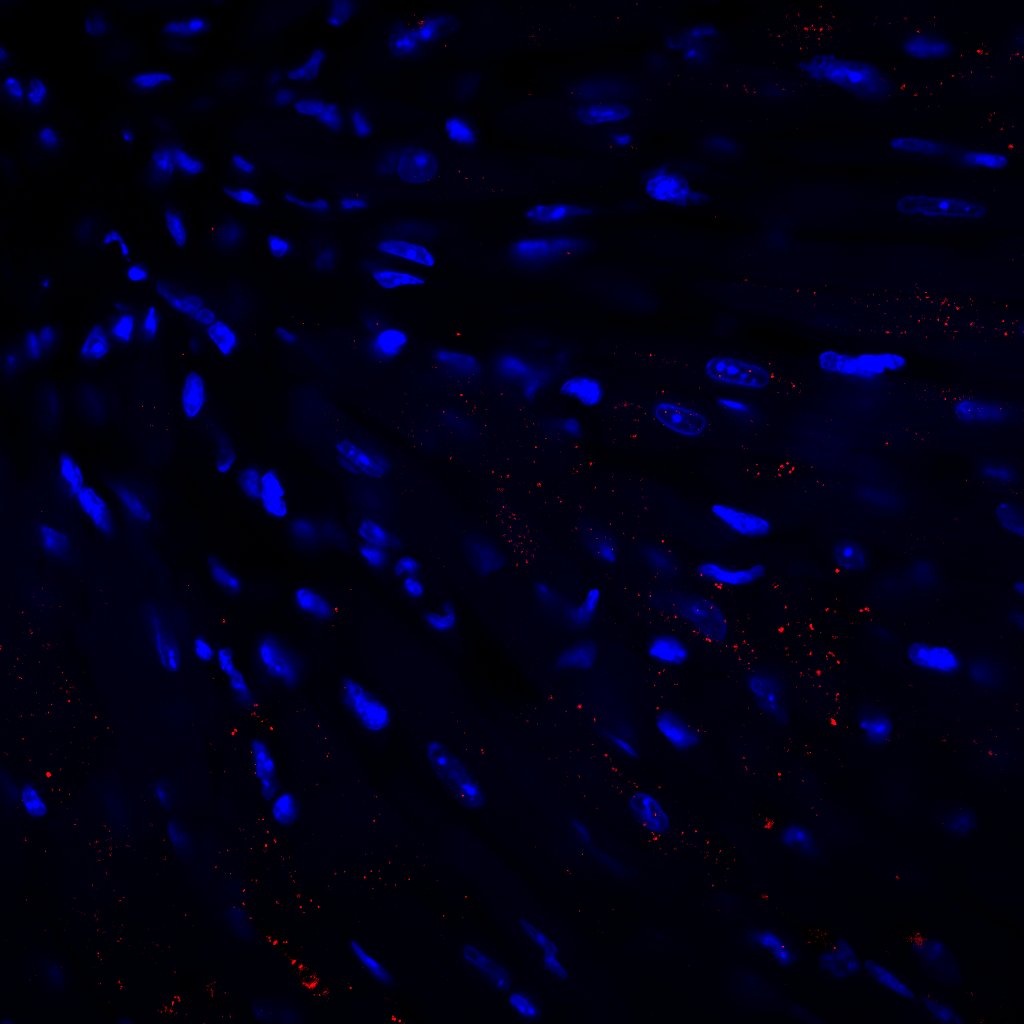

Supplement: Figure 6—figure supplement 2—source data 1. [file elife-98372-fig6-figsupp2-data1.zip › Figure 6-supplementary figure 2-data1/Figure_6-figure supplement_2_source_data_1_Figure_A_Heart_homo_cl-CASP7(Merge).jpg]

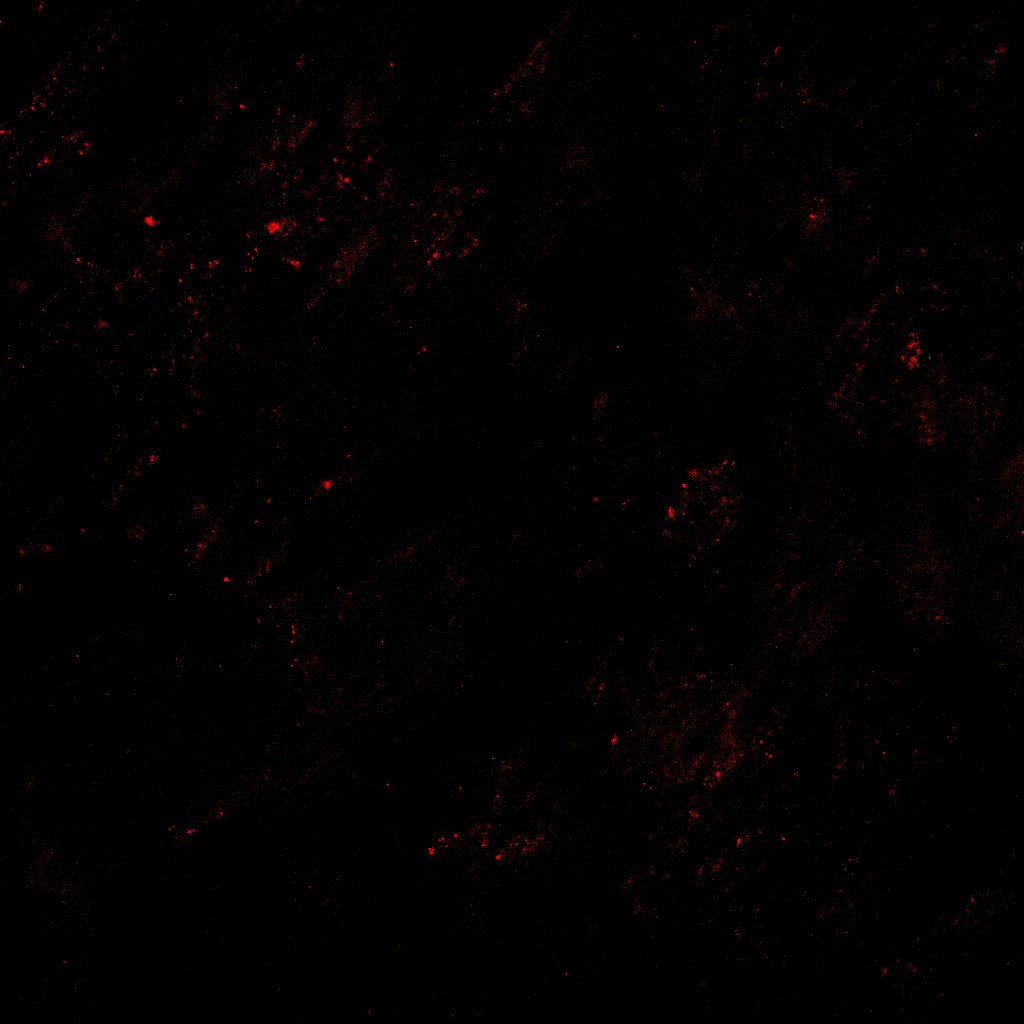

Supplement: Figure 6—figure supplement 2—source data 1. [file elife-98372-fig6-figsupp2-data1.zip › Figure 6-supplementary figure 2-data1/Figure_6-figure supplement_2_source_data_1_Figure_A_Heart_WT_ABT-199_cl-CASP7(cl-CASP7).jpg]

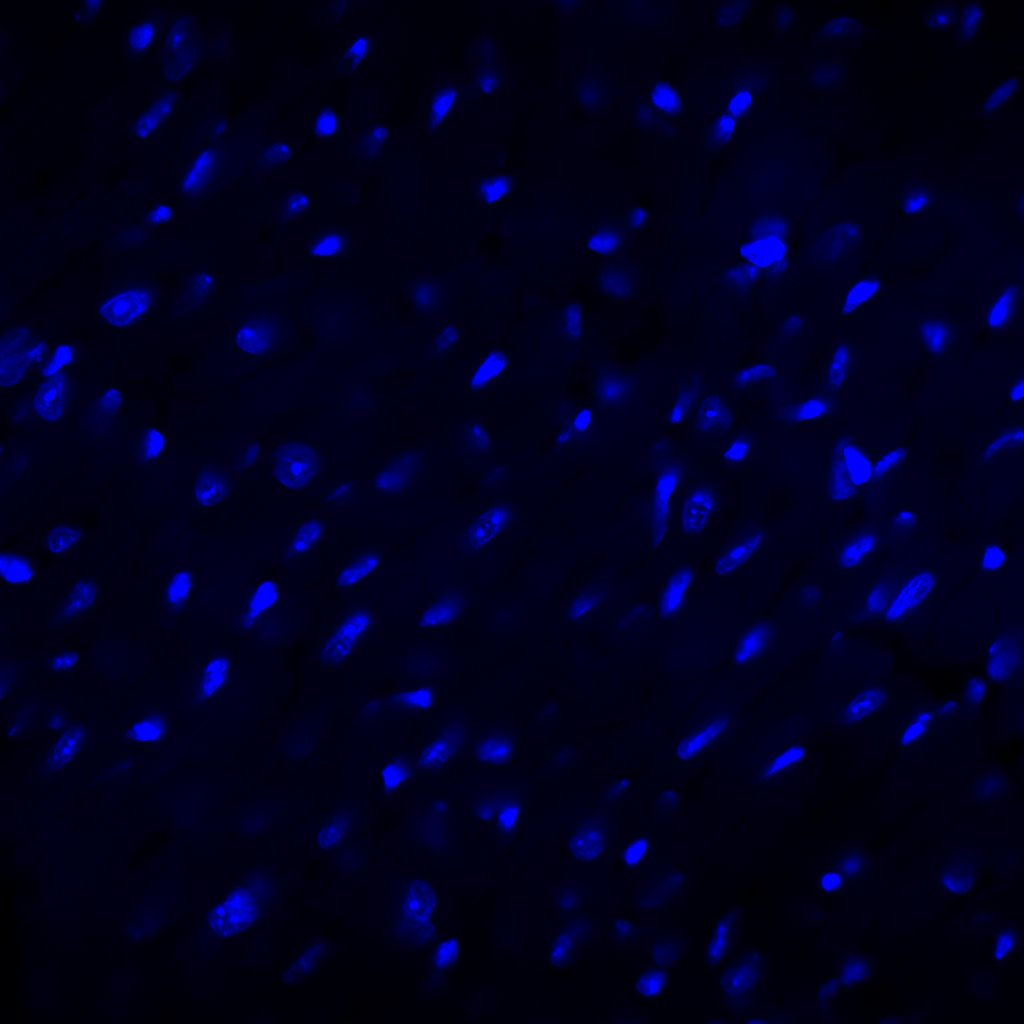

Supplement: Figure 6—figure supplement 2—source data 1. [file elife-98372-fig6-figsupp2-data1.zip › Figure 6-supplementary figure 2-data1/Figure_6-figure supplement_2_source_data_1_Figure_A_Heart_WT_ABT-199_cl-CASP7(DAPI).jpg]

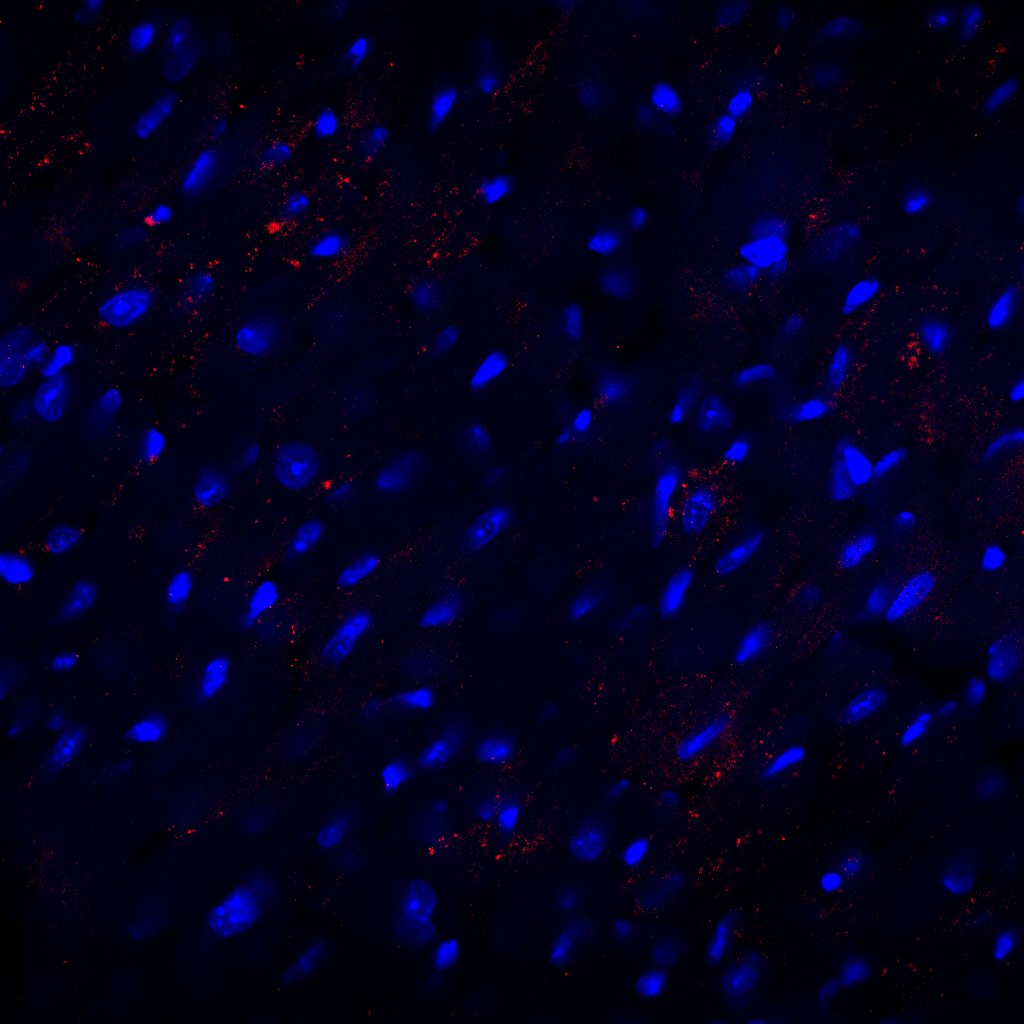

Supplement: Figure 6—figure supplement 2—source data 1. [file elife-98372-fig6-figsupp2-data1.zip › Figure 6-supplementary figure 2-data1/Figure_6-figure supplement_2_source_data_1_Figure_A_Heart_WT_ABT-199_cl-CASP7(Merge).jpg]

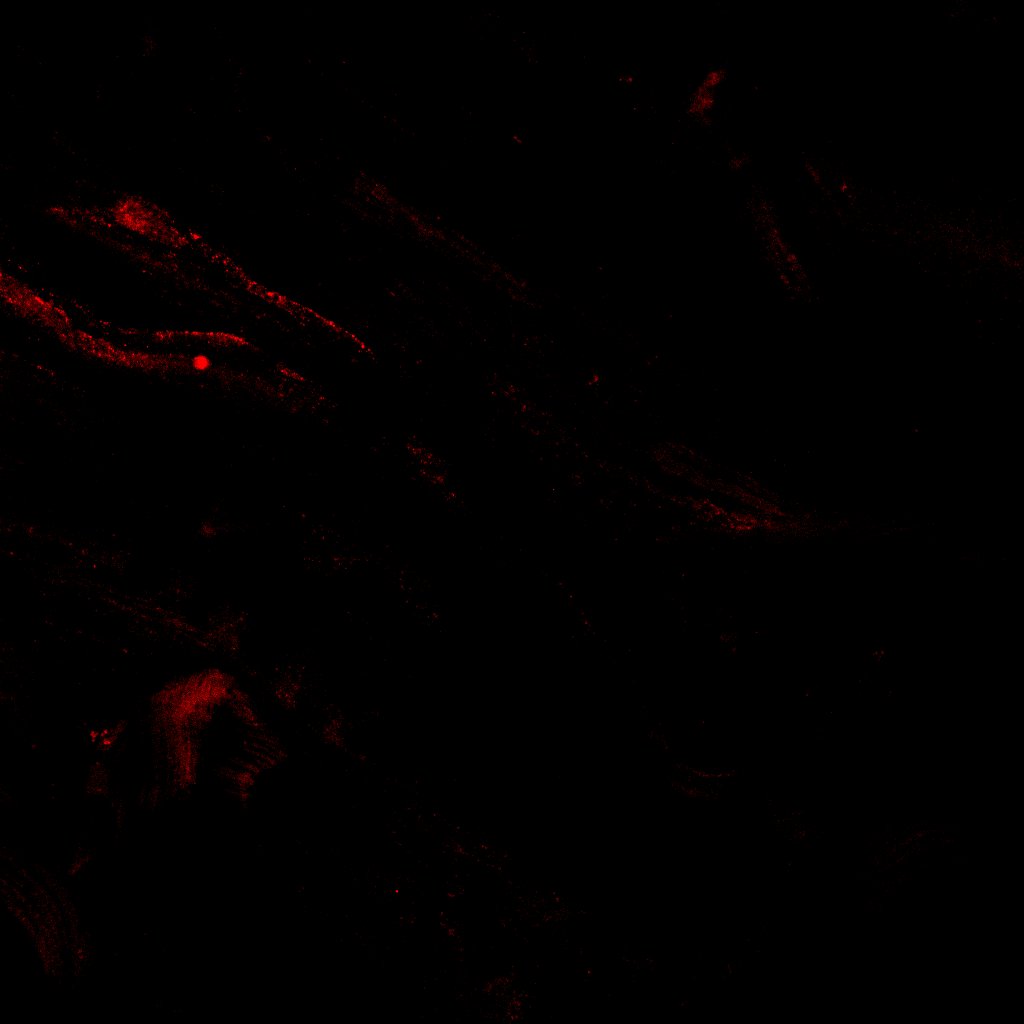

Supplement: Figure 6—figure supplement 2—source data 1. [file elife-98372-fig6-figsupp2-data1.zip › Figure 6-supplementary figure 2-data1/Figure_6-figure supplement_2_source_data_1_Figure_A_Heart_WT_cl-CASP7(cl-CASP7).jpg]

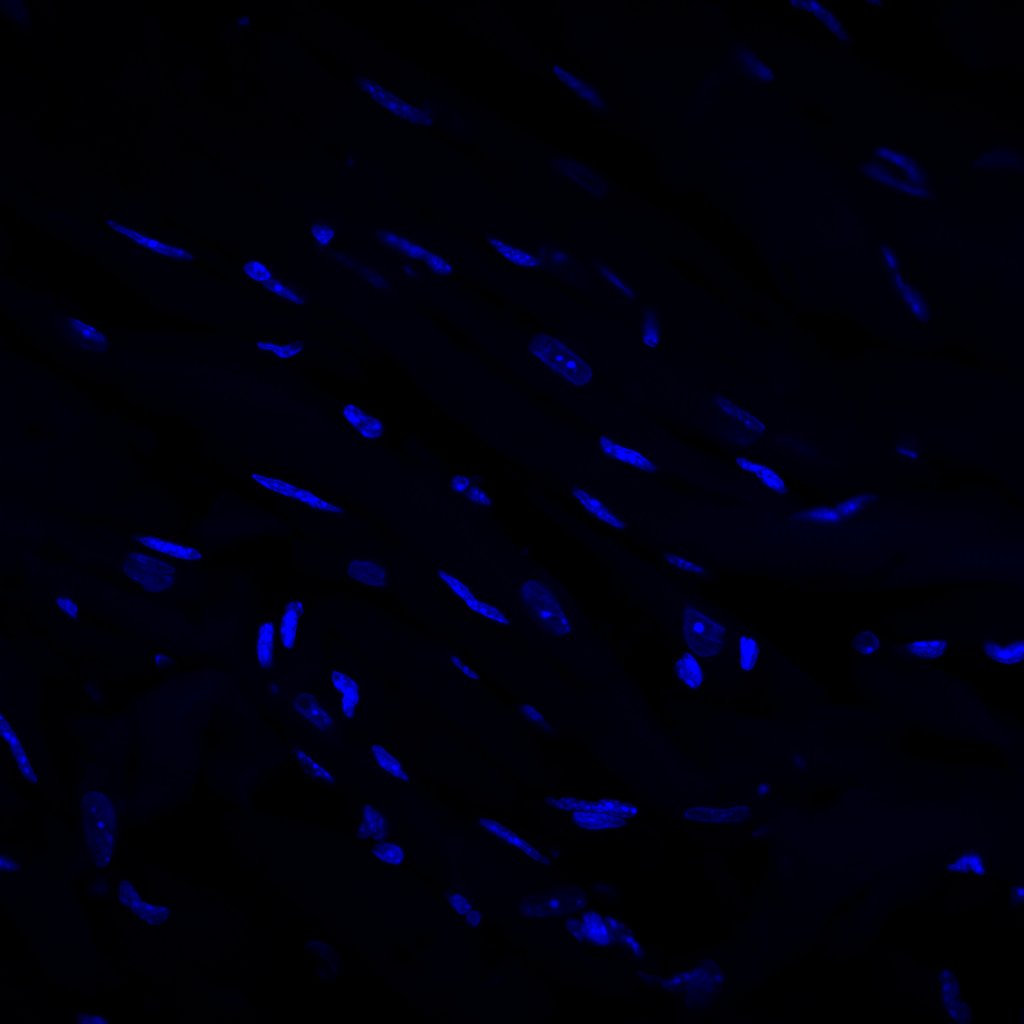

Supplement: Figure 6—figure supplement 2—source data 1. [file elife-98372-fig6-figsupp2-data1.zip › Figure 6-supplementary figure 2-data1/Figure_6-figure supplement_2_source_data_1_Figure_A_Heart_WT_cl-CASP7(DAPI).jpg]

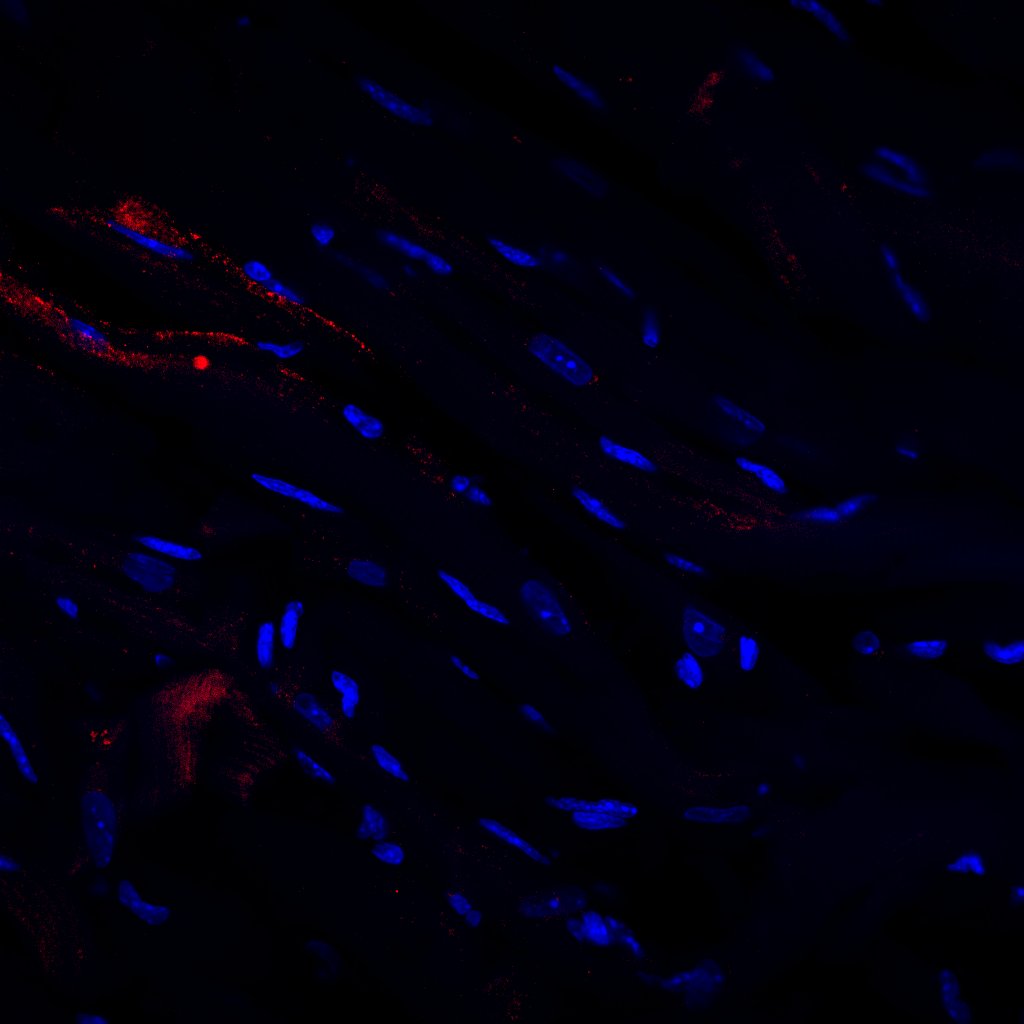

Supplement: Figure 6—figure supplement 2—source data 1. [file elife-98372-fig6-figsupp2-data1.zip › Figure 6-supplementary figure 2-data1/Figure_6-figure supplement_2_source_data_1_Figure_A_Heart_WT_cl-CASP7(Merge).jpg]

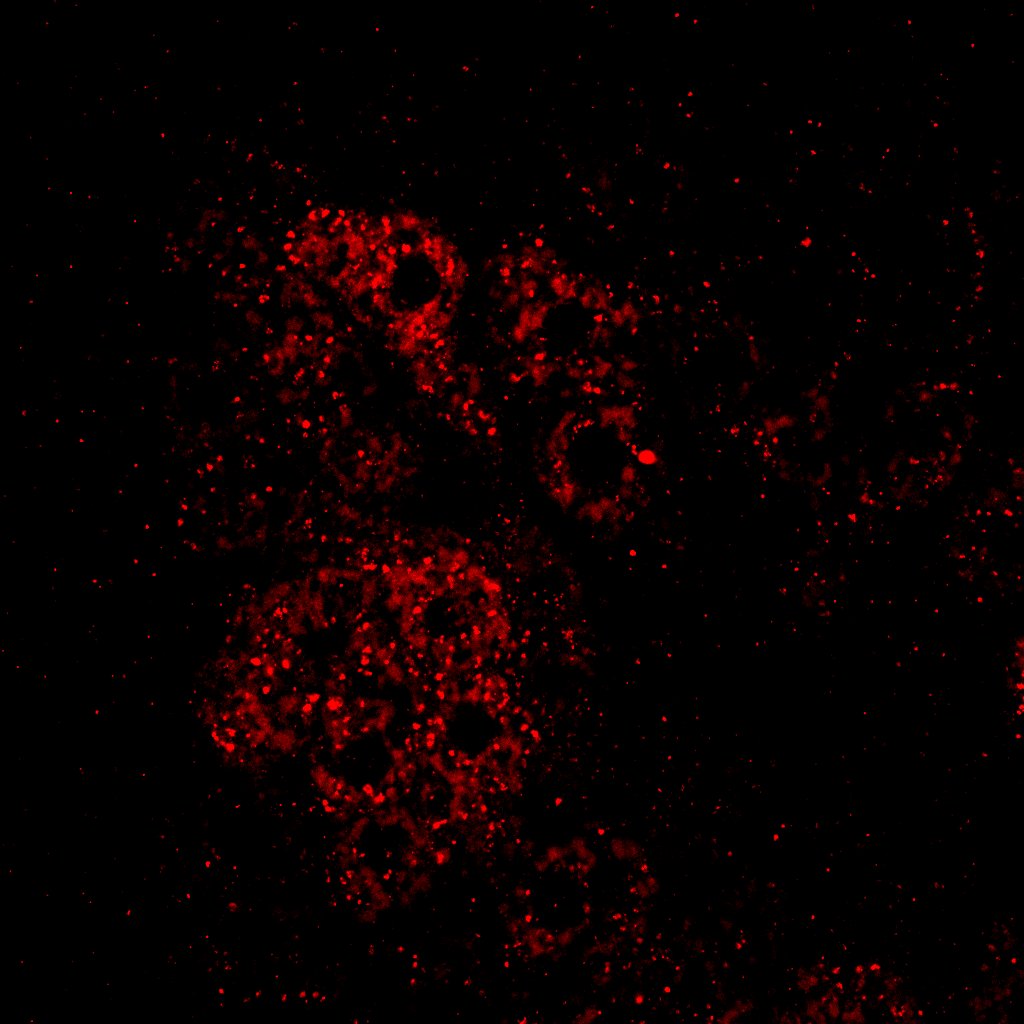

Supplement: Figure 6—figure supplement 2—source data 1. [file elife-98372-fig6-figsupp2-data1.zip › Figure 6-supplementary figure 2-data1/Figure_6-figure supplement_2_source_data_1_Figure_A_Liver_homo_ABT-199_cl-CASP7(cl-CASP7).jpg]

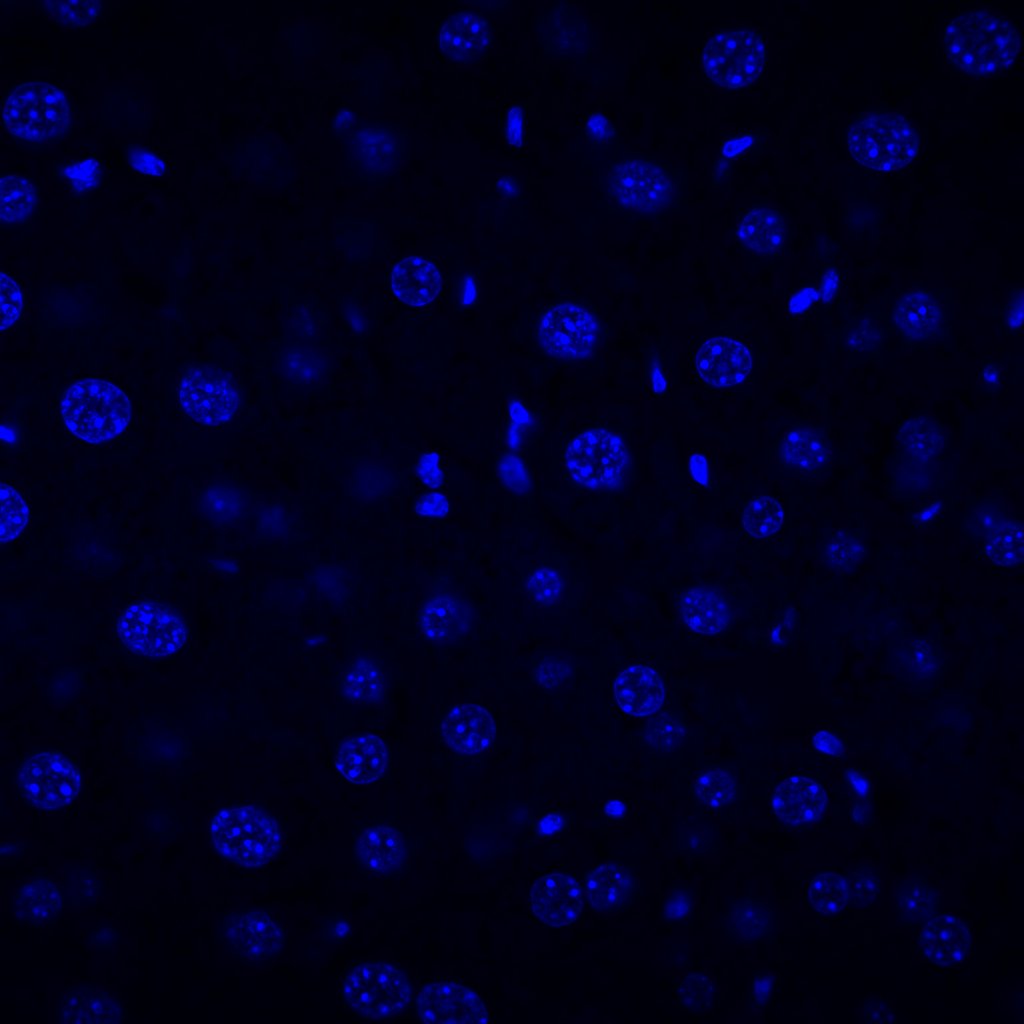

Supplement: Figure 6—figure supplement 2—source data 1. [file elife-98372-fig6-figsupp2-data1.zip › Figure 6-supplementary figure 2-data1/Figure_6-figure supplement_2_source_data_1_Figure_A_Liver_homo_ABT-199_cl-CASP7(DAPI).jpg]

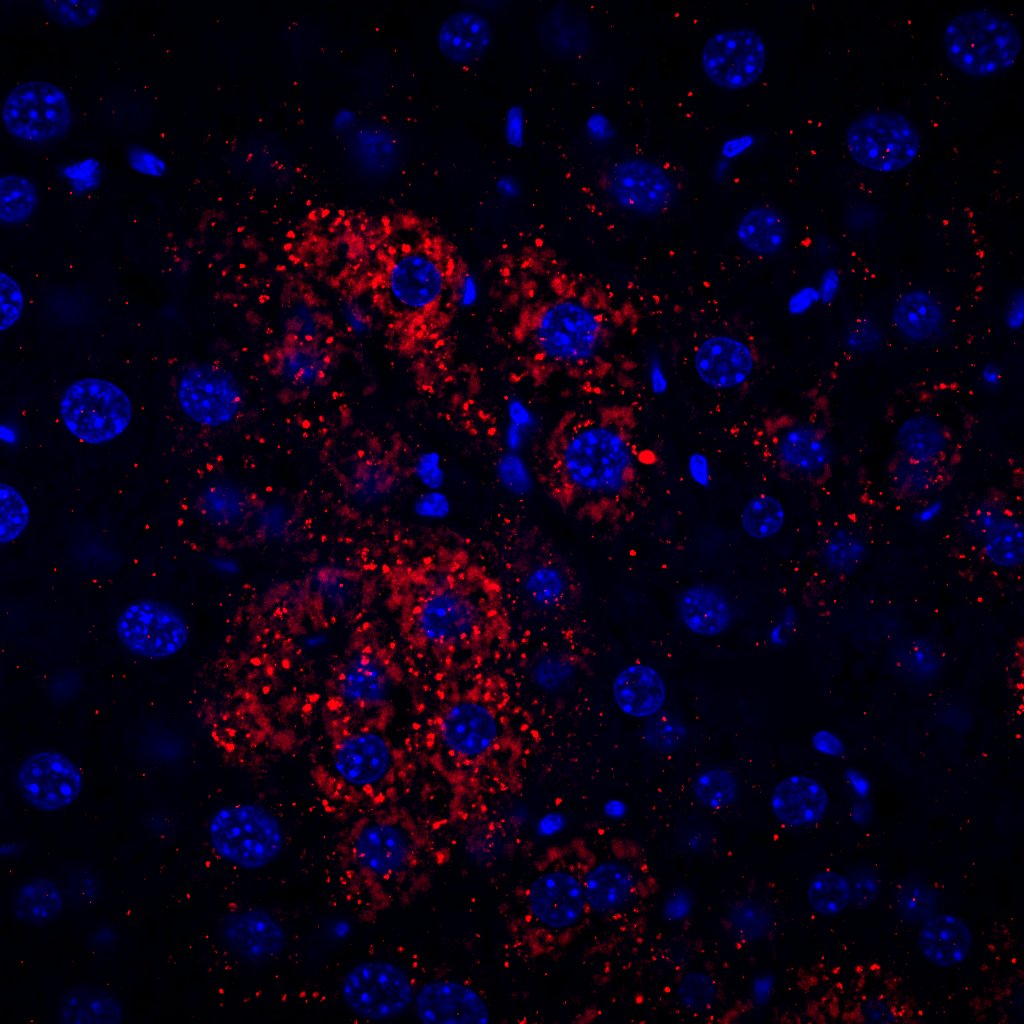

Supplement: Figure 6—figure supplement 2—source data 1. [file elife-98372-fig6-figsupp2-data1.zip › Figure 6-supplementary figure 2-data1/Figure_6-figure supplement_2_source_data_1_Figure_A_Liver_homo_ABT-199_cl-CASP7(Merge).jpg]

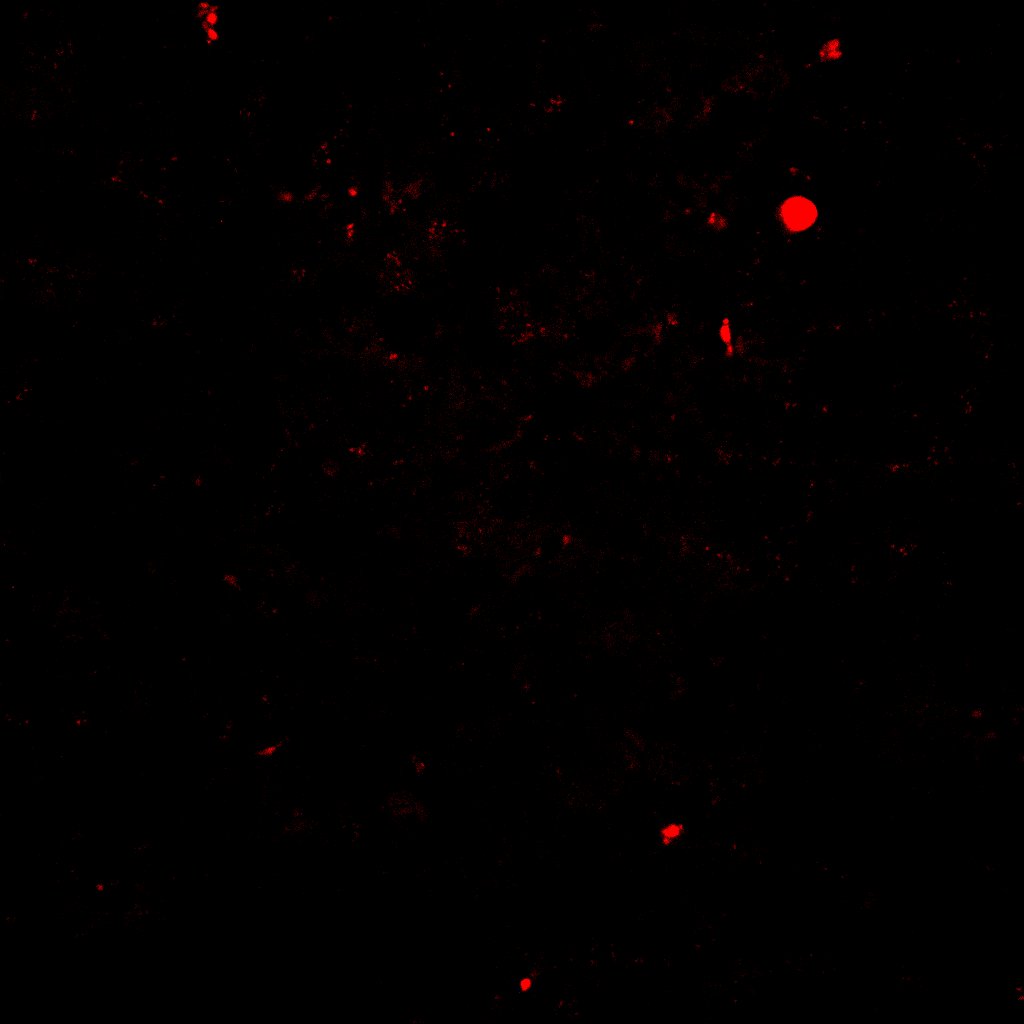

Supplement: Figure 6—figure supplement 2—source data 1. [file elife-98372-fig6-figsupp2-data1.zip › Figure 6-supplementary figure 2-data1/Figure_6-figure supplement_2_source_data_1_Figure_A_Liver_homo_cl-CASP7(cl-CASP7).jpg]

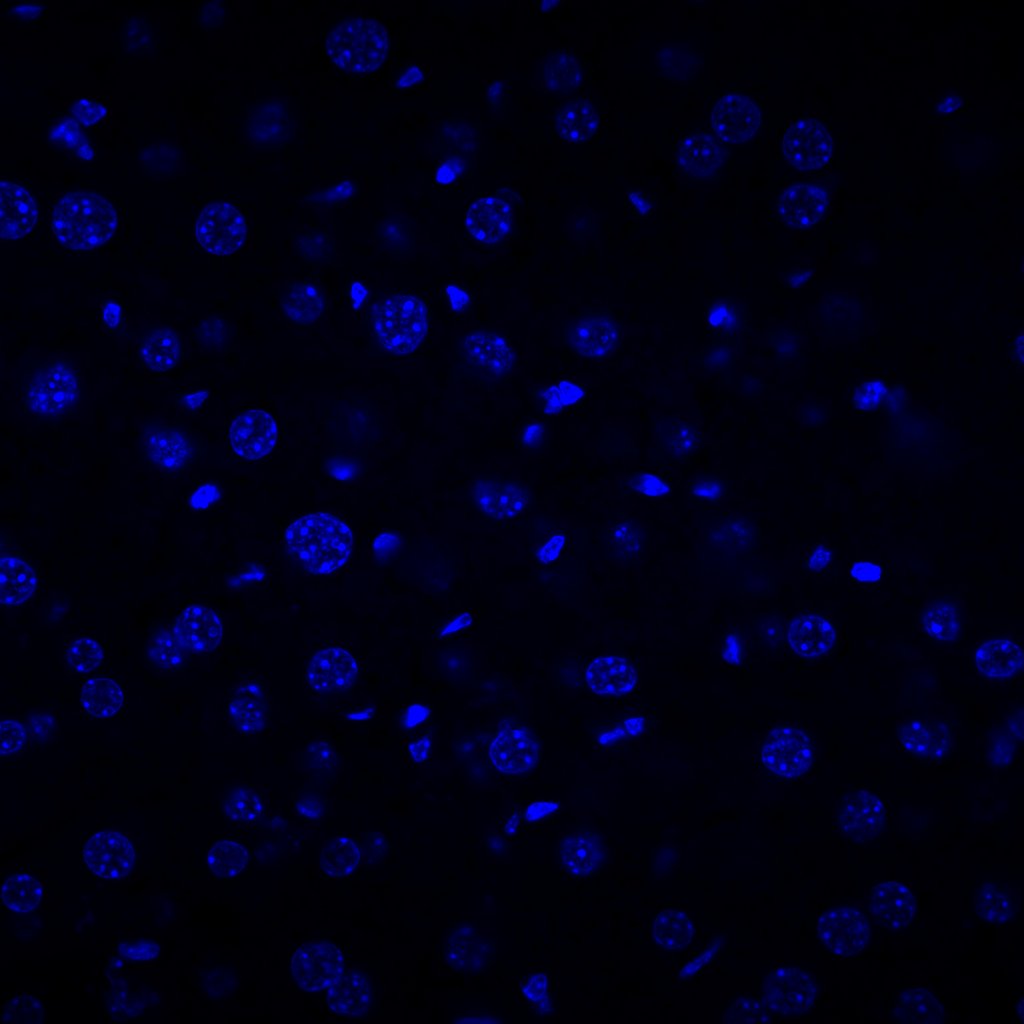

Supplement: Figure 6—figure supplement 2—source data 1. [file elife-98372-fig6-figsupp2-data1.zip › Figure 6-supplementary figure 2-data1/Figure_6-figure supplement_2_source_data_1_Figure_A_Liver_homo_cl-CASP7(DAPI).jpg]

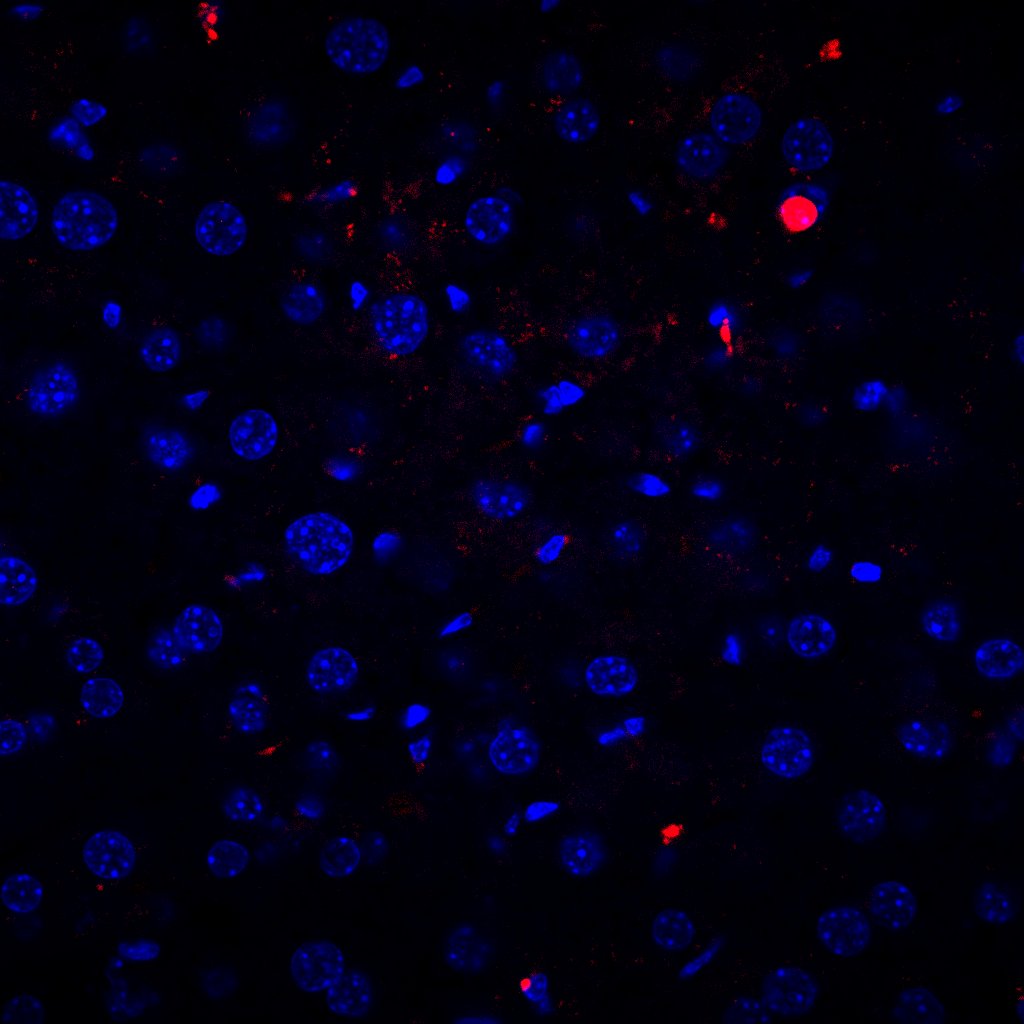

Supplement: Figure 6—figure supplement 2—source data 1. [file elife-98372-fig6-figsupp2-data1.zip › Figure 6-supplementary figure 2-data1/Figure_6-figure supplement_2_source_data_1_Figure_A_Liver_homo_cl-CASP7(Merge).jpg]

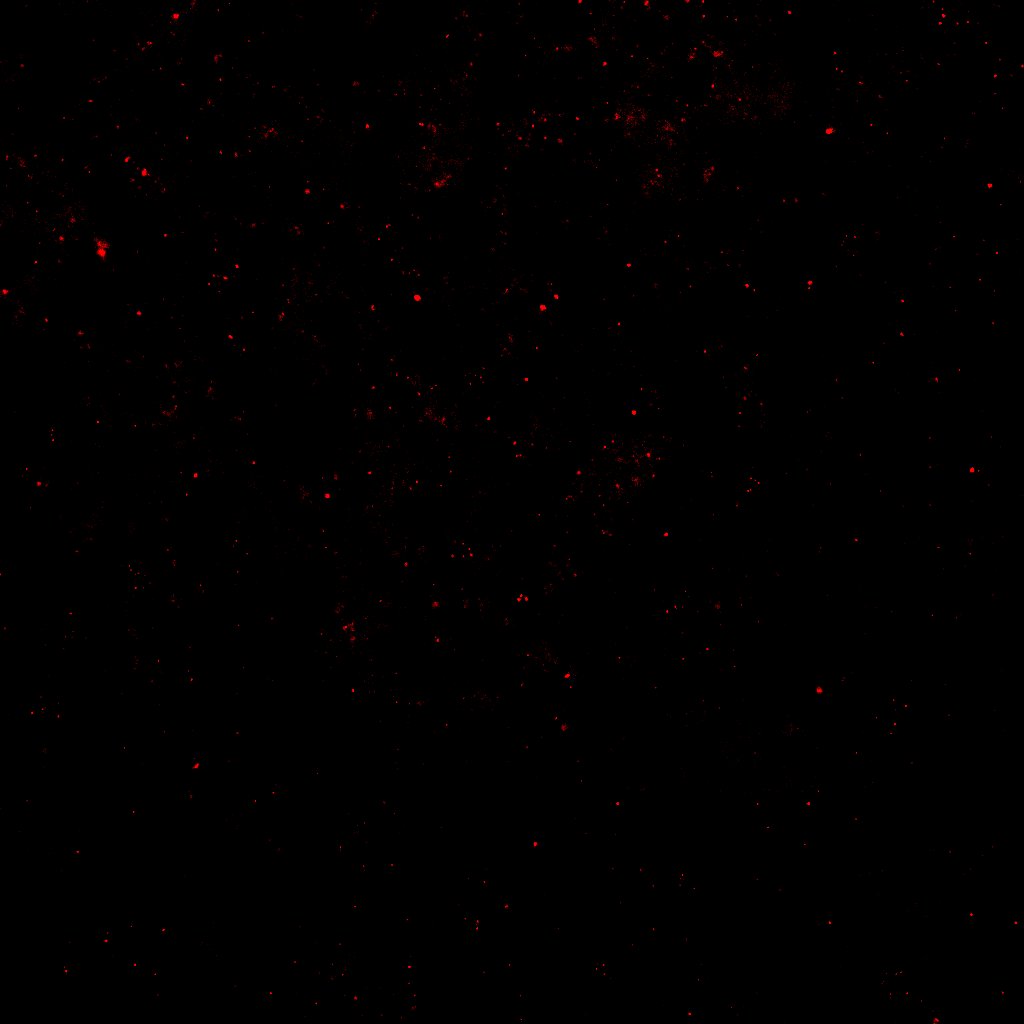

Supplement: Figure 6—figure supplement 2—source data 1. [file elife-98372-fig6-figsupp2-data1.zip › Figure 6-supplementary figure 2-data1/Figure_6-figure supplement_2_source_data_1_Figure_A_Liver_WT_ABT-199_cl-CASP7(cl-CASP7).jpg]

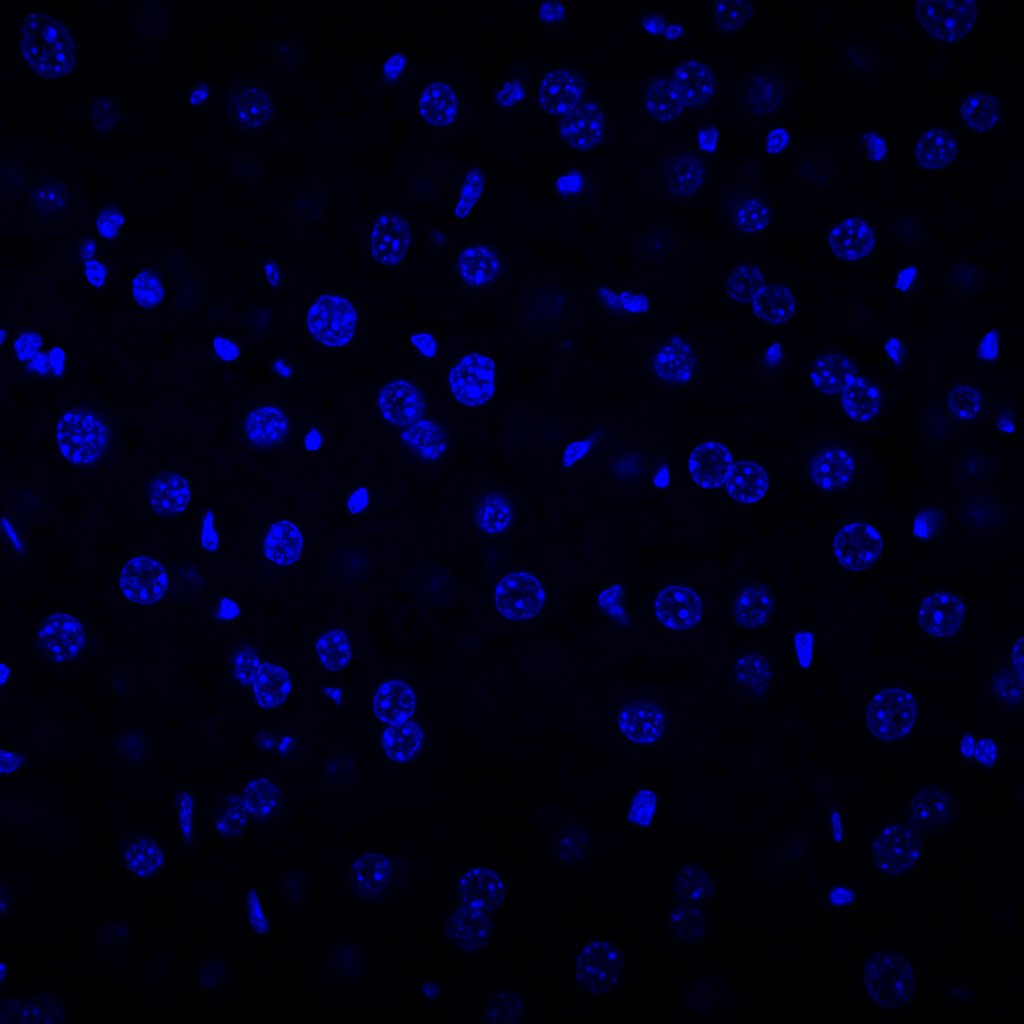

Supplement: Figure 6—figure supplement 2—source data 1. [file elife-98372-fig6-figsupp2-data1.zip › Figure 6-supplementary figure 2-data1/Figure_6-figure supplement_2_source_data_1_Figure_A_Liver_WT_ABT-199_cl-CASP7(DAPI).jpg]

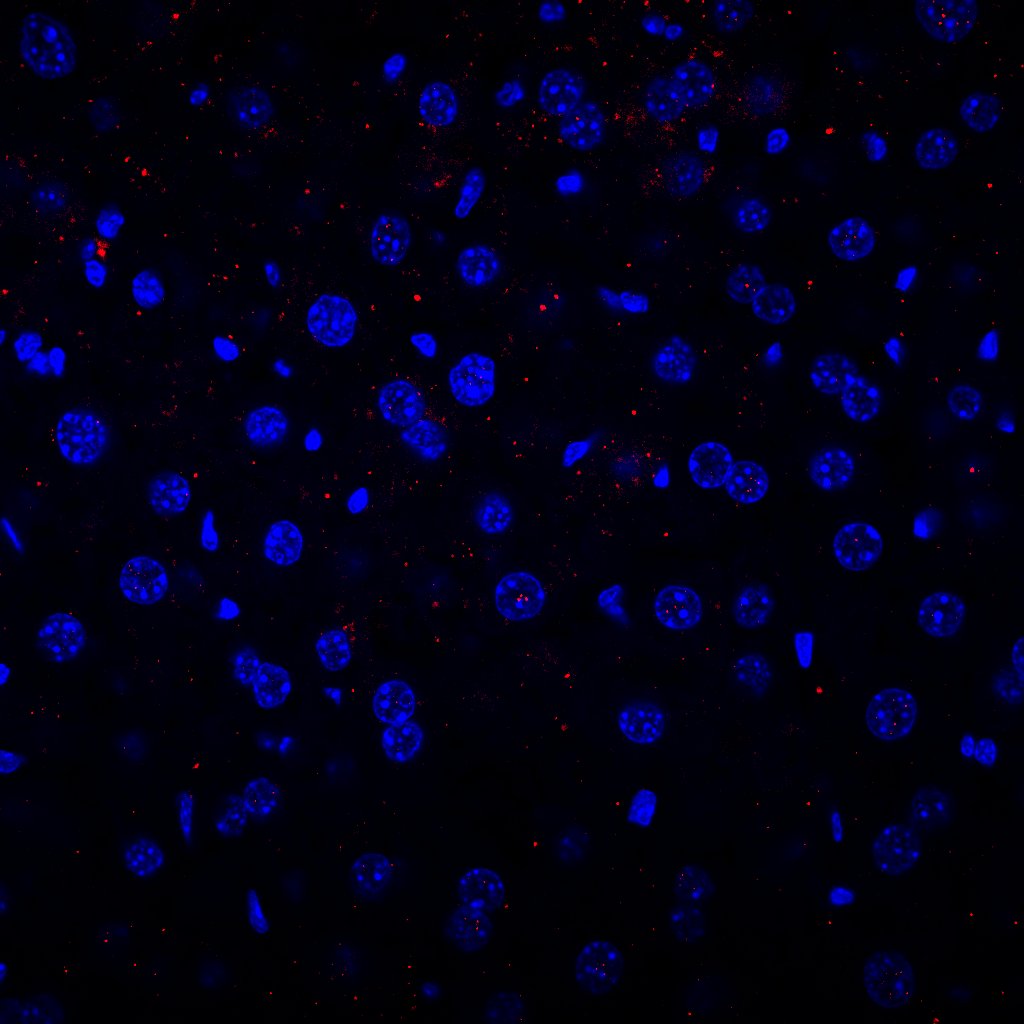

Supplement: Figure 6—figure supplement 2—source data 1. [file elife-98372-fig6-figsupp2-data1.zip › Figure 6-supplementary figure 2-data1/Figure_6-figure supplement_2_source_data_1_Figure_A_Liver_WT_ABT-199_cl-CASP7(Merge).jpg]

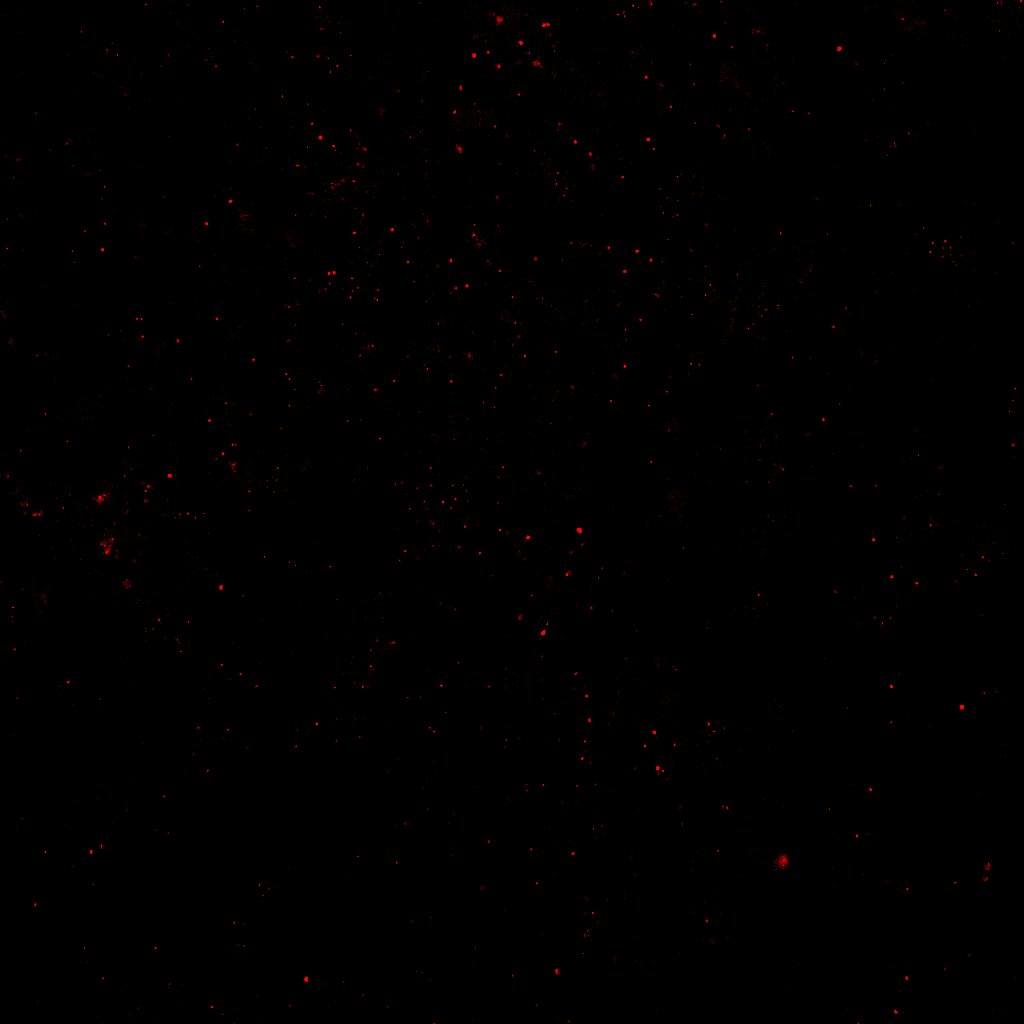

Supplement: Figure 6—figure supplement 2—source data 1. [file elife-98372-fig6-figsupp2-data1.zip › Figure 6-supplementary figure 2-data1/Figure_6-figure supplement_2_source_data_1_Figure_A_Liver_WT_cl-CASP7(cl-CASP7).jpg]

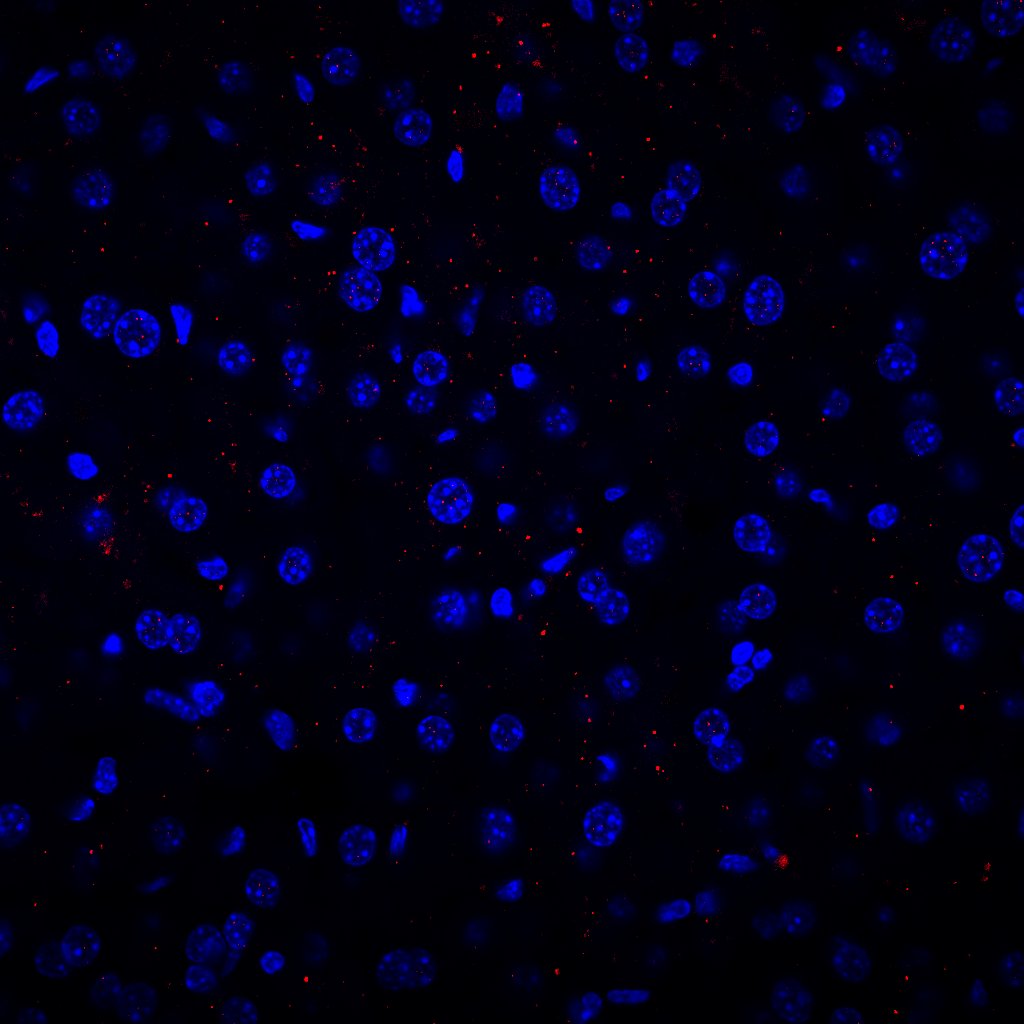

Supplement: Figure 6—figure supplement 2—source data 1. [file elife-98372-fig6-figsupp2-data1.zip › Figure 6-supplementary figure 2-data1/Figure_6-figure supplement_2_source_data_1_Figure_A_Liver_WT_cl-CASP7(Merge).jpg]

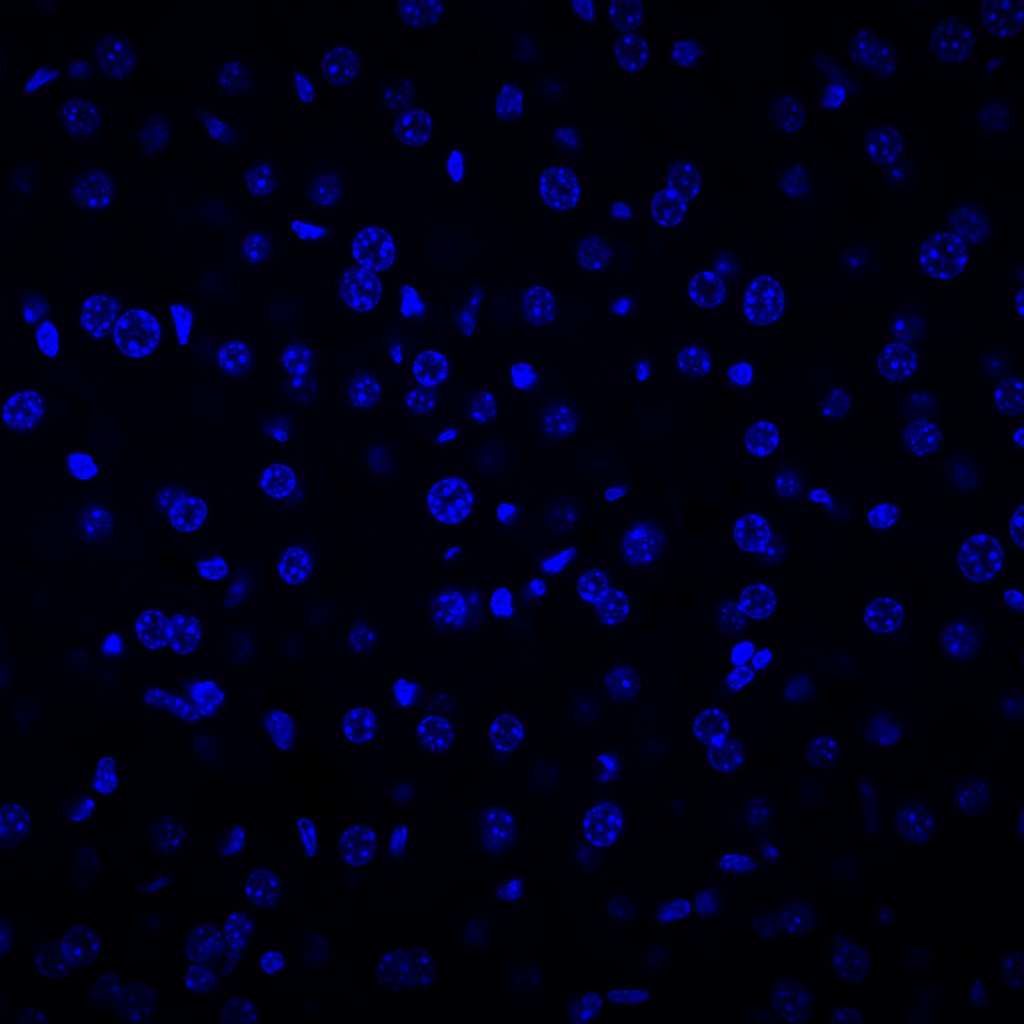

Supplement: Figure 6—figure supplement 2—source data 1. [file elife-98372-fig6-figsupp2-data1.zip › Figure 6-supplementary figure 2-data1/Figure_6-figure supplement_2_source_data_1_Figure_A_Liver_WT_cl-CASP7(DAPI).jpg]

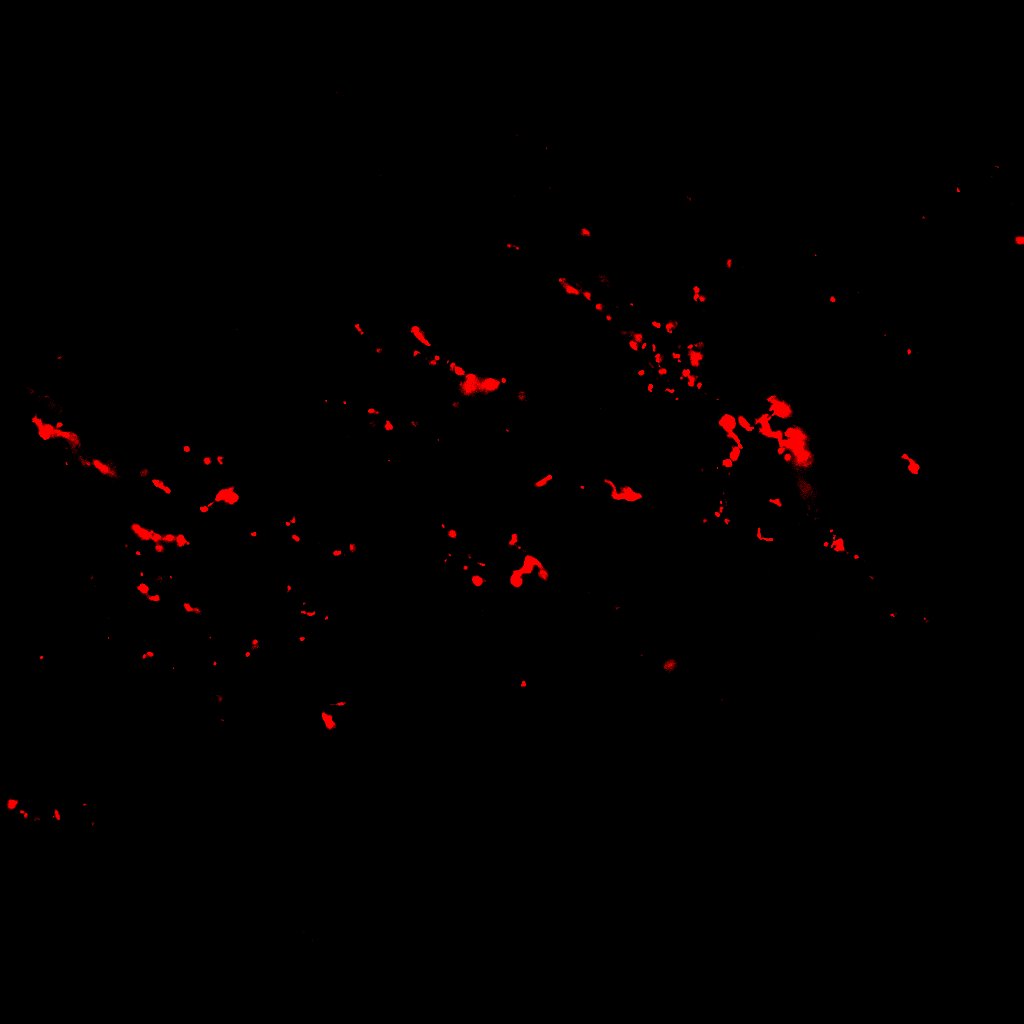

Supplement: Figure 6—figure supplement 2—source data 1. [file elife-98372-fig6-figsupp2-data1.zip › Figure 6-supplementary figure 2-data1/Figure_6-figure supplement_2_source_data_1_Figure_D_Heart_homo_ABT-199_cl-PARP1(cl-PARP1).jpg]

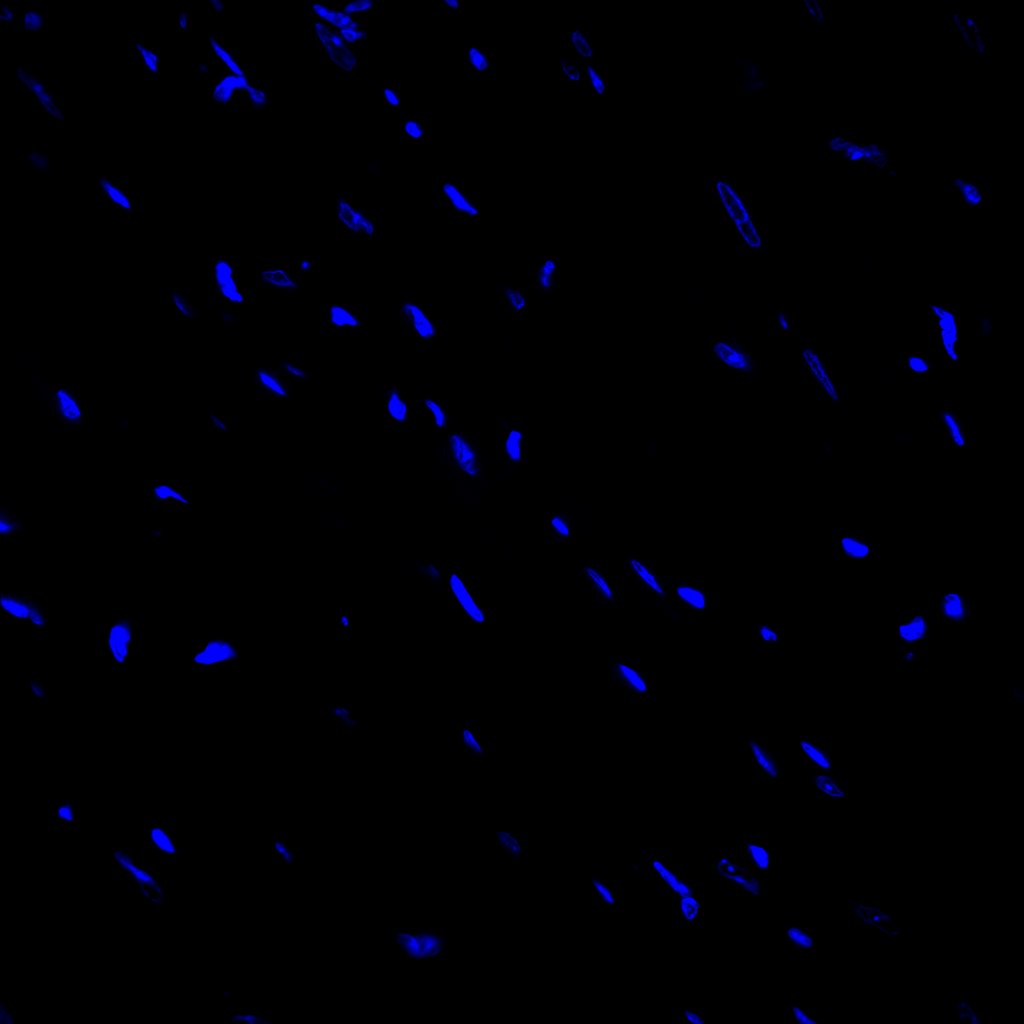

Supplement: Figure 6—figure supplement 2—source data 1. [file elife-98372-fig6-figsupp2-data1.zip › Figure 6-supplementary figure 2-data1/Figure_6-figure supplement_2_source_data_1_Figure_D_Heart_homo_ABT-199_cl-PARP1(DAPI).jpg]

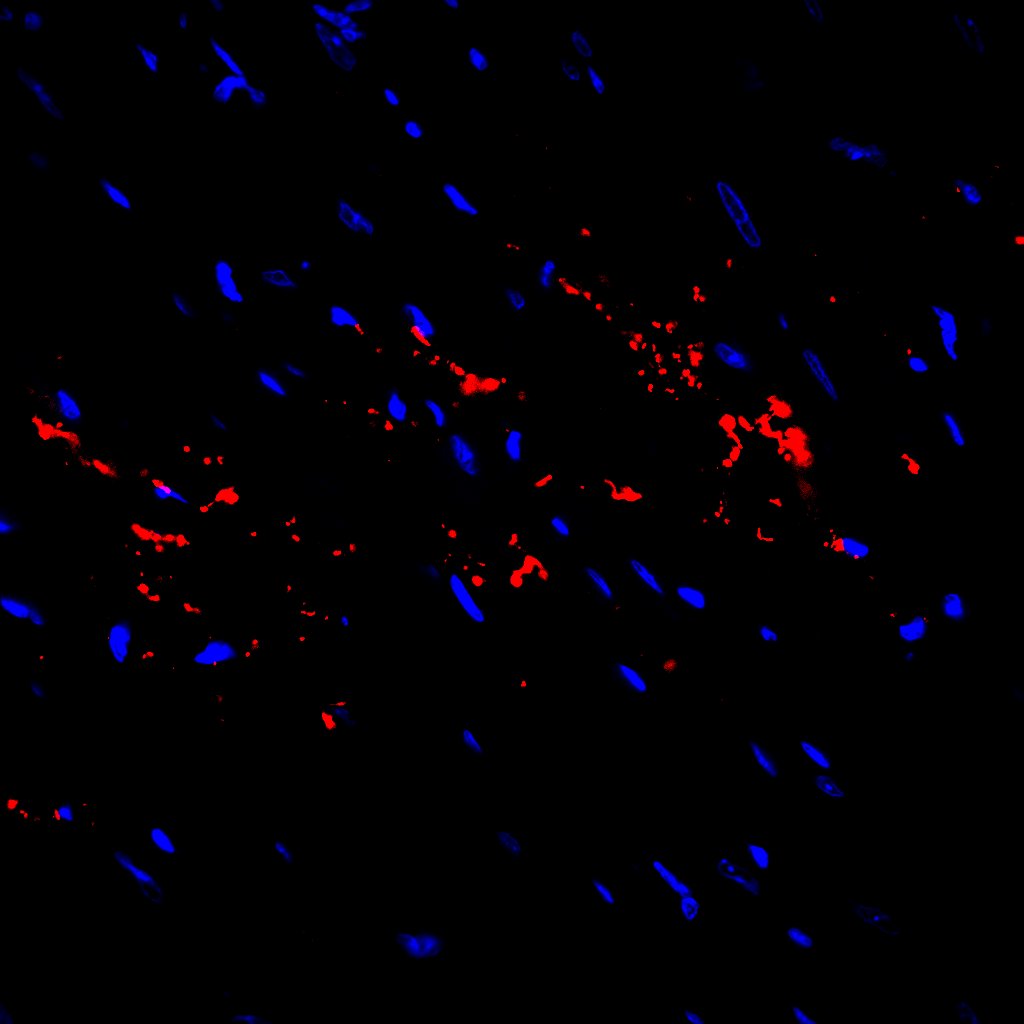

Supplement: Figure 6—figure supplement 2—source data 1. [file elife-98372-fig6-figsupp2-data1.zip › Figure 6-supplementary figure 2-data1/Figure_6-figure supplement_2_source_data_1_Figure_D_Heart_homo_ABT-199_cl-PARP1(Merge).jpg]

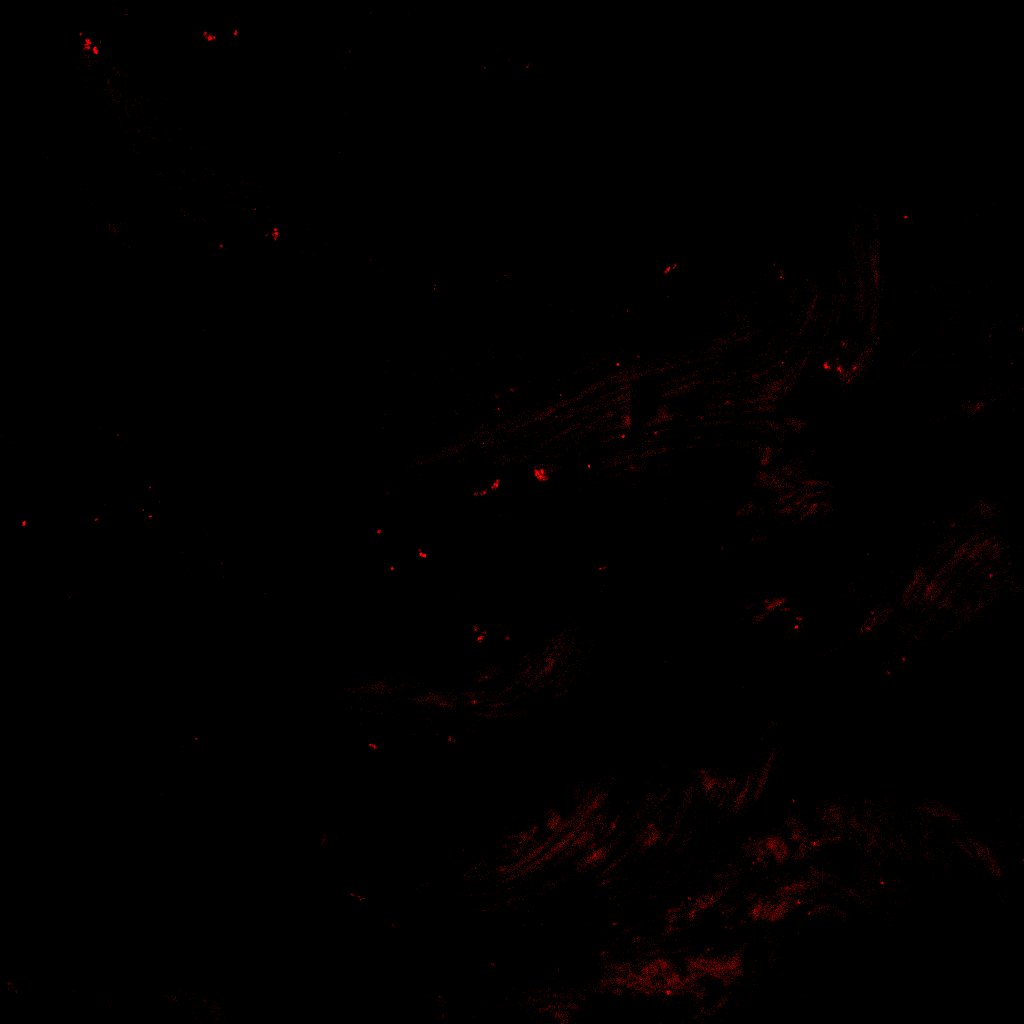

Supplement: Figure 6—figure supplement 2—source data 1. [file elife-98372-fig6-figsupp2-data1.zip › Figure 6-supplementary figure 2-data1/Figure_6-figure supplement_2_source_data_1_Figure_D_Heart_homo_cl-PARP1(cl-PARP1).jpg]

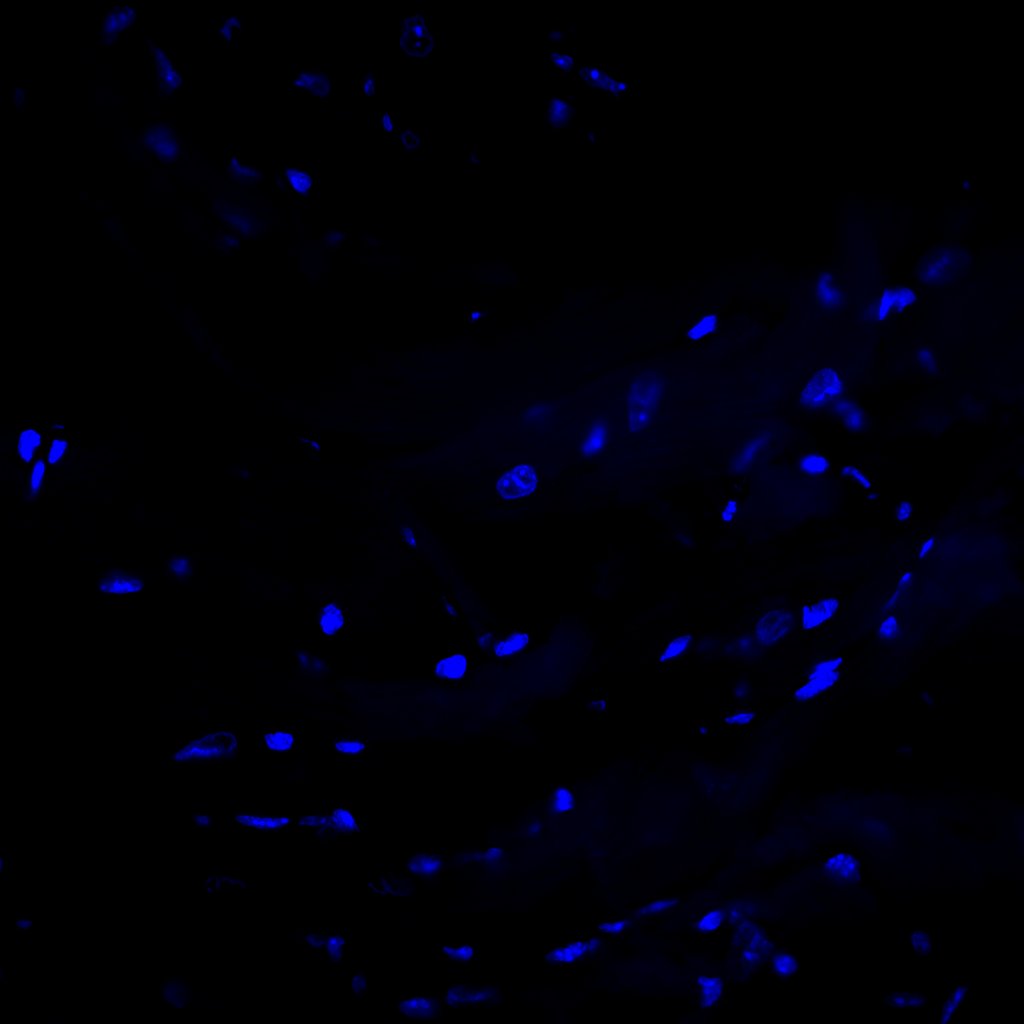

Supplement: Figure 6—figure supplement 2—source data 1. [file elife-98372-fig6-figsupp2-data1.zip › Figure 6-supplementary figure 2-data1/Figure_6-figure supplement_2_source_data_1_Figure_D_Heart_homo_cl-PARP1(DAPI).jpg]

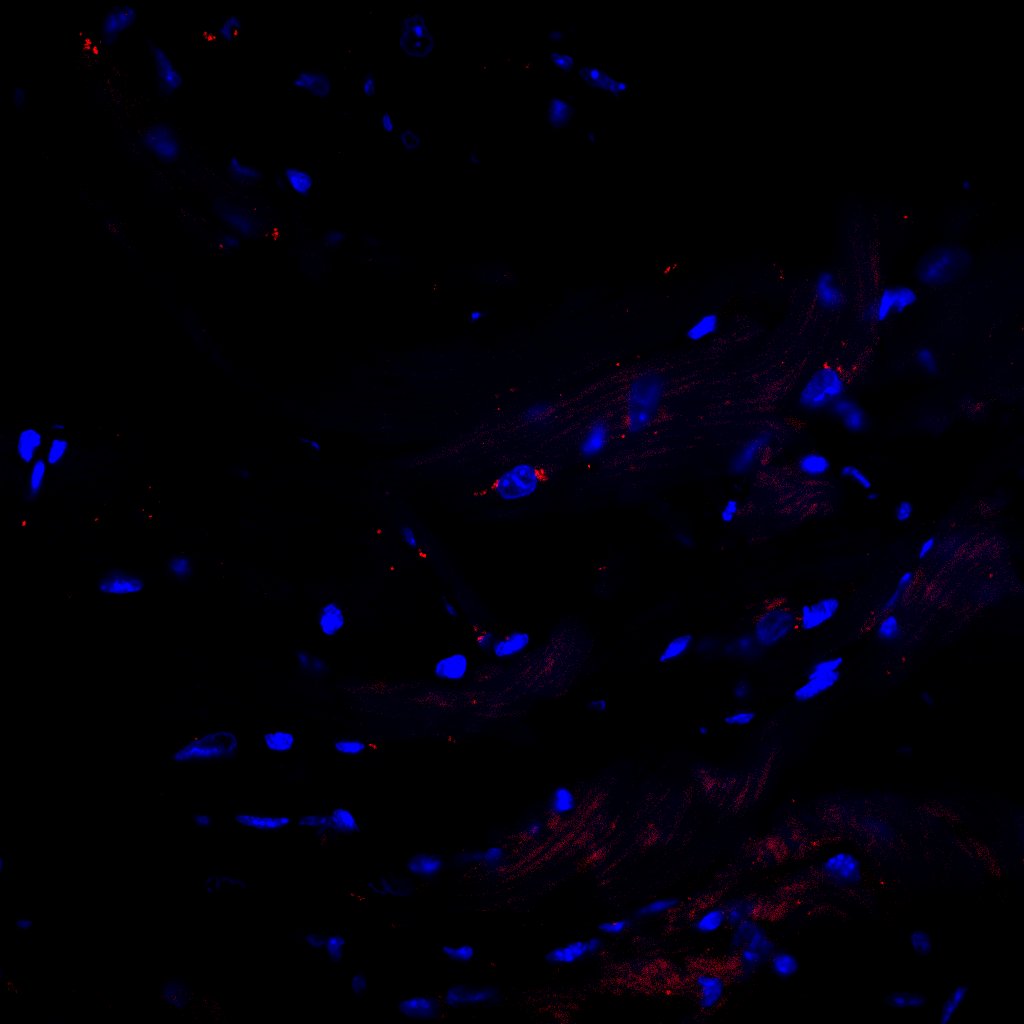

Supplement: Figure 6—figure supplement 2—source data 1. [file elife-98372-fig6-figsupp2-data1.zip › Figure 6-supplementary figure 2-data1/Figure_6-figure supplement_2_source_data_1_Figure_D_Heart_homo_cl-PARP1(Merge).jpg]

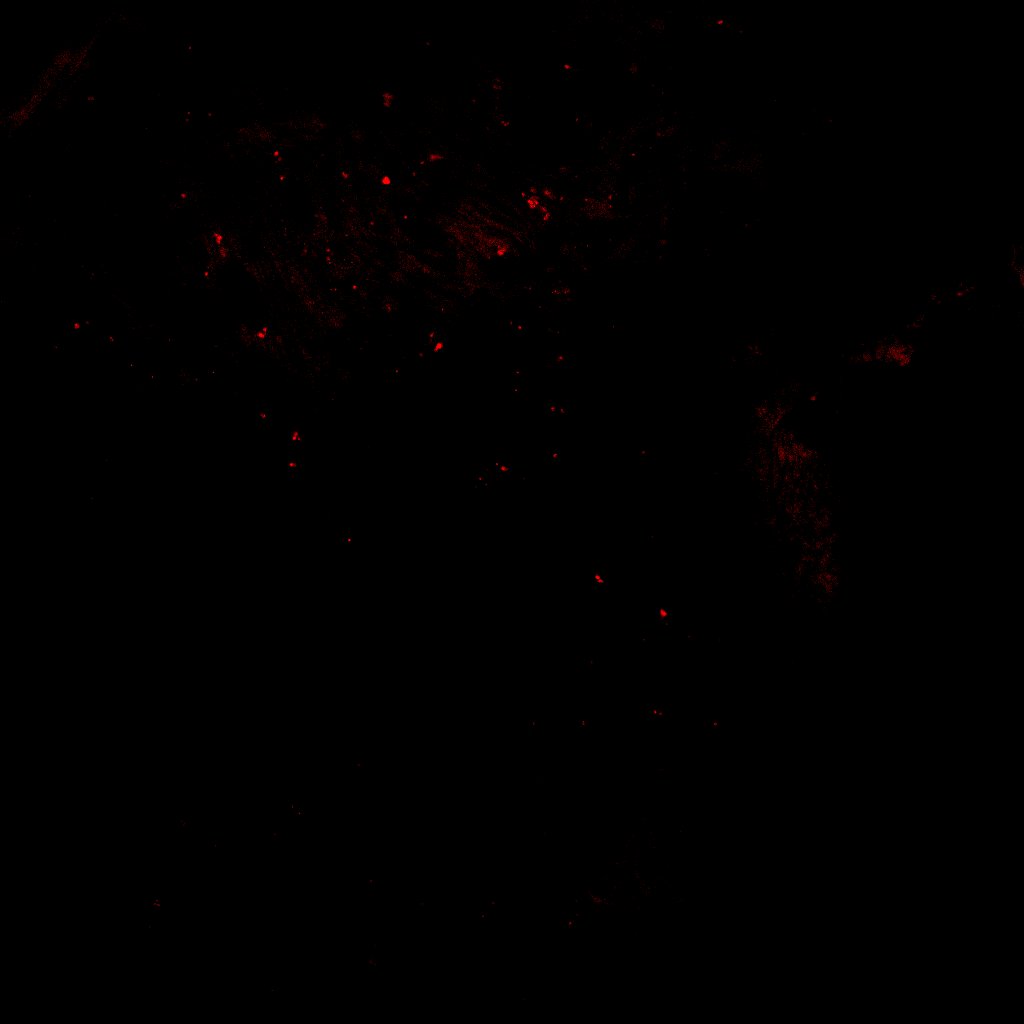

Supplement: Figure 6—figure supplement 2—source data 1. [file elife-98372-fig6-figsupp2-data1.zip › Figure 6-supplementary figure 2-data1/Figure_6-figure supplement_2_source_data_1_Figure_D_Heart_WT_ABT-199_cl-PARP1(cl-PARP1).jpg]
