# Supplementary figures and images for: Targeting the WSB2–NOXA axis in cancer cells for enhanced sensitivity to BCL-2 family protein inhibitors (part 5 of 5)
Source: eLife. 2025 Jul 23;13:RP98372. doi: 10.7554/eLife.98372 (PMC12286604; doi:10.7554/eLife.98372)

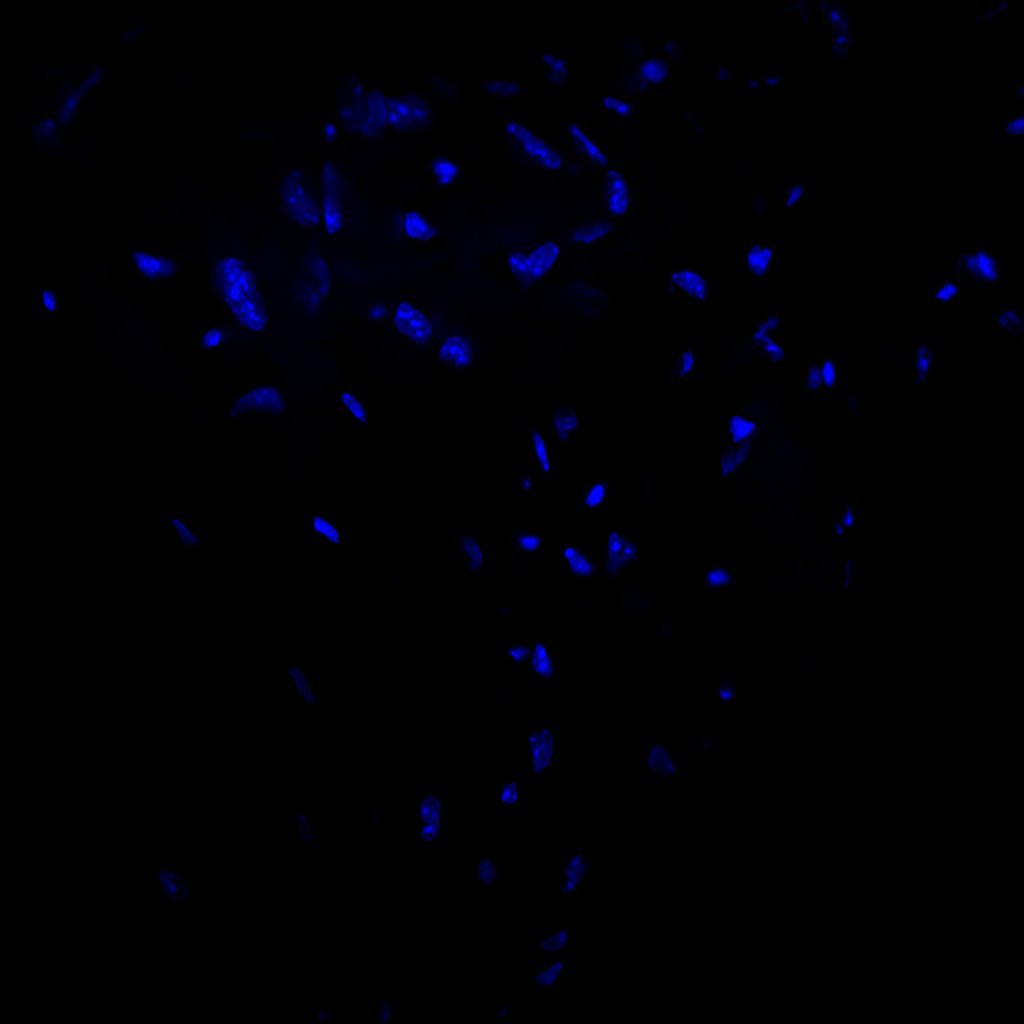

Supplement: Figure 6—figure supplement 2—source data 1. [file elife-98372-fig6-figsupp2-data1.zip › Figure 6-supplementary figure 2-data1/Figure_6-figure supplement_2_source_data_1_Figure_D_Heart_WT_ABT-199_cl-PARP1(DAPI).jpg]

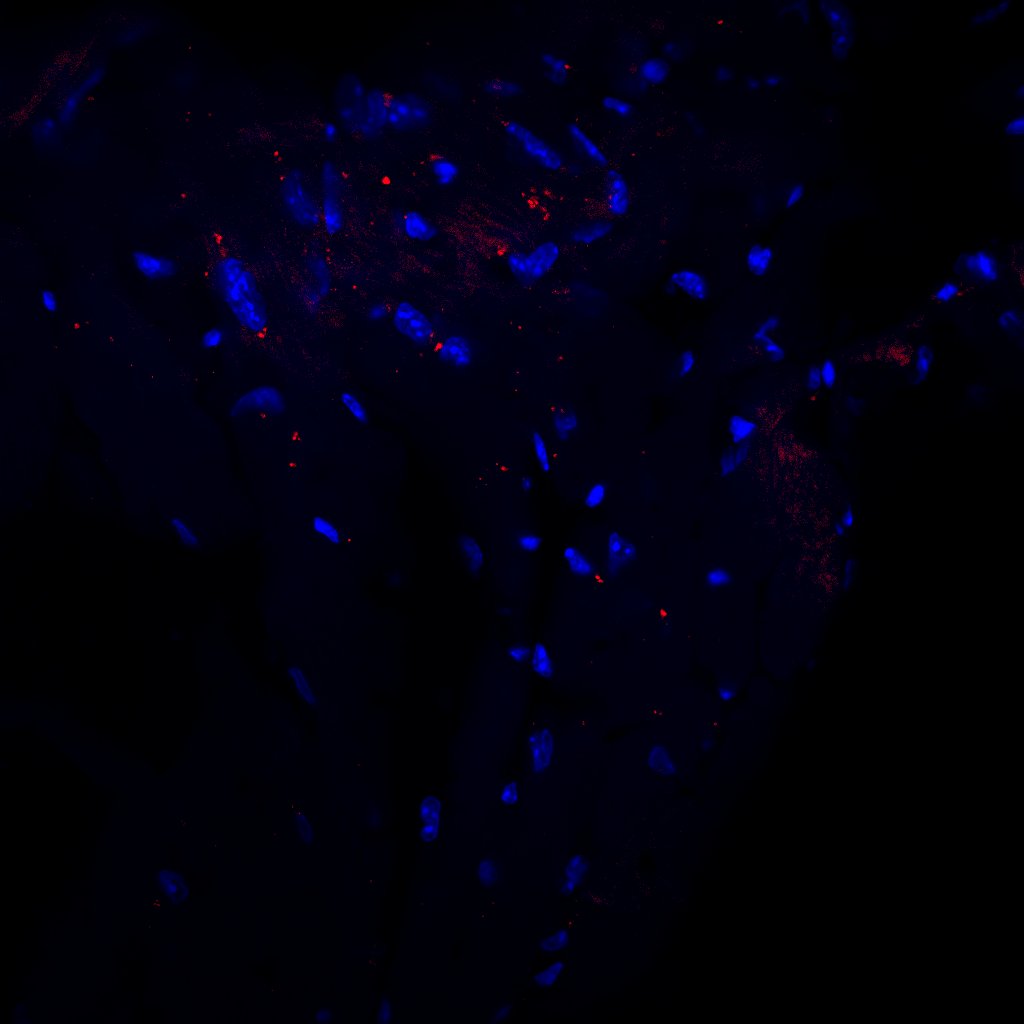

Supplement: Figure 6—figure supplement 2—source data 1. [file elife-98372-fig6-figsupp2-data1.zip › Figure 6-supplementary figure 2-data1/Figure_6-figure supplement_2_source_data_1_Figure_D_Heart_WT_ABT-199_cl-PARP1(Merge).jpg]

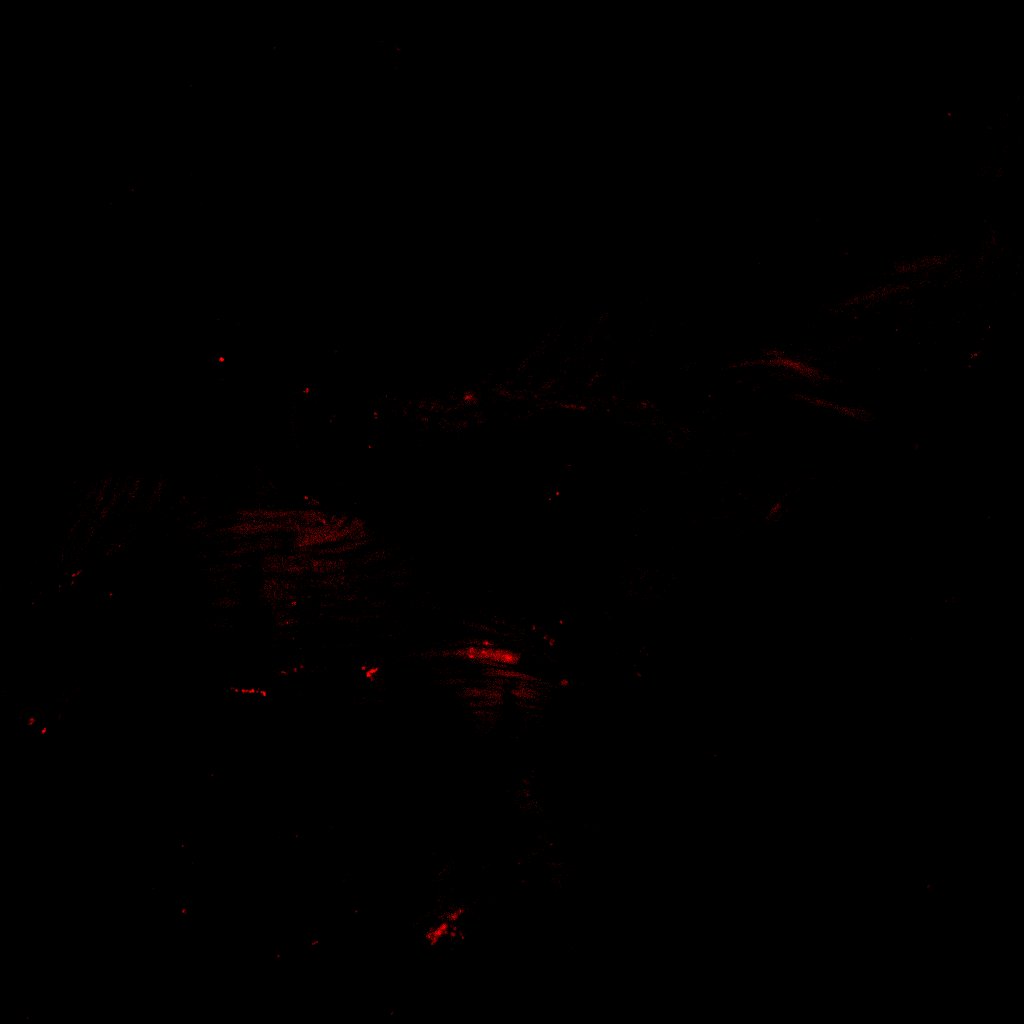

Supplement: Figure 6—figure supplement 2—source data 1. [file elife-98372-fig6-figsupp2-data1.zip › Figure 6-supplementary figure 2-data1/Figure_6-figure supplement_2_source_data_1_Figure_D_Heart_WT_cl-PARP1(cl-PARP1).jpg]

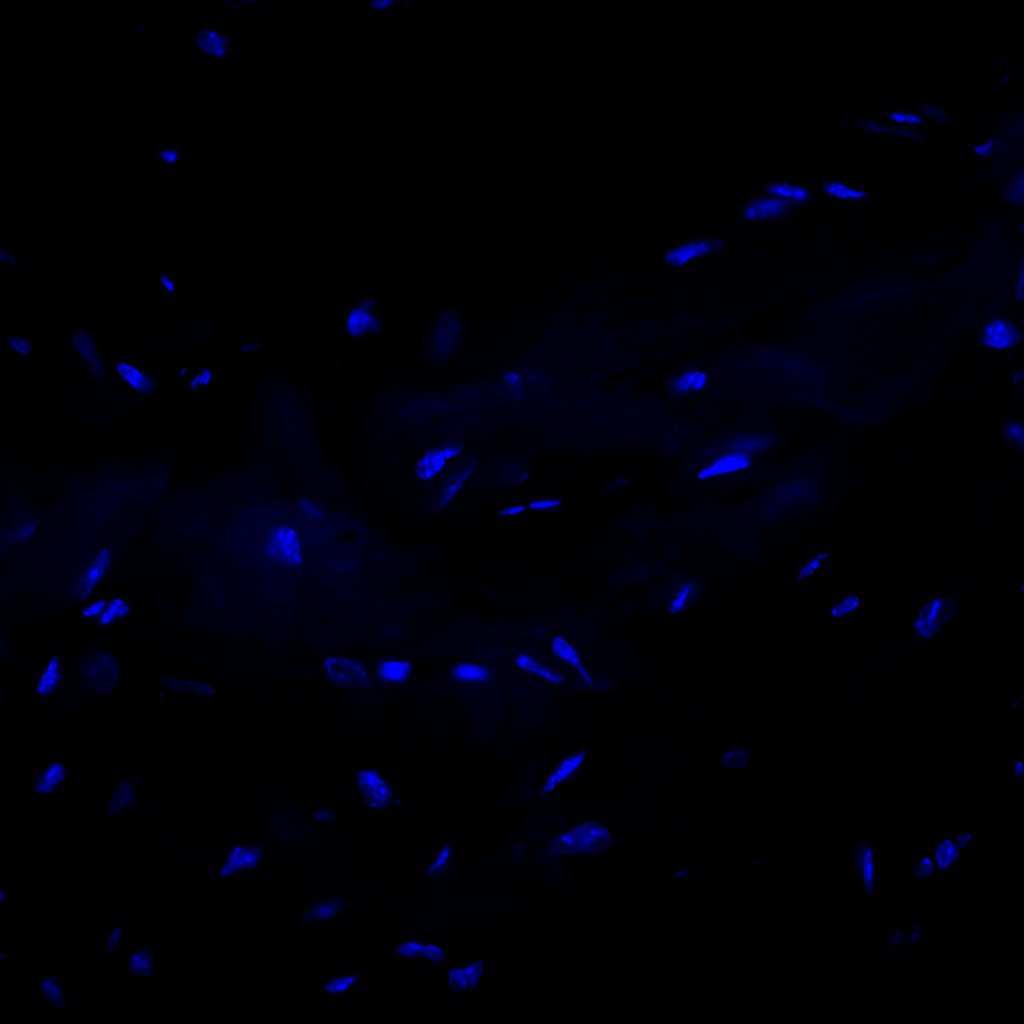

Supplement: Figure 6—figure supplement 2—source data 1. [file elife-98372-fig6-figsupp2-data1.zip › Figure 6-supplementary figure 2-data1/Figure_6-figure supplement_2_source_data_1_Figure_D_Heart_WT_cl-PARP1(DAPI).jpg]

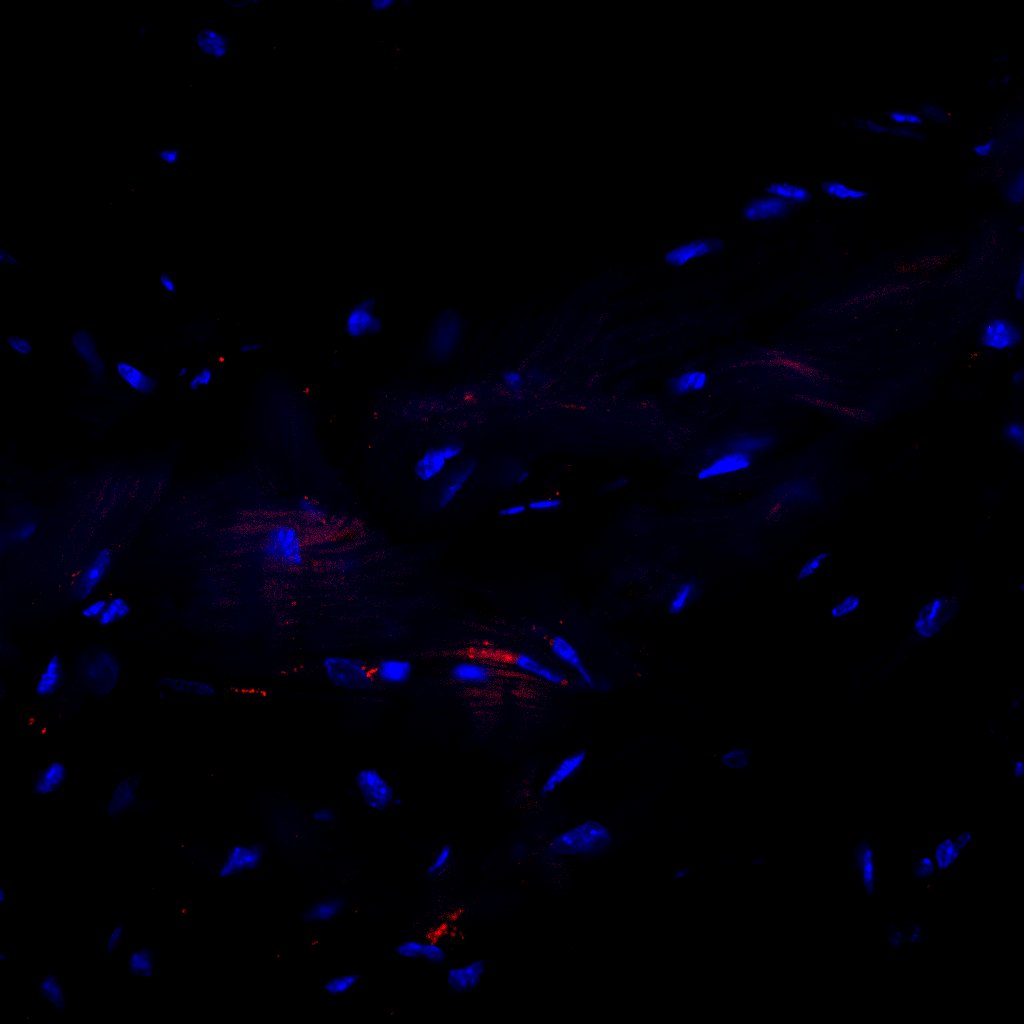

Supplement: Figure 6—figure supplement 2—source data 1. [file elife-98372-fig6-figsupp2-data1.zip › Figure 6-supplementary figure 2-data1/Figure_6-figure supplement_2_source_data_1_Figure_D_Heart_WT_cl-PARP1(Merge).jpg]

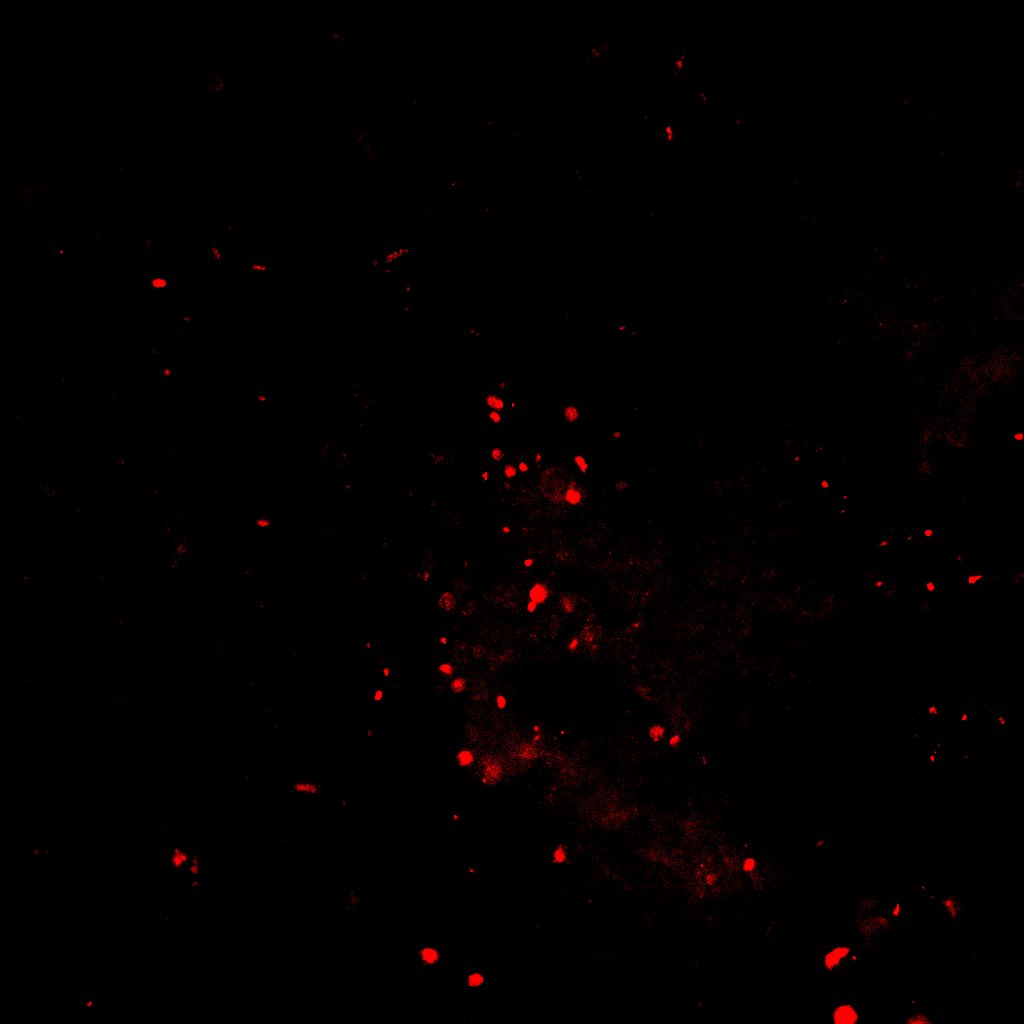

Supplement: Figure 6—figure supplement 2—source data 1. [file elife-98372-fig6-figsupp2-data1.zip › Figure 6-supplementary figure 2-data1/Figure_6-figure supplement_2_source_data_1_Figure_D_Liver_homo_ABT-199_cl-PARP1(cl-PARP1).jpg]

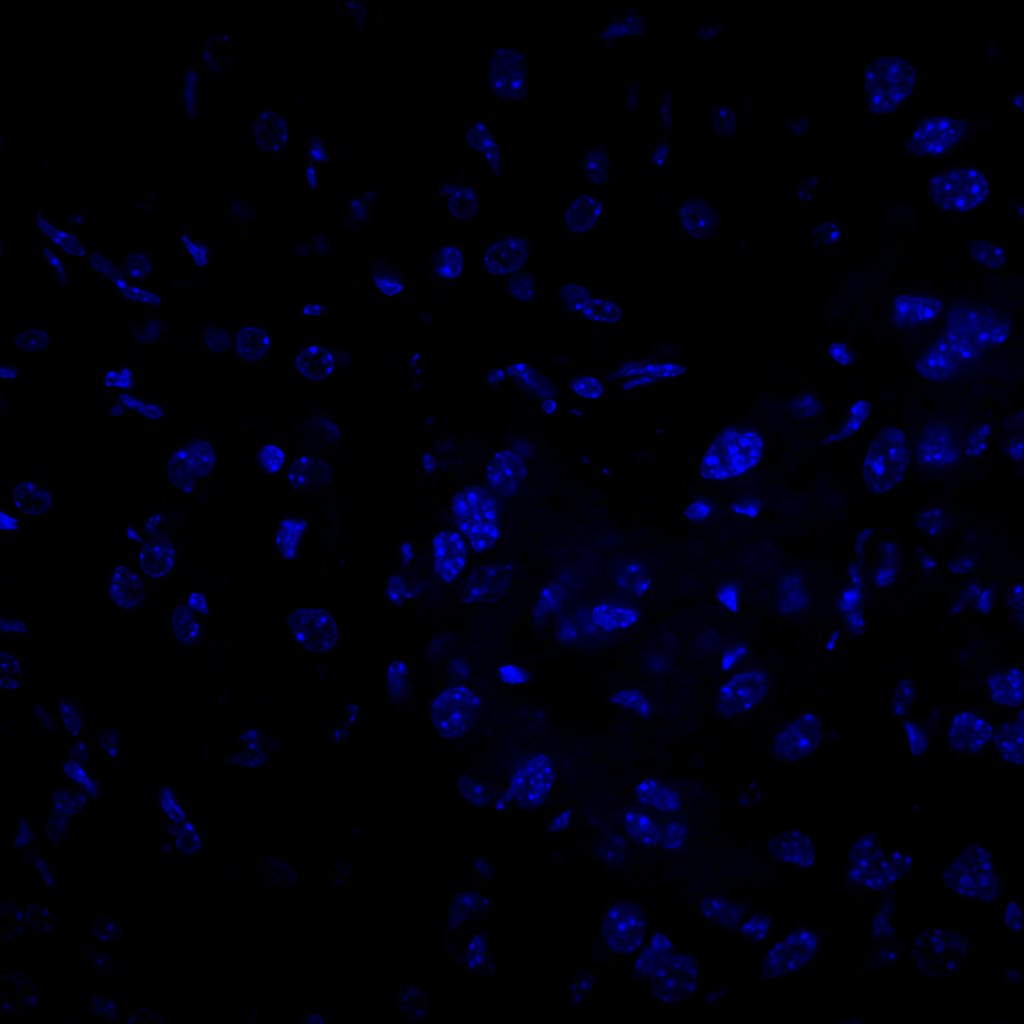

Supplement: Figure 6—figure supplement 2—source data 1. [file elife-98372-fig6-figsupp2-data1.zip › Figure 6-supplementary figure 2-data1/Figure_6-figure supplement_2_source_data_1_Figure_D_Liver_homo_ABT-199_cl-PARP1(DAPI).jpg]

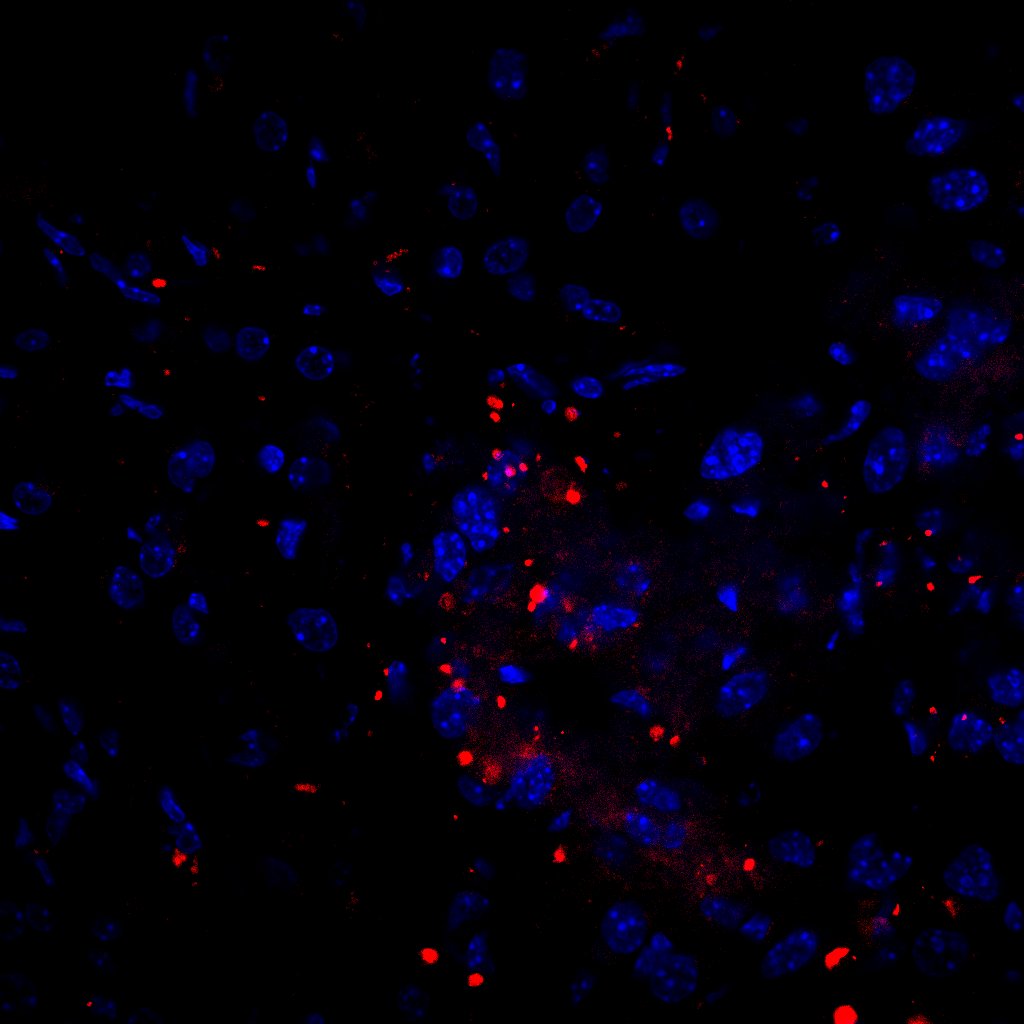

Supplement: Figure 6—figure supplement 2—source data 1. [file elife-98372-fig6-figsupp2-data1.zip › Figure 6-supplementary figure 2-data1/Figure_6-figure supplement_2_source_data_1_Figure_D_Liver_homo_ABT-199_cl-PARP1(Merge).jpg]

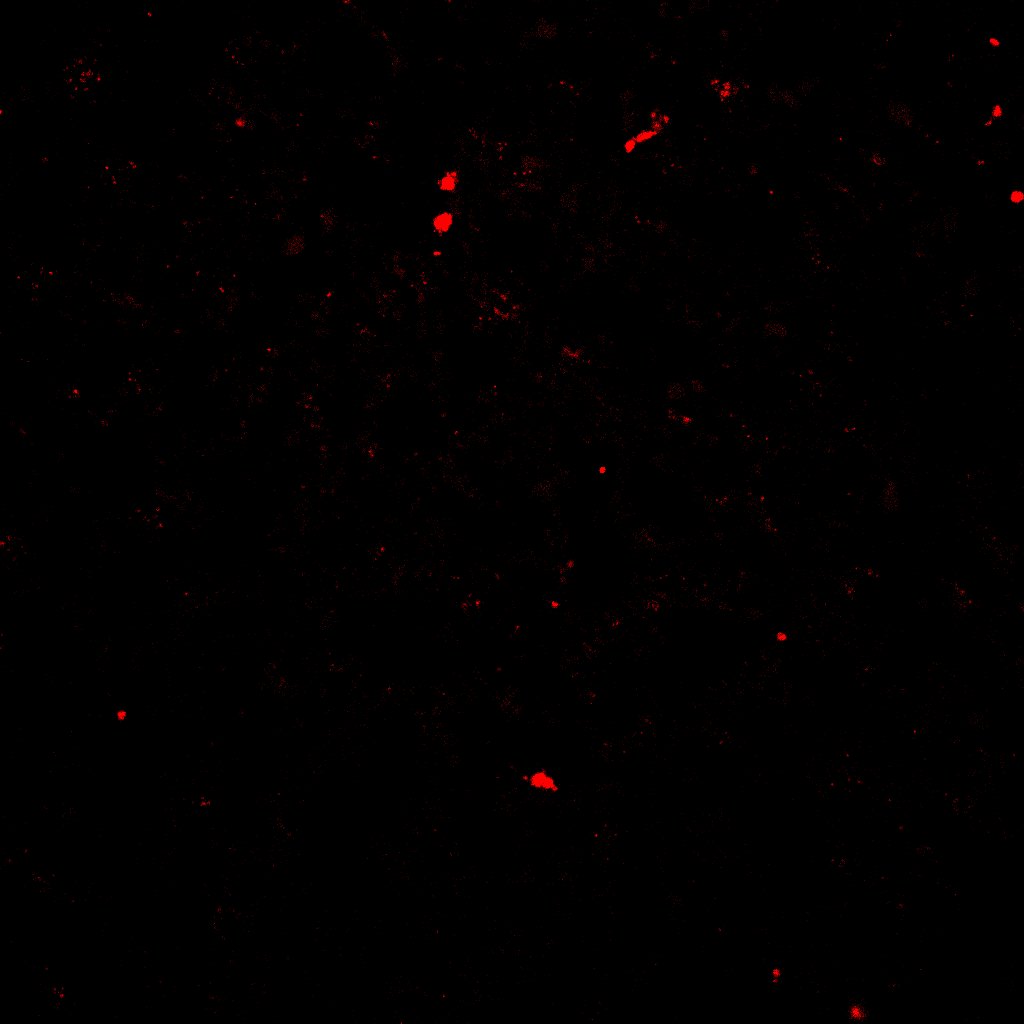

Supplement: Figure 6—figure supplement 2—source data 1. [file elife-98372-fig6-figsupp2-data1.zip › Figure 6-supplementary figure 2-data1/Figure_6-figure supplement_2_source_data_1_Figure_D_Liver_homo_cl-PARP1(cl-PARP1).jpg]

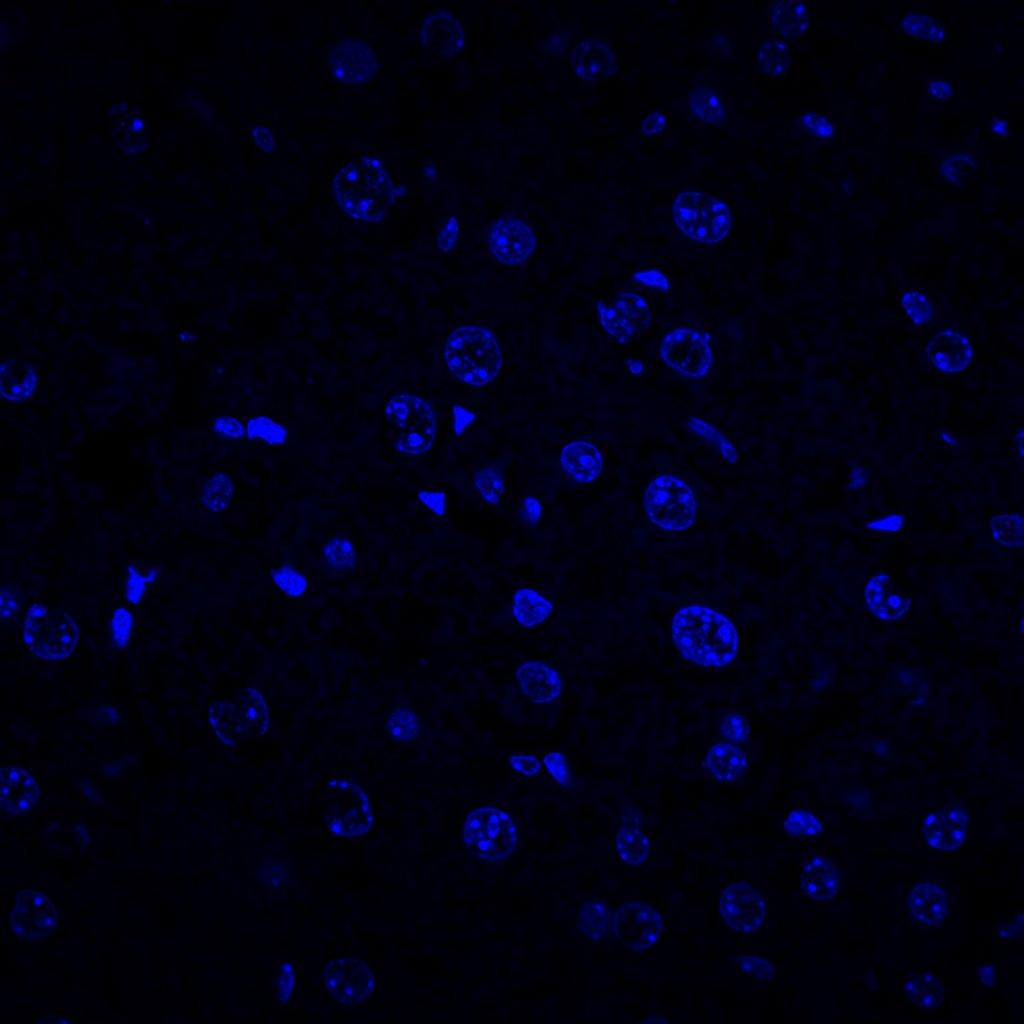

Supplement: Figure 6—figure supplement 2—source data 1. [file elife-98372-fig6-figsupp2-data1.zip › Figure 6-supplementary figure 2-data1/Figure_6-figure supplement_2_source_data_1_Figure_D_Liver_homo_cl-PARP1(DAPI).jpg]

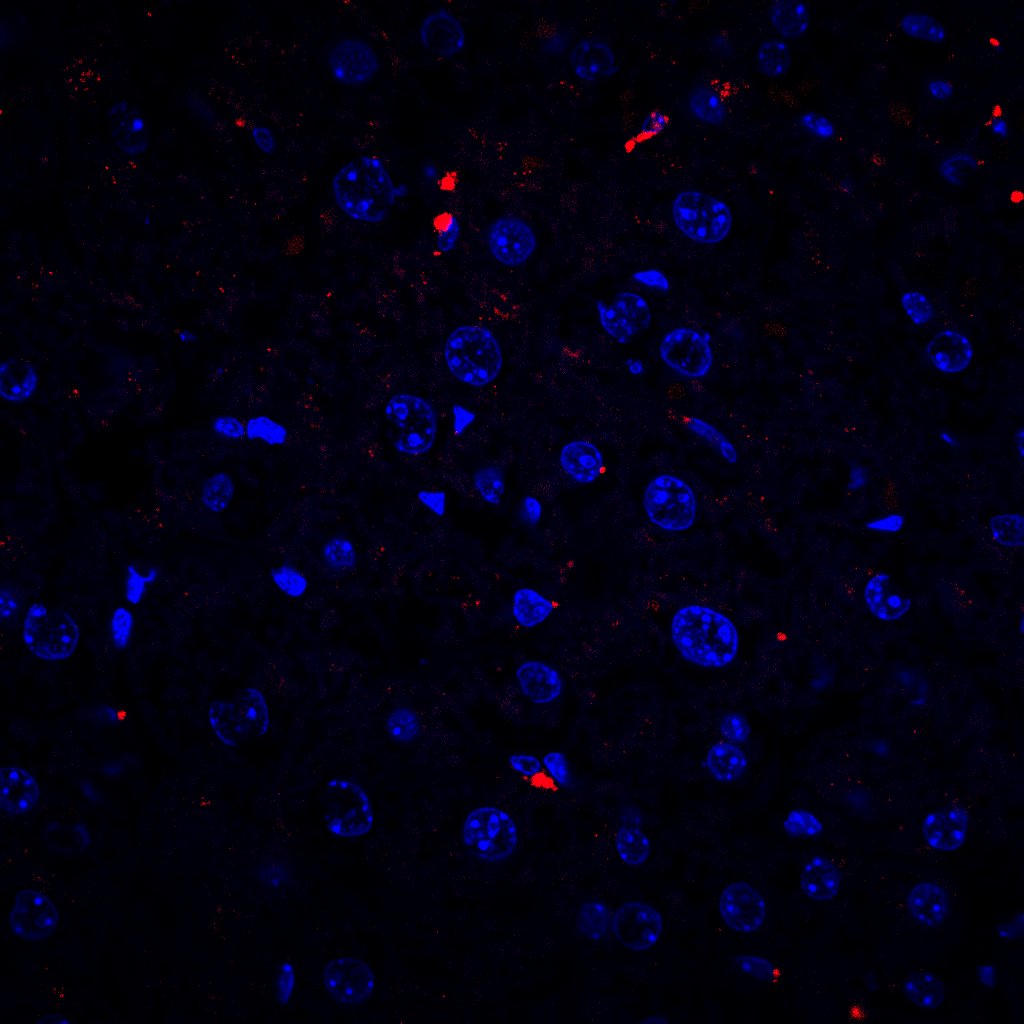

Supplement: Figure 6—figure supplement 2—source data 1. [file elife-98372-fig6-figsupp2-data1.zip › Figure 6-supplementary figure 2-data1/Figure_6-figure supplement_2_source_data_1_Figure_D_Liver_homo_cl-PARP1(Merge).jpg]

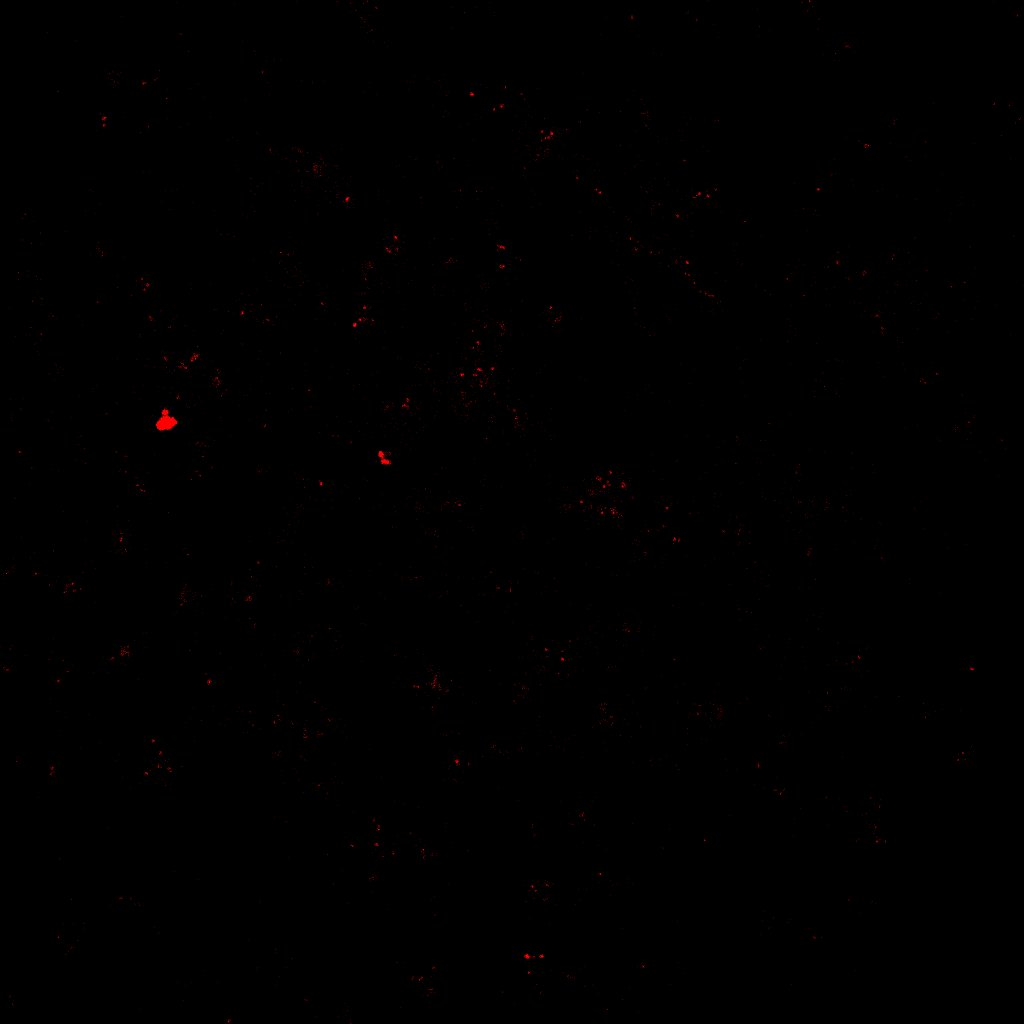

Supplement: Figure 6—figure supplement 2—source data 1. [file elife-98372-fig6-figsupp2-data1.zip › Figure 6-supplementary figure 2-data1/Figure_6-figure supplement_2_source_data_1_Figure_D_Liver_WT_ABT-199_cl-PARP1(cl-PARP1).jpg]

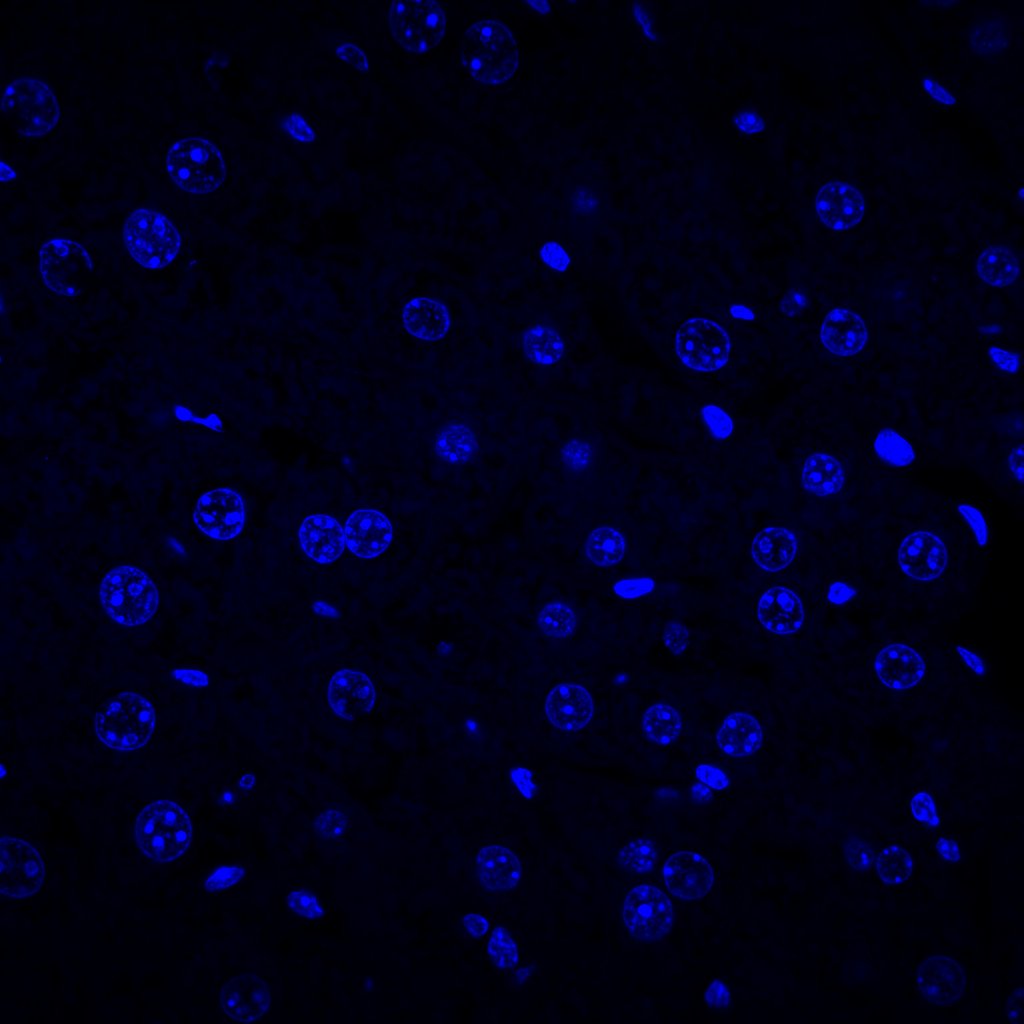

Supplement: Figure 6—figure supplement 2—source data 1. [file elife-98372-fig6-figsupp2-data1.zip › Figure 6-supplementary figure 2-data1/Figure_6-figure supplement_2_source_data_1_Figure_D_Liver_WT_ABT-199_cl-PARP1(DAPI).jpg]

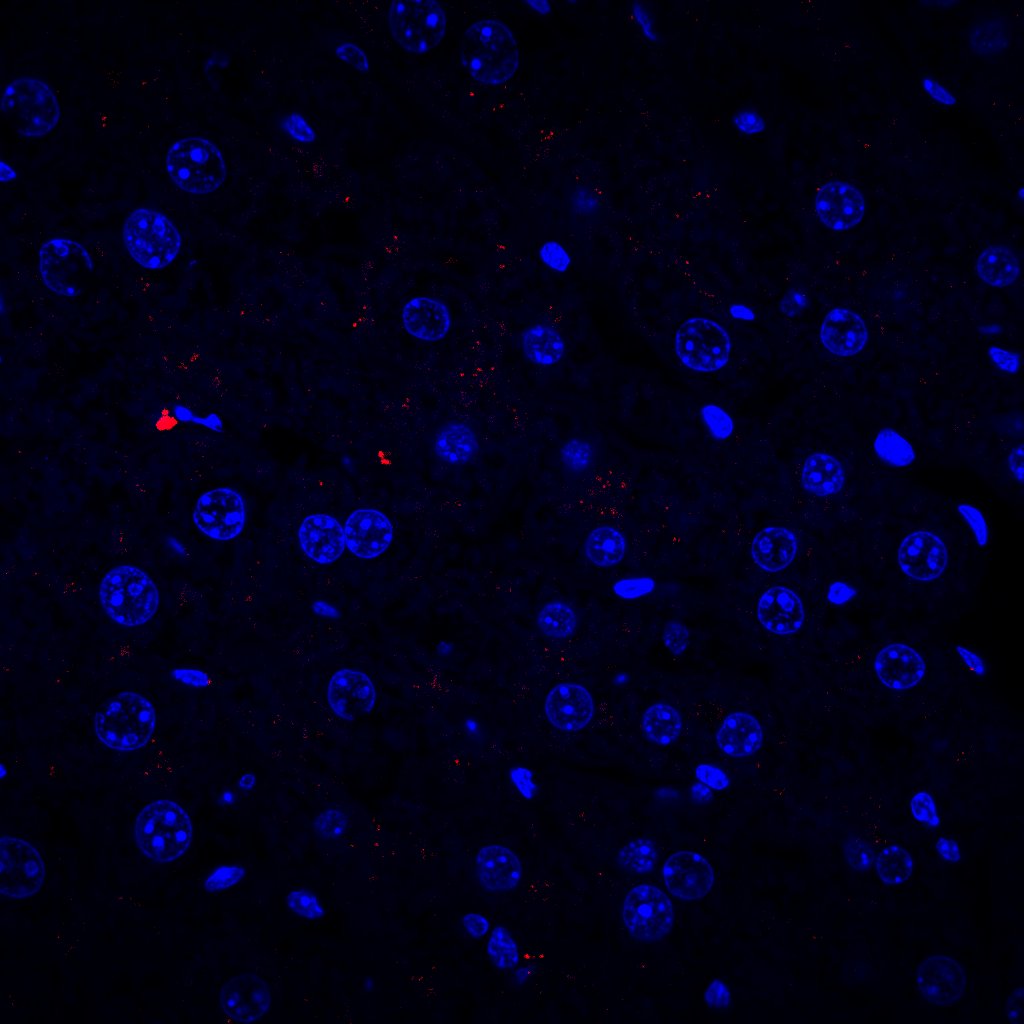

Supplement: Figure 6—figure supplement 2—source data 1. [file elife-98372-fig6-figsupp2-data1.zip › Figure 6-supplementary figure 2-data1/Figure_6-figure supplement_2_source_data_1_Figure_D_Liver_WT_ABT-199_cl-PARP1(Merge).jpg]

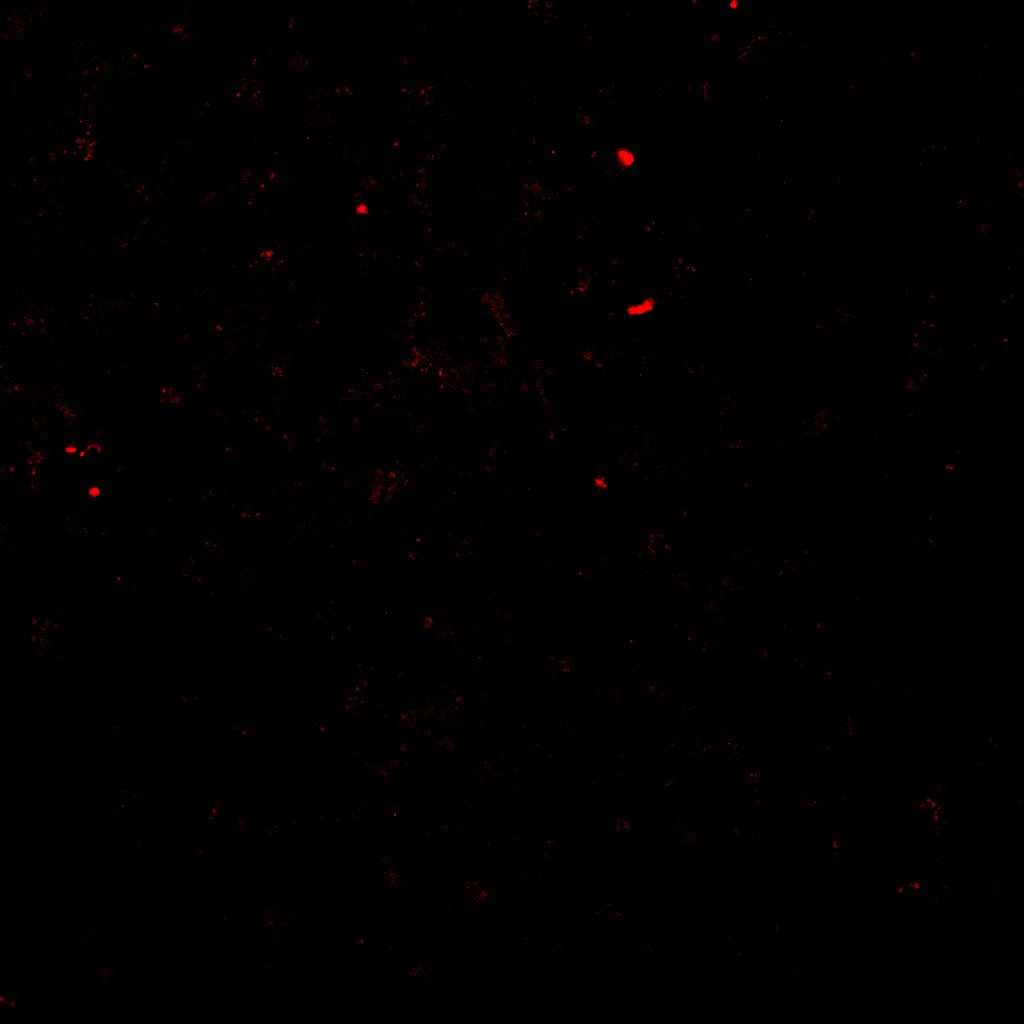

Supplement: Figure 6—figure supplement 2—source data 1. [file elife-98372-fig6-figsupp2-data1.zip › Figure 6-supplementary figure 2-data1/Figure_6-figure supplement_2_source_data_1_Figure_D_Liver_WT_cl-PARP1(cl-PARP1).jpg]

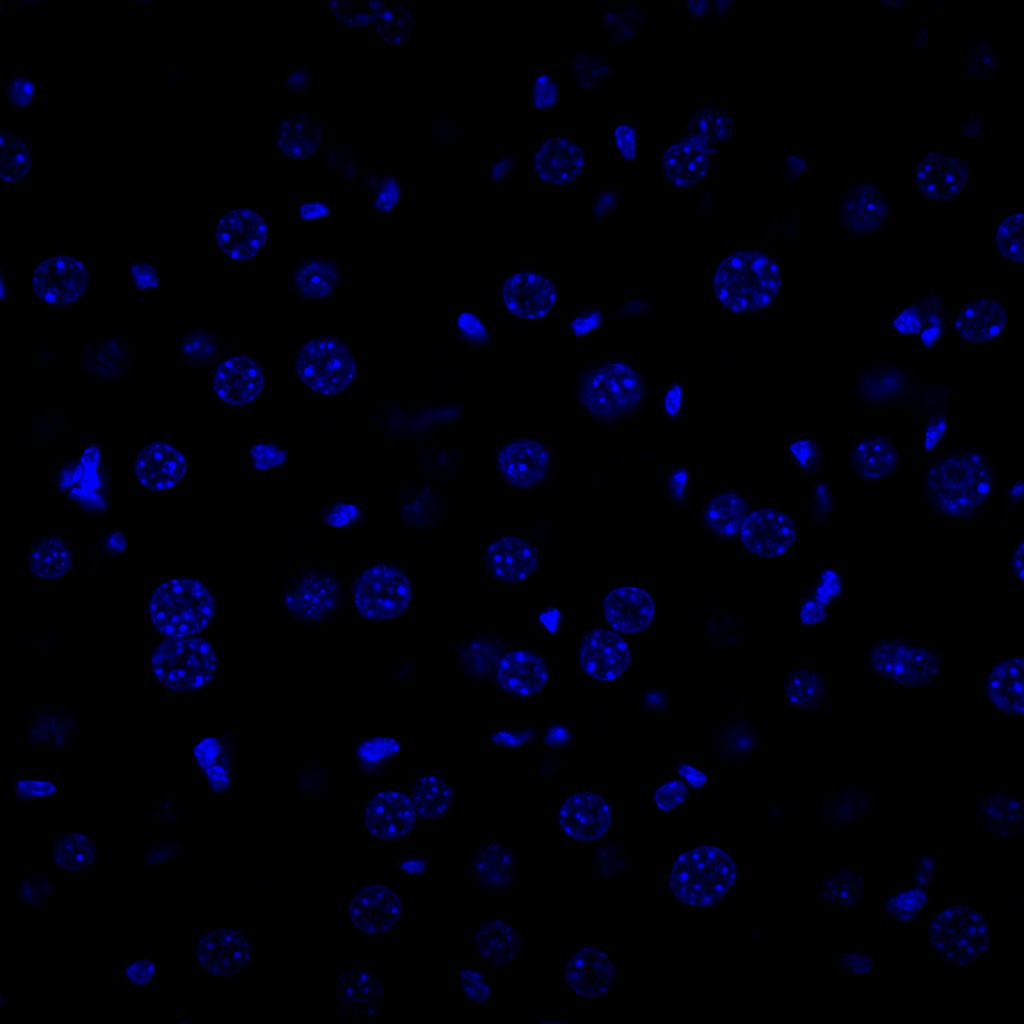

Supplement: Figure 6—figure supplement 2—source data 1. [file elife-98372-fig6-figsupp2-data1.zip › Figure 6-supplementary figure 2-data1/Figure_6-figure supplement_2_source_data_1_Figure_D_Liver_WT_cl-PARP1(DAPI).jpg]

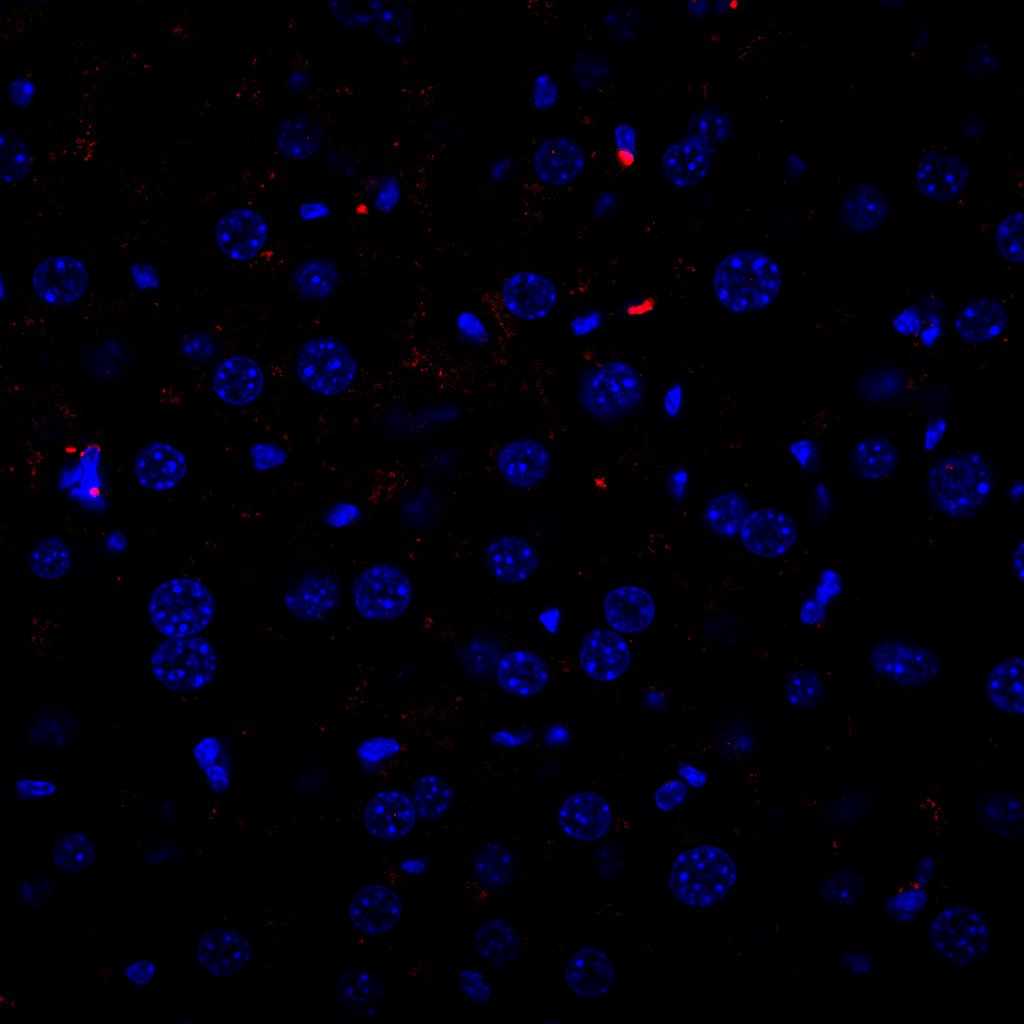

Supplement: Figure 6—figure supplement 2—source data 1. [file elife-98372-fig6-figsupp2-data1.zip › Figure 6-supplementary figure 2-data1/Figure_6-figure supplement_2_source_data_1_Figure_D_Liver_WT_cl-PARP1(Merge).jpg]

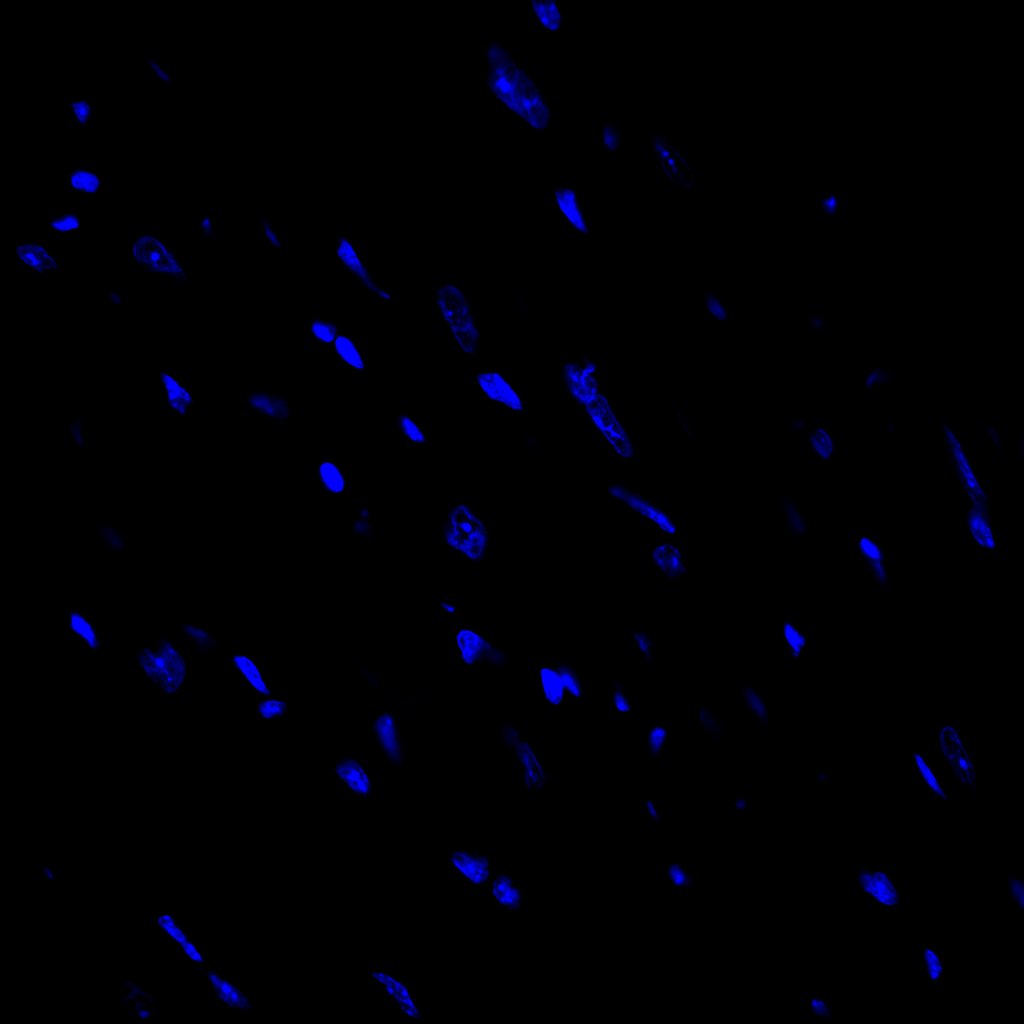

Supplement: Figure 6—figure supplement 2—source data 1. [file elife-98372-fig6-figsupp2-data1.zip › Figure 6-supplementary figure 2-data1/Figure_6-figure supplement_2_source_data_1_Figure_G_Heart_homo_ABT-199_TUNEL(DAPI).jpg]

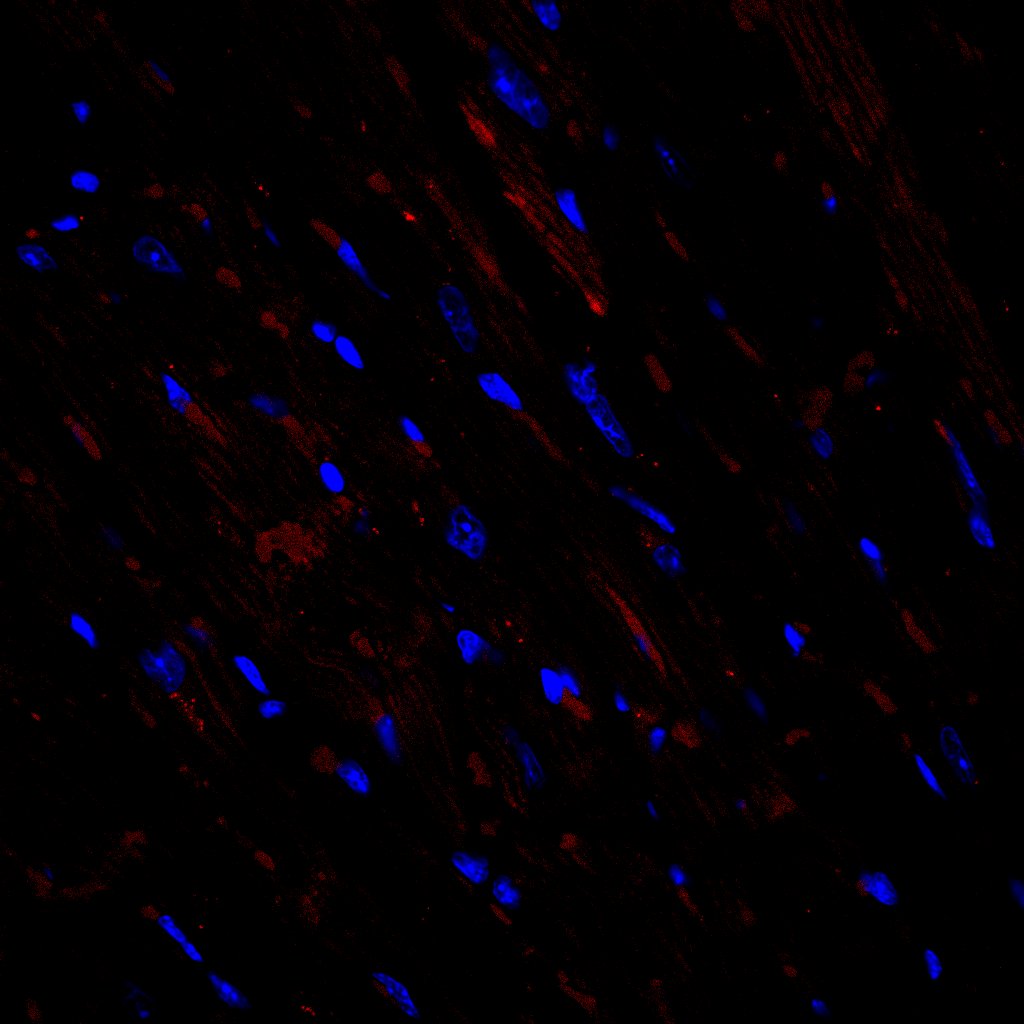

Supplement: Figure 6—figure supplement 2—source data 1. [file elife-98372-fig6-figsupp2-data1.zip › Figure 6-supplementary figure 2-data1/Figure_6-figure supplement_2_source_data_1_Figure_G_Heart_homo_ABT-199_TUNEL(Merge).jpg]

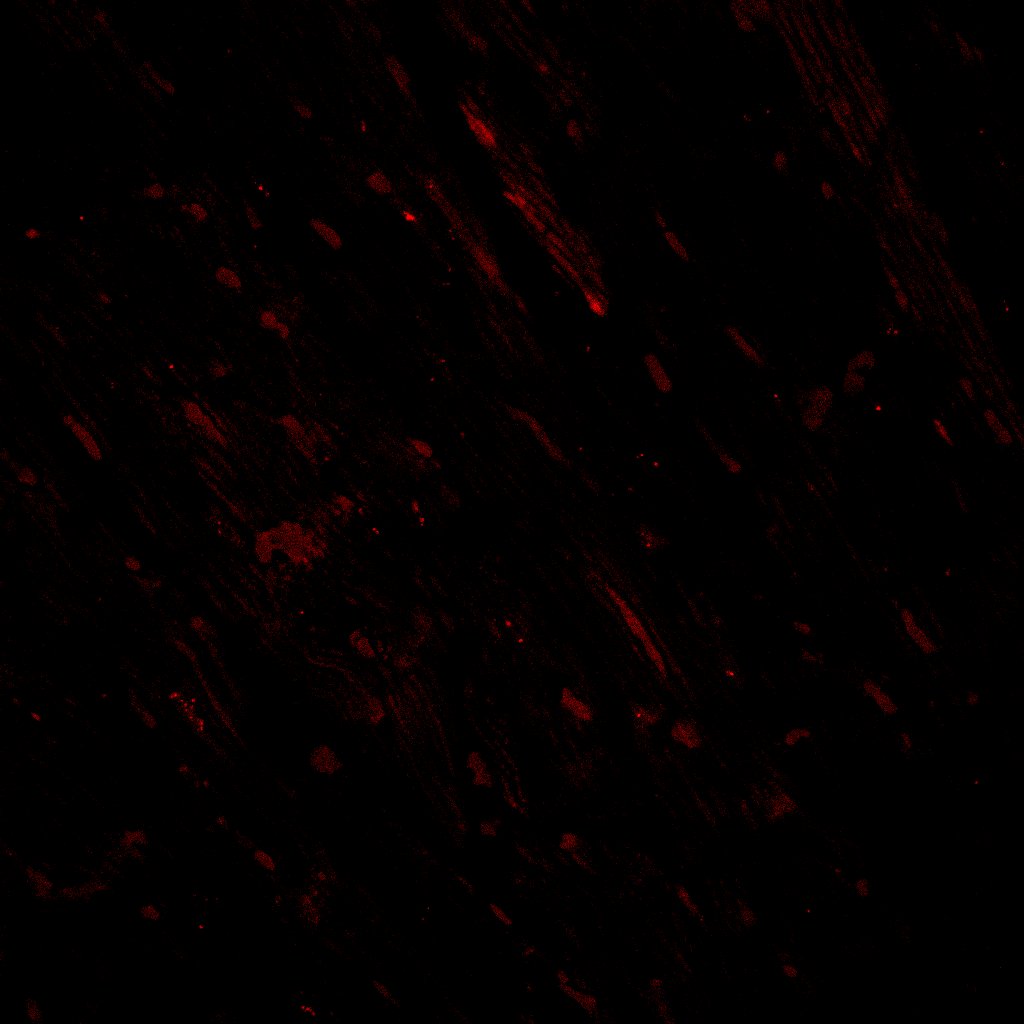

Supplement: Figure 6—figure supplement 2—source data 1. [file elife-98372-fig6-figsupp2-data1.zip › Figure 6-supplementary figure 2-data1/Figure_6-figure supplement_2_source_data_1_Figure_G_Heart_homo_ABT-199_TUNEL(TUNEL).jpg]

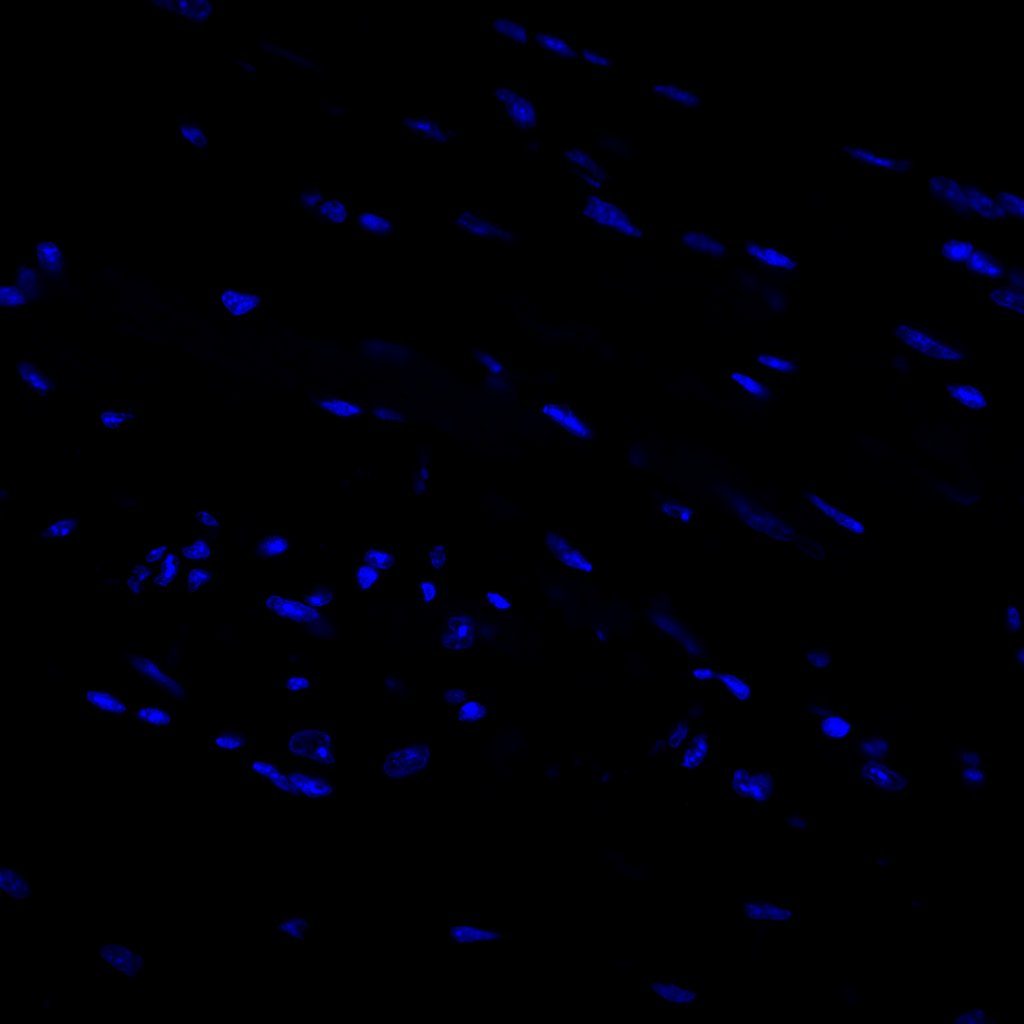

Supplement: Figure 6—figure supplement 2—source data 1. [file elife-98372-fig6-figsupp2-data1.zip › Figure 6-supplementary figure 2-data1/Figure_6-figure supplement_2_source_data_1_Figure_G_Heart_homo_TUNEL(DAPI).jpg]

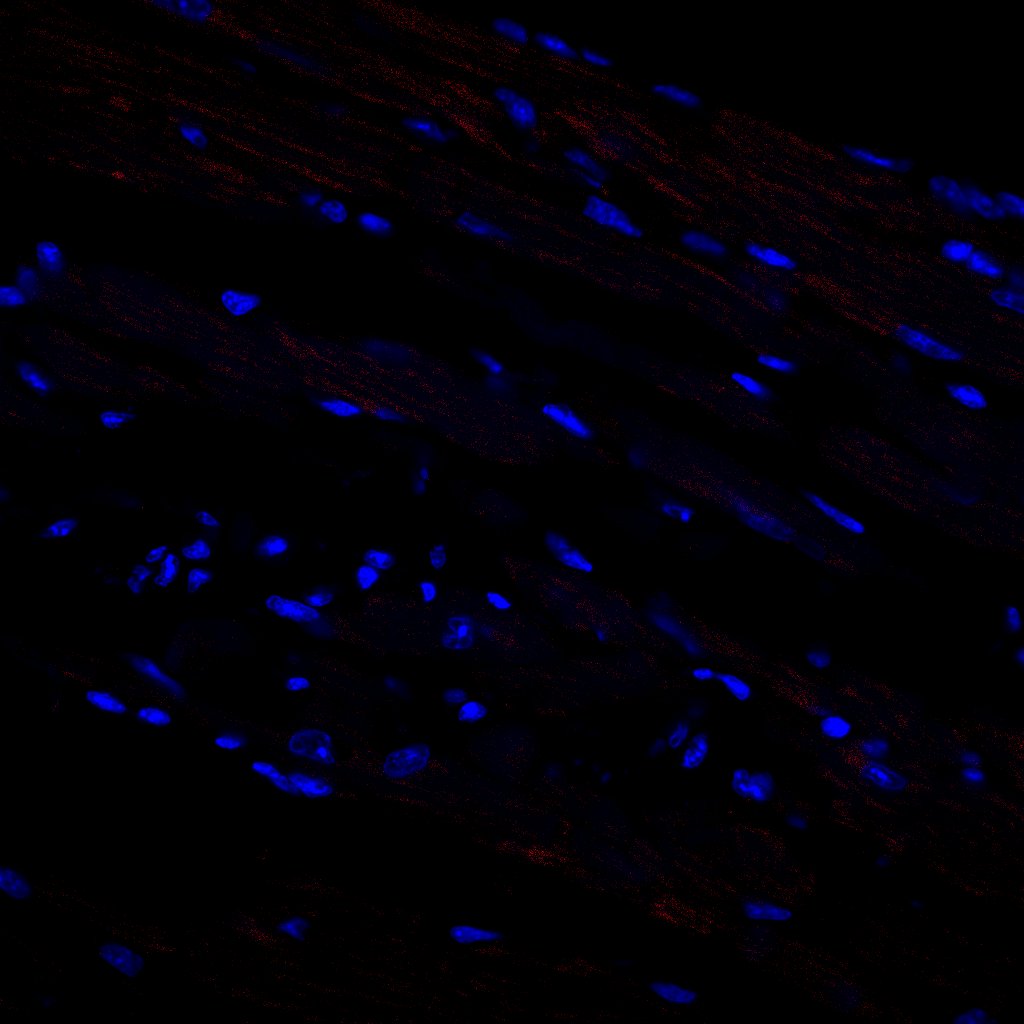

Supplement: Figure 6—figure supplement 2—source data 1. [file elife-98372-fig6-figsupp2-data1.zip › Figure 6-supplementary figure 2-data1/Figure_6-figure supplement_2_source_data_1_Figure_G_Heart_homo_TUNEL(Merge).jpg]

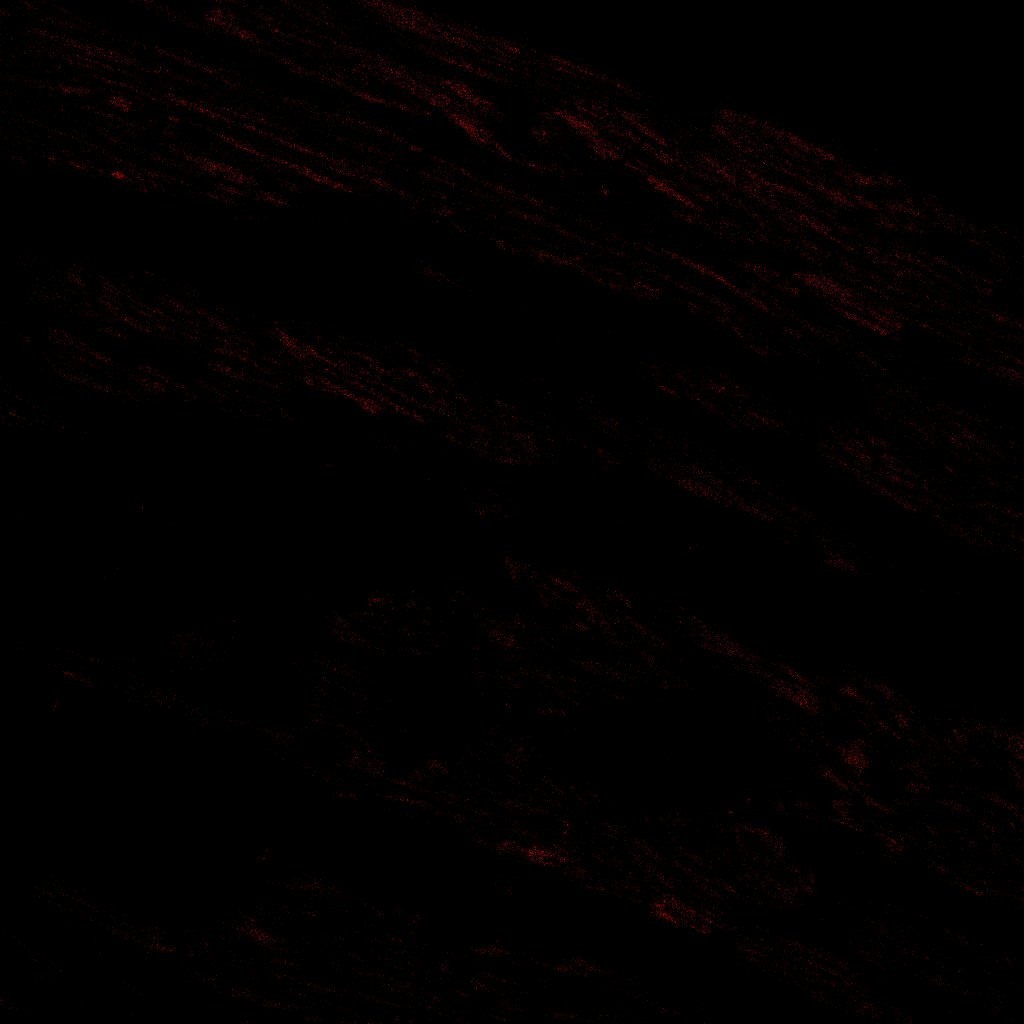

Supplement: Figure 6—figure supplement 2—source data 1. [file elife-98372-fig6-figsupp2-data1.zip › Figure 6-supplementary figure 2-data1/Figure_6-figure supplement_2_source_data_1_Figure_G_Heart_homo_TUNEL(TUNEL).jpg]

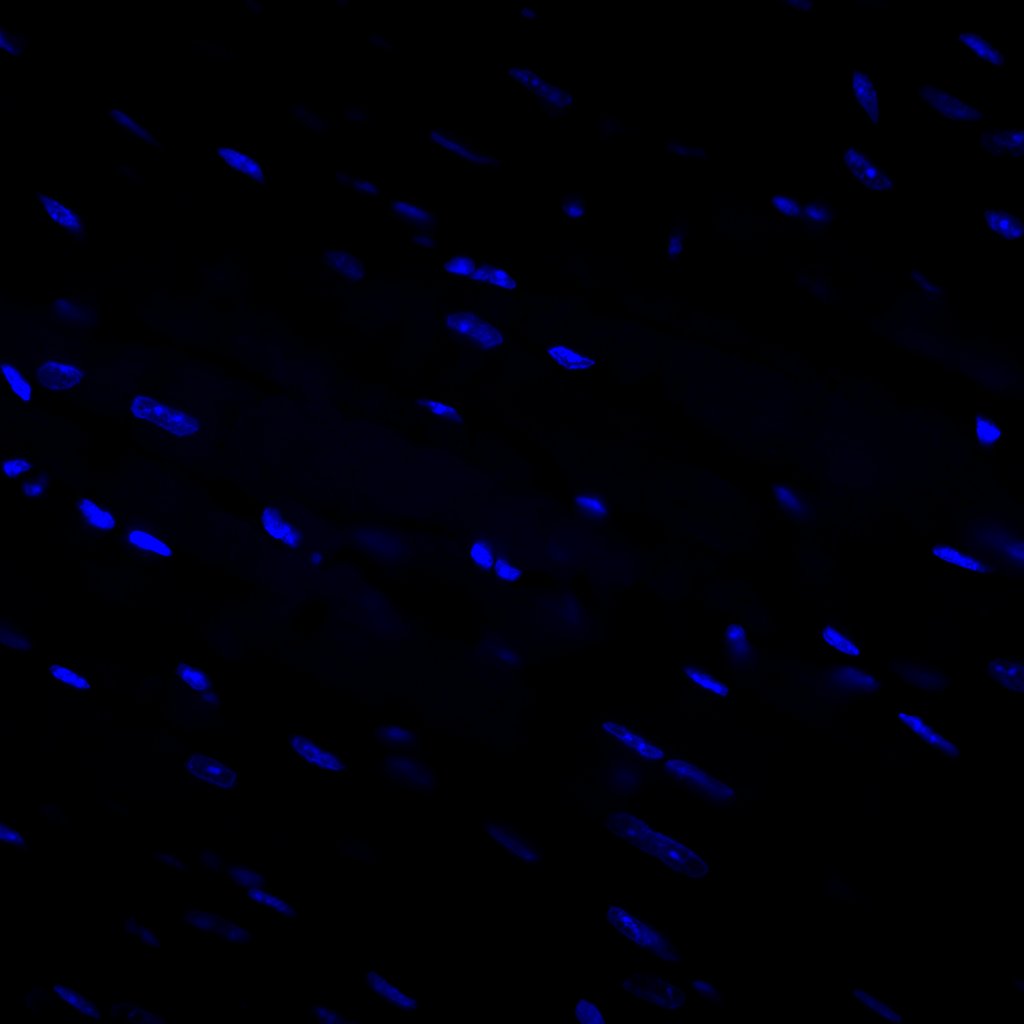

Supplement: Figure 6—figure supplement 2—source data 1. [file elife-98372-fig6-figsupp2-data1.zip › Figure 6-supplementary figure 2-data1/Figure_6-figure supplement_2_source_data_1_Figure_G_Heart_WT_ABT-199_TUNEL(DAPI).jpg]

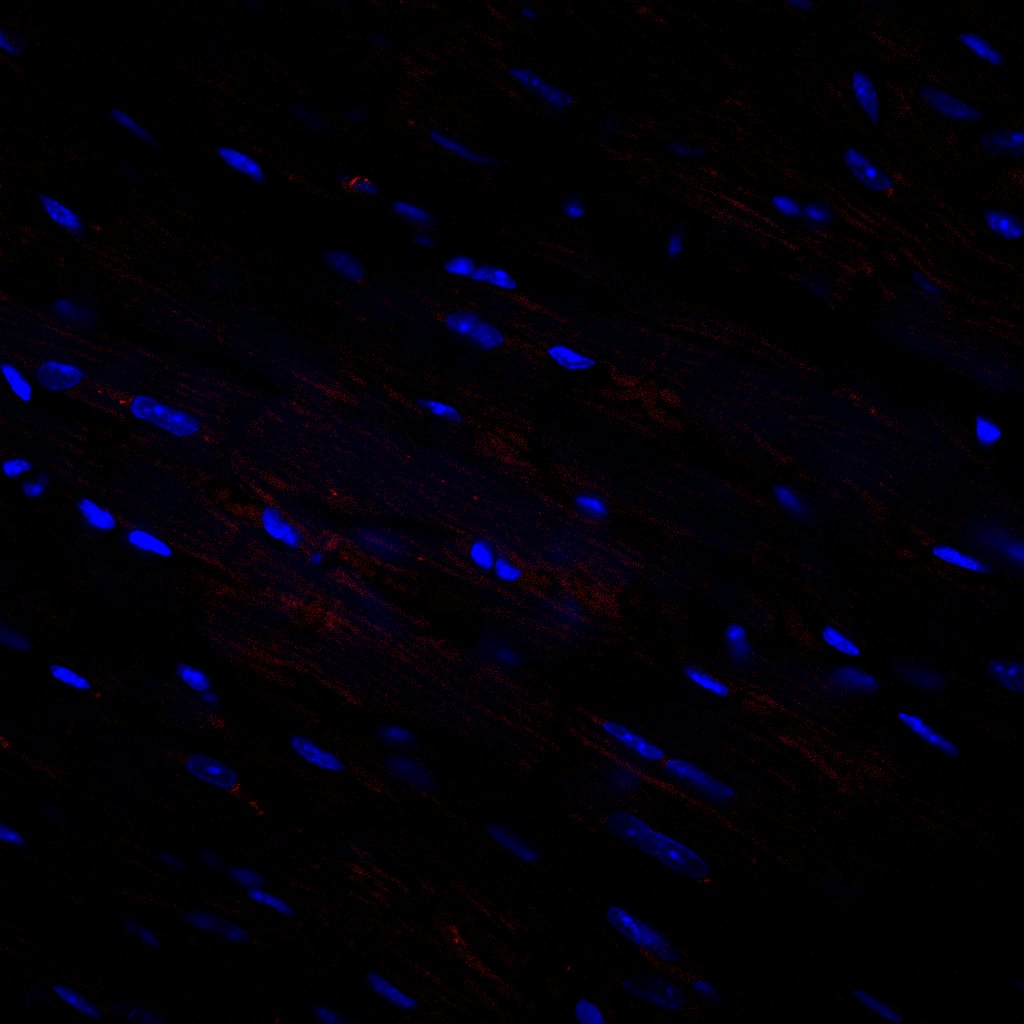

Supplement: Figure 6—figure supplement 2—source data 1. [file elife-98372-fig6-figsupp2-data1.zip › Figure 6-supplementary figure 2-data1/Figure_6-figure supplement_2_source_data_1_Figure_G_Heart_WT_ABT-199_TUNEL(Merge).jpg]

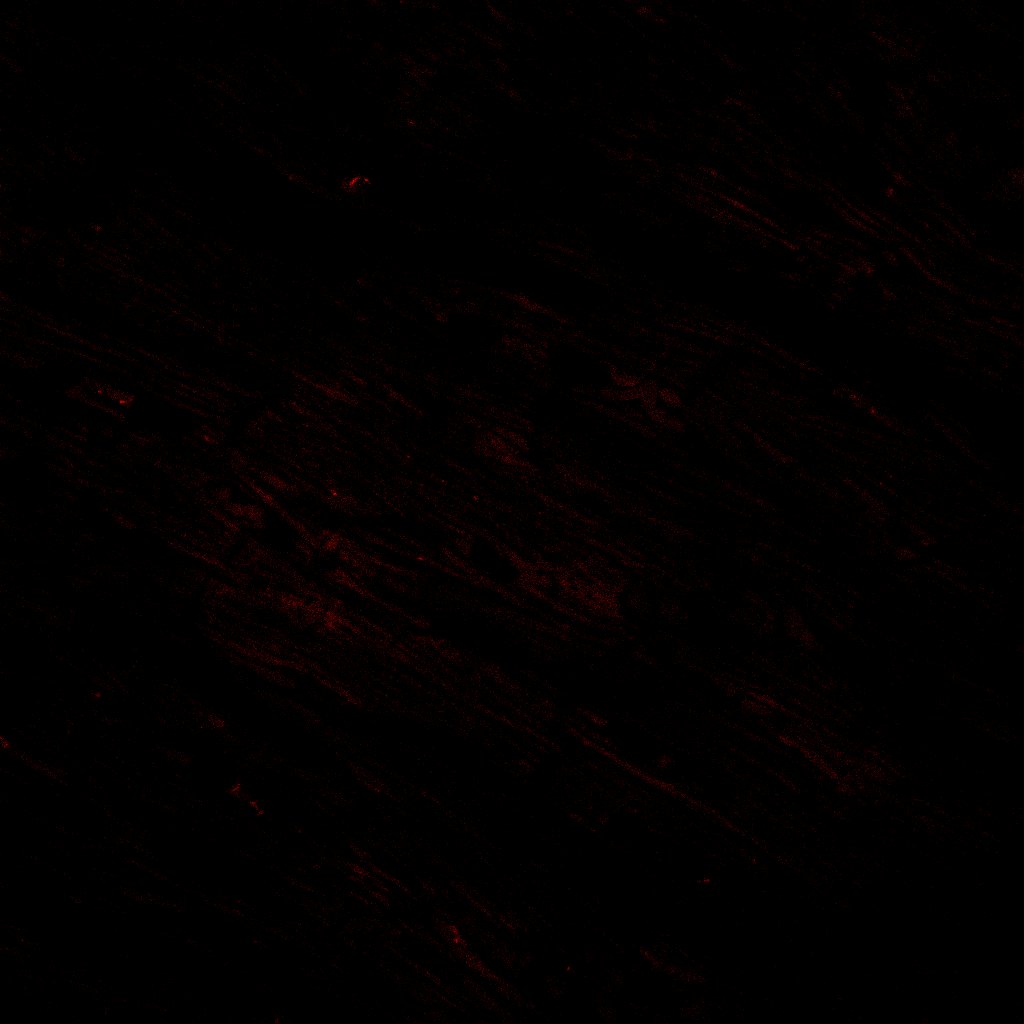

Supplement: Figure 6—figure supplement 2—source data 1. [file elife-98372-fig6-figsupp2-data1.zip › Figure 6-supplementary figure 2-data1/Figure_6-figure supplement_2_source_data_1_Figure_G_Heart_WT_ABT-199_TUNEL(TUNEL).jpg]

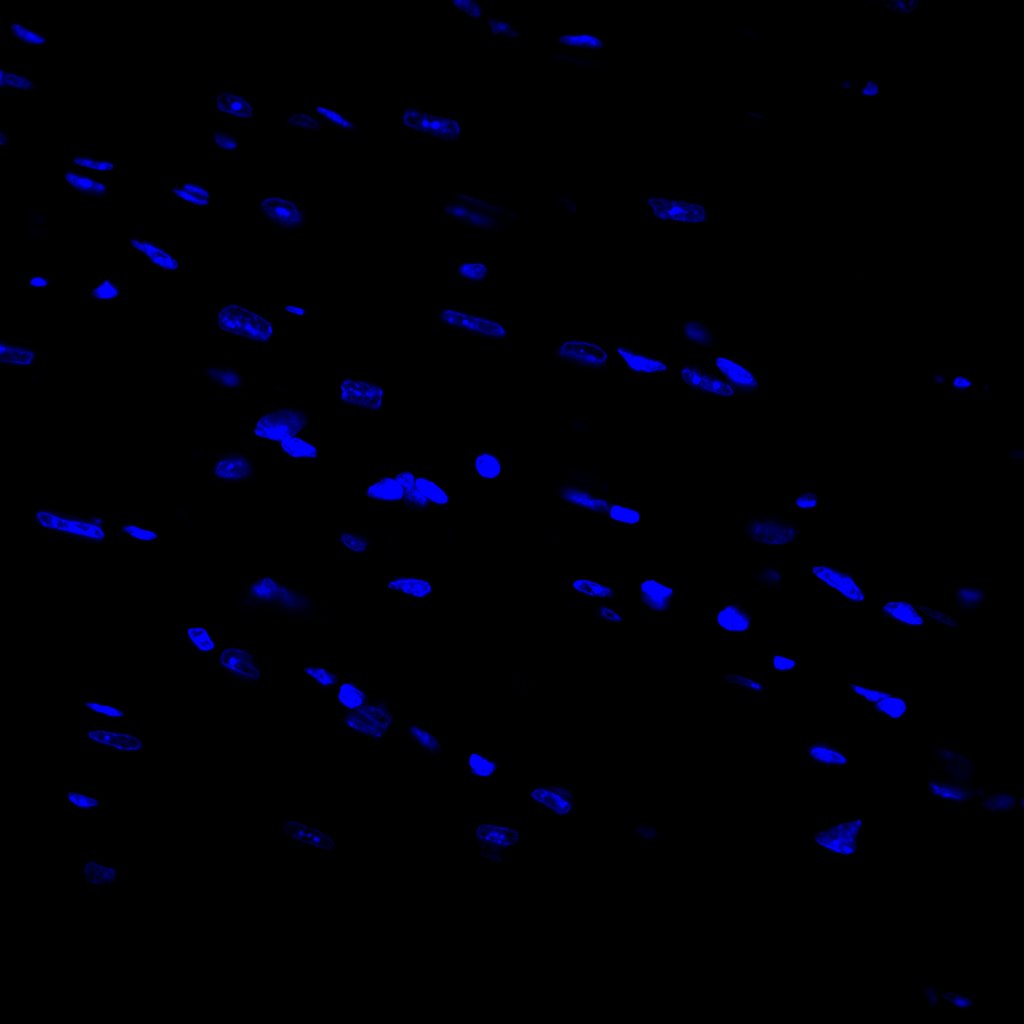

Supplement: Figure 6—figure supplement 2—source data 1. [file elife-98372-fig6-figsupp2-data1.zip › Figure 6-supplementary figure 2-data1/Figure_6-figure supplement_2_source_data_1_Figure_G_Heart_WT_TUNEL(DAPI).jpg]

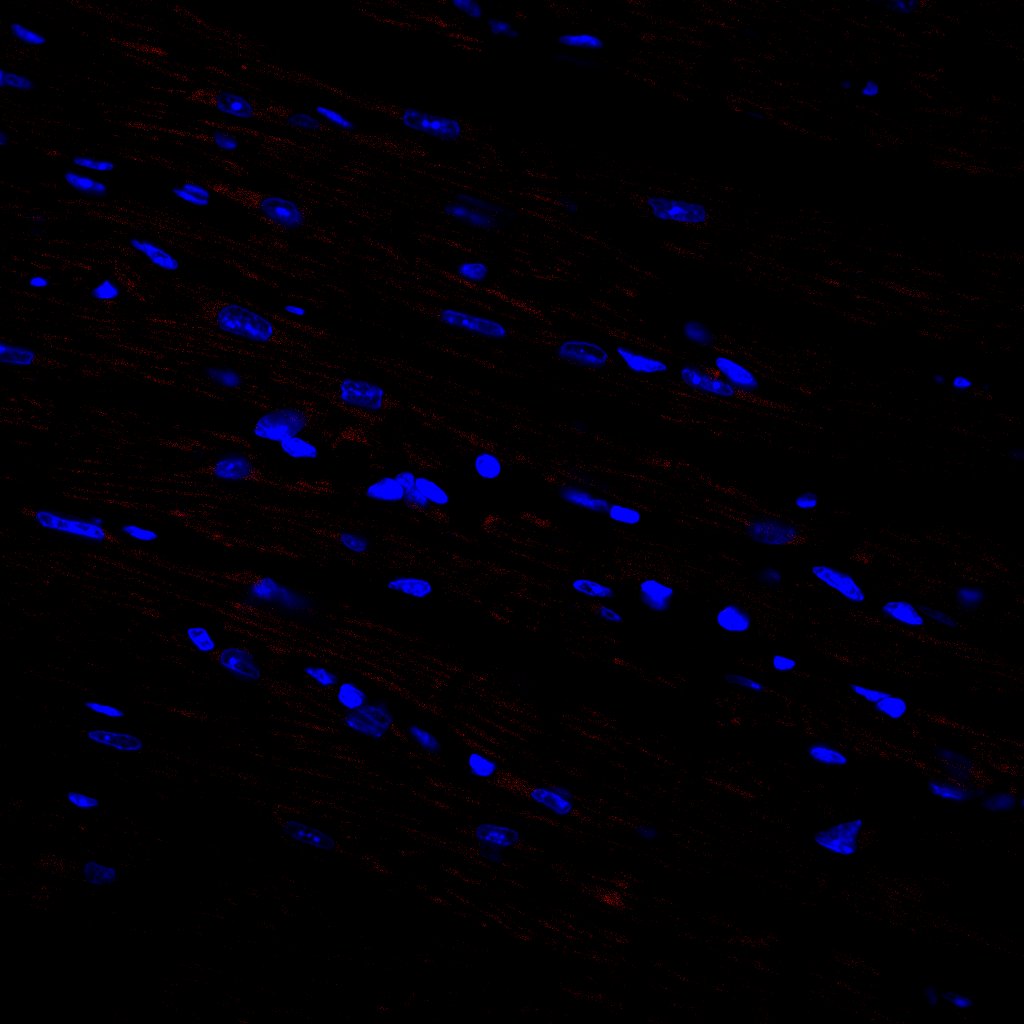

Supplement: Figure 6—figure supplement 2—source data 1. [file elife-98372-fig6-figsupp2-data1.zip › Figure 6-supplementary figure 2-data1/Figure_6-figure supplement_2_source_data_1_Figure_G_Heart_WT_TUNEL(Merge).jpg]

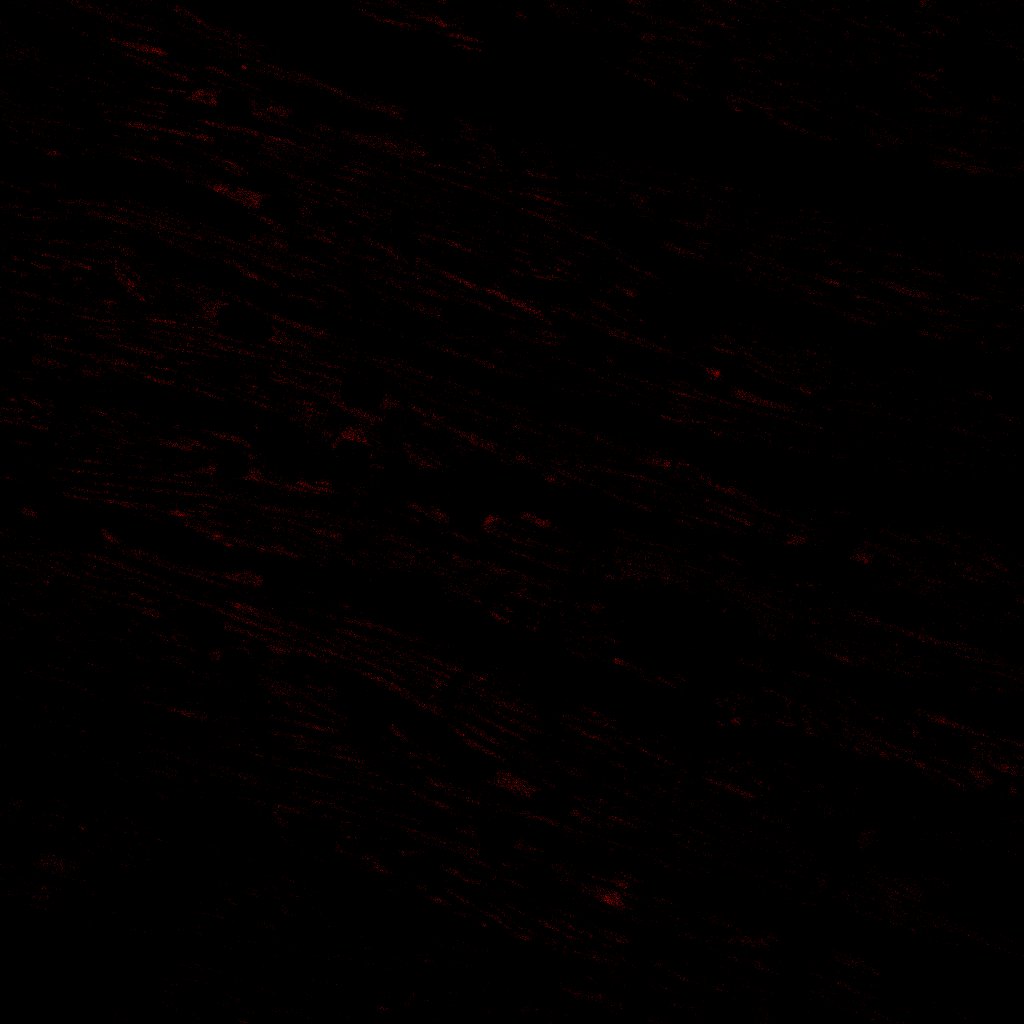

Supplement: Figure 6—figure supplement 2—source data 1. [file elife-98372-fig6-figsupp2-data1.zip › Figure 6-supplementary figure 2-data1/Figure_6-figure supplement_2_source_data_1_Figure_G_Heart_WT_TUNEL(TUNEL).jpg]

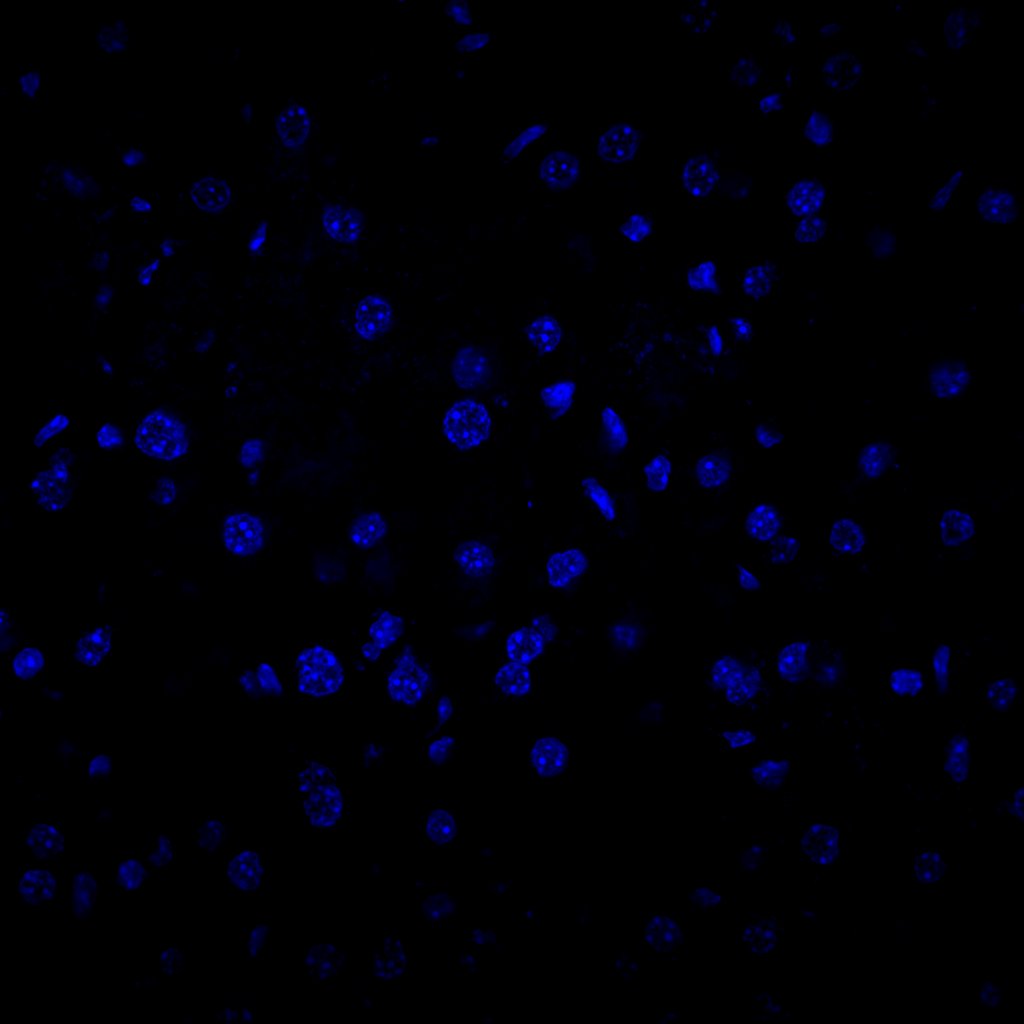

Supplement: Figure 6—figure supplement 2—source data 1. [file elife-98372-fig6-figsupp2-data1.zip › Figure 6-supplementary figure 2-data1/Figure_6-figure supplement_2_source_data_1_Figure_G_Liver_homo_ABT-199_TUNEL(DAPI).jpg]

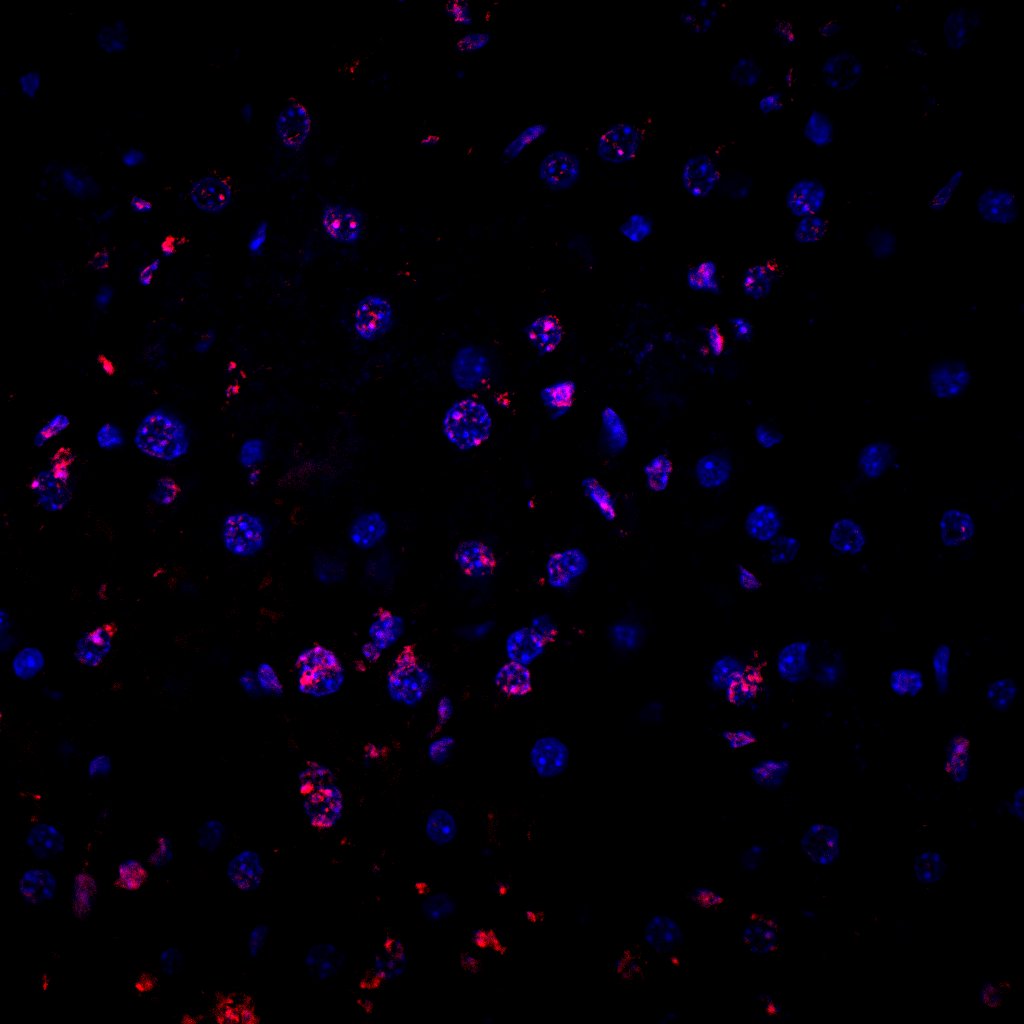

Supplement: Figure 6—figure supplement 2—source data 1. [file elife-98372-fig6-figsupp2-data1.zip › Figure 6-supplementary figure 2-data1/Figure_6-figure supplement_2_source_data_1_Figure_G_Liver_homo_ABT-199_TUNEL(Merge).jpg]

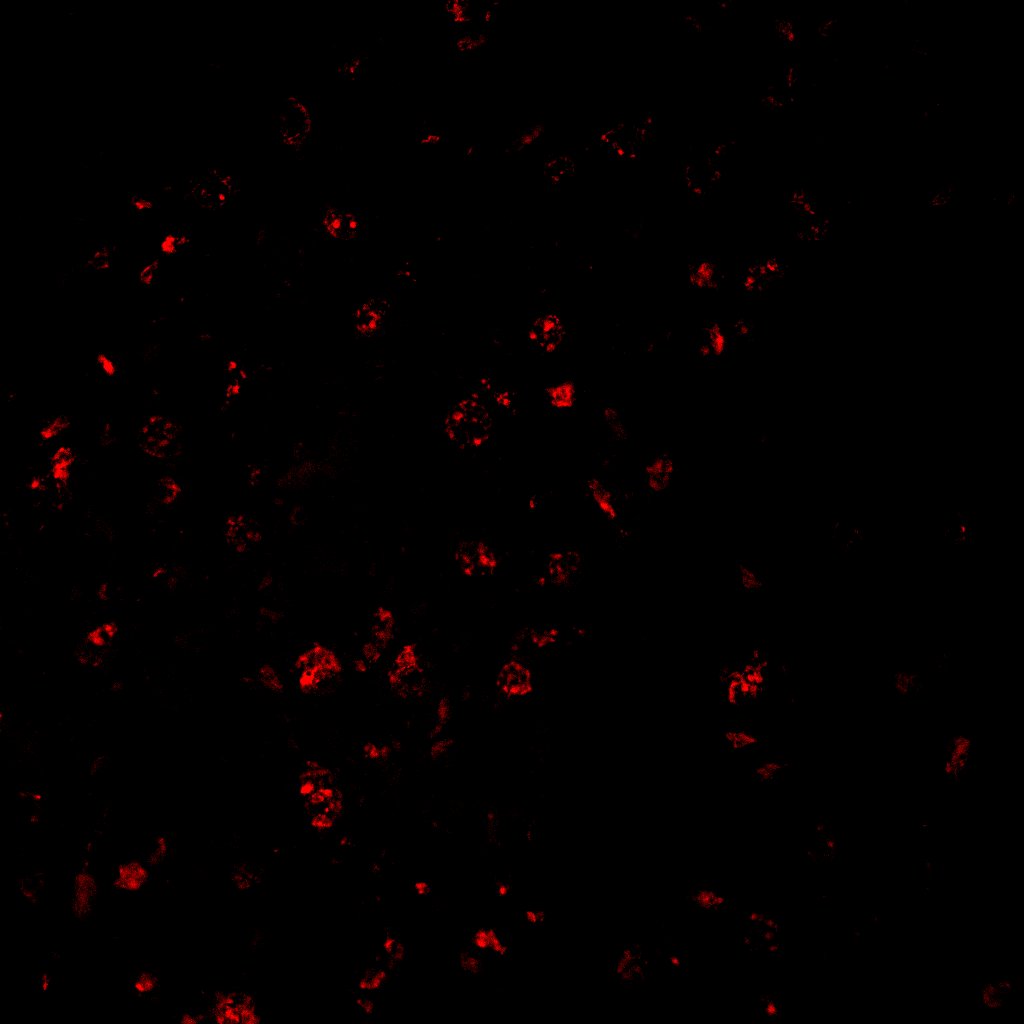

Supplement: Figure 6—figure supplement 2—source data 1. [file elife-98372-fig6-figsupp2-data1.zip › Figure 6-supplementary figure 2-data1/Figure_6-figure supplement_2_source_data_1_Figure_G_Liver_homo_ABT-199_TUNEL(TUNEL).jpg]

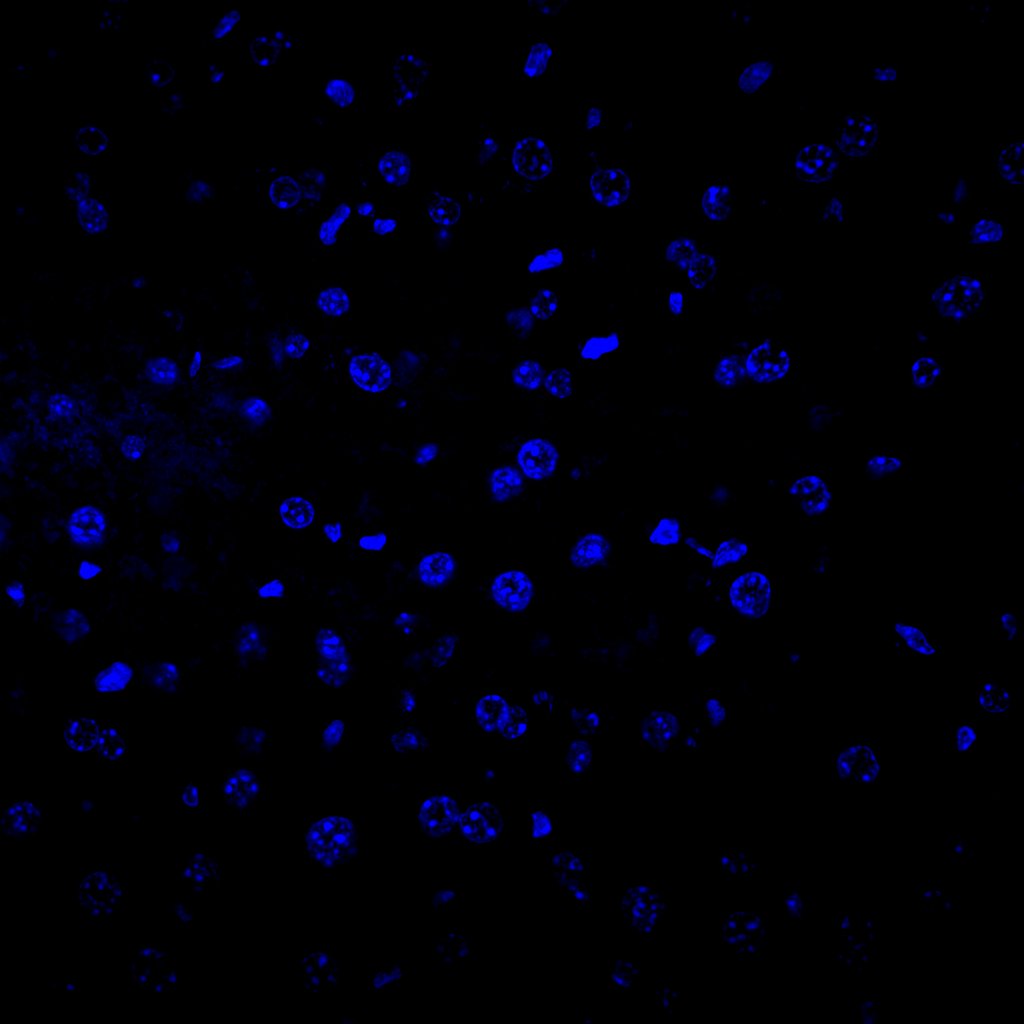

Supplement: Figure 6—figure supplement 2—source data 1. [file elife-98372-fig6-figsupp2-data1.zip › Figure 6-supplementary figure 2-data1/Figure_6-figure supplement_2_source_data_1_Figure_G_Liver_homo_TUNEL(DAPI).jpg]

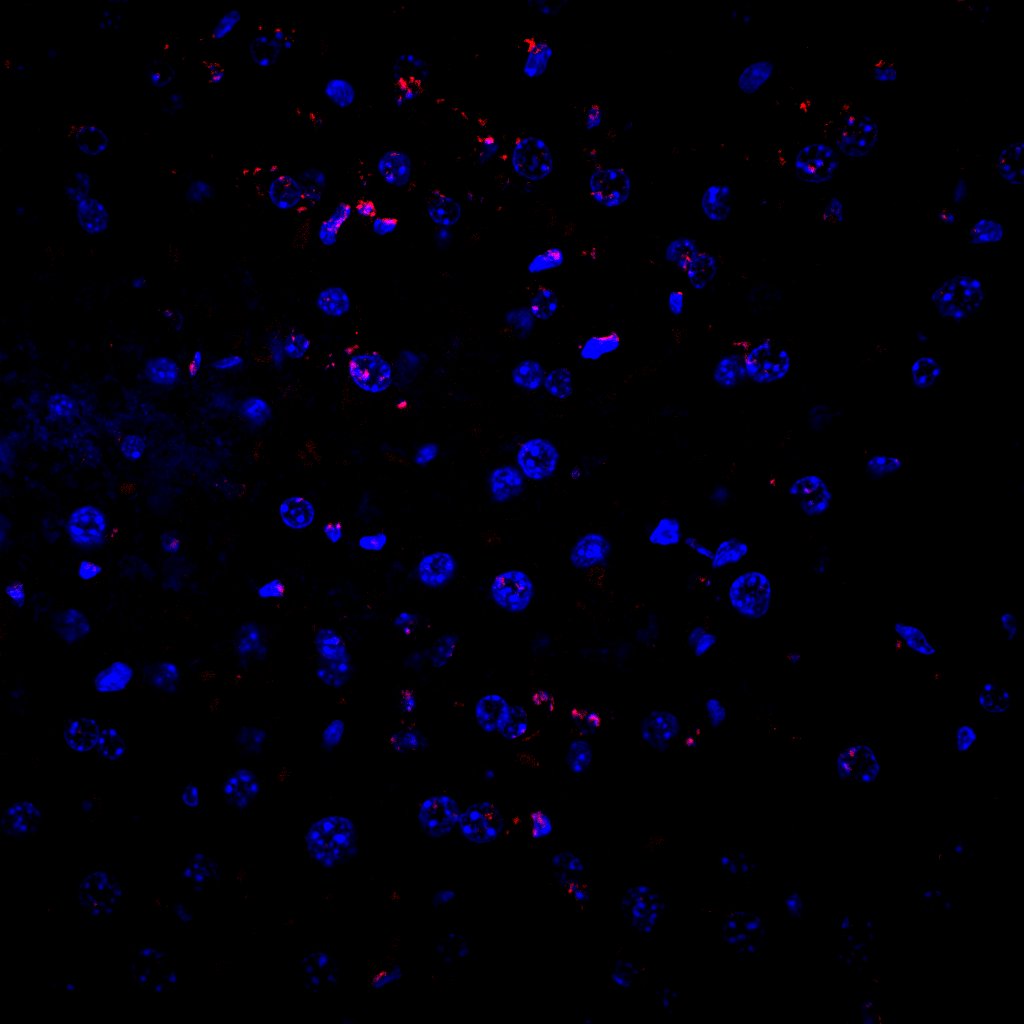

Supplement: Figure 6—figure supplement 2—source data 1. [file elife-98372-fig6-figsupp2-data1.zip › Figure 6-supplementary figure 2-data1/Figure_6-figure supplement_2_source_data_1_Figure_G_Liver_homo_TUNEL(Merge).jpg]

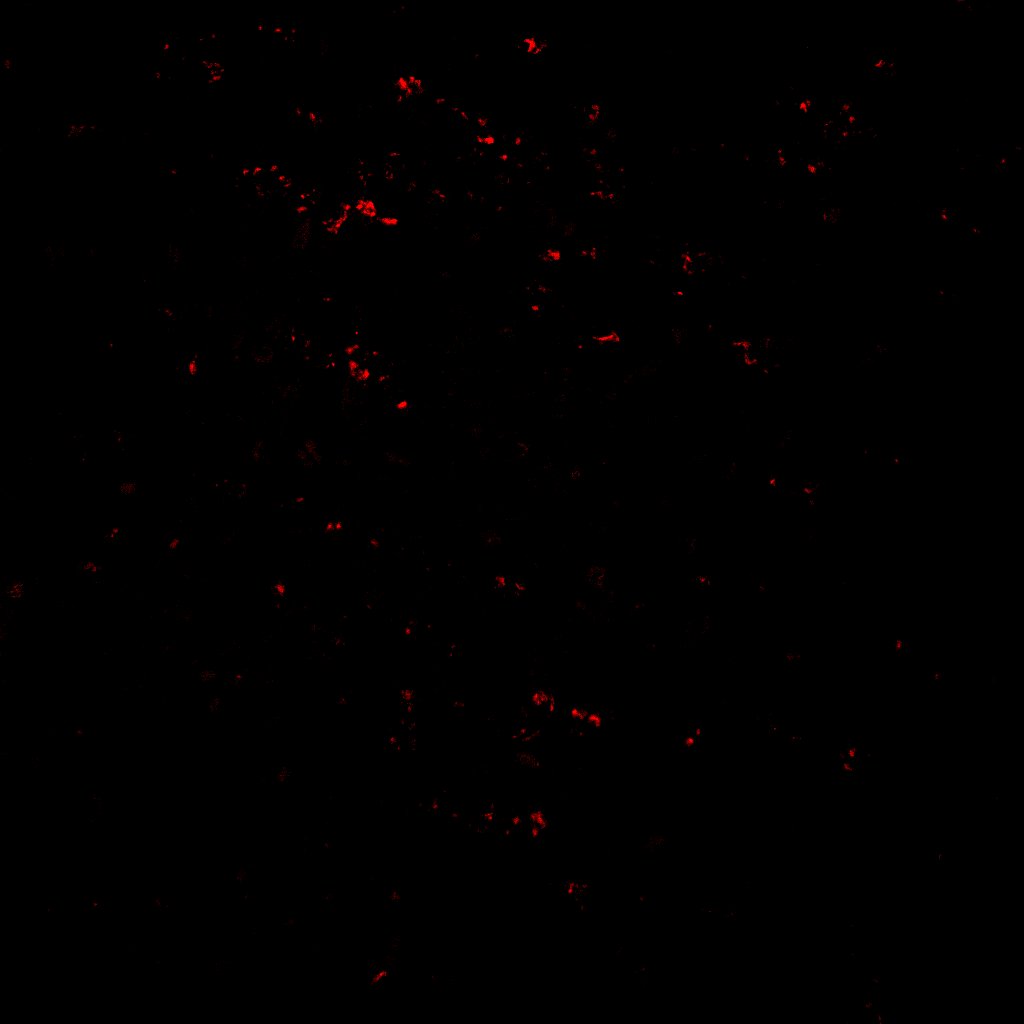

Supplement: Figure 6—figure supplement 2—source data 1. [file elife-98372-fig6-figsupp2-data1.zip › Figure 6-supplementary figure 2-data1/Figure_6-figure supplement_2_source_data_1_Figure_G_Liver_homo_TUNEL(TUNEL).jpg]

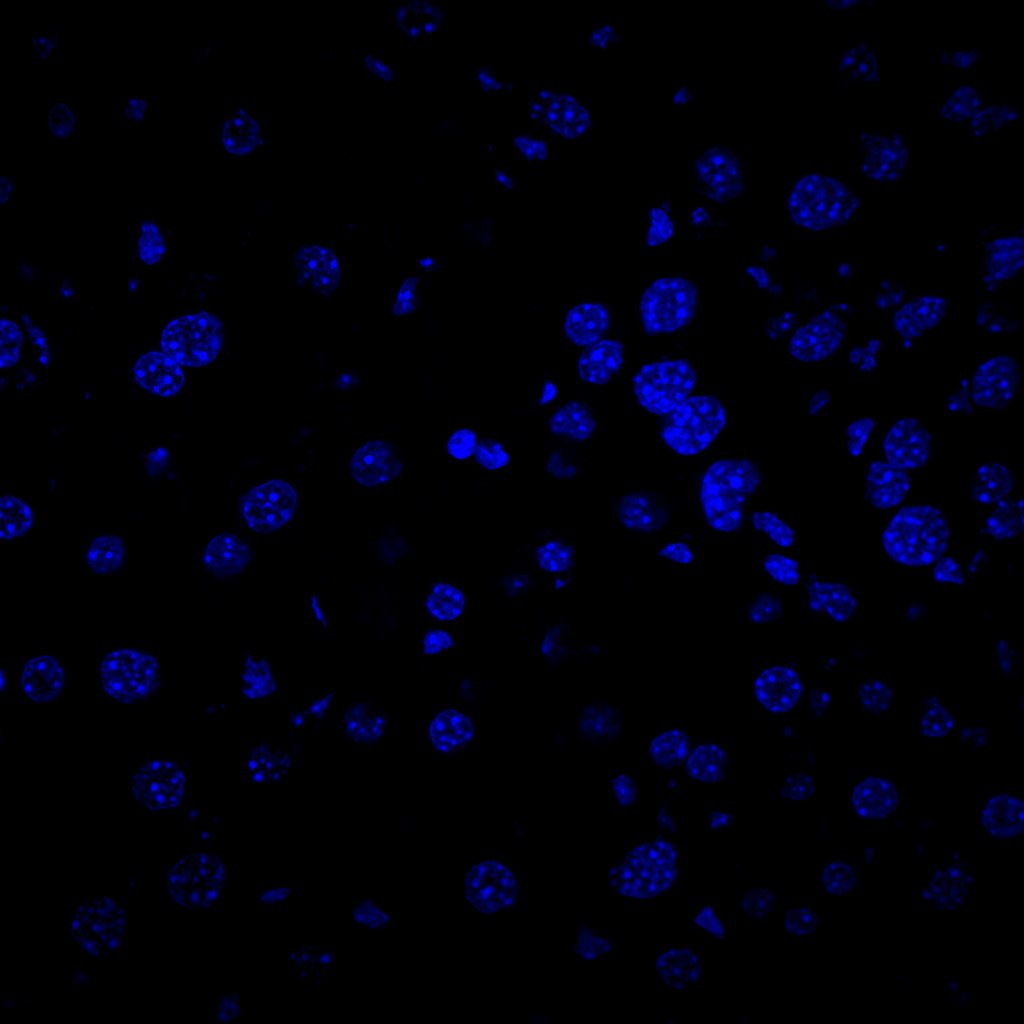

Supplement: Figure 6—figure supplement 2—source data 1. [file elife-98372-fig6-figsupp2-data1.zip › Figure 6-supplementary figure 2-data1/Figure_6-figure supplement_2_source_data_1_Figure_G_Liver_WT_ABT-199_TUNEL(DAPI).jpg]

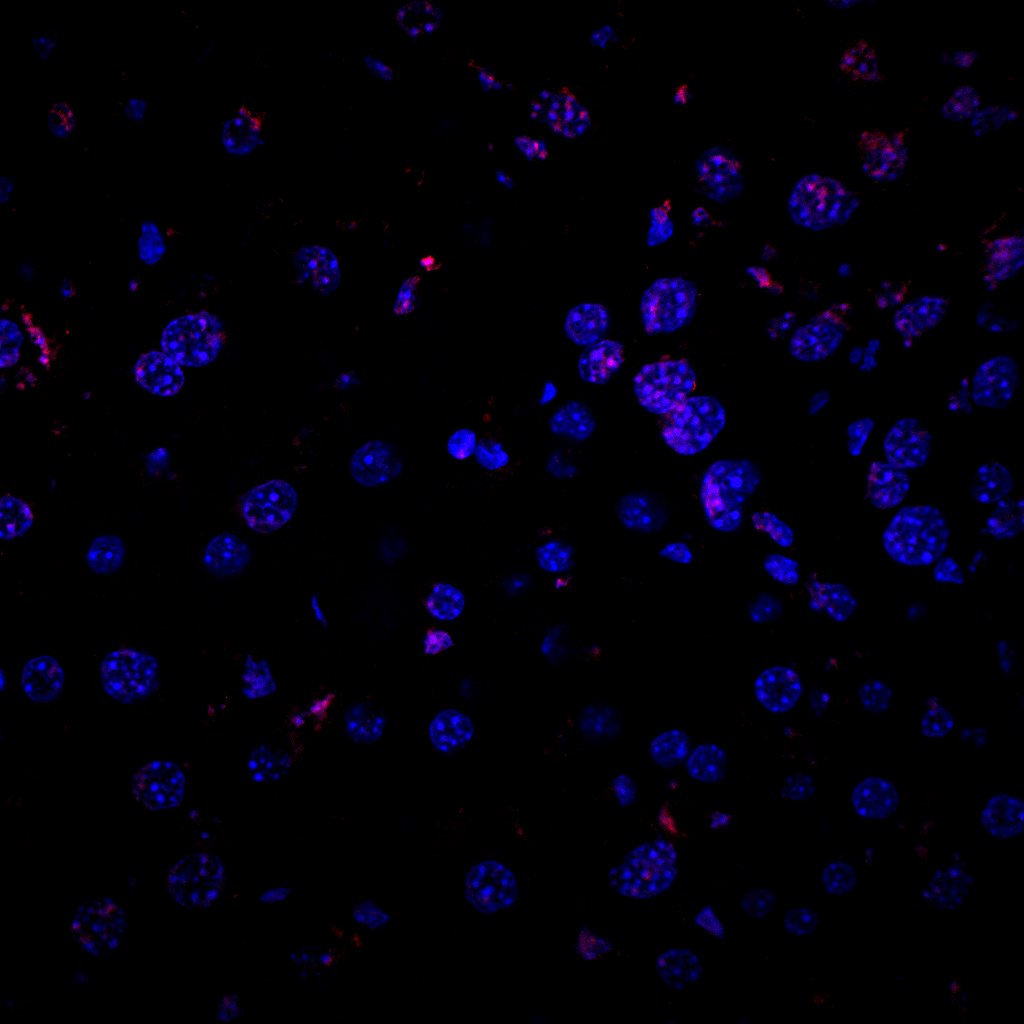

Supplement: Figure 6—figure supplement 2—source data 1. [file elife-98372-fig6-figsupp2-data1.zip › Figure 6-supplementary figure 2-data1/Figure_6-figure supplement_2_source_data_1_Figure_G_Liver_WT_ABT-199_TUNEL(Merge).jpg]

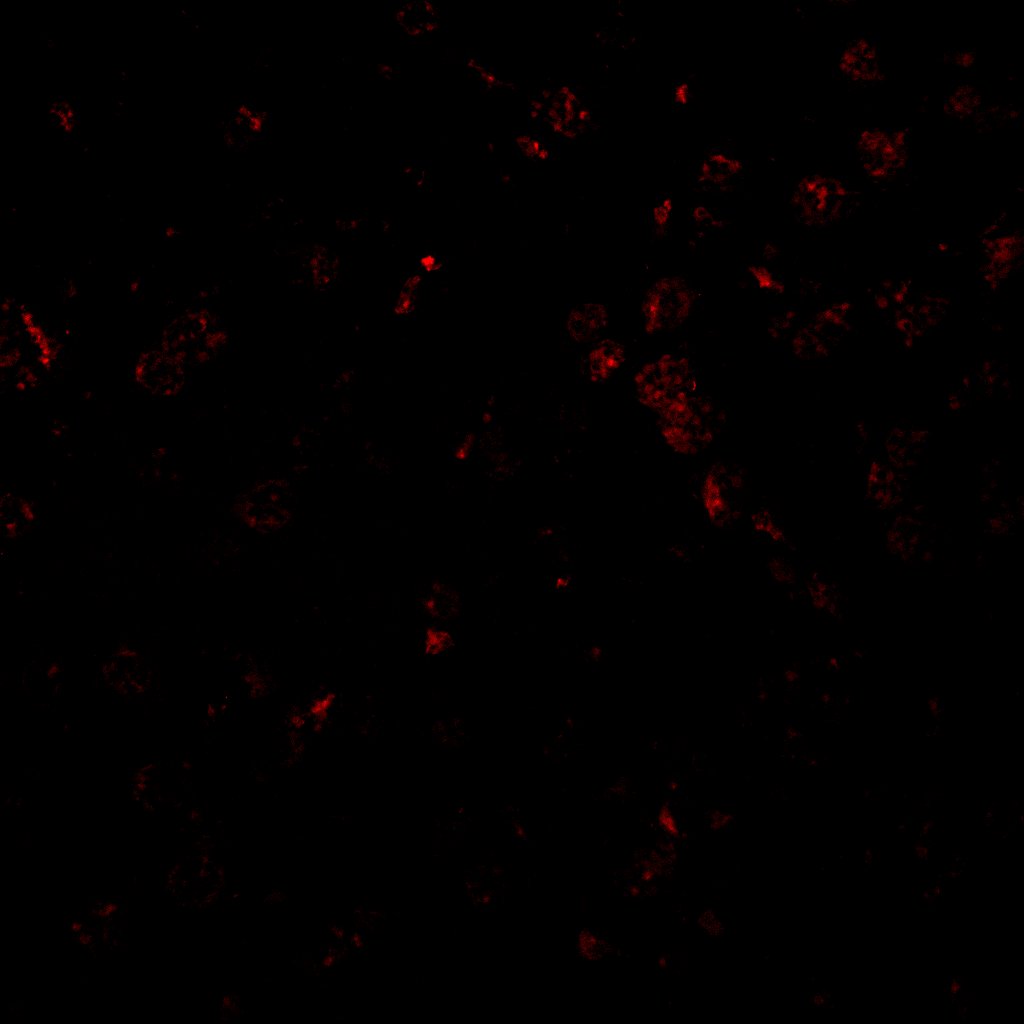

Supplement: Figure 6—figure supplement 2—source data 1. [file elife-98372-fig6-figsupp2-data1.zip › Figure 6-supplementary figure 2-data1/Figure_6-figure supplement_2_source_data_1_Figure_G_Liver_WT_ABT-199_TUNEL(TUNEL).jpg]

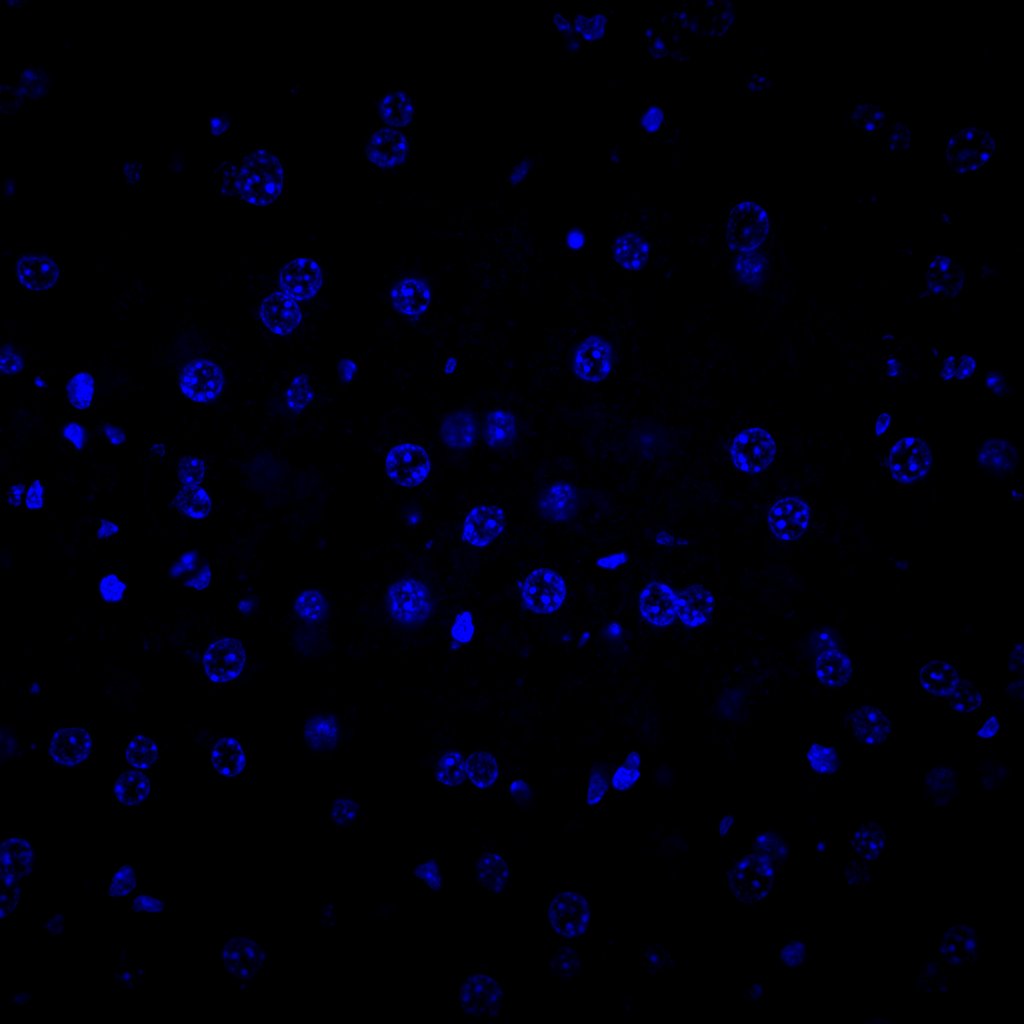

Supplement: Figure 6—figure supplement 2—source data 1. [file elife-98372-fig6-figsupp2-data1.zip › Figure 6-supplementary figure 2-data1/Figure_6-figure supplement_2_source_data_1_Figure_G_Liver_WT_TUNEL(DAPI).jpg]

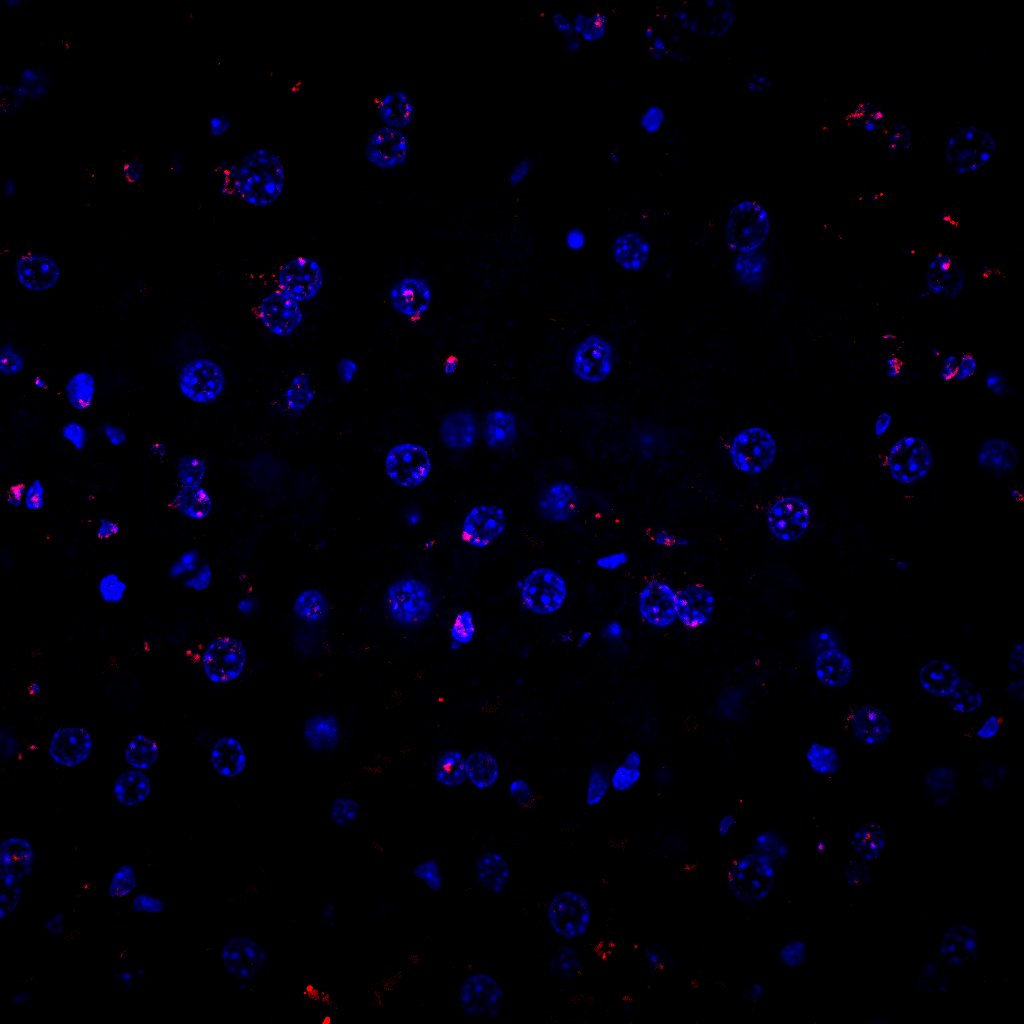

Supplement: Figure 6—figure supplement 2—source data 1. [file elife-98372-fig6-figsupp2-data1.zip › Figure 6-supplementary figure 2-data1/Figure_6-figure supplement_2_source_data_1_Figure_G_Liver_WT_TUNEL(Merge).jpg]

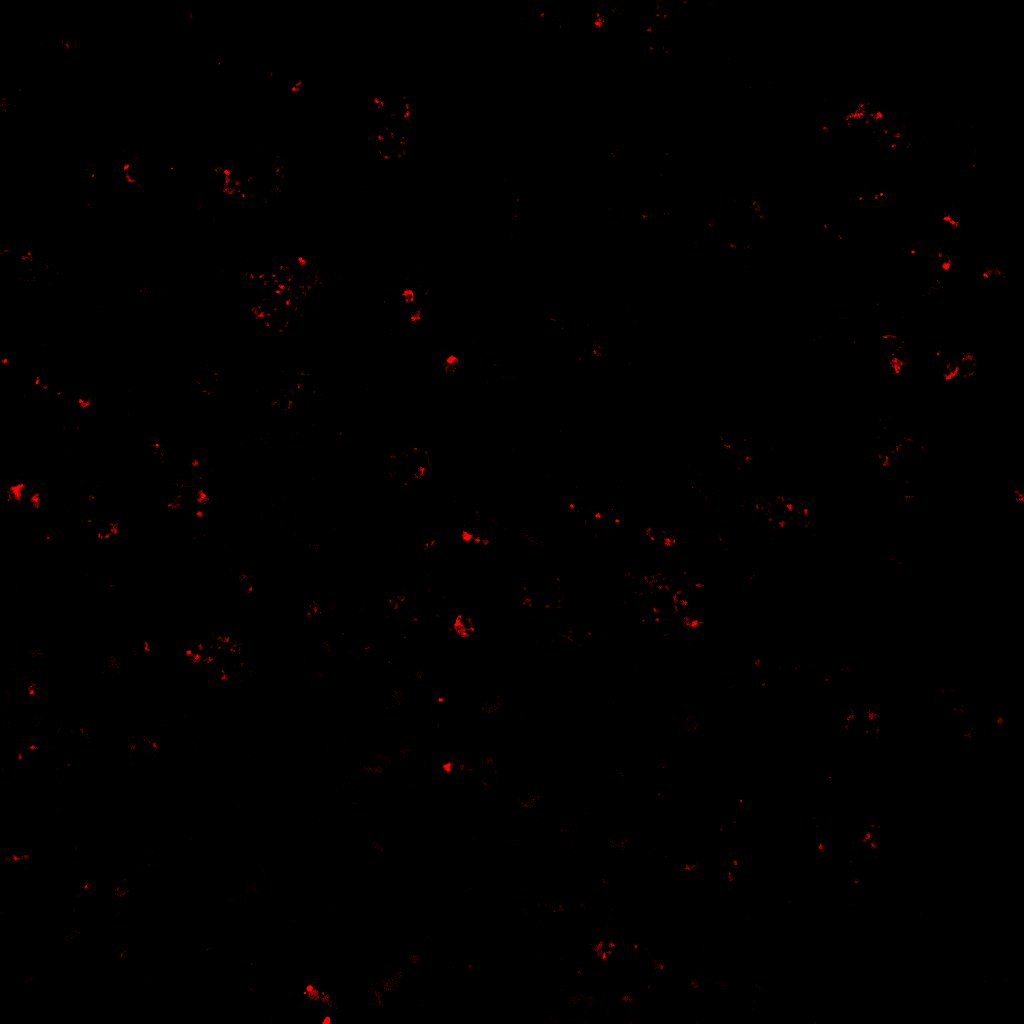

Supplement: Figure 6—figure supplement 2—source data 1. [file elife-98372-fig6-figsupp2-data1.zip › Figure 6-supplementary figure 2-data1/Figure_6-figure supplement_2_source_data_1_Figure_G_Liver_WT_TUNEL(TUNEL).jpg]

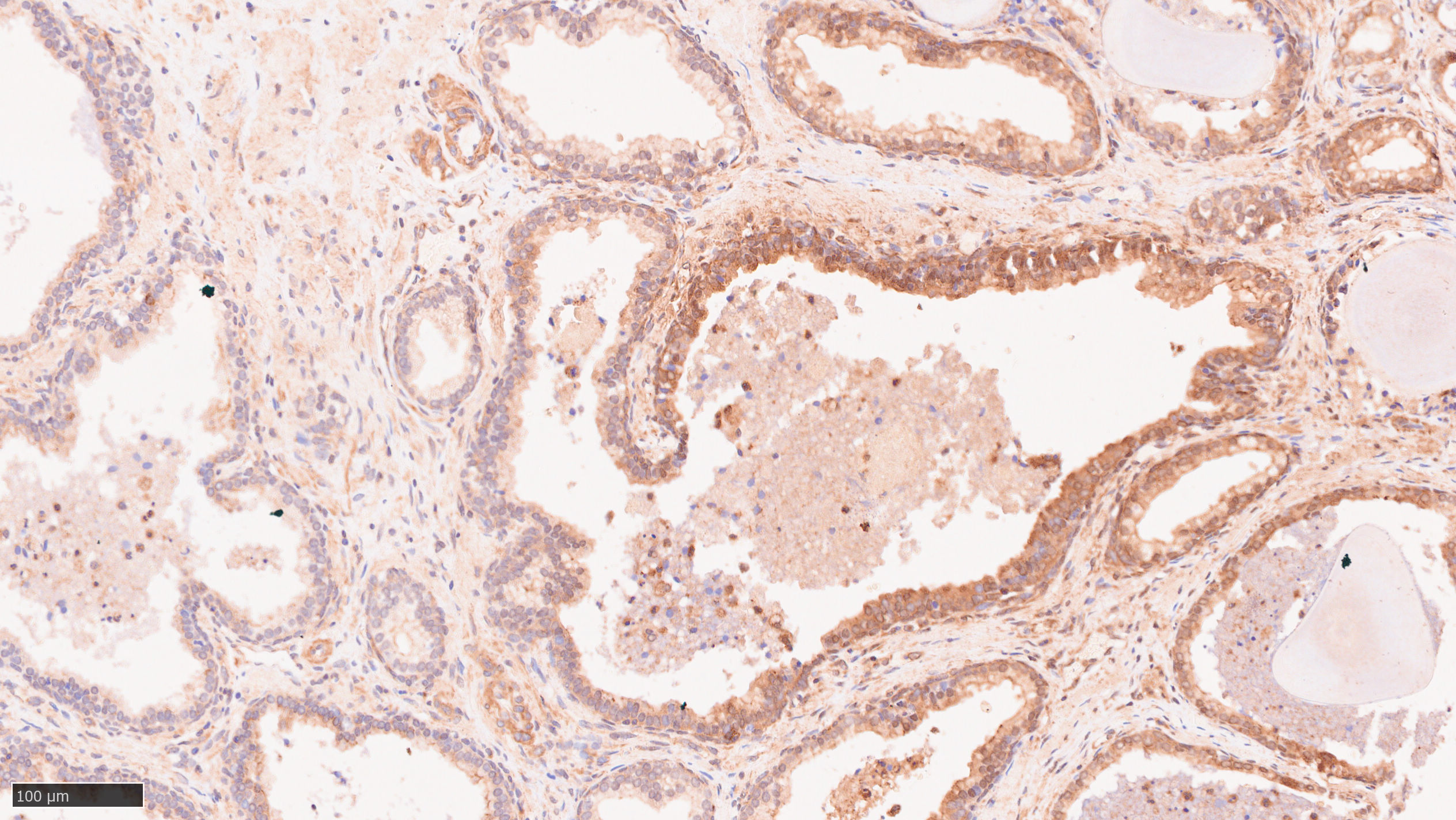

Supplement: Figure 7—source data 1. [file elife-98372-fig7-data1.zip › Figure 7-data1/Figure_7-source_data_1-Figure_7K_High expression.jpg]

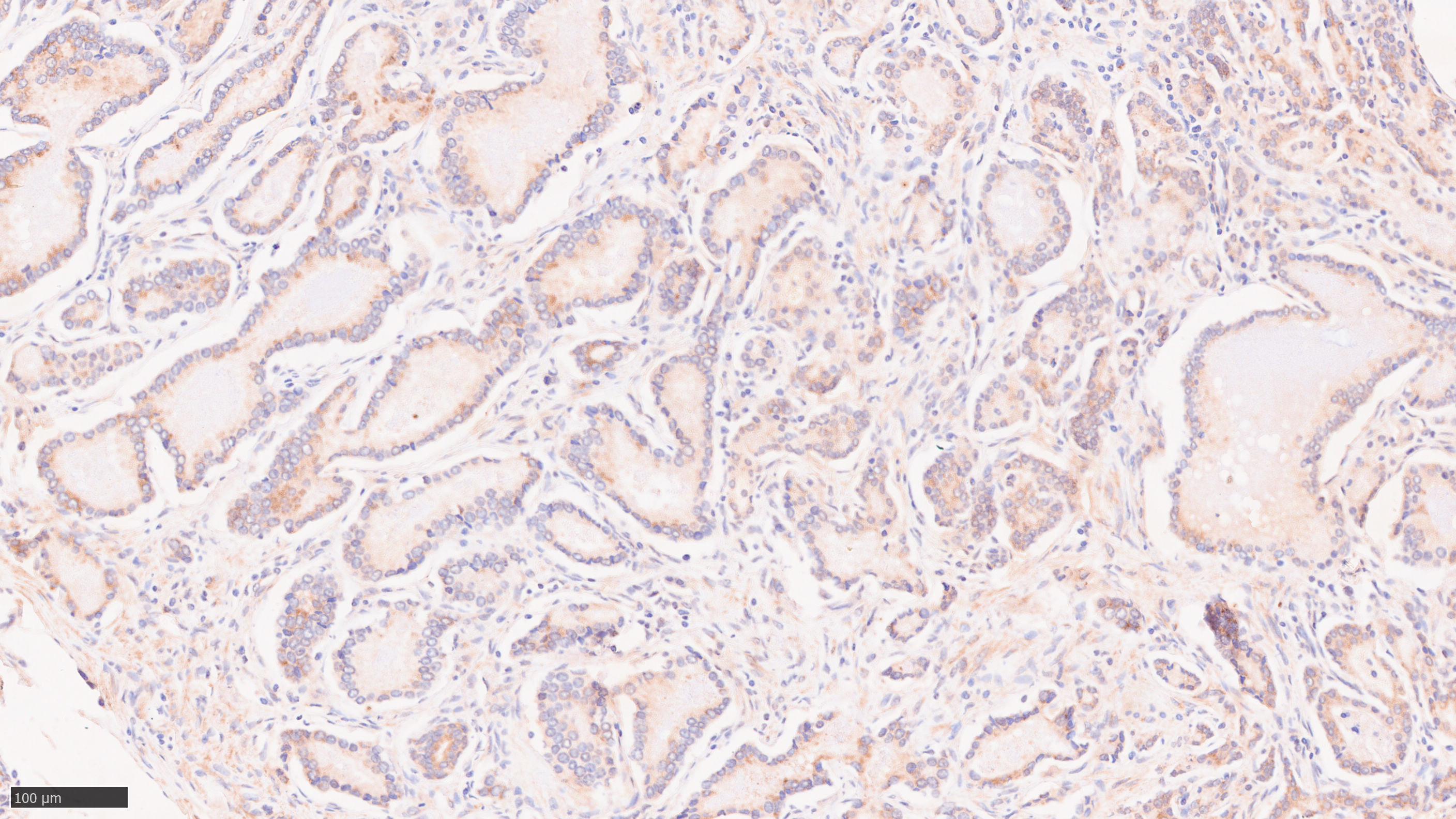

Supplement: Figure 7—source data 1. [file elife-98372-fig7-data1.zip › Figure 7-data1/Figure_7-source_data_1-Figure_7K_low expression.jpg]

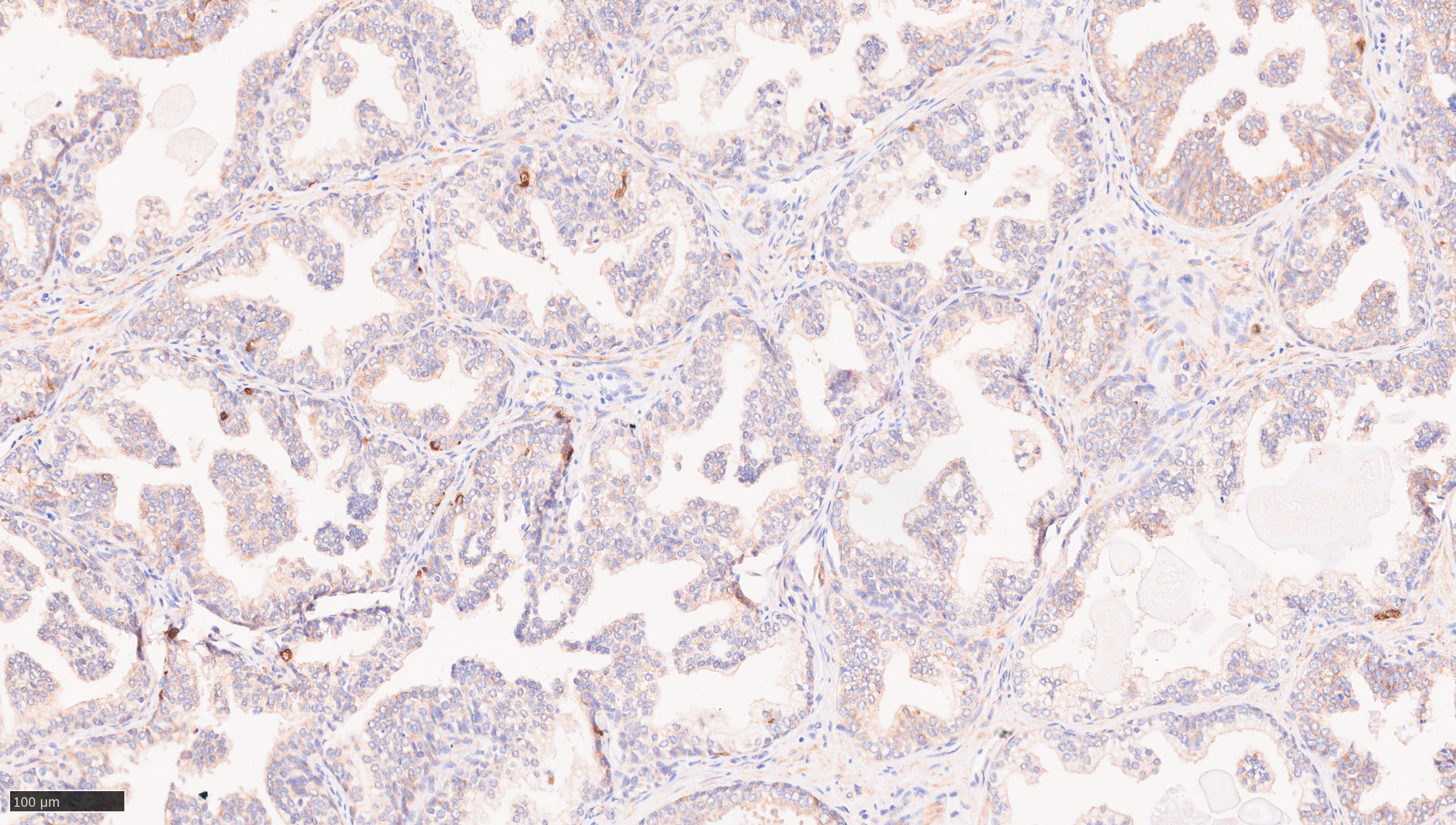

Supplement: Figure 7—source data 1. [file elife-98372-fig7-data1.zip › Figure 7-data1/Figure_7-source_data_1-Figure_7K_negative.jpg]

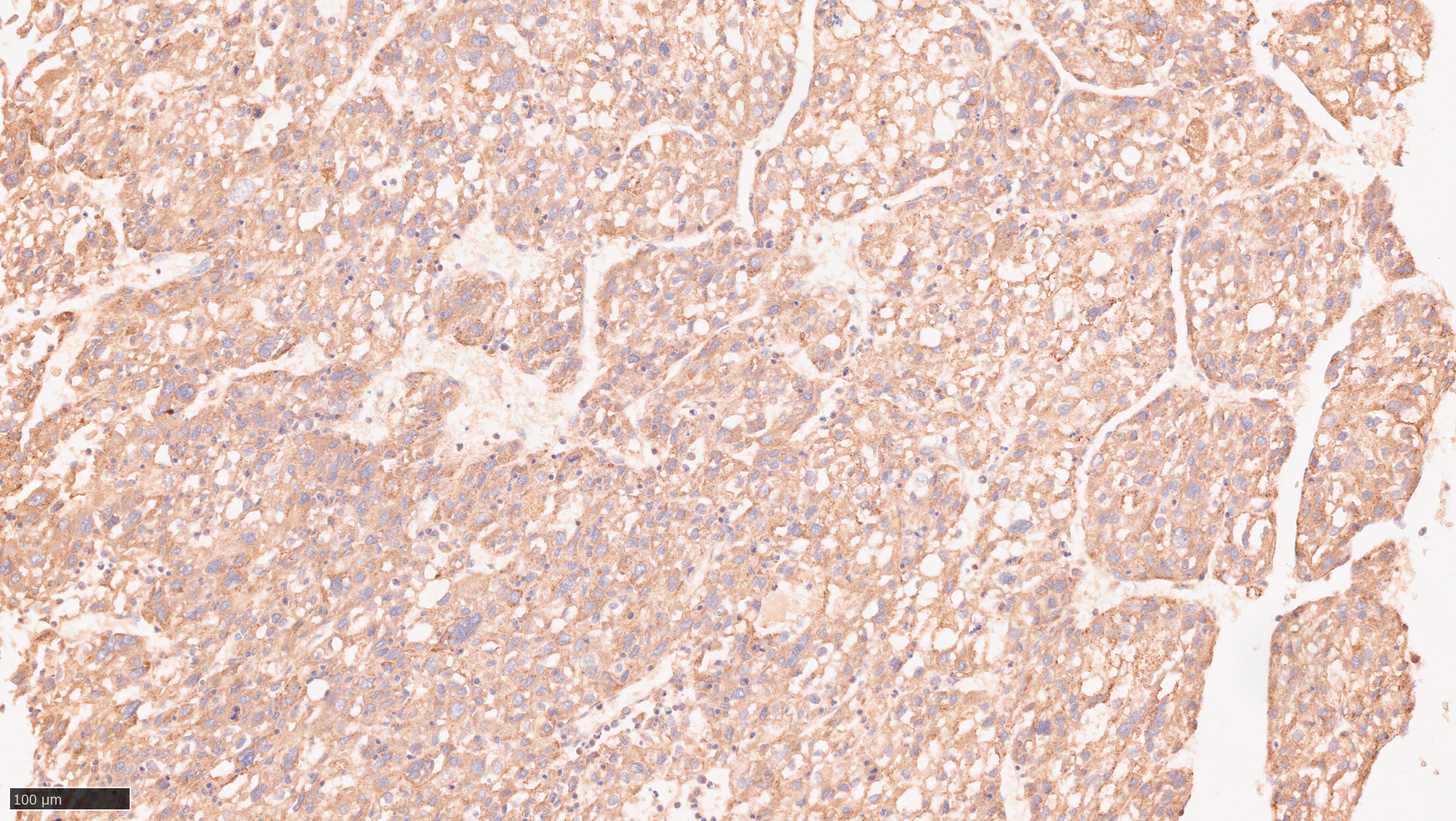

Supplement: Figure 7—source data 1. [file elife-98372-fig7-data1.zip › Figure 7-data1/Figure_7-source_data_1-Figure_7M_High expression.jpg]

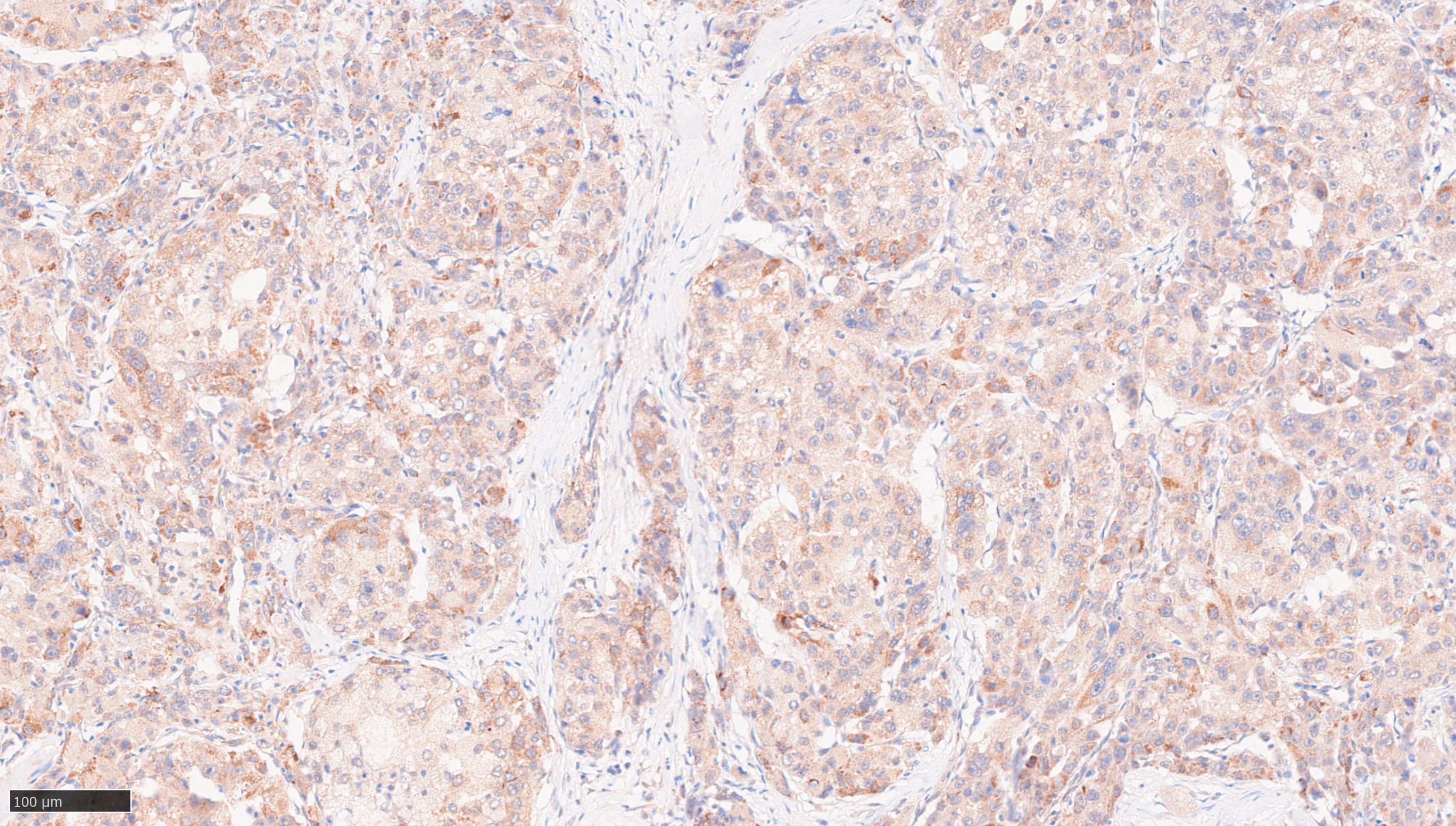

Supplement: Figure 7—source data 1. [file elife-98372-fig7-data1.zip › Figure 7-data1/Figure_7-source_data_1-Figure_7M_low expression.jpg]

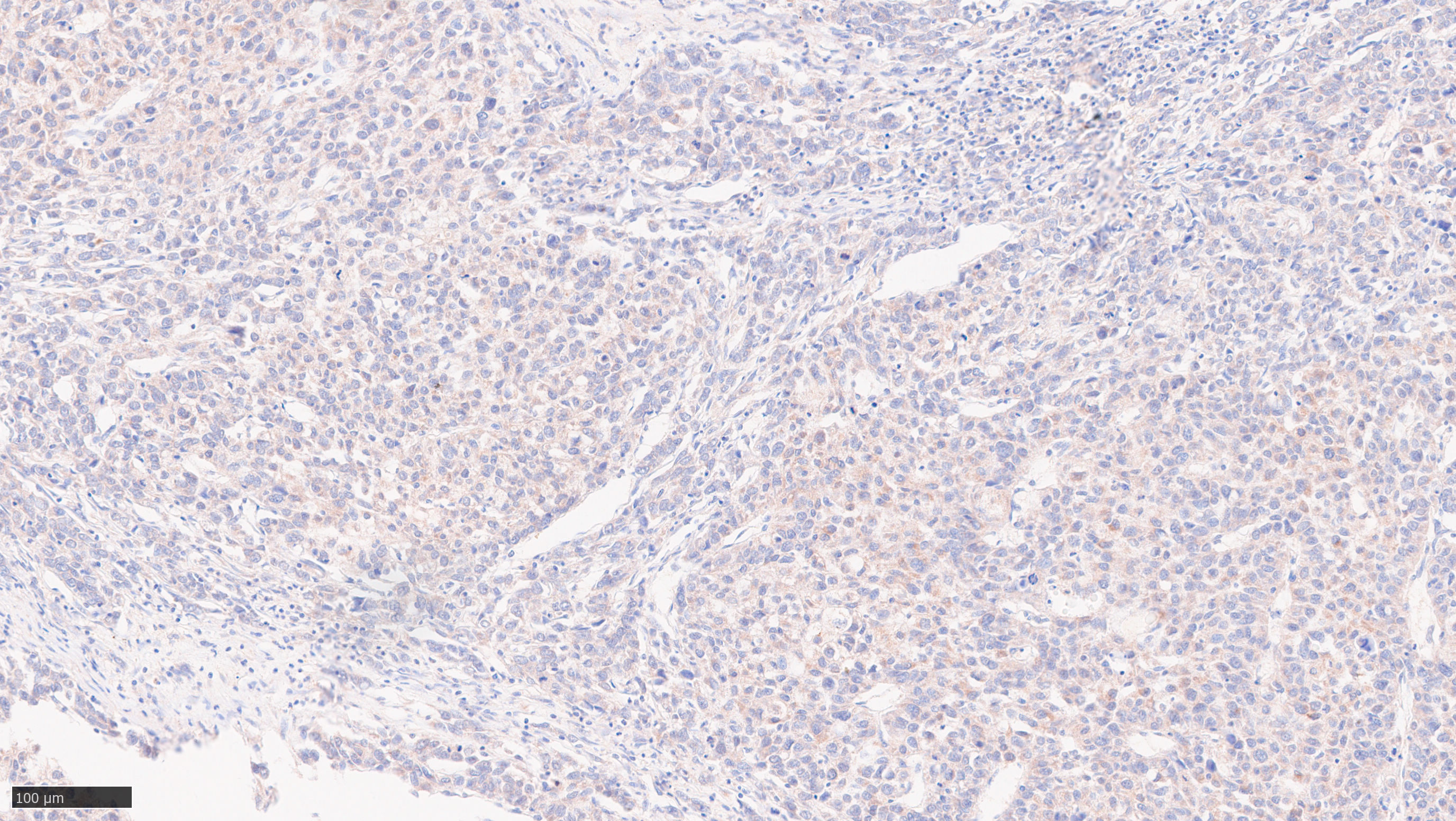

Supplement: Figure 7—source data 1. [file elife-98372-fig7-data1.zip › Figure 7-data1/Figure_7-source_data_1-Figure_7M_negative_expression.jpg]
